# Investigating the use of ultrasonography for the antenatal diagnosis of structural congenital anomalies in low-income and middle-income countries: a systematic review

**DOI:** 10.1136/bmjpo-2020-000684

**Published:** 2020-08-20

**Authors:** Stephanie Michele Goley, Sidonie Sakula-Barry, Nana Adofo-Ansong, Laurence Isaaya Ntawunga, Maame Tekyiwa Botchway, Ann Horton Kelly, Naomi Wright

**Affiliations:** 1 Department of Global Health & Social Medicine, King’s College London, London, UK; 2 Health Information Department, World Cancer Research Fund, London, UK; 3 Department of Paediatrics, Mafikeng Provincial Hospital, Mafikeng, South Africa; 4 Rwanda Spina Bifida and Hydrocephalus Relief, University of Rwanda, Kigali, Rwanda; 5 Department of Paediatric Surgery, University of the Witwatersrand, Johannesburg-Braamfontein, Gauteng, South Africa; 6 King’s Centre for Global Health and Health Partnerships, King’s College London, London, UK

**Keywords:** screening, neonatology, congenital abnorm

## Abstract

**Background:**

Congenital anomalies are the fifth leading cause of under-5 mortality globally. The greatest burden is faced by those in low/middle-income countries (LMICs), where over 95% of deaths occur. Many of these deaths may be preventable through antenatal diagnosis and early intervention. This systematic literature review investigates the use of antenatal ultrasound to diagnose congenital anomalies and improve the health outcomes of infants in LMICs.

**Methods:**

A systematic literature review was conducted using three search strings: (1) structural congenital anomalies; (2) LMICs; and (3) antenatal diagnosis. The search was conducted on the following databases: Medline, Embase, PubMed and the Cochrane Library. Title, abstract and full-text screening was undertaken in duplicate by two reviewers independently. Consensus among the wider authorship was sought for discrepancies. The primary analysis focused on the availability and effectiveness of antenatal ultrasound for diagnosing structural congenital anomalies. Secondary outcomes included neonatal morbidity and mortality, termination rates, referral rates for further antenatal care and training level of the ultrasonographer. Relevant policy data were sought.

**Results:**

The search produced 4062 articles; 97 were included in the review. The median percentage of women receiving an antenatal ultrasound examination was 50.0% in African studies and 90.7% in Asian studies (range 6.8%–98.8%). Median detection rates were: 16.7% Africa, 34.3% South America, 34.7% Asia and 47.3% Europe (range 0%–100%). The training level of the ultrasound provider may affect detection rates. Four articles compared morbidity and mortality outcomes, with inconclusive results. Significant variations in termination rates were found (0%–98.3%). No articles addressed referral rates.

**Conclusion:**

Antenatal detection of congenital anomalies remains highly variable across LMICs and is particularly low in sub-Saharan Africa. Further research is required to investigate the role of antenatal diagnosis for improving survival from congenital anomalies in LMICs.

**PROSPERO registration number:**

CRD42019105620.

## Introduction

Congenital anomalies are one of the leading causes of neonatal morbidity and mortality globally. The greatest burden of disease is faced by those in low/middle-income countries (LMICs), as 94% of congenital anomalies occur in these regions.^1^ Congenital anomalies comprise 9% of the total global burden of surgical disease and account for 57.7 million disability-adjusted life years lost annually across the globe.^2^ Recent estimates suggest that approximately 303 000 neonates die annually from congenital anomalies before reaching just 4 weeks of age.^3^ However, many experts believe that this is an underestimate, due to a lack of congenital anomaly registries and some neonates dying without a diagnosis or inclusion within current statistics.

The WHO defines congenital anomalies as either structural or functional abnormalities which occur during intrauterine development.^3^ Structural anomalies are physical abnormalities that occur when the organs or skeletal structure are improperly formed. These can often be detected on ultrasound antenatally and are the focus of this review. Some common structural congenital anomalies include heart defects, cleft lip and palate, neural tube defects, limb deformities and abdominal wall defects. Many structural anomalies require immediate surgical intervention at birth to avoid death or preventable disability. In such cases, antenatal diagnosis permits delivery at a centre where the appropriate surgical care can be provided on delivery, for example, gastroschisis where the intestines protrude through a hole in the abdominal wall at birth. In high-income countries (HICs), where the majority of cases are antenatally diagnosed, mortality is less than 5%, while in many LMICs, with limited antenatal diagnosis, the mortality rate can be as high as 100%.^4–6^

The use of ultrasound technology in LMICs has significantly increased in recent years, as ultrasound machines have become more compact, transportable and affordable.^7^ Yet, a great number of congenital anomalies that can be detected antenatally via ultrasound go undiagnosed. Factors identified as barriers to effective antenatal ultrasound include limited training, equipment shortages, faulty ultrasound equipment and lack of maintenance services.^7^ In recent years, higher global priority has been given to neonatal health. Sustainable development goal 3.2 aims to end all preventable under-5 deaths and reduce neonatal mortality in every country to 12 per 1000 live births.^8^ In 2010, the WHO released the 63rd World Health Assembly Report on Birth Defects, recommending ‘prevention whenever possible, to implement screening programs and to provide care and ongoing support to children with birth defects and their families’.^9^

To develop a better understanding of antenatal ultrasound provision in LMICs, this study aimed to systematically investigate the availability and effectiveness of antenatal ultrasound in the diagnosis of structural congenital anomalies in LMICs. It further aimed to evaluate the effects of antenatal ultrasound diagnosis on mortality and morbidity outcomes, termination rates and referral for further antenatal care and management planning. Additionally, it assessed the level of training of ultrasonographers undertaking antenatal scans and relevant antenatal ultrasound policies in LMICs. This information is vital to help clarify the existing disparities in antenatal ultrasound provision and the potential benefits for improved health outcomes.

## Methodology

Preferred Reporting Items for Systematic Reviews and Meta-Analyses guidelines have been followed when conducting this systematic review (online supplementary file 1).^10 11^ A protocol for this systematic review was published in *BMJ Paediatrics Open*.^12^

10.1136/bmjpo-2020-000684.supp1 Supplementary data

### Search strategy

A search was conducted using three search strings: (1) structural congenital anomalies, (2) LMICs and (3) antenatal diagnosis using ultrasound (online supplementary file 2). Using the Ovid programme, an electronic database search was conducted on Medline, Embase, PubMed and the Cochrane Library. These searches were filtered to only include studies with human subjects. An example of the search in Medline can be found in online supplementary file 3. Only fetuses with a structural congenital anomaly as listed in search string 1 were included. Only studies from LMICs were included; these were limited to the English language. Studies with less than five patients were excluded. A further search was conducted on the WHO website to identify relevant grey literature, particularly related to antenatal ultrasound policy. The following terms were searched in the WHO Reproductive Health Library: ultrasound, ultrasonography, congenital anomalies, congenital abnormalities, congenital anomaly, congenital abnormality, birth defect, antenatal detection, prenatal detection, antenatal diagnosis and prenatal diagnosis. Following the search of each term, the results were expanded using a snowball strategy to optimise the inclusion of all relevant data.

10.1136/bmjpo-2020-000684.supp2 Supplementary data

10.1136/bmjpo-2020-000684.supp3 Supplementary data

### Study design

All forms of evidence-based research were included. This includes systematic reviews, meta-analyses, randomised controlled trials, descriptive observational studies, case-control studies, cohort studies and case series.

### Methodological quality

Although the researchers intended to use the Cochrane Risk of Bias for Non-Randomised Studies of Interventions and the revised tool to assess Risk of Bias in Randomised Trials V.2.0 to evaluate methodological quality, the majority of studies included in this systematic review were not interventional studies. Overall, the data were heterogenous and descriptive in nature, which was not suitable for existing quality assessment tools.

### Study screening

References produced from the search results were added to EndNote V.X8 and duplicates were removed. The articles were then uploaded to Covidence and screened in duplicate. Articles that did not meet the study criteria were removed.

### Data extraction and synthesis

Data extraction was undertaken by the principal investigator. The data extraction table can be found in online supplementary file 4. The primary analysis focused on the availability and effectiveness of antenatal ultrasound for structural congenital anomalies. Secondary outcomes included neonatal morbidity and mortality, termination rates and referral rates for further antenatal care. The results are presented in tables and descriptive statistics (range and median) have been calculated regionally.

10.1136/bmjpo-2020-000684.supp4 Supplementary data

### Patient and public involvement

Given that this is a systematic literature review, there was no patient or public involvement for the collection of data and literature review. Public involvement will be important for prioritising antenatal ultrasound on the political agenda and improving antenatal care programmes. To disseminate the results of this study, international charities and organisations involving structural congenital anomalies will be approached to assist in circulation.

## Results

### Study screening

The search produced 4062 articles. Of these, 745 duplicates were removed. The remaining 3317 articles underwent abstract and title review by two independent reviewers. Of the 3317 articles screened, 2826 were excluded. Four hundred and ninety-one articles were then reviewed by two independent reviewers in full text. At this stage, 316 articles were excluded; 73 for non-English language (online supplementary file 5).

10.1136/bmjpo-2020-000684.supp5 Supplementary data

**Figure 1:**
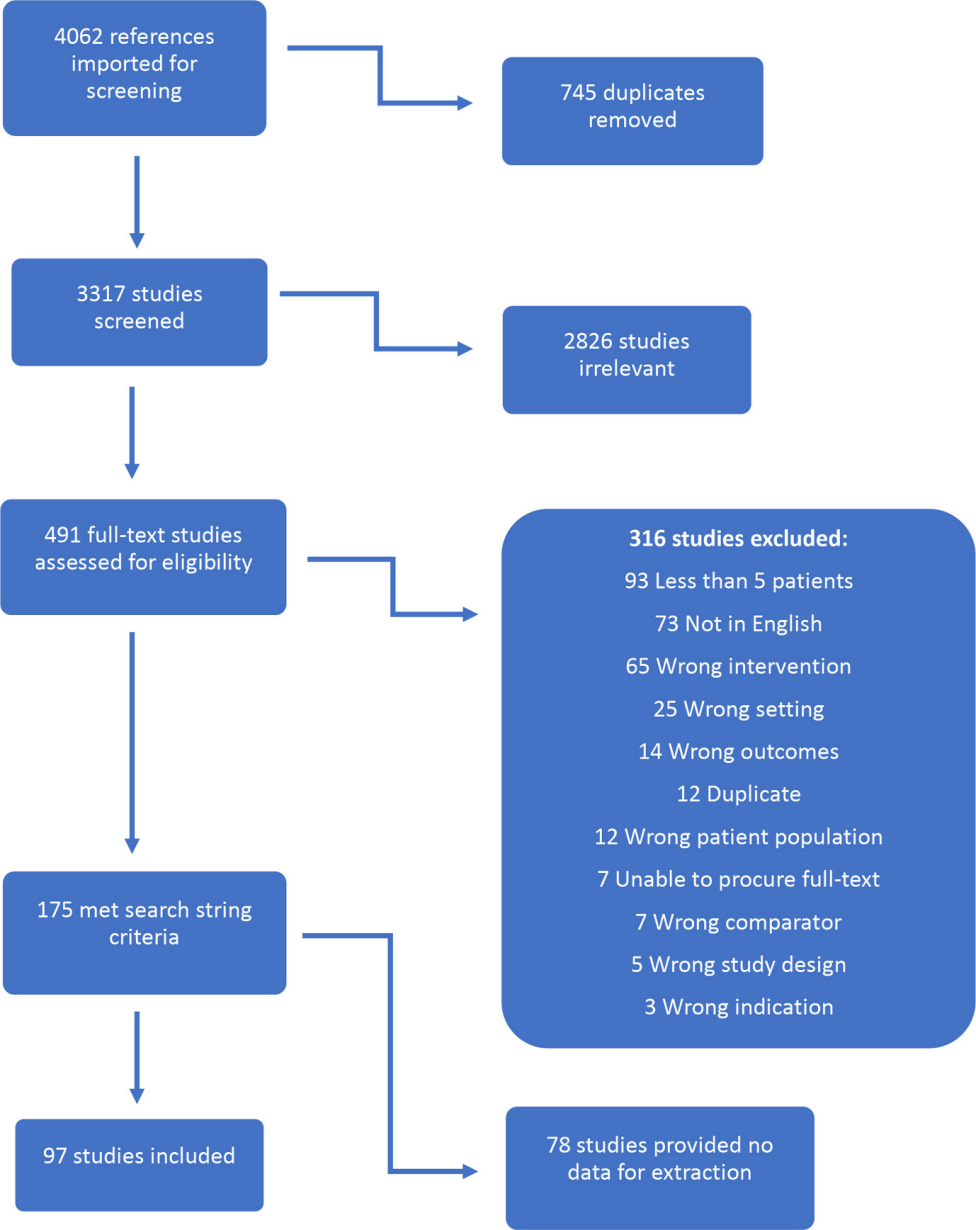
Flow chart of the screening process.

**Figure 2:**
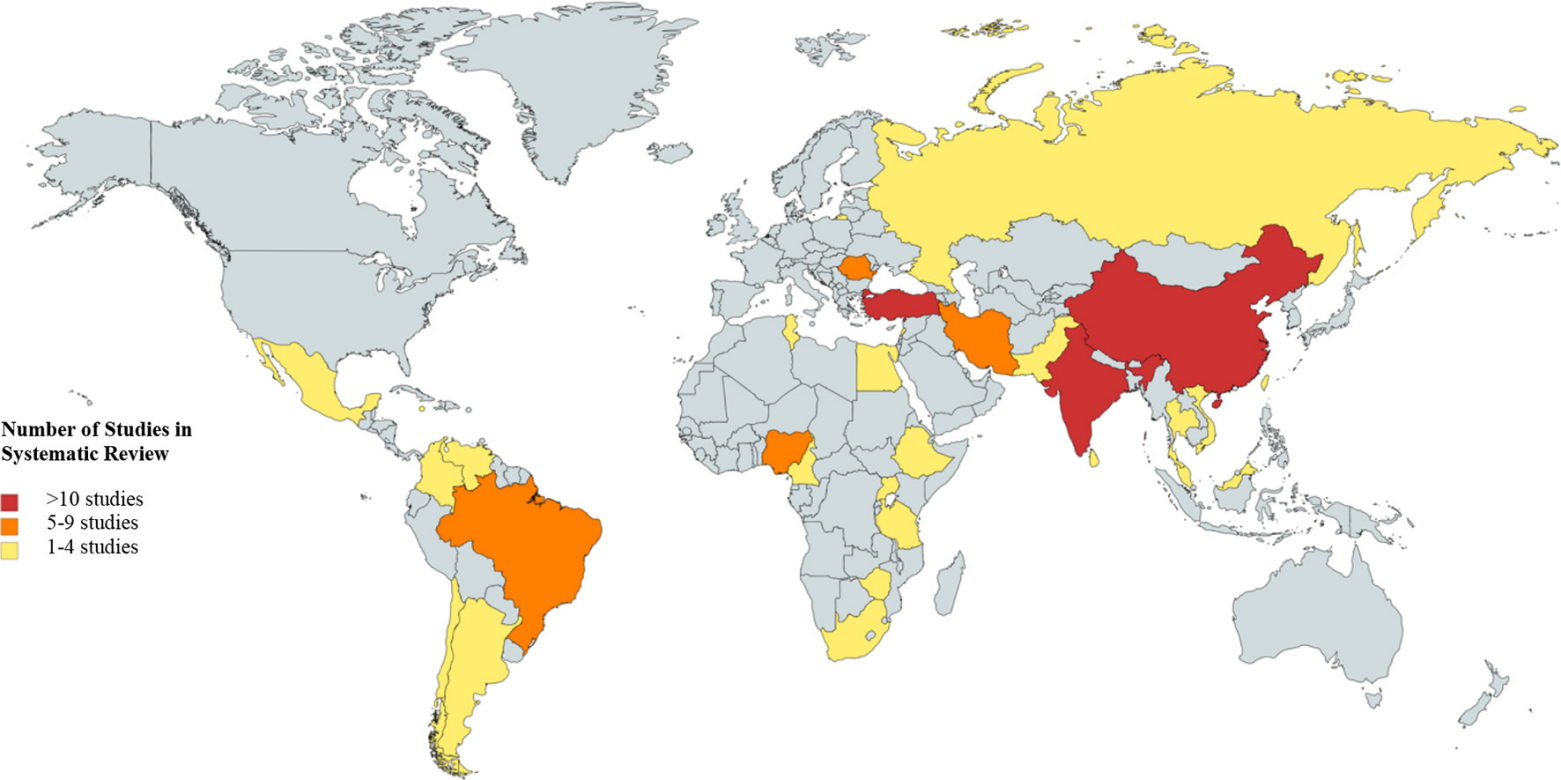
Regional depiction of articles included in the systematic review.

One hundred and seventy-five articles were found to meet all inclusion criteria listed in the search strings. Of these, 78 provided no data relevant to the study and thus were excluded. Ninety-seven studies were included in the data extraction phase (figure 1). Although all LMICs as defined by the World Bank were included in the search, not all countries yielded results in the text screening. One hundred and thirty-eight LMICs were included in the literature search; however, only 29 countries (21%) had any data that met the inclusion criteria (figure 2, online supplementary file 6).

10.1136/bmjpo-2020-000684.supp6 Supplementary data

It is also notable that the majority of included studies were conducted on an institutional level. Thus, while the data from these studies provide important information from the countries of this review, they are by no means a representative sample of an entire country or even an entire city. Each article also varied widely in the information it provided, ranging from antenatal detection rates to policy analysis. Given the heterogeneity of data extrapolated from these articles, it was not feasible to perform a meta-analysis.

### Percentage of women receiving antenatal ultrasound

Twenty-one studies (12 retrospective and 9 prospective observational studies) in 12 countries provided data on this (table 1). There was significant variation in the percentage of women receiving antenatal ultrasound scans, ranging from 6.8% in a Tanzanian study to 98.8% in a study from China. The data suggest a particularly low rate of women receiving antenatal ultrasound in Africa, with a median of 50.0% compared with 90.7% in Asia. No studies were conducted in Europe or South America, and only one study was conducted in North America (Jamaica, 98.2%).

**Table 1:** Percentage of women receiving antenatal ultrasound

Author(s)	Study location	Study type	Study population	# of women in study	# of women who received antenatal ultrasound (%)
**Africa**					
de Paul Djientcheu *et al*^22^	Cameroon	Retrospective descriptive observational study	Institutional; patients with NTDs admitted to neonatology unit	69	27 (39.1%)
Abdur-Rahman *et al*^23^	Nigeria	Retrospective descriptive observational study	Institutional; patients with abdominal wall defects at a tertiary health centre in the North-Central geopolitical zone of Nigeria	56	51 (91.1%)
Adeleye *et al* 24	Nigeria	Prospective cross-sectional study	Institutional; patients presenting with major CNS anomalies at tertiary hospital	54	43 (79.6%)
Adeleye and Joel-Medewase^25^	Nigeria	Retrospective cross-sectional survey	Institutional; patients with CNS defects at a neurosurgeon’s practice	151	91 (60.3%)
Bankole *et al*^26^	Nigeria	Prospective descriptive observational study	Institutional; patients presenting with CNS anomalies at tertiary hospital	108	54 (50%)
Idowu and Olawehinmi^27^	Nigeria	Prospective descriptive observational study	Institutional; patients presenting with NTDs at tertiary hospital	94	91 (96.8%)
Okafor *et al*^28^	Nigeria	Prospective cohort study	Institutional; patients with PUV at tertiary hospital	31	22 (71%)
Sekabira and Hadley^29^	South Africa	Retrospective descriptive observational study	Institutional; patients with gastroschisis at tertiary hospital	106	25 (23.6%)
Santos *et al*^30^	Tanzania	Prospective descriptive observational study	Institutional; patients with hydrocephalus at tertiary medical facility	125	9 (6.8%)
Wesonga *et al*^31^	Uganda	Prospective cohort study	Institutional; patients with gastroschisis at a tertiary hospital	41	10 (24.4%)
Munjanja *et al*^32^	Zimbabwe	Prospective descriptive observational study	Institutional; all patients delivered at Greater Harare Obstetric Unit	36 514	4429 (12.1%)
Total	11 studies, 6 countries	4 retrospective, 7 prospective observational studies	11 institutional	37 349	4852 Median: 50% Range: 6.8%–96.8%
**Asia**					
Lu *et al*^33^	China	Retrospective cross-sectional study	National; fetuses with NTDs	424	419 (98.8%)
Bhat *et al*^34^	India	Retrospective descriptive observational study	Institutional; patients admitted to NICU with CDH*	16	11 (68.8%)
Raman *et al*^35^	India	Retrospective descriptive observational study	Institutional; symptomatic patients with congenital cystic lung lesions at tertiary care centre	40	6 (15%)
Saha *et al*^36^	India	Retrospective descriptive observational study	Institutional; all deliveries at rural medical college	7365	6682 (90.7%)
Sood *et al*^37^	India	Retrospective descriptive observational study	Institutional; patients with NTDs at tertiary hospital	65	44 (67.7%)
Kazmi *et al*^38^	Iran	Prospective descriptive observational study	Institutional; patients referred to tertiary centre for myelomeningocele evaluation and management	140	136 (97.1%)
Samadirad *et al*^39^	Iran	Retrospective descriptive observational study	Regional; fetuses with congenital anomalies	639	557 (87.2%)
Ho *et al*^40^	Malaysia	Retrospective cohort study	Regional; births in Kinta District (253 cases with congenital anomalies and 506 control cases)	759	705 (92.9%)
Kitisomprayoonkul and Tongsong^41^	Thailand	Prospective descriptive observational study	Institutional; patients with NTDs at tertiary hospital	46	42 (91.3%)
Total	9 studies, 5 countries	7 retrospective, 2 prospective observational studies	6 institutional, 2 regional, 1 national	9494	8602 Median: 90.7% Range: 15%–98.8%
**North America**					
Johnson *et al*^42^	Jamaica	Retrospective observational review	Institutional; patients with congenital anomalies at tertiary hospital	55	54 (98.2%)
Total	1 study, 1 country	1 retrospective observational study	1 institutional	55	54 Median: N/A Range: N/A

CDH, congenital diaphragmatic hernia; CNS, central nervous system; NICU, neonatal intensive care unit; NTD, neural tube defects; PUV, posterior urethral valves.

### Effectiveness of antenatal ultrasound

Sixty-five studies (46 retrospective and 18 prospective observational studies and a parent survey) in 22 countries provided data on detection rates (table 2). Detection rates varied widely across studies, from 0% to 100%, with little correlation according to geographical region or type of anomaly. In Africa, the median detection rate was 16.7%, which is low compared with other LMICs, with 34.3% in South America, 34.7% in Asia and 47.3% in Europe. There was only one study from North America (Jamaica, 77.2%). Of the studies conducted from Africa, 8 of the 15 were in Nigeria and hence may not be representative of the whole region.

**Table 2:** Effectiveness of antenatal ultrasound

Author(s)	Study location	Study type	Study population	# of women in study	# of women who received antenatal diagnosis (%)
**Africa**					
de Paul Djientcheu *et al*^22^	Cameroon	Retrospective descriptive observational study	Institutional; patients with NTDs admitted to neonatology unit	27	8 (29.6%)
Sorri and Mesfin^43^	Ethiopia	Retrospective cross-sectional study	Multicentre; patients with NTDs at two tertiary hospitals	177	127 (71.8%)
Abdur-Rahman *et al*^23^	Nigeria	Retrospective descriptive observational study	Institutional; patients with abdominal wall defects at a tertiary health centre in the North-Central geopolitical zone of Nigeria	56	1 (1.8%)
Adeleye *et al*^24^	Nigeria	Prospective cross-sectional study	Institutional; patients presenting with major CNS anomalies at tertiary hospital	43	6 (14%)
Adeleye and Joel-Medwase^25^	Nigeria	Retrospective cross-sectional survey	Institutional; patients presenting with CNS anomalies at a neurosurgeon’s practice	146	26 (17.8%)
Akinmoladun *et al*^44^	Nigeria	Prospective descriptive observational study	Institutional; patients attending clinic for ultrasound screening	16	15 (93.8%)
Amadi and Eghwrudjakpor^45^	Nigeria	Retrospective descriptive observational study	Institutional; all patients with encephalocele at tertiary hospital	17	5 (29.4%)
Bankole *et al*^26^	Nigeria	Prospective descriptive observational study	Institutional; patients presenting with CNS anomalies at tertiary hospital	108	0 (0%)
Idowu and Olawehinmi^27^	Nigeria	Prospective descriptive observational study	Institutional; patients presenting with NTDs at tertiary hospital	91	23 (25.3%)
Okafor *et al*^28^	Nigeria	Prospective cohort study	Institutional; patients with PUV at tertiary hospital	31	2 (6.5%)
Choopa *et al*^46^	South Africa	Retrospective descriptive observational study	Institutional; patients with PUV at paediatric nephrology unit	60	10 (16.7%)
Sekabira and Hadley^29^	South Africa	Retrospective descriptive observational study	Institutional; patients with gastroschisis at tertiary hospital	106	13 (12.3%)
Chanoufi *et al*^47^	Tunisia	Retrospective case series (6 cases)	Institutional; cases of acardiac twins at maternity centre	6	1 (16.7%)
Wesonga *et al*^31^	Uganda	Prospective cohort study	Institutional; patients with gastroschisis at a tertiary hospital	41	1 (2.4%)
Munjanja *et al*^32^	Zimbabwe	Prospective descriptive observational study	Institutional; patients with congenital anomalies at obstetrical unit	91	46 (50.5%)
Total	15 studies, 7 countries	8 retrospective, 7 prospective observational studies	14 institutional, 1 multicentre	1016	284 Median: 16.7% Range: 0%–93.8%
**Asia**					
Deng *et al*^48^	China	Retrospective cross-sectional study	National; patients with omphalocele as reported in Chinese national birth defects monitoring network 1996–2006	827	322 (38.9%)
Hong *et al*^49^	China	Retrospective cohort study	Multicentre; patients with gastroschisis	17	3 (17.6%)
Liao *et al*^50^	China	Retrospective descriptive observational study	Institutional; patients with limb abnormalities at maternity and child health hospital	36	28 (77.8%)
Liu *et al*^51^	China	Retrospective cross-sectional study	Institutional; patients with congenital anomalies at a tertiary hospital	233	71 (30.5%)
Lu *et al*^33^	China	Retrospective cross-sectional study	National; patients with NTDs	424	361 (85.1%)
Shi *et al*^52^	China	Retrospective descriptive observational study	Institutional; cases of conjoined twins at tertiary hospital	7	4 (57.1%)
Weng *et al*^53^	China	Retrospective descriptive observational study	Institutional; patients with congenital choledochal cyst at specialty women’s hospital	21	19 (90.5%)
Bhat *et al*^34^	India	Retrospective descriptive observational study	Institutional; patients admitted to NICU with CDH	16	4 (25%)
Kumar *et al*^54^	India	Retrospective descriptive observational study	Institutional; symptomatic patients with congenital bronchopulmonary anomalies	25	2 (8%)
Raman *et al*^35^	India	Retrospective descriptive observational study	Institutional; symptomatic patients with congenital cystic lung lesions at tertiary care centre	40	3 (7.5%)
Rattan *et al*^55^	India	Retrospective descriptive observational study	Institutional; patients operated on for oesophageal atresia and tracheoesophageal fistula at a tertiary care centre	693	63 (9.1%)
Sanghvi *et al*^56^	India	Prospective descriptive observational study	Institutional; patients with renal anomalies at tertiary centre	125	65 (52%)
Sarin *et al*^57^	India	Retrospective case series (18 cases)	Institutional; patients with duodenal webs at tertiary hospital in India	18	2 (11.1%)
Sharada *et al*^58^	India	Retrospective descriptive observational study	Institutional; patients diagnosed with unilateral multicystic dysplastic kidney at tertiary hospital	47	34 (72.3%)
Singh *et al*^59^	India	Retrospective descriptive observational study	Institutional; patients with unilateral multicystic dysplastic kidney at tertiary centre	22	12 (54.5%)
Solanki *et al*^60^	India	Retrospective case series (6 cases)	Institutional; patients diagnosed with crossed fused renal ectopia at tertiary hospital	6	1 (16.7%)
Kazmi *et al*^38^	Iran	Prospective descriptive observational study	Institutional; patients referred to tertiary centre for myelomeningocele evaluation and management	136	33 (24.3%)
Mirshemirani *et al*^61^	Iran	Retrospective descriptive observational study	Institutional; patients treated for PUV at a tertiary hospital	98	20 (20.4%)
Shahkar *et al*^62^	Iran	Retrospective descriptive observational study	Institutional; patients with congenital pulmonary mass at a tertiary hospital	47	10 (21.3%)
Ho *et al*^40^	Malaysia	Retrospective cohort study	Regional; births in Kinta District (253 cases with congenital anomalies and 506 control cases)	252	37 (14.7%)
Munim *et al*^63^	Pakistan	Retrospective cohort study	Institutional; patients with diaphragmatic hernia at tertiary hospital	65	41 (63.1%)
Kitisomprayoonkul and Tongsong^41^	Thailand	Prospective descriptive observational study	Institutional; patients with NTDs at tertiary hospital	42	42 (100%)
Pitukkijronnakorn *et al*^64^	Thailand	Prospective cross- sectional study	Institutional; patients diagnosed with major congenital anomalies at tertiary hospital	316	144 (45.6%)
Srisupundit *et al*^65^	Thailand	Prospective descriptive observational study	Institutional; patients undergoing antenatal ultrasound at a university teaching hospital in Chiang Mai	34	24 (70.6%)
Total	24 studies, 6 countries	19 retrospective, 5 prospective observational studies	20 institutional, 1 multicentre, 1 regional, 2 national	3547	1345 Median: 34.7% Range: 7.5%–100%
**Europe**					
Iliescu *et al*^66^	Romania	Prospective descriptive observational study	Multicentre; patients at two institutions with major congenital anomalies	76	74 (97.4%)
Ognean *et al*^67^	Romania	Retrospective case series (7 cases)	Institutional; patients with oesophageal atresia at a tertiary centre	7	0 (0%)
Tarca and Aprodu^68^	Romania	Retrospective descriptive observational study	Institutional; patients with omphalocele at tertiary hospital	105	14 (13.3%)
Tarca and Aprodu^69^	Romania	Retrospective descriptive observational study	Institutional; patients with gastroschisis at tertiary hospital	54	9 (16.7%)
Tarca *et al*^70^	Romania	Retrospective descriptive observational study	Institutional; patients with gastroschisis at tertiary hospital	114	13 (11.4%)
Tudorache *et al*^71^	Romania	Retrospective descriptive observational study	Institutional; patients with cases of left-sided CDH at tertiary hospital	21	11 (52.4%)
Postoev *et al* 72	Russia	Retrospective cross-sectional study	Regional; patients with congenital anomalies in the Kola Peninsula (data from two birth defect registries)	232	81 (34.9%)
Aygun *et al*^73^	Turkey	Retrospective descriptive observational study	Institutional; patients with NTDs at tertiary hospital	100	72 (72%)
Dane *et al*^74^	Turkey	Prospective descriptive observational study	Institutional; fetuses with incurable congenital anomalies and curable severe congenital anomalies at a training and research hospital	24	23 (95.8%)
Orgul *et al*^75^	Turkey	Retrospective descriptive observational study	Institutional; patients with gastrointestinal tract malformations at a university children’s hospital	56	34 (60.7%)
Oztekin *et al*^76^	Turkey	Prospective descriptive observational study	Institutional; patients with a major structural congenital anomaly at an obstetrics and gynaecology teaching hospital	21	19 (90.5%)
Sahinoglu *et al*^77^	Turkey	Retrospective case series (6 cases)	Institutional; patients with limb body wall complex at a women and children’s research hospital	6	5 (83.3%)
Tabel *et al*^78^	Turkey	Prospective descriptive observational study	Institutional; patients with kidney or urinary tract anomalies at a university hospital	76	32 (42.1%)
Taskapilioglu *et al*^79^	Turkey	Retrospective descriptive observational study	Institutional; patients with open spina bifida at tertiary centre	78	26 (33.3%)
Total	14 studies, 3 countries	10 retrospective, 4 prospective observational studies	12 institutional, 1 multicentre, 1 regional	970	413 Median: 47.3% Range: 0%–97.4%
**North America**					
Johnson *et al*^42^	Jamaica	Retrospective descriptive observational study	Institutional; patients with congenital anomalies at tertiary hospital	57	44 (77.2%)
Total	1 study, 1 country	1 retrospective observational study	1 institutional	57	44 Median: N/A Range: N/A
**South America**					
Campana *et al*^80^	Argentina, Brazil, Chile, and Venezuela	Prospective descriptive observational study	Multicountry; patients with congenital anomalies in 18 Latin American hospitals	812	457 (56.3%)
Germani *et al*^81^	Argentina	Retrospective descriptive observational study	Institutional; patients with choledochal cyst at a private hospital	12	4 (33.3%)
Wyszynski *et al*^82^	Argentina	Survey	Institutional; patients with non-syndromic oral cleft (collected from parents’ survey data)	165	7 (4.2%)
Carvalho *et al*^83^	Brazil	Prospective cohort study	Institutional; patients with major congenital anomalies at a tertiary hospital	130	93 (71.5%)
Luiza *et al*^84^	Brazil	Retrospective cross-sectional study	Institutional; patients with orofacial cleft at a specialised society attending to cleft patients	168	7 (4.2%)
Tannuri *et al*^85^	Brazil	Retrospective descriptive observational study	Multicentre; patients with gastroschisis at three tertiary centres	163	134 (82.2%)
Vilela *et al*^86^	Brazil	Retrospective cross-sectional study	Institutional; patients with gastroschisis at a tertiary hospital	31	10 (32.3%)
Correa *et al*^87^	Colombia	Retrospective case-control study	City-wide; data from Bogota Congenital Malformations Surveillance Program	167	82 (49.1%)
de Rovetto *et al*^88^	Colombia	Retrospective descriptive observational study	City-wide; patients with congenital renal agenesis at centres in Cali, Colombia	38	8 (21.1%)
Rosselli *et al*^89^	Colombia	Retrospective descriptive observational study	City-wide; patients with congenital talipes equinovarus in Bogota, Colombia	178	61 (34.3%)
Saldarriaga *et al*^90^	Colombia	Retrospective cross-sectional study	City-wide; patients with congenital anomalies diagnosable by antenatal ultrasound in NICUs of Cali, Colombia	217	117 (53.9%)
Total	11 studies, 5 countries	8 retrospective, 2 prospective observational studies, 1 survey	5 institutional, 1 multicentre, 4 city-wide, 1 multicountry	2078	980 Median: 34.3% Range: 4.2%–82.2%

CDH, congenital diaphragmatic hernia; CNS, central nervous system; NICU, neonatal intensive care unit; NTD, neural tube defects; PUV, posterior urethral valves.

### Training of personnel performing ultrasound examination

Fifteen of the studies detailed the training of the personnel providing the ultrasound scans (table 3). Several of the included studies mentioned that the scans were performed by ‘experienced sonographers,’ but provided little detail as to the actual level of training of these providers. This makes it difficult to accurately assess the role that training may have in the detection of structural congenital anomalies.

**Table 3:** Training of personnel performing ultrasound examination

Author(s)	Study location	# of anomalies detected (%)	Information about training of personnel performing antenatal ultrasound examinations
**Africa**			
Adeleye *et al*^24^	Nigeria	6/43 (14)	Radiologists performed 5% of cases; medical doctors performed 11%; unknown training/status performed 84% of cases
Adeleye and Joel-Medewase^25^	Nigeria	26/146 (17.8)	22% of ultrasounds performed by a radiologist; sonographers in rest of the cases were personnel with unknown training; authors noted that prenatal diagnosis was significantly more likely in cases where sonographer was certified radiologist
Akinmoladun *et al*^44^	Nigeria	15/16 (93.8)	A consultant radiologist trained in fetal anomaly scanning performed all the scans (the authors note that this radiologist received extensive training at a renowned centre in the UK)
Idowu and Olawehinmi^27^	Nigeria	23/91 (25.3)	Authors noted that low diagnosis ‘may be due to the high prevalence of the test being done by non-specialist (untrained radiologist) in our environment’
Wesonga *et al*^31^	Uganda	1/41 (2.4)	Performed by ultrasound technicians holding a diploma; no further information about diploma
**Asia**			
Liao *et al*^50^	China	28/36 (77.8)	Ten certified physicians participated in the study protocol, each of whom has more than 5 years of experience in fetal sonography
Xie *et al*^91^	China	Not specified	2 sonographers—1 with 10 years of experience in obstetric sonography and the other with 22 years of experience
Sanghvi *et al*^56^	India	65/125 (52)	Performed by ‘experienced sonologists’
Ghavami and Abedinzadeh^92^	Iran	Not specified	Performed by ‘two expert operators’
Pitukkijronnakorn *et al*^64^	Thailand	144/316 (45.6)	All scans were performed by an obstetrician who was trained as a level one ultrasonography; in cases of uncertain abnormal findings, the women were reviewed by a level two obstetrician with repeated scans
**Europe**			
Iliescu *et al*^66^	Romania	74/76 (97.4)	Scans performed by obstetricians specialising in prenatal diagnosis (including the anomaly scan and echocardiography) who had held accreditation for the 11–14 weeks assessment for at least 5 years prior to the start of the study period
Dane *et al*^74^	Turkey	23/24 (95.8)	2 operators with approximately 6 years and 2 years of experience in gestational ultrasound scanning
Kutuk *et al*^93^	Turkey	Not specified	All ultrasound scans performed by ‘two experienced maternal-fetal specialists’
Oztekin *et al*^76^	Turkey	19/21 (90.5)	All scans performed by the same experienced radiologist
**North America**			
Johnson *et al*^42^	Jamaica	44/57 (77.2)	8 OB/GYN residents in training for at least 2 years

### Morbidity and mortality outcomes

Only four studies produced any data comparing the morbidity and mortality outcomes between neonates with an antenatal diagnosis versus neonates with a postnatal diagnosis (table 4). In the study that addressed gastroschisis, outcomes were more favourable for neonates who had received an antenatal diagnosis compared with those who had not (20% vs 66.7% mortality). This was not the case for the study which addressed congenital diaphragmatic hernia (CDH); however, this may reflect that more severe forms of anomalies are easier to detect antenatally.

**Table 4:** Morbidity and mortality outcomes

Author(s)	Study location	Patient population	Mortality with antenatal diagnosis	Mortality without antenatal diagnosis	Morbidity with antenatal diagnosis	Morbidity without antenatal diagnosis
**Asia**						
Bhat *et al*^34^	India	Institutional; patients with CDH	4/4 (100%)	3/12 (25%)	N/A	4/9 (44.4%)
**Europe**						
Savran *et al*^94^	Turkey	Institutional; patients with duodenal atresia	0/9 (0%)	0/6 (0%)	0/9 (0%)	1/6 (16.7%)
**North America**						
Johnson *et al*^42^	Jamaica	Institutional; patients with congenital anomalies	19/44 (43.2%)	5/13 (38.5%)	11/29 (37.9%)	9/12 (75%)
**South America**						
Vilela *et al*^86^	Brazil	Institutional; patients with gastroschisis	2/10 (20%)	14/21 (66.7%)	Not specified	Not specified

CDH, congenital diaphragmatic hernia.

### Termination rates

Twenty-five studies (21 retrospective and 3 prospective observational studies and an ethnographic study) in 15 countries provided data on termination rates (table 5). Termination rates were highly varied, with a median of 17.1% in Africa, 34.4% in Asia, 50.2% in Europe and 62.4% in South America (range 0%–98.3%). Only one study from Africa evaluated termination rates for lethal anomalies and had just five participants. Thus, it is difficult to compare the termination rate of lethal anomalies with other regions, which contain such data. Termination rates can also be affected by the type of anomaly, the severity, the gestational age at diagnosis, the national termination policies and the cultural appropriateness of termination. Hence, while these termination rates offer valuable insight, it is necessary to also consider the underlying determinants that have impacted termination decisions.

**Table 5:** Termination rates

Author(s)	Study location	Study type	Study population	# of fetuses	# of fetuses terminated (%)
**Africa**					
de Paul Djientcheu *et al*^22^	Cameroon	Retrospective descriptive observational study	Institutional; patients with NTDs	8	0 (0%)
Shalaby *et al*^95^	Egypt	Retrospective cross-sectional study	Institutional; patients with urinary anomalies	41	11 (26.8%)
Sorri and Mesfin^43^	Ethiopia	Retrospective cross-sectional study	Multi-centre; patients with NTDs at two tertiary hospitals	177	13 (7.3%)
Akinmoladun *et al*^44^	Nigeria	Prospective descriptive observational study	Institutional; patients with lethal congenital anomalies	5	4 (80%)
Total	4 studies, 4 countries	3 retrospective, 1 prospective observational studies	3 institutional, 1 multicentre	231	28 Median: 17.1% Range: 0%–80%
**Asia**					
Li *et al*^96^	China	Retrospective descriptive observational survey	Regional; patients with NTDs	160	72 (45%)
Lu *et al*^33^	China	Retrospective cross-sectional study	National (data from 20 counties); patients with NTDs	361	355 (98.3%)
Xie *et al*^91^	China	Retrospective descriptive observational study	Institutional; patients with bronchopulmonary sequestration	22	8 (36.4%)
Zhang *et al*^97^	China	Retrospective descriptive observational study	Institutional; patients with pulmonary sequestration	68	2 (2.9%)
Kashyap *et al*^98^	India	Retrospective descriptive observational study	Institutional; patients with lethal congenital anomalies detected prior to 20 weeks of gestation	103	80 (77.7%)
Kumar *et al*^99^	India	Prospective cohort study	Institutional; patients with severe renal anomalies	55	9 (16.4%)
Kumar *et al*^100^	India	Prospective descriptive observational study	Institutional; patients with renal anomalies	136	12 (8.8%)
Sanghvi *et al*^56^	India	Prospective descriptive observational study	Institutional; patients with lethal renal anomalies	7	2 (28.6%)
Samadirad *et al*^39^	Iran	Retrospective descriptive observational study	Regional; patients with congenital anomalies	603	201 (33.3%)
Munim *et al*^63^	Pakistan	Retrospective cohort study	Institutional; patients with diaphragmatic hernia	41	6 (14.6%)
Hsieh *et al*^101^	Taiwan	Retrospective descriptive observational study	Institutional; patients with CDH	31	11 (35.5%)
Jaruratanasirikul *et al*^102^	Thailand	Retrospective cross-sectional study	Regional; patients with NTDs	28	12 (42.9%)
Pitukkijronnakorn *et al*^64^	Thailand	Prospective cross-sectional study	Institutional; patients with congenital anomalies	316	87 (27.5%)
Gammeltoft *et al*^103^	Vietnam	Ethnographic study	Institutional; patients with congenital anomalies	30	17 (56.7%)
Total	14 studies, 7 countries	9 retrospective, 4 prospective observational studies; 1 ethnographic study	10 institutional, 3 regional, 1 national	1961	874 Median: 34.4% Range: 2.9%–98.3%
**Europe**					
Tudorache *et al*^71^	Romania	Retrospective descriptive observational study	Institutional; patients with severe CDH diagnosed in the second trimester of pregnancy	6	4 (66.7%)
Aygun *et al*^73^	Turkey	Retrospective descriptive observational study	Institutional; patients with NTDs	72	0 (0%)
Oztarhan *et al*^104^	Turkey	Retrospective cohort study	Institutional; patients with lethal congenital anomalies	1906	640 (33.6%)
Sahinoglu *et al*^77^	Turkey	Retrospective case series (6 cases)	Institutional; patients with body wall complex	6	4 (66.7%)
Total	4 studies, 2 countries	4 retrospective observational studies	4 institutional	1990	648 Median: 50.2% Range: 0%–66.7%
**North America**					
Johnson *et al*^42^	Jamaica	Retrospective descriptive observational study	Institutional; patients with congenital anomalies	44	10 (22.7%)
Total	1 study, 1 country	1 retrospective observational study	1 institutional	44	10 Median: N/A Range: N/A
**South America**					
Brizot *et al*^105^	Brazil	Retrospective descriptive observational study	Institutional; pairs of conjoined twins in which surgical separation was impossible and the condition lethal	36	30 (83.3%)
Pelizzari *et al*^106^	Brazil	Retrospective cohort study	Institutional; patients with anencephaly	29	12 (41.4%)
Total	2 studies, 1 country	2 retrospective observational studies	2 institutional	65	42 (64.6%) Median: 62.4% Range: 41.4%–83.3%

CDH, congenital diaphragmatic hernia; NTD, neural tube defects.

### Referral rates for further antenatal care and management planning

No studies addressed this issue.

### Policy data

Thirteen articles provided policy data from 10 countries (table 6). Only two studies, in India and Brazil, mentioned national policies for antenatal ultrasound simply stating that they did not exist. Termination of pregnancy remains a highly sensitive topic in many communities, which is reflected in the variation of policies across the globe.

**Table 6:** Policy data

Author(s)	Study location	Policy data about antenatal screening and/or termination legislation
**Africa**		
Oloyede and Oyedele^107^	Nigeria	In Nigeria, the two existing pregnancy termination laws are restrictive in nature. However, termination is often done when a fetus is malformed on the grounds of preserving the mental health of the women.
**Asia**		
Acharya *et al*^108^	India	India has no definite policy for the ultrasound screening for fetal abnormalities and antenatal diagnostic techniques. The law in India says that those who meet the criteria of the PCPNDT Act can perform an ultrasound scan and they must be sufficiently trained and registered with the proper authority.
Neogi^109^	India	Abortion was legalised in India in 1971 under the Medical Termination of Pregnancy Act. It permits abortion by 1 doctor before 12 weeks of gestation but if the duration of pregnancy is more than 12 weeks but less than 20 weeks, then the opinion of 2 medical practitioners is necessary to terminate the pregnancy.
Phadke *et al*^110^	India	In India, the Medical Termination of Pregnancy Act of 1971 (The MTP Act, No. 34 of 1971) does not allow pregnancy termination on grounds of fetal abnormality after 20 weeks of gestation.
Ranji and Dykes^111^	Iran	According to the regulations of the Iranian Ministry of Health, ultrasound examinations during pregnancy must be carried out by radiologists.
Arawi and Nassar^112^	Lebanon	Lebanese law stipulates that pregnancy termination is forbidden except when the pregnancy endangers the health of the mother and only after consulting with two physicians.
Senanayake and de Silva^113^	Sri Lanka	In Sri Lanka, it is illegal to terminate a pregnancy even in cases of early diagnosis (11–14 weeks of gestation).
**Europe**		
Hostiuc *et al*^114^	Romania	According to Romanian law, abortion over 14 weeks is only allowed in cases of severe congenital defects and pregnancies that threaten the life of the mother.
Oztarhan *et al*^104^	Turkey	Turkish law authorises pregnancy termination voluntarily until 10 weeks in unwanted pregnancies and at any gestational age for medical indications that are considered potentially life threatening to the mother or fetus. The legal process requires one obstetrician and one physician to agree that pregnancy termination is valid for a medical reason.
**North America**		
Lisker *et al*^115^	Mexico	Pregnancy termination is illegal in most Mexican States, except in the case of rape or if the mother’s life is at risk by the continuation of pregnancy. In Mexico City and 12 of the 31 states, the presence of a severe congenital anomalies has become a justification for the legal termination of pregnancy.
**South America**		
Groisman *et al*^116^	Argentina	According to the Argentinian criminal code, termination of pregnancy is illegal unless the pregnancy is threat to woman's life or pregnancy is consequence of rape of a mentally retarded woman. In the city of Buenos Aires, it is legal to induce labour after 24 weeks of gestational age in case of anencephaly and other lethal conditions.
Benute *et al*^117^	Brazil	Brazilian law does not include lethal fetal malformation as an indication for pregnancy termination; however, many couples ask a court for permission to terminate a pregnancy on the grounds that it is the option which creates less suffering.
Mirlesse and Ville^118^	Brazil	Ultrasound is not explicitly recommended by Brazilian authorities. Brazilian legislation considers termination of pregnancy to be a crime (except in cases of rape or pregnancies which risk the mother’s life). However, for lethal fetal malformations, it is possible to apply to the courts for an exceptional authorisation to abort. These requests require a medical referral centre to perform an ultrasound and prepare a very detailed file.

PCPNDT, Pre-Conception and Pre-Natal Diagnostic Techniques Act.

### Policy assessment

WHO guidelines recommend the need for one antenatal ultrasound scan prior to 24 weeks gestation.^13^ Studies suggest that the ideal detection window for structural congenital anomalies is 19–21 weeks of gestation.^14^ At this point, it is possible to detect most structural congenital anomalies and is within the legal termination timeframe for many countries. Of note, many of the congenital anomalies detected antenatally in this review were not diagnosed until after 24 weeks gestation. This may be explained by the timing of the first antenatal ultrasound and/or the level of ultrasonographer training. The WHO recommends that ultrasound trainees receive at least 3–6 months of training, culminating in 300–500 ultrasound examinations.^15^ A recent study found that the majority of ultrasound providers in LMICs do not have the minimum training as set by the WHO.^16^ Hence, many ultrasound practitioners in LMICs may not have the skills to accurately detect congenital anomalies.

## Discussion

The median proportion of women receiving an antenatal ultrasound varied from 50.0% in Africa to 90.7% in Asia. It is likely that these are an overestimate of the true population rates considering that the majority of studies were undertaken at tertiary facilities. To fully understand what percentage of women receive antenatal ultrasound, further studies must be conducted at a population level, regionally and nationally, rather than at an institutional level. Research must also address the availability and accessibility of antenatal ultrasound and the barriers to receiving a scan.

Detection rates varied widely, from 0% to 100%, with the lowest reported rates in Africa (16.7%). Low detection rates may be because ultrasound providers did not specifically screen for congenital anomalies. Currently, many women in LMICs receive antenatal ultrasound examinations for the assessment of pregnancy progress, such as to determine the gestational age, sex of the baby and to hear the heartbeat, rather than to detect anomalies. This is in contrast to HICs where the majority of women receive an anomaly scan around 20 weeks gestation.^14^ Another possible reason for low detection may be the training level of the ultrasound provider; there appears to be a trend between higher levels of training and higher detection rates. This warrants further investigation to determine minimum training requirements and associated policy and monitoring.

The First Look Study is an important randomised controlled trial which assessed the use of antenatal ultrasound in the Democratic Republic of the Congo, Guatemala, Kenya, Pakistan and Zambia.^17^ Although 95% of women in their intervention group received antenatal ultrasound scans (compared with 43% in the control group) and detection rates improved, hospital delivery did not increase for complicated pregnancies and thus there was no resultant improvement in neonatal mortality. In an additional survey by the same group, barriers to referral attendance included cost, distance and lack of transportation.^18^ For women who did attend referral, barriers included not being connected to the correct provider and being told to return at a later time.^18^ The authors conclude that without improvement of subsequent care, antenatal ultrasound offered limited impact.^17^ Hence, to reduce neonatal morbidity and mortality, detection of an anomaly must be followed by referral for antenatal counselling and delivery at a tertiary centre which can provide the necessary surgical care at birth where required. It is also necessary to offer termination for conditions which are incompatible with life, where culturally acceptable.

Hence, it is vital to further investigate barriers to accessing delivery at a paediatric surgery centre once a congenital anomaly has been diagnosed and ways to address these barriers. Future studies must also investigate the effects of both antenatal diagnosis and delivery at a tertiary paediatric surgery centre on morbidity and mortality outcomes in the LMIC setting; this systematic review highlighted a severe lack of such vital data. The recently completed Global PaedSurg study may provide such data for a selection of common gastrointestinal congenital anomalies globally, which collectively have a particularly high mortality in the LMIC setting.^19^ As anomaly screening rates increase in LMICs, it will be also be important to monitor termination rates along with reasons for termination, to ensure the benefits of antenatal diagnosis are optimised both clinically and ethically.

To address some of these issues, there is a need for global collaboration. This collaboration must include members from multidisciplinary backgrounds, including policymakers, obstetricians, neonatologists, paediatric surgeons, midwives and allied professionals. The Global Initiative for Children’s Surgery (GICS) is a multidisciplinary collaborative whose aim is to improve health outcomes for children requiring surgery in LMICs.^20^ This initiative connects the expertise of providers in LMICs and HICs and is committed to expanding the representation and leadership of stakeholders in LMICs. GICS has recently created a congenital anomalies working group, which is planning some of the following projects: (1) to produce guidelines on how to diagnose structural congenital anomalies via antenatal ultrasound; (2) to produce referral and management guidelines following an antenatal diagnosis; and (3) to produce information sheets that can be translated into various languages for parents that contain details about common congenital anomalies. Global collaboration must also extend to the level of the WHO and the Ministries of Health to ensure that recommendations are detailed in policy and implemented into practice.

If these steps are taken, improvements in neonatal health outcomes may be realised, as seen in HICs. Early detection and immediate surgical intervention of congenital anomalies, such as gastroschisis, has been effective in significantly reducing neonatal mortality in HICs.^5^ The mortality of gastroschisis has significantly improved in HICs over a period of 50 years, to less than 5% today.^5^ This can be attributed to improvements in accurately diagnosing gastroschisis antenatally, monitoring the fetus for complications, and planning for delivery at a facility with paediatric surgeons available.^21^ Similar trends have been seen for other congenital anomalies in HICs such as intestinal atresia, CDH, omphalocele, oesophageal atresia and posterior urethral values. By understanding the current role of antenatal ultrasound in LMICs and the barriers to detection, referral and management of structural congenital anomalies, appropriate interventions can be implemented to help improve outcomes.

Although this systematic review provides useful data, it is also important to note a few of the limitations of the study. First, only articles in English were included in this systematic review, which may exclude other relevant studies. This study used four electronic databases for the search. The expansion of search databases to include African Journals Online, Scielo and Regional WHO’s African Index Medicus may have provided other studies from LMICs that were not indexed in the search engines used. It is vital to include these databases in future research focusing on LMICs. Furthermore, it is important to note that antenatal ultrasound has further diagnostic capabilities, such as detecting abnormal growth or improper placental position and this review only focused on the detection of structural congenital anomalies. Further studies could also include other uses of antenatal ultrasound for improving neonatal and indeed maternal health outcomes. Finally, the policy data in this study represent what was accurate when the studies were published. Some of the policy data may now be out of date.

## Conclusion

The data from this review suggest that the percentage of women in LMICs who receive an antenatal ultrasound examination varies considerably and is particularly low in sub-Saharan African countries. Even when antenatal ultrasound scans are performed, accurate detection rates are often very low. The level of training (and the type of training) of the sonographer may be indicative of the accuracy of diagnosis. Only four studies delineated the morbidity and mortality outcomes among neonates with an antenatal diagnosis compared with postnatal diagnosis. Hence, although the benefits of antenatal ultrasound are widely documented in HICs, data are severely lacking in LMICs. It is clear that the use of antenatal ultrasound in LMICs is not maximised to its highest potential.

**What is known about the subject?**

- Congenital anomalies are the fifth leading cause of death in children under 5 years of age globally.
- Ninety-seven per cent of congenital anomaly deaths occur in low/middle-income countries (LMICs), many of which may be preventable with antenatal diagnosis and planned surgical intervention following birth.
- Antenatal ultrasound examinations in HICs are commonplace and highly accurate, but accessibility and effectiveness are limited in LMICs.

**What this study adds?**

- Rates of antenatal ultrasound examination vary significantly in LMICs, ranging from 6.8% to 98.8%.
- There is significant variation in the accuracy of antenatal diagnosis in LMICs, with detection rates varying from 0% to 100% (median of 16.7% in Africa).
- Available data suggest that the level of ultrasonographer training may affect the accuracy of diagnosis, but further research into this is required.

## Supplementary Material

Author's manuscript
