# Supplementary material for: Investigating the use of ultrasonography for the antenatal diagnosis of structural congenital anomalies in low-income and middle-income countries: a systematic review
Source: BMJ Paediatr Open. 2020 Aug 20;4(1):e000684. doi: 10.1136/bmjpo-2020-000684 (PMC7443309; doi:10.1136/bmjpo-2020-000684)
Supplement: Supplementary data [file bmjpo-2020-000684supp001.pdf]

Supplementary File 1

Investigating the Use of Ultrasonography for the Antenatal Diagnosis of Structural Congenital Anomalies in Low- and Middle-Income Countries: A Systematic Review

PRISMA (Preferred Reporting Items for Systematic Reviews and Meta-Analyses) 2009 Checklist

Section/topic	#	Checklist item	Section Heading
**TITLE**			
Title	1	Identify the report as a systematic review, meta-analysis, or both.	Title
**ABSTRACT**			
Structured summary	2	Provide a structured summary including, as applicable: background; objectives; data sources; study eligibility criteria, participants, and interventions; study appraisal and synthesis methods; results; limitations; conclusions and implications of key findings; systematic review registration number.	Abstract
**INTRODUCTION**			
Rationale	3	Describe the rationale for the review in the context of what is already known.	Introduction
Objectives	4	Provide an explicit statement of questions being addressed with reference to participants, interventions, comparisons, outcomes, and study design (PICOS).	Introduction
**METHODS**			
Protocol and registration	5	Indicate if a review protocol exists, if and where it can be accessed (e.g., Web address), and, if available, provide registration information including registration number.	Methodology
Eligibility criteria	6	Specify study characteristics (e.g., PICOS, length of follow-up) and report characteristics (e.g., years considered, language, publication status) used as criteria for eligibility, giving rationale.	Methodology
Information sources	7	Describe all information sources (e.g., databases with dates of coverage, contact with study authors to identify additional studies) in the search and date last searched.	Methodology
Search	8	Present full electronic search strategy for at least one database, including any limits used, such that it could be repeated.	Methodology
Study selection	9	State the process for selecting studies (i.e., screening, eligibility, included in systematic review, and, if applicable, included in the meta-analysis).	Methodology
Data collection process	10	Describe method of data extraction from reports (e.g., piloted forms, independently, in duplicate) and any processes for obtaining and confirming data from investigators.	Methodology
Data items	11	List and define all variables for which data were sought (e.g., PICOS, funding sources) and any assumptions and simplifications made.	Methodology

Risk of bias in individual studies	12	Describe methods used for assessing risk of bias of individual studies (including specification of whether this was done at the study or outcome level), and how this information is to be used in any data synthesis.	Methodology
Summary measures	13	State the principal summary measures (e.g., risk ratio, difference in means).	Methodology
Synthesis of results	14	Describe the methods of handling data and combining results of studies, if done, including measures of consistency (e.g., I^2) for each meta-analysis.	Methodology

From: Moher D, Liberati A, Tetzlaff J, Altman DG, The PRISMA Group (2009). Preferred Reporting Items for Systematic Reviews and Meta-Analyses: The PRISMA Statement. PLoS Med 6(6): e1000097. doi:10.1371/journal.pmed1000097
