# Supplementary material for: Investigating the use of ultrasonography for the antenatal diagnosis of structural congenital anomalies in low-income and middle-income countries: a systematic review
Source: BMJ Paediatr Open. 2020 Aug 20;4(1):e000684. doi: 10.1136/bmjpo-2020-000684 (PMC7443309; doi:10.1136/bmjpo-2020-000684)
Supplement: Supplementary data [file bmjpo-2020-000684supp002.pdf]

Supplementary File 2

Investigating the Use of Ultrasonography for the Antenatal Diagnosis of Structural Congenital Anomalies in Low- and Middle-Income Countries: A Systematic Review

Search Strings

Search String 1	Search String 2	Search String 3
Congenital Anomalies, Congenital Abnormalities, Congenital Malformation, Fetal Malformation, Birth Defects, Anencephaly, Conjoined Twins, Congenital Heart Defects, Anorectal Malformations, Anal Stenosis, Anal Atresia, Imperforate Anus, Biliary Atresia, Choledochal Cyst, Diaphragmatic Eventration, Esophageal Atresia, Tracheoesophageal Fistula, Intestinal Atresia, Duodenal Obstruction, Duodenal Atresia, Colonic Atresia, Malrotation, Apple Peel Syndrome, Congenital Diaphragmatic Hernias, Gastroschisis, Abdominal Wall Defects, Exomphalos, Omphalocele, Congenital Limb Deformities, Neural Tube Defects, Bronchogenic Cyst, Bronchopulmonary Sequestration, Congenital Cystic Adenomatoid Malformation of Lung, Renal Anomalies, Genito-urinary Anomalies, Maxillofacial Abnormalities, Mouth Abnormalities, Umbilical Hernia, Hirschsprung Disease, Ganglionic Megacolon, Rectosigmoid Aganglionosis, Colonic Aganglionosis, Intestinal Aganglionosis, Volvulus, Intestinal Volvulus	LMICs, Low- and Middle-Income Countries, Developing Countries, Low-Resource Settings, Underdeveloped Countries, Low-Income Countries, Middle-Income Countries, Limited Resource Settings, Africa South of the Sahara, Sub-Saharan Africa, Less Resourced Communities, Afghanistan, Albania, Algeria, American Samoa, Angola, Argentina, Armenia, Azerbaijan, Bangladesh, Belarus, Belize, Benin, Bhutan, Bolivia, Bosnia and Herzegovina, Botswana, Brazil, Bulgaria, Burkina Faso, Burundi, Cabo Verde, Cambodia, Cameroon, Central African Republic, Chad, China, Colombia, Comoros, Democratic Republic of the Congo, DRC, Republic of the Congo, Costa Rica, Cote d'Ivoire, Ivory Coast, Croatia, Cuba, Djibouti, Dominica, Dominican Republic, Ecuador, Egypt, El Salvador, Equatorial Guinea, Eritrea, Ethiopia, Fiji, Gabon, Gambia, Georgia, Ghana, Grenada, Guatemala, Guinea, Guinea-Bissau, Guyana, Haiti, Honduras, India, Indonesia, Islamic Republic of Iran, Iraq, Jamaica, Jordan, Kazakhstan, Kenya, Kiribati, Democratic People's Republic of Korea, Kosovo, Kyrgyz Republic, Lao PDR, Laos, Lebanon, Lesotho, Liberia, Libya, Macedonia Republic, Madagascar, Malawi, Malaysia, Maldives, Mali, Marshall Islands, Mauritania, Mauritius, Mexico, Micronesia, Moldova, Mongolia, Montenegro, Morocco, Mozambique, Myanmar, Namibia, Nauru, Nepal, Nicaragua, Niger, Nigeria, Pakistan, Panama, Papua New Guinea, Paraguay, Peru, Philippines, Romania, Russian Federation, Rwanda, Samoa, Sao Tome and Principe, Senegal, Serbia, Sierra Leone, Solomon Islands, Somalia, Somaliland, South Africa, South Sudan, Sri Lanka, Saint Lucia, Saint Vincent and the Grenadines, Sudan, Suriname, Swaziland, Syrian Arab Republic, Syria, Tajikistan, Tanzania, Thailand, Timor-Leste, East Timor, Togo, Tonga, Tunisia, Turkey, Turkmenistan, Tuvalu, Uganda, Ukraine, Uzbekistan, Vanuatu, Venezuela, Vietnam, West Bank and Gaza, Republic of Yemen, Zambia, Zimbabwe	Antenatal Diagnosis, Prenatal Diagnosis, Antenatal Screening, Prenatal Screening, Antenatal Ultrasound, Prenatal Ultrasound, Antenatal Ultrasonography, Prenatal Ultrasonography
