# Supplementary material for: Investigating the use of ultrasonography for the antenatal diagnosis of structural congenital anomalies in low-income and middle-income countries: a systematic review
Source: BMJ Paediatr Open. 2020 Aug 20;4(1):e000684. doi: 10.1136/bmjpo-2020-000684 (PMC7443309; doi:10.1136/bmjpo-2020-000684)
Supplement: Supplementary data [file bmjpo-2020-000684supp003.pdf]

Supplementary File 3

Investigating the Use of Ultrasonography for the Antenatal Diagnosis of Structural Congenital Anomalies in Low- and Middle-Income Countries: A Systematic Review

Search Strategy as Run on Ovid MEDLINE(R) Epub Ahead of Print, In-Process & Other Non-Indexed Citations, Ovid MEDLINE(R) Daily, Ovid MEDLINE and Versions(R) <1946 to June 27, 2018>

No.	Search Terms	Results
1	exp Congenital Abnormalities/ or congenital anomal*.mp.	559922
2	exp Congenital Abnormalities/ or Congenital Abnormal*.mp.	555311
3	Congenital Malformation*.mp.	13826
4	Fetal Malformation*.mp.	1917
5	Birth Defect*.mp.	9122
6	Anencephaly.mp. or exp ANENCEPHALY/	3875
7	Conjoined Twins.mp. or exp Twins, Conjoined/	2022
8	exp Heart Defects, Congenital/ or Congenital Heart Defect*.mp.	142854
9	Anorectal Malformation*.mp.	1896
10	Anal Stenosis.mp.	389
11	Anal Atresia.mp. or exp Anus, Imperforate/	2815
12	Imperforate Anus.mp. or exp Anus, Imperforate/	2992
13	Biliary atresia.mp. or exp Biliary Atresia/	4482
14	exp Choledochal Cyst/ or Choledochal Cyst*.mp.	2825
15	exp Diaphragmatic Eventration/ or Diaphragmatic Eventration*.mp.	943
16	Esophageal Atresia.mp. or exp Esophageal Atresia/	4173
17	exp Tracheoesophageal Fistula/ or Tracheo-?esophageal fistula*.mp.	3676
18	Intestinal Atresia.mp. or exp Intestinal Atresia/	2301
19	Duodenal atresia.mp.	839
20	Colonic Atresia.mp.	152
21	Malrotation.mp.	2393
22	Apple peel syndrome.mp.	16
23	exp Hernias, Diaphragmatic, Congenital/ or congenital diaphragmatic hernia*.mp.	5530
24	Gastroschisis.mp. or exp GASTROSCHISIS/	2313
25	Abdominal Wall Defect*.mp.	2009
26	Exomphalos.mp. or exp Hernia, Umbilical/	3740
27	Omphalocele.mp. or exp Hernia, Umbilical/	4242
28	Congenital Limb Deformities.mp. or exp Limb Deformities, Congenital/	22483
29	exp Neural Tube Defects/ or Neural Tube Defect*.mp.	29051
30	exp Bronchogenic Cyst/ or Bronchogenic Cyst*.mp.	1810
31	exp Bronchopulmonary Sequestration/ or Bronchopulmonary sequestration*.mp.	2022
32	exp "Cystic Adenomatoid Malformation of Lung, Congenital"/ or Congenital Cystic adenomatoid malformation of lung*.mp.	874
33	Renal Anomal*.mp.	1185
34	exp Urogenital Abnormalities/ or Genito-urinary Anomalies.mp.	57735
35	exp Maxillofacial Abnormalities/ or Maxillofacial Abnormalit*.mp.	28265
36	exp Mouth Abnormalities/ or Mouth Abnormalit*.mp.	24459
37	Umbilical Hernia.mp. or exp Hernia, Umbilical/	4200
38	Hirschsprung disease.mp. or exp Hirschsprung Disease/	4567
39	exp Megacolon/ or Ganglionic Megacolon.mp.	7053
40	Rectosigmoid aganglionosis.mp.	15

41	Colonic Aganglionosis.mp.	311
42	Intestinal Aganglionosis.mp.	190
43	Volvulus.mp. or exp Intestinal Volvulus/	8927
44	LMICs.mp.	2001
45	(low- and middle-income countr*).mp. [mp=title, abstract, original title, name of substance word, subject heading word, floating sub-heading word, keyword heading word, protocol supplementary concept word, rare disease supplementary concept word, unique identifier, synonyms]	10910
46	Low-resource setting*.mp.	2719
47	Underdeveloped Countries.mp.	817
48	low-income countries.mp.	4707
49	exp Developing Countries/ or Developing countr*.mp.	115302
50	middle-income countries.mp.	11136
51	limited resource setting*.mp.	253
52	Africa South of the Sahara.mp. or exp "Africa South of the Sahara"/	184102
53	Sub-Saharan Africa.mp. or exp "Africa South of the Sahara"/	190396
54	Less Resourced communities.mp.	1
55	Afghanistan.mp. or exp AFGHANISTAN/	6065
56	Albania.mp. or exp ALBANIA/	1182
57	Algeria.mp. or exp ALGERIA/	3621
58	American Samoa.mp.	354
59	Angola.mp. or exp ANGOLA/	1403
60	Argentina.mp. or exp ARGENTINA/	19610
61	Armenia.mp. or exp ARMENIA/	1676
62	Azerbaijan.mp. or exp AZERBAIJAN/	1765
63	Bangladesh.mp. or exp BANGLADESH/	12907
64	Belarus.mp. or exp "Republic of Belarus"/	2553
65	Belize.mp. or exp BELIZE/	868
66	Benin.mp. or exp BENIN/	3441
67	exp BHUTAN/ or Bhutan.mp.	628
68	Bolivia.mp. or exp BOLIVIA/	3594
69	(Bosnia and Herzegovina).mp. [mp=title, abstract, original title, name of substance word, subject heading word, floating sub-heading word, keyword heading word, protocol supplementary concept word, rare disease supplementary concept word, unique identifier, synonyms]	2477
70	Botswana.mp. or exp BOTSWANA/	2247
71	Brazil.mp. or exp BRAZIL/	101919
72	Bulgaria.mp. or exp BULGARIA/	7554
73	Burkina Faso.mp. or exp Burkina Faso/	3904
74	Burundi.mp. or exp BURUNDI/	871
75	Cabo Verde.mp. or exp Cabo Verde/	211
76	Cambodia.mp. or exp CAMBODIA/	4102
77	Cameroon.mp. or exp CAMEROON/	6804
78	Central African Republic.mp. or exp Central African Republic/	1096
79	Chad.mp. or exp CHAD/	1144
80	China.mp. or exp CHINA/	205799
81	Colombia.mp. or exp COLOMBIA/	12533
82	Comoros.mp. or exp COMOROS/	427
83	Democratic Republic of the Congo.mp. or exp "Democratic Republic of the Congo"/	4506
84	exp "Democratic Republic of the Congo"/ or DRC.mp.	4827
85	Republic of the Congo.mp. or exp Congo/	6176
86	Costa Rica.mp. or exp Costa Rica/	5088
87	Cote d'Ivoire.mp. or exp Cote d'Ivoire/	3527
88	Ivory Coast.mp. or exp Cote d'Ivoire/	3432

89	Croatia.mp. or exp CROATIA/	8261
90	Cuba.mp. or exp CUBA/	6343
91	Djibouti.mp. or exp DJIBOUTI/	372
92	Dominica.mp. or exp DOMINICA/	430
93	Dominican Republic.mp. or exp Dominican Republic/	2128
94	Ecuador.mp. or exp ECUADOR/	4722
95	exp EGYPT/ or Egypt.mp.	18702
96	El Salvador.mp. or exp El Salvador/	1319
97	Equatorial Guinea.mp. or exp Equatorial Guinea/	419
98	Eritrea.mp. or exp ERITREA/	518
99	Ethiopia.mp. or exp ETHIOPIA/	13354
100	Fiji.mp. or exp FIJI/	1620
101	Gabon.mp. or exp GABON/	1925
102	exp GAMBIA/ or Gambia.mp.	2958
103	exp "GEORGIA (REPUBLIC)"/ or exp GEORGIA/ or Georgia.mp.	15842
104	Ghana.mp. or exp GHANA/	9432
105	Grenada.mp. or exp GRENADA/	308
106	Guatemala.mp. or exp GUATEMALA/	3907
107	Guinea.mp. or exp GUINEA-BISSAU/ or exp EQUATORIAL GUINEA/ or exp GUINEA/ or exp PAPUA NEW GUINEA/ or exp NEW GUINEA/	160074
108	Guyana.mp. or exp GUYANA/	1136
109	Haiti.mp. or exp HAITI/	3707
110	Honduras.mp. or exp HONDURAS/	1736
111	India.mp. or exp INDIA/	129215
112	Indonesia.mp. or exp INDONESIA/	13107
113	exp Iran/ or Islamic Republic of Iran.mp.	21280
114	exp IRAQ/ or Iraq.mp.	9035
115	Jamaica.mp. or exp JAMAICA/	4427
116	Jordan.mp. or exp JORDAN/	6040
117	Kazakhstan.mp. or exp KAZAKHSTAN/	3153
118	Kenya.mp. or exp KENYA/	18798
119	Kiribati.mp. or exp Micronesia/	1934
120	Democratic People's Republic of Korea.mp. or exp "Democratic People's Republic of Korea"/	246
121	Kosovo.mp. or exp KOSOVO/	840
122	Kyrgyz Republic.mp. or exp Kyrgyzstan/	1225
123	exp Laos/ or Lao PDR.mp.	1876
124	Laos.mp. or exp LAOS/	2431
125	Lebanon.mp. or exp LEBANON/	5011
126	Lesotho.mp. or exp LESOTHO/	645
127	Liberia.mp. or exp LIBERIA/	1667
128	Libya.mp. or exp LIBYA/	1554
129	Macedonia Republic.mp. or exp "Macedonia (Republic)"/	475
130	Madagascar.mp. or exp MADAGASCAR/	4734
131	Malawi.mp. or exp MALAWI/	6317
132	Malaysia.mp. or exp MALAYSIA/	18390
133	Maldives.mp. or exp Indian Ocean Islands/	10595
134	Mali.mp. or exp MALI/	3511
135	Marshall Islands.mp. or exp Micronesia/	1959
136	Mauritania.mp. or exp MAURITANIA/	626
137	Mauritius.mp. or exp MAURITIUS/	953
138	exp MEXICO/ or Mexico.mp.	53282
139	Micronesia.mp. or exp MICRONESIA/	2243
140	Moldova.mp. or exp MOLDOVA/	920

141	Mongolia.mp. or exp MONGOLIA/	3809
142	Montenegro.mp. or exp MONTENEGRO/	749
143	Morocco.mp. or exp MOROCCO/	6855
144	Mozambique.mp. or exp MOZAMBIQUE/	3236
145	Myanmar.mp. or exp MYANMAR/	3198
146	Namibia.mp. or exp NAMIBIA	1502
147	Nauru.mp. or exp Micronesia/	1921
148	Nepal.mp. or exp NEPAL/	9337
149	Nicaragua.mp. or exp NICARAGUA/	1949
150	exp NIGER/ or Niger.mp.	12299
151	Nigeria.mp. or exp NIGERIA/	32627
152	Pakistan.mp. or exp PAKISTAN/	20153
153	exp PANAMA/ or Panama.mp.	4265
154	Papua New Guinea.mp. or exp Papua New Guinea/	5003
155	Paraguay.mp. or exp PARAGUAY/	1433
156	Peru.mp. or exp PERU/	11249
157	Philippines.mp. or exp PHILIPPINES/	10698
158	Romania.mp. or exp ROMANIA/	11521
159	exp Russia/ or Russian Federation.mp.	51376
160	Rwanda.mp. or exp RWANDA/	2883
161	exp AMERICAN SAMOA/ or Samoa.mp. or exp SAMOA/ or exp "INDEPENDENT STATE OF SAMOA"/	1024
162	(Sao Tome and Principe).mp. [mp=title, abstract, original title, name of substance word, subject heading word, floating sub-heading word, keyword heading word, protocol supplementary concept word, rare disease supplementary concept word, unique identifier, synonyms]	135
163	Senegal.mp. or exp SENEGAL/	7067
164	Serbia.mp. or exp SERBIA/	4574
165	Sierra Leone.mp. or exp Sierra Leone/	2103
166	Solomon Islands.mp. or exp Melanesia/	6193
167	Somalia.mp. or exp SOMALIA/	1968
168	Somaliland.mp.	136
169	South Africa.mp. or exp South Africa/	47075
170	South Sudan.mp. or exp South Sudan/	397
171	Sri Lanka.mp. or exp Sri Lanka/	7342
172	Saint Lucia.mp. or exp Saint Lucia/	114
173	(Saint Vincent and the Grenadines).mp. [mp=title, abstract, original title, name of substance word, subject heading word, floating sub-heading word, keyword heading word, protocol supplementary concept word, rare disease supplementary concept word, unique identifier, synonyms]	57
174	exp SUDAN/ or Sudan.mp.	8429
175	Suriname.mp. or exp SURINAME/	1098
176	Swaziland.mp. or exp SWAZILAND/	818
177	Syrian Arab Republic.mp.	73
178	Syria.mp. or exp SYRIA/	2407
179	Tajikistan.mp. or exp TAJIKISTAN/	910
180	Tanzania.mp. or exp TANZANIA/	12921
181	Thailand.mp. or exp THAILAND/	32186
182	exp Timor-Leste/ or Timor-Leste.mp.	311
183	East Timor.mp.	187
184	Togo.mp. or exp TOGO/	1491
185	Tonga.mp. or exp TONGA/	463
186	Tunisia.mp. or exp TUNISIA/	9057
187	exp TURKEY/ or Turkey.mp.	46898

188	Turkmenistan.mp. or exp TURKMENISTAN/	717
189	Tuvalu.mp. or exp Micronesia/	1884
190	Uganda.mp. or exp UGANDA/	13956
191	Ukraine.mp. or exp UKRAINE/	16702
192	Uzbekistan.mp. or exp UZBEKISTAN/	2161
193	Vanuatu.mp. or exp VANUATU/	629
194	Venezuela.mp. or exp VENEZUELA/	6620
195	exp VIETNAM/ or Vietnam.mp.	15919
196	(West Bank and Gaza).mp. [mp=title, abstract, original title, name of substance word, subject heading word, floating sub-heading word, keyword heading word, protocol supplementary concept word, rare disease supplementary concept word, unique identifier, synonyms]	194
197	Republic of Yemen.mp. or exp Yemen/	1264
198	Zambia.mp. or exp ZAMBIA/	5404
199	Zimbabwe.mp. or exp ZIMBABWE/	6879
200	Antenatal Diagnosis.mp.	2383
201	Prenatal Diagnosis.mp. or exp Prenatal Diagnosis/	74636
202	Antenatal Screening.mp.	1193
203	Prenatal Screening.mp.	2579
204	Antenatal Ultrasound.mp.	702
205	Prenatal Ultrasound.mp.	2203
206	Antenatal Ultrasonography.mp.	200
207	Prenatal Ultrasonography.mp. or exp Ultrasonography, Prenatal/	30427
208	1 or 2 or 3 or 4 or 5 or 6 or 7 or 8 or 9 or 10 or 11 or 12 or 13 or 14 or 15 or 16 or 17 or 18 or 19 or 20 or 21 or 22 or 23 or 24 or 25 or 26 or 27 or 28 or 29 or 30 or 31 or 32 or 33 or 34 or 35 or 36 or 37 or 38 or 39 or 40 or 41 or 42 or 43	607001
209	44 or 45 or 46 or 47 or 48 or 49 or 50 or 51 or 52 or 53 or 54 or 55 or 56 or 57 or 58 or 59 or 60 or 61 or 62 or 63 or 64 or 65 or 66 or 67 or 68 or 69 or 70 or 71 or 72 or 73 or 74 or 75 or 76 or 77 or 78 or 79 or 80 or 81 or 82 or 83 or 84 or 85 or 86 or 87 or 88 or 89 or 90 or 91 or 92 or 93 or 94 or 95 or 96 or 97 or 98 or 99 or 100 or 101 or 102 or 103 or 104 or 105 or 106 or 107 or 108 or 109 or 110 or 111 or 112 or 113 or 114 or 115 or 116 or 117 or 118 or 119 or 120 or 121 or 122 or 123 or 124 or 125 or 126 or 127 or 128 or 129 or 130 or 131 or 132 or 133 or 134 or 135 or 136 or 137 or 138 or 139 or 140 or 141 or 142 or 143 or 144 or 145 or 146 or 147 or 148 or 149 or 150 or 151 or 152 or 153 or 154 or 155 or 156 or 157 or 158 or 159 or 160 or 161 or 162 or 163 or 164 or 165 or 166 or 167 or 168 or 169 or 170 or 171 or 172 or 173 or 174 or 175 or 176 or 177 or 178 or 179 or 180 or 181 or 182 or 183 or 184 or 185 or 186 or 187 or 188 or 189 or 190 or 191 or 192 or 193 or 194 or 195 or 196 or 197 or 198 or 199 (1385344)	1385344
210	200 or 201 or 202 or 203 or 204 or 205 or 206 or 207	77525
211	208 and 209 and 210	896
