# Supplementary material for: Investigating the use of ultrasonography for the antenatal diagnosis of structural congenital anomalies in low-income and middle-income countries: a systematic review
Source: BMJ Paediatr Open. 2020 Aug 20;4(1):e000684. doi: 10.1136/bmjpo-2020-000684 (PMC7443309; doi:10.1136/bmjpo-2020-000684)
Supplement: Supplementary data [file bmjpo-2020-000684supp004.pdf]

Supplementary File 4

Investigating the Use of Ultrasonography for the Antenatal Diagnosis of Structural Congenital Anomalies in Low- and Middle-Income Countries: A Systematic Review

Data Extraction Template

**Reference**	Reference (Full)
	Title
	Author(s)
	Publication Date
	Year of Publication
	Journal
**Place of Study**	Hospital
	City
	Country
	Continent
**Study Type**	Type of Study (RCT, Cohort, Case-Control, etc.)
**Publication Status**	Has Study Been Published?
**Study Population**	Number of Patients
	Patient Cohort (Institutional, Multi-Center, Regional, National, etc.)
	Patient Cohort (All Patients Presenting with a Condition vs. Those with an Antenatal Diagnosis)
	Gestational Age (Mean, Median)
	Type of Anomaly

**Diagnosis**	Type of Ultrasound (Abdominal vs. Transvaginal; 2D vs. 3D)
	Percent Receiving Antenatal Ultrasound
	Percent with Any Anomaly Detected
	Percent with Accurate Antenatal Diagnosis
	Incidence of Anomaly
	Training of Ultrasonographer
	Referral Rate for Further Antenatal Care
	Referral Rate to Tertiary Clinic
	Percentage Born in a Pediatric Surgery Center
**Outcomes**	Mortality Rate in Live Births
	Termination Rate
	Complications (Using Clavien-Dindo Classification)
	Additional Notes Regarding the Effect of Antenatal Diagnosis on Outcomes
	Other Benefits of Antenatal Diagnosis (i.e., Parental Counselling, Option for Termination, etc.)
	Comments Regarding Policy
	Additional Notes/Other Useful Information
