# Supplementary material for: Investigating the use of ultrasonography for the antenatal diagnosis of structural congenital anomalies in low-income and middle-income countries: a systematic review
Source: BMJ Paediatr Open. 2020 Aug 20;4(1):e000684. doi: 10.1136/bmjpo-2020-000684 (PMC7443309; doi:10.1136/bmjpo-2020-000684)
Supplement: Supplementary data [file bmjpo-2020-000684supp005.pdf]

Supplementary File 5

Investigating the Use of Ultrasonography for the Antenatal Diagnosis of Structural Congenital Anomalies in Low- and Middle-Income Countries: A Systematic Review

Table of Studies Excluded By Language

**Language**	**Number of Studies**
Chinese	23
Russian	16
Spanish	15
French	12
Portuguese	6
Romanian	1
**Total**	**73**
