# Supplementary material for: Investigating the use of ultrasonography for the antenatal diagnosis of structural congenital anomalies in low-income and middle-income countries: a systematic review
Source: BMJ Paediatr Open. 2020 Aug 20;4(1):e000684. doi: 10.1136/bmjpo-2020-000684 (PMC7443309; doi:10.1136/bmjpo-2020-000684)
Supplement: Supplementary data [file bmjpo-2020-000684supp006.pdf]

Supplementary File 6

Investigating the Use of Ultrasonography for the Antenatal Diagnosis of Structural Congenital Anomalies in Low- and Middle-Income Countries: A Systematic Review

List of Countries Represented in Systematic Review Data Extraction

Country	No. of Studies (%)
India	17 (17.0%)
China	10 (10.0%)
Turkey	10 (10.0%)
Brazil	9 (9.0%)
Nigeria	9 (9.0%)
Romania	7 (7.0%)
Iran	6 (6.0%)
Argentina	4 (4.0%)
Colombia	4 (4.0%)
Thailand	4 (4.0%)
South Africa	2 (2.0%)
Cameroon	1 (1.0%)
Chile	1 (1.0%)
Egypt	1 (1.0%)
Ethiopia	1 (1.0%)
Jamaica	1 (1.0%)
Lebanon	1 (1.0%)
Malaysia	1 (1.0%)
Mexico	1 (1.0%)
Pakistan	1 (1.0%)
Russia	1 (1.0%)
Sri Lanka	1 (1.0%)
Taiwan	1 (1.0%)
Tanzania	1 (1.0%)
Tunisia	1 (1.0%)
Uganda	1 (1.0%)
Venezuela	1 (1.0%)
Vietnam	1 (1.0%)
Zimbabwe	1 (1.0%)
**Total**	**100**

This table represents all of the countries from the 97 included studies. 96 studies were conducted in a single country and 1 study was conducted across four countries.
