# Supplementary material for: Investigating the use of ultrasonography for the antenatal diagnosis of structural congenital anomalies in low-income and middle-income countries: a systematic review
Source: BMJ Paediatr Open. 2020 Aug 20;4(1):e000684. doi: 10.1136/bmjpo-2020-000684 (PMC7443309; doi:10.1136/bmjpo-2020-000684)
Supplement: Author's manuscript [file bmjpo-2020-000684.draft_revisions.pdf]

BMJ Paediatrics Open

BMJ Paediatrics Open is committed to open peer review. As part of this commitment we make the peer review history of every article we publish publicly available.

When an article is published we post the peer reviewers' comments and the authors' responses online. We also post the versions of the paper that were used during peer review. These are the versions that the peer review comments apply to.

The versions of the paper that follow are the versions that were submitted during the peer review process. They are not the versions of record or the final published versions. They should not be cited or distributed as the published version of this manuscript.

BMJ Paediatrics Open is an open access journal and the full, final, typeset and author-corrected version of record of the manuscript is available on our site with no access controls, subscription charges or pay-per-view fees (http://bmjpaedsopen.bmj.com).

If you have any questions on BMJ Paediatrics Open's open peer review process please email info.bmjpo@bmj.com

BMJ Paediatrics Open

Investigating the Use of Ultrasonography for the Antenatal Diagnosis of Structural Congenital Anomalies in Low- and Middle-Income Countries: A Systematic Review

Journal:	*BMJ Paediatrics Open*
Manuscript ID	bmjpo-2020-000684
Article Type:	Original research
Date Submitted by the Author:	11-Mar-2020

Complete List of Authors:	Goley, Stephanie; King's College London, Department of Global Health & Social Medicine<br>Sakula-Barry, Sidonie; World Cancer Research Fund<br>Adofo-Ansong, Nana; Mafikeng Provincial Hospital<br>Isaaya Ntawunga, Laurence; University of Rwanda, Rwanda Spina Bifida and Hydrocephalus Relief<br>Tekyiwa Botchway, Maame; University of the Witwatersrand<br>Kelly, Ann; King's College London, Department of Global Health & Social Medicine<br>Wright, Naomi; King's College London, King’s Centre for Global Health and Health Partnerships

Keywords:	Screening, Neonatology, Congenital Abnorm

SCHOLARONE™
Manuscripts

I, the Submitting Author has the right to grant and does grant on behalf of all authors of the Work (as defined in the below author licence), an exclusive licence and/or a non-exclusive licence for contributions from authors who are: i) UK Crown employees; ii) where BMJ has agreed a CC-BY licence shall apply, and/or iii) in accordance with the terms applicable for US Federal Government officers or employees acting as part of their official duties; on a worldwide, perpetual, irrevocable, royalty-free basis to BMJ Publishing Group Ltd ("BMJ") its licensees and where the relevant Journal is co-owned by BMJ to the co-owners of the Journal, to publish the Work in this journal and any other BMJ products and to exploit all rights, as set out in our licence.

The Submitting Author accepts and understands that any supply made under these terms is made by BMJ to the Submitting Author unless you are acting as an employee on behalf of your employer or a postgraduate student of an affiliated institution which is paying any applicable article publishing charge ("APC") for Open Access articles. Where the Submitting Author wishes to make the Work available on an Open Access basis (and intends to pay the relevant APC), the terms of reuse of such Open Access shall be governed by a Creative Commons licence – details of these licences and which Creative Commons licence will apply to this Work are set out in our licence referred to above.

Other than as permitted in any relevant BMJ Author's Self Archiving Policies, I confirm this Work has not been accepted for publication elsewhere, is not being considered for publication elsewhere and does not duplicate material already published. I confirm all authors consent to publication of this Work and authorise the granting of this licence.

Confidential: For Review Only

Investigating the Use of Ultrasonography for the Antenatal Diagnosis of Structural Congenital Anomalies in Low- and Middle-Income Countries: A Systematic Review

Stephanie Goley[1], Sidonie Sakula-Barry[2], Nana Adofo-Ansong[3], Laurence Isaaya Ntawunga[4], Maame Tekyiwa Botchway[5], Ann H. Kelly[1], Naomi Wright[6]

[1]Department of Global Health & Social Medicine, King's College London, London, UK
[2]World Cancer Research Fund UK, London, UK
[3]Mafikeng Provincial Hospital, Mafikeng, South Africa
[4]Rwanda Spina Bifida and Hydrocephalus Relief, University of Rwanda, Kigali, Rwanda
[5]University of Witwatersrand, Johannesburg, South Africa
[6]King's Centre for Global Health and Health Partnerships, School of Population Health and Environmental Sciences, King's College London, London, UK

Corresponding Author:

Stephanie Goley, BA MSc
Department of Global Health & Social Medicine, King's College London, London, UK
Correspondence to: Centre for Global Health and Health Partnerships, Suite 2.13 Weston Education Centre, Cutcombe Road, London, SE5 9RJ
Telephone: +1 (815) 830-5360

Authors' Email Addresses:

info@stephgoley.com
s.sakulabarry@gmail.com
adfnan001@myuct.ac.za
rsbhrelief@gmail.com
1596010@students.wits.ac.za
ann.kelly@kcl.ac.uk
naomiwright@doctors.org.uk

Confidential: For Review Only

What is known?

- Congenital anomalies are the fifth leading cause of death in children under 5-years of age globally.
- 97% of congenital anomaly deaths occur in LMICs, many of which may be preventable with antenatal diagnosis and planned surgical intervention following birth.
- Antenatal ultrasound examinations in HICs are commonplace and highly accurate, but accessibility and effectiveness are limited in LMICs.

What this study adds?

- Rates of antenatal ultrasound examination vary significantly in LMICs, ranging from 6.8% to 97.1%.
- There is significant variation in the accuracy of antenatal diagnosis in LMICs, with detection rates varying from 0% to 100% (mean of 25.6% in Africa).
- Available data suggests that the level of ultrasonographer training may affect the accuracy of diagnosis, but more research must be done to be conclusive.

Confidential: For Review Only

Abstract

Background: Congenital anomalies are the 5th leading cause of under-5 mortality globally. The greatest burden is faced by those in developing countries, where over 95% of deaths occur. Many of these deaths may have been preventable through antenatal diagnosis and early intervention. This systematic literature review investigates the use of antenatal ultrasound to diagnose congenital anomalies and improve the health outcomes of infants in low- and middle-income countries (LMICs).

Methods: A systematic literature review was conducted using three search strings: 1) structural congenital anomalies; 2) LMICs; and 3) antenatal diagnosis. The search was conducted on the following electronic databases: MEDLINE, EMBASE, PubMed, and the Cochrane Library. Title, abstract and full-text screening was undertaken in duplicate by two reviewers independently using Covidence. Consensus among the wider authorship was sought for discrepancies. The primary analysis focused on the availability and effectiveness of antenatal ultrasound for structural congenital anomalies. Secondary outcomes included neonatal morbidity and mortality, termination rates, and referral rates for further antenatal care. Descriptive statistics and a narrative synthesis have been reported.

Results: The search produced 4,062 articles, of which 154 met all inclusion criteria and were relevant for data extraction. The percentage of women receiving antenatal ultrasound examinations varied from 6.8% to 98.2%. Detection rates ranged from 0% to 100%, with a mean of 25.6% in Africa. The level of training of the ultrasound provider may be an important factor, however, there is insignificant data to make a firm conclusion. Only five articles compared morbidity and mortality outcomes, with inconclusive results. Significant variations in termination rates were found. No articles addressed referral rates.

Conclusion: Antenatal detection of congenital anomalies remains highly variable across LMICs and is particularly low in sub-Saharan Africa. Further research is required to investigate the role of antenatal diagnosis for improving survival from congenital anomalies in LMICs.

PROSPERO Registration ID: CRD42019105620

Confidential: For Review Only

Introduction

Congenital anomalies are one of the leading causes of neonatal morbidity and mortality globally. The greatest burden of disease is faced by those in low- and middle-income countries (LMICs), as 94% of congenital anomalies occur in these regions [1]. Congenital anomalies comprise 9% of the total global burden for surgical diseases and account for a total of 57.7 million disability-adjusted life years (DALYs) lost annually across the globe [2]. Recent estimates suggest that approximately 303,000 neonates die annually from congenital anomalies before reaching just four weeks of age [3]. However, many experts believe that these statistics are conservative, as there is a significant lack of data on the prevalence of congenital anomalies in LMICs. The data that is available is likely to be downwardly skewed by underreporting, a lack of registries for congenital anomalies, and unreliable medical records in many LMICs. Further, some congenital anomalies that are not visible externally, such as congenital diaphragmatic hernia or esophageal atresia, may not be diagnosed and hence recorded as the cause of death.

There are numerous factors which contribute to the high burden of congenital anomalies in LMICs, especially sub-Saharan Africa (SSA). Many pregnant women in LMICs face higher levels of micro- and macronutrient deficiencies which are associated with the occurrence of birth defects. This can be seen specifically in the case of folate deficiencies leading to neural tube defects [4]. Culturally-informed practices such as fasting during pregnancy, the ingestion of certain herbal remedies or the use of medications which may be teratogenic could have a role to play, as could an increased prevalence of infections during pregnancy [1, 5]. There is also an increased fertility rate of 4-6% in SSA which results in a higher incidence of neonates born with congenital anomalies [6].

Early detection and diagnosis of congenital anomalies is critical. The high survival rates for neonates diagnosed with congenital anomalies in high-income countries (HICs) have shown that many conditions can be treated with immediate surgical intervention following birth. Unfortunately, emergency neonatal surgical care is often not available after birth in many LMICs, particularly for conditions involving the gastrointestinal tract [7]. In the case of gastroschisis, for example, the mortality rate for neonates in HICs is less than 5% [8]; however, in many LMICs, the mortality rate is as high as 100% [9, 10]. Although ultrasound technology is widely available in HICs, a number of factors constrain the use of effective antenatal ultrasound in LMICs.

This study primarily aimed to systematically investigate the availability and effectiveness of antenatal ultrasound in the diagnosis of structural congenital anomalies in LMICs. It further aimed to evaluate the effects of antenatal ultrasound diagnosis on mortality and morbidity

outcomes, termination rates and referral for further antenatal care and management planning. Additionally, the level of training of ultrasonographers undertaking antenatal scans was assessed and relevant antenatal ultrasound policy in LMICs. This information is vital to help clarify the existing disparities in antenatal ultrasound provision and the potential benefits for improved health outcomes.

Methodology

Preferred Reporting Items for Systematic review and Meta-Analysis (PRISMA) guidelines have been followed when conducting this systematic review (Supplementary File 1) [11, 12]. A protocol for this systematic review was published in *BMJ Paediatrics Open* [13].

Patient and Public Involvement

Given that this is a systematic literature review, there was no patient or public involvement for the collection of data and literature review. Public involvement will be important for prioritizing antenatal ultrasound on the political agenda and improving antenatal care programs. To disseminate the results of this study, international charities and organizations involving structural congenital anomalies will be approached to assist in circulation.

Search Strategy

A search was conducted using three search strings (Table 1). Search string 1 encompasses structural congenital anomalies. Search string 2 focuses on the setting (LMICs). Search string 3 incorporates antenatal diagnosis of structural congenital anomalies, with a focus on the use of ultrasound for detection. Using the Ovid program, an electronic database search was conducted on MEDLINE, EMBASE, PubMed, and the Cochrane Library. These searches were filtered to only include studies with human subjects. An example of the search in MEDLINE can be found in Supplementary File 2. Only fetuses with a structural congenital anomaly as listed in search string 1 were included. Only studies from LMICs were included; these were limited to the English language. Studies with less than five patients were excluded.

Table 1: Search Strings

Search String 1	Search String 2	Search String 3
Congenital Anomalies, Congenital Abnormalities, Congenital Malformation, Fetal Malformation, Birth Defects, Anencephaly, Conjoined Twins, Congenital Heart Defects, Anorectal Malformations, Anal Stenosis, Anal Atresia,	LMICs, Low- and Middle-Income Countries, Developing Countries, Low-Resource Settings, Underdeveloped Countries, Low-Income Countries, Middle-Income Countries, Limited Resource Settings, Africa South of the Sahara, Sub-Saharan Africa, Less Resourced Communities, Afghanistan, Albania, Algeria, American Samoa, Angola, Argentina, Armenia, Azerbaijan, Bangladesh, Belarus, Belize, Benin, Bhutan, Bolivia, Bosnia and	Antenatal Diagnosis, Prenatal Diagnosis, Antenatal Screening, Prenatal Screening, Antenatal Ultrasound, Prenatal Ultrasound, Antenatal Ultrasonography, Prenatal Ultrasonography

Confidential: For Review Only

Imperforate Anus, Biliary Atresia, Choledochal Cyst, Diaphragmatic Eventration, Esophageal Atresia, Tracheoesophageal Fistula, Intestinal Atresia, Duodenal Obstruction, Duodenal Atresia, Colonic Atresia, Malrotation, Apple Peel Syndrome, Congenital Diaphragmatic Hernias, Gastroschisis, Abdominal Wall Defects, Exomphalos, Omphalocele, Congenital Limb Deformities, Neural Tube Defects, Bronchogenic Cyst, Bronchopulmonary Sequestration, Congenital Cystic Adenomatoid Malformation of Lung, Renal Anomalies, Genito-urinary Anomalies, Maxillofacial Abnormalities, Mouth Abnormalities, Umbilical Hernia, Hirschsprung Disease, Ganglionic Megacolon, Rectosigmoid Aganglionosis, Colonic Aganglionosis, Intestinal Aganglionosis, Volvulus, Intestinal Volvulus	Herzegovina, Botswana, Brazil, Bulgaria, Burkina Faso, Burundi, Cabo Verde, Cambodia, Cameroon, Central African Republic, Chad, China, Colombia, Comoros, Democratic Republic of the Congo, DRC, Republic of the Congo, Costa Rica, Cote d'Ivoire, Ivory Coast, Croatia, Cuba, Djibouti, Dominica, Dominican Republic, Ecuador, Egypt, El Salvador, Equatorial Guinea, Eritrea, Ethiopia, Fiji, Gabon, Gambia, Georgia, Ghana, Grenada, Guatemala, Guinea, Guinea-Bissau, Guyana, Haiti, Honduras, India, Indonesia, Islamic Republic of Iran, Iraq, Jamaica, Jordan, Kazakhstan, Kenya, Kiribati, Democratic People's Republic of Korea, Kosovo, Kyrgyz Republic, Lao PDR, Laos, Lebanon, Lesotho, Liberia, Libya, Macedonia Republic, Madagascar, Malawi, Malaysia, Maldives, Mali, Marshall Islands, Mauritania, Mauritius, Mexico, Micronesia, Moldova, Mongolia, Montenegro, Morocco, Mozambique, Myanmar, Namibia, Nauru, Nepal, Nicaragua, Niger, Nigeria, Pakistan, Panama, Papua New Guinea, Paraguay, Peru, Philippines, Romania, Russian Federation, Rwanda, Samoa, Sao Tome and Principe, Senegal, Serbia, Sierra Leone, Solomon Islands, Somalia, Somaliland, South Africa, South Sudan, Sri Lanka, Saint Lucia, Saint Vincent and the Grenadines, Sudan, Suriname, Swaziland, Syrian Arab Republic, Syria, Tajikistan, Tanzania, Thailand, Timor-Leste, East Timor, Togo, Tonga, Tunisia, Turkey, Turkmenistan, Tuvalu, Uganda, Ukraine, Uzbekistan, Vanuatu, Venezuela, Vietnam, West Bank and Gaza, Republic of Yemen, Zambia, Zimbabwe	

Confidential: For Review Only

A further search was conducted on the WHO website to identify relevant grey literature, particularly related to antenatal ultrasound policy. The following terms were searched in the WHO Reproductive Health Library: ultrasound, ultrasonography, congenital anomalies, congenital abnormalities, congenital anomaly, congenital abnormality, birth defect, antenatal detection, prenatal detection, antenatal diagnosis, and prenatal diagnosis. Following the search of each term, the results were expanded using a snowball strategy in order to ensure the inclusion of all relevant data.

Data Extraction and Synthesis

Data extraction was undertaken by the principal investigator. The data extraction table can be found in Supplementary File 3. The results are presented using descriptive statistics (range and mean) and narrative synthesis.

Results

Study Screening

The search produced 4,062 articles. Of these, 745 duplicates were removed. The remaining 3,317 articles underwent an abstract and title review with two independent reviewers. Of the 3,317 articles screened, 2,826 were excluded. 491 articles were then reviewed by two independent reviewers in full-text. At this stage, 316 articles were excluded (Table 2).

Table 2: Articles Excluded Based on Language

**Language**	**Number of Articles**
Chinese	23
Russian	16
Spanish	15
French	12
Portuguese	6
Romanian	1
**Total**	**73**

175 articles were found to meet all inclusion criteria listed in the search strings. Of these, 21 provided no data relevant to the study and thus were excluded. 154 studies were included in the data extraction phase (Figure 1, Supplementary File 4). Although all LMICs as defined by the World Bank were included in the search, not all countries yielded results in the text screening. 138 LMICs were included in the literature search, however, only 35 countries (24.4%) had any data that met the inclusion criteria (Figure 2, Supplementary File 5).

It is also notable that 109 studies (63%) were conducted on an institutional level. Thus, while the data from these studies provides important information from the countries of this review, they are by no means a representative sample of an entire country or even an entire city. Each article

Confidential: For Review Only

also varied widely in the information it provided, ranging from antenatal detection rates to policy analysis. Given the heterogeneity of data extrapolated from these articles, it was not feasible to perform a meta-analysis.

Percent of Women Receiving Antenatal Ultrasound

Twenty-four of the 154 studies discuss the percentage of women receiving antenatal ultrasound examinations. The data suggests significant variation ranging from 6.8% to 98.2%, with particularly low examination rates in African countries. In Africa, the average percentage of women receiving an antenatal ultrasound examination is as follows: 6.8% in Tanzania, 12.1% in Zimbabwe, 23.6% in South Africa, 24% in Uganda, 32.8% in Cameroon and 77.8% in Nigeria [14-23]. It is important to note, however, that only 10 studies addressed the provision rates of antenatal ultrasound in Africa, so this may not be truly representative of each respective country.

Data suggests that when women do attend antenatal clinic appointments, they are often not receiving antenatal ultrasound examinations. Sekabira and Hadley revealed that 75% of participants in their study had attended antenatal clinic appointments, but just 23.6% had been given an examination via antenatal ultrasound [16]. These findings were mirrored in a similar study conducted in Kampala, Uganda where 93% of pregnant women had received antenatal care, but only 24% of these women had antenatal ultrasound scans performed [17].

However, not all studies reported low rates of antenatal ultrasound provision. One study in Chiang Mai, Thailand reported that 91.3% had received a scan [24]. In two Nigerian studies, antenatal ultrasound rates were as high as 80% and 91% amongst their cohorts [21, 22]. Despite high instances of scans, both studies reported low rates of accurate congenital anomaly detection, at less than 14% and 2%, respectively [21, 22].

Effectiveness of Antenatal Ultrasound

Seventy-five of the 154 studies reported the detection rates of structural congenital anomalies via antenatal ultrasound. Among these studies, there are substantial variations in the detection rates with little correlation to geographical region or type of anomaly. Detection rates varied from 0% to 100%, with a mean of 25.6% in Africa. In China detection rates ranged from 17.6% to 90.5%, while detection rates in Brazil ranged from 4.2% to 82.2%, and in India ranged from 8% to 100% [25-30]. In a study from Brazil, gastroschisis was diagnosed in 82.2% of patients, while a study in Romania reported detection of just 11.4% of cases [28, 31]. In China, one study reported 81.2% antenatal ultrasound detection of encephalocele, while a study in India reported 0% detection for the same condition [32, 33].

In a Malaysian study, it was shown that although 93% of women received scans, just 14.2% of all structural congenital anomalies were detected [34]. Another study in Iran reported that 97.1% of patients received at least one ultrasound scan during pregnancy [35]. Despite the high percentage of women having ultrasound scans, only 24.3% of patients with myelomeningocele were detected [35]. This suggests that even when antenatal ultrasound rates are high, the ultrasound scan may not be effective at detecting congenital anomalies.

Confidential: For Review Only

Training of Personnel Performing Ultrasound Examination

Only 16 of the 154 studies mentioned the level of training of the sonographer. Many of these 16 articles briefly mentioned that the scans were performed by 'experienced sonographers,' but gave no indication as to the level of training these providers had received. In one study in Turkey, for instance, 90.5% of major structural defects were detected when the scans were performed by "the same experienced radiologist" [36]. Of these, 73.7% of anomalies were detected early at the first scan, at an average of 13 weeks and 1 day, while 26.3% were detected late at the second scan, at an average of 19 weeks 2 days [36]. In another study in Turkey, antenatal ultrasound detected 96.2% of upper extremity malformations [37]. The authors indicated that all ultrasound examinations were performed by two specialists in maternal-fetal diagnostics, but gave no specification as to what precisely this entails [37]. A third study, also conducted at a hospital in Turkey, reported an antenatal detection rate of 97.4% when scans were performed by obstetricians specializing in antenatal diagnosis [38]. These physicians held accreditations in performing 11-14 week assessments for at least five years before participating in the study [38].

Similar findings were reported in several studies conducted in other countries. In a study conducted in Jamaica, 77% of anomalies were detected and the scans were conducted by eight OB/GYN residents who each had been training for at least two years [39]. In China, an investigation showed that 77.8% of limb deformities were detected [40]. These scans were conducted by 10 certified physicians who each had over five years of experience in performing antenatal ultrasounds [40]. In one study in Nigeria, 93.8% of anomalies were detected [41]. The authors of this study indicated that all ultrasound exams were performed by a consultant radiologist who had received extensive training in antenatal ultrasonography from a highly-respected medical center in the United Kingdom [41].

However, multiple studies have reported that many of the personnel performing antenatal ultrasounds in LMICs have limited training. One study conducted in select sub-Saharan countries found that only 40.4% of sonographers had completed a short course, while 38.3% had received no formal ultrasound training [42]. In a study conducted by Wesonga et al. looking at neonates presenting with gastroschisis in Uganda, 93% of mothers in the cohort presented for antenatal care and 24% received an antenatal ultrasound [17]. However, only two fetuses received an antenatal diagnosis of an abdominal wall defect and one was incorrectly diagnosed with omphalocele [17]. The ultrasound providers in this study were noted to hold a diploma with no other formal training.

It is worth noting that many of the studies showing greater detection rates were conducted in Turkey, which may have alternative explanations for the high detection rates. Overall, the lack of information regarding sonographer training makes it difficult to accurately assess the role that training may have in the detection of structural congenital anomalies. While the results are inconclusive, some inferences can be drawn from the studies where training was mentioned as to its impact on antenatal detection. Further research is necessary to confirm these preliminary conclusions and to elucidate the level of training that allows for accurate detection.

Morbidity and Mortality Outcomes

Confidential: For Review Only

Of the 154 studies that underwent data extraction, only five produced any data comparing the morbidity and mortality outcomes between neonates with an antenatal diagnosis versus neonates with a postnatal diagnosis. Given the insufficient level of available data, it is clear that more research must be conducted to provide a deeper understanding of the impact of antenatal ultrasound on neonatal and childhood morbidity and mortality outcomes in LMICs. From the available data in the five studies included, no trends emerged and thus the data is inconclusive. However, the data provided from each of the studies offers valuable insights which may help in devising future research.

One study by Vilela et al. in Brazil reported that prenatal diagnosis of gastroschisis was associated with a 70% reduction in mortality risk [43]. The authors noted that 80% of the neonates in their study with an antenatal diagnosis survived, compared to 33.3% of neonates without an antenatal diagnosis [43]. Of the group without antenatal diagnosis, 90% of deaths were attributed to sepsis [43].

In another study conducted at Kasturba Medical College Hospital in India, 11 out of 16 neonates with congenital diaphragmatic hernias underwent ultrasonography [44]. Four (25%) were diagnosed antenatally, while 12 (75%) were diagnosed after birth [44]. After one year, 0/4 (0%) of the antenatally diagnosed neonates had survived, compared to 9/12 (75%) of the postnatally diagnosed neonates [44]. The authors note that the higher level of mortality amongst the antenatally diagnosed neonates could be attributed to a higher severity of the condition resulting in them being more easily detectable, an association that has also been reported in high-income country data regarding congenital diaphragmatic hernia in particular [44].

Of note, no studies reported the effect of antenatal ultrasound on morbidity outcomes for patients with congenital anomalies.

Termination Rates

Termination rates varied significantly, from 6.35% of mothers opting for pregnancy termination for renal anomalies in India, to 98.3% opting for termination of neural tube defects in rural China [45, 46]. There was significant variation in termination rates between studies for fatal anomalies. For example, a study in Sao Paulo, Brazil noted that 41.4% of mothers terminated cases of anencephaly, whereas in Mumbai, India 28.6% of cases with fatal renal anomalies were terminated [47, 48]. In Nigeria, 80% of fatal anomaly cases were terminated [41]. Some studies conducted in countries with more stringent termination policies noted that not all termination requests were granted. For example, one study in Sao Paulo, Brazil noted that 19 cases of conjoined twins which were deemed fatal applied for termination, but only 14 were granted [49].

Referral Rates for Further Antenatal Care and Management Planning

No studies addressed this issue.

Policy Assessment

Confidential: For Review Only

Currently, there is a lack of regulation across sub-Saharan Africa in regards to antenatal ultrasound. WHO guidelines suggest the need for one antenatal ultrasound scan prior to 24 weeks gestation [50]. Of note, many of the congenital anomalies detected antenatally in this review were not diagnosed until after 24 weeks gestation. This may be explained by a lack of or limited ultrasonographer training or late presentation of the women for scanning/uncertainty of gestational age. The WHO recommends that ultrasound trainees receive at least 3-6 months of training, culminating in 300-500 ultrasound examinations [51]. There is a lack of regulation of training, particularly amongst private ultrasound providers.

Discussion

The use of ultrasound technology in LMICs has significantly increased in recent years, as ultrasound machines have become more compact, transportable, and affordable [52]. Yet, a great number of congenital anomalies that can be detected antenatally via ultrasound go undiagnosed. Factors identified as barriers to effective ultrasound use include limited training, equipment shortages, faulty ultrasound equipment, and lack of maintenance services [52]. Many ultrasound exams, particularly in developing countries, are performed by practitioners with limited formal training. One study found that the majority of ultrasound providers in LMICs do not have the minimum training as set by the WHO [53]. It is therefore likely that many practitioners in LMICs are unable to accurately detect congenital anomalies via ultrasound scan. Even where such training exists, lack of regulation means that screening for congenital anomalies may not be routine amongst antenatal ultrasound examinations taking place.

While global recommendations from the WHO specifically address the use of ultrasound technology, this is not always implemented in practice. Without an explicit consideration of how to address this problem, many congenital anomalies will remain undetected. In recent years, higher global priority has been given to neonatal health. Following the conclusion of the Millennium Development Goals in 2015, 17 new Sustainable Development Goals (SDGs) were developed to continue the success of the program in another 15-year span [54]. SDG 3 aims to ensure healthy lives and promote well-being for all at all ages. As part of this goal, there is a sub-goal to end all preventable under-five deaths with the specific aim to reduce neonatal mortality in every country to 12 per 1,000 live births [54]. In addition to the development goals set by the UN, the WHO has devised new strategies and polices focusing on improving neonatal healthcare around the world. In 2010, the WHO released the Sixty-Third World Health Assembly Report on Birth Defects, which aimed to further elucidate the definition, epidemiology, burden, and interventions for birth defects [55].

Antenatal ultrasound is both vital for defining the burden of congenital anomalies in LMICs globally and for reducing associated mortality through the provision of planned deliveries at pediatric surgery centers that can provide the appropriate care at birth. There must be a focus on implementation of the WHO guidelines recommending the provision of at least one antenatal ultrasound scan, including anomaly detection, for all women in the world – work at the Ministry of Health and service delivery levels is required [50]. Furthermore, regulation of such service provision is required across both government and private sectors to ensure quality and hence optimized detection rates. Finally, detection of an anomaly must be followed by a referral for antenatal counselling by a pediatric surgical team, the offer of termination for conditions which

Confidential: For Review Only

are incompatible with life in regions where this is culturally acceptable and importantly delivery at a tertiary center, which can provide appropriate surgical care at birth for those who need it. Once this action is taken, the positive impacts on neonatal and maternal healthcare may be profound.

Although this study is useful in determining that interventions must be put in place to improve availability of effective antenatal ultrasound in LMICs and that more research must be done, it is also important to note a few of the limitations of the study. First, only articles in English were included in this systematic review, which may exclude other relevant studies. Furthermore, it is important to note that antenatal ultrasound has further diagnostic capabilities, such as detecting abnormal growth or improper placental position and this review only focused on the detection of structural congenital anomalies. Further studies could also include other uses of antenatal ultrasound for improving neonatal and indeed maternal health outcomes.

Conclusion

The data from this review suggests that the percentage of women in LMICs who receive an antenatal ultrasound examination varies considerably and is particularly low in sub-Saharan African countries. Even when antenatal ultrasound scans are performed, accurate detection rates are often very low. The level of training (and the type of training) of the sonographer may be indicative of the accuracy of diagnosis. Only five studies delineated the morbidity and mortality outcomes amongst neonates with an antenatal diagnosis compared to postnatal diagnosis. Hence, although the benefits of antenatal ultrasound are widely documented in HICs, data is severely lacking in LMICs. It is clear that the use of antenatal ultrasound in LMICs is not maximized to its highest potential.

Acknowledgements

We would like to thank the library services at King's College London for help with the systematic review process.

Contributors

NW conceived the idea for this study. NW, AK, and SG devised the study design. SG, SSB, NAA, LIN, MTB, and NW performed the literature review. SG drafted the manuscript with significant contributions from NW and AK. All authors reviewed and approval the final submitted manuscript.

Confidential: For Review Only

Funding

NW receives funding from the Wellcome Trust to undertake a Clinical PhD in Global Health at King's Centre for Global Health and Health Partnerships, King's College London (Funder Reference: 203905/Z/16/Z).

Competing Interests

The authors have no competing interests to declare.

References

1. Sitkin, N.A., et al., *Congenital anomalies in low- and middle-income countries: the unborn child of global surgery.* World J Surg, 2015. **39**(1): p. 36-40.
2. Farmer, D., et al., *Surgical Interventions for Congenital Anomalies*, in *Essential Surgery: Disease Control Priorities, Third Edition (Volume 1)*, H.T. Debas, et al., Editors. 2015: Washington (DC).
3. WHO. *WHO Fact Sheets - Congenital Anomalies* 2015 May 12, 2018]; Available from: http://www.who.int/news-room/fact-sheets/detail/congenital-anomalies.
4. Darnton-Hill, I. and U.C. Mkparu, *Micronutrients in pregnancy in low- and middle-income countries.* Nutrients, 2015. **7**(3): p. 1744-68.
5. Flores, A., et al., *Building capacity for birth defects surveillance in Africa: Implementation of an intermediate birth defects surveillance workshop.* J Glob Health Perspect, 2015. **2015**.
6. Chirdan, L.B., et al., *Challenges of training and delivery of pediatric surgical services in Africa.* J Pediatr Surg, 2010. **45**(3): p. 610-8.
7. Abdul-Mumin, A., et al., *Burden of Neonatal Surgical Conditions in Northern Ghana.* World J Surg, 2019.
8. Bradnock, T.J., et al., *Gastroschisis: one year outcomes from national cohort study.* BMJ, 2011. **343**: p. d6749.
9. Badrinath, R., et al., *Outcomes and unmet need for neonatal surgery in a resource-limited environment: estimates of global health disparities from Kampala, Uganda.* J Pediatr Surg, 2014. **49**(12): p. 1825-30.
10. Wright, N.J., A. Zani, and N. Ade-Ajayi, *Epidemiology, management and outcome of gastroschisis in Sub-Saharan Africa: Results of an international survey.* Afr J Paediatr Surg, 2015. **12**(1): p. 1-6.
11. Liberati, A., et al., *The PRISMA statement for reporting systematic reviews and meta-analyses of studies that evaluate health care interventions: explanation and elaboration.* J Clin Epidemiol, 2009. **62**(10): p. e1-34.
12. Moher, D., et al., *Preferred reporting items for systematic reviews and meta-analyses: the PRISMA Statement.* Open Med, 2009. **3**(3): p. e123-30.
13. Goley, S., et al., *Investigating the use of ultrasonography for the antenatal diagnosis of structural congenital anomalies in low-income and middle-income countries: systematic review protocol* BMJ Paediatrics Open, 2019. **3**(1).
14. Santos, M.M., et al., *Infant hydrocephalus in sub-Saharan Africa: the reality on the Tanzanian side of the lake.* J Neurosurg Pediatr, 2017. **20**(5): p. 423-431.

Confidential: For Review Only

15. Munjanja, S.P., et al., *Audit of ultrasound scanning: antenatal diagnosis of congenital abnormalities in Harare, Zimbabwe.* East African Medical Journal, 1987. **64**(9): p. 601-5.
16. Sekabira, J. and G.P. Hadley, *Gastroschisis: a third world perspective.* Pediatric Surgery International, 2009. **25**(4): p. 327-9.
17. Wesonga, A.S., et al., *Gastroschisis in Uganda: Opportunities for improved survival.* Journal of Pediatric Surgery, 2016. **51**(11): p. 1772-1777.
18. de Paul Djientcheu, V., et al., *Management of neural tube defects in a Sub-Saharan African country: the situation in Yaounde, Cameroon.* Journal of the Neurological Sciences, 2008. **275**(1-2): p. 29-32.
19. Idowu, O.E. and O.S. Olawehinmi, *Surgical congenital central nervous system anomalies in a tropical teaching hospital.* British Journal of Neurosurgery, 2012. **26**(5): p. 726-9.
20. Okafor, H.U., S.O. Ekenze, and S.N. Uwaezuoke, *Posterior urethral valves: determinants of outcome in a developing country.* J Paediatr Child Health, 2013. **49**(2): p. 115-9.
21. Abdur-Rahman, L.O., N.A. Abdulrasheed, and J.O. Adeniran, *Challenges and outcomes of management of anterior abdominal wall defects in a Nigerian tertiary hospital.* Afr J Paediatr Surg, 2011. **8**(2): p. 159-63.
22. Adeleye, A.O., M.D. Dairo, and K.G. Olowookere, *Central nervous system congenital malformations in a developing country: issues and challenges against their prevention.* Childs Nerv Syst, 2010. **26**(7): p. 919-24.
23. Bankole, O.B., S.O. Arigbabu, and O.O. Kanu, *Spinal neural tube defects in Lagos University Teaching Hospital, Nigeria.* Nigerian Quarterly Journal of Hospital Medicine, 2012. **22**(1): p. 22-4.
24. Kitisomprayoonkul, N. and T. Tongsong, *Neural tube defects: a different pattern in northern Thai population.* Journal of the Medical Association of Thailand, 2001. **84**(4): p. 483-8.
25. Hong, L., et al., *Modified silo technique-An easy and effective method to improve the survival rate of neonates with gastroschisis in Shanghai.* European Journal of Obstetrics, Gynecology, & Reproductive Biology, 2010. **148**(1): p. 31-4.
26. Weng, R., et al., *Prenatal diagnosis and prognosis assessment of congenital choledochal cyst in 21 cases.* J Obstet Gynaecol, 2016. **36**(3): p. 324-7.
27. Luiza, A., et al., *A descriptive epidemiology study of oral cleft in sergipe, Brazil.* Int Arch Otorhinolaryngol, 2013. **17**(4): p. 390-4.
28. Tannuri, A.C., et al., *Evolution of critically ill patients with gastroschisis from three tertiary centers.* Clinics (Sao Paulo, Brazil), 2011. **66**(1): p. 17-20.
29. Kumar, B., L.D. Agrawal, and S.B. Sharma, *Congenital bronchopulmonary malformations: A single-center experience and a review of literature.* Annals of Thoracic Medicine, 2008. **3**(4): p. 135-139.
30. Vijayaraghavan, S.B., A.S. Prema, and P. Suganyadevi, *Sonographic Depiction of the Fetal Anus and Its Utility in the Diagnosis of Anorectal Malformations.* Journal of Ultrasound in Medicine, 2011. **30**(1): p. 37-45.
31. Tarca, E., I. Ciongradi, and S.G. Aprodu, *Birth Weight, Compromised Bowel and Sepsis are the Main Variables Significantly Influencing Outcome in Gastroschisis.* Chirurgia (Bucuresti), 2015. **110**(2): p. 151-6.

Confidential: For Review Only

32. Jin, L., et al., *Prevalence of Neural Tube Defects and the Impact of Prenatal Diagnosis in Three Districts of Beijing, China.* Paediatric and Perinatal Epidemiology, 2017. **31**(4): p. 293-300.
33. Sood, M., et al., *Neural tubal defects in an east Delhi hospital.* Indian Journal of Pediatrics, 1991. **58**(3): p. 363-5.
34. Ho, J.J., M.K. Thong, and N.K. Nurani, *Prenatal detection of birth defects in a Malaysian population: estimation of the influence of termination of pregnancy on birth prevalence in a developing country.* Australian & New Zealand Journal of Obstetrics & Gynaecology, 2006. **46**(1): p. 55-7.
35. Kazmi, S.S., et al., *The prenatal ultrasonographic detection of myelomeningocele in patients referred to Children's Hospital Medical Center: a cross sectional study.* Reprod Health, 2006. **3**: p. 6.
36. Oztekin, D., et al., *Embryonic heart rate as a prognostic factor for chromosomal abnormalities.* Journal of Ultrasound in Medicine, 2009. **28**(5): p. 609-14.
37. Kutuk, M.S., et al., *Prenatal diagnosis of upper extremity malformations with ultrasonography: Diagnostic features and perinatal outcome.* J Clin Ultrasound, 2017. **45**(5): p. 267-276.
38. Iliescu, D., et al., *Improved detection rate of structural abnormalities in the first trimester using an extended examination protocol.* Ultrasound Obstet Gynecol, 2013. **42**(3): p. 300-9.
39. Johnson, N., et al., *Congenital abnormalities at a tertiary center in Jamaica: an 18-month maternal-fetal medicine experience.* Journal of Maternal-Fetal & Neonatal Medicine, 2012. **25**(6): p. 687-91.
40. Liao, Y.M., et al., *Routine screening for fetal limb abnormalities in the first trimester.* Prenatal Diagnosis, 2016. **36**(2): p. 117-26.
41. Akinmoladun, J.A., et al., *Routine prenatal ultrasound anomaly screening program in a Nigerian university hospital: Redefining obstetrics practice in a developing African country.* Nigerian Medical Journal, 2015. **56**(4): p. 263-7.
42. Carrera, J.M., *Obstetric Ultrasounds in Africa: Is it Necessary to Promote their Appropriate Use?* Donald School Journal of Ultrasound in Obstetrics and Gynecology, 2011. **5**(3): p. 289-296.
43. Vilela, P.C., et al., *Risk factors for adverse outcome of newborns with gastroschisis in a Brazilian hospital.* Journal of Pediatric Surgery, 2001. **36**(4): p. 559-64.
44. Bhat, Y.R., V. Kumar, and A. Rao, *Congenital diaphragmatic hernia in a developing country.* Singapore Med J, 2008. **49**(9): p. 715-8.
45. Kumar, M., et al., *Prenatal sonographic evaluation and postnatal outcome of renal anomalies.* Indian J Hum Genet, 2012. **18**(1): p. 75-82.
46. Lu, Q.B., et al., *Investigation of ultrasound screening efficiency for neural tube defects during pregnancy in rural areas of China.* Public Health, 2011. **125**(9): p. 639-44.
47. Pelizzari, E., et al., *Characteristics of fetuses evaluated due to suspected anencephaly: a population-based cohort study in southern Brazil.* Sao Paulo Medical Journal = Revista Paulista de Medicina, 2015. **133**(2): p. 101-8.
48. Sanghvi, K.P., et al., *Antenatal diagnosis of congenital renal malformations using ultrasound.* J Trop Pediatr, 1998. **44**(4): p. 235-40.
49. Brizot, M.L., et al., *Conjoined twins pregnancies: experience with 36 cases from a single center.* Prenat Diagn, 2011. **31**(12): p. 1120-5.

Confidential: For Review Only

50. WHO. *WHO recommendation on early ultrasound in pregnancy*. The WHO Reproductive Health Library 2016; Available from: https://extranet.who.int/rhl/topics/preconception-pregnancy-childbirth-and-postpartum-care/antenatal-care/who-recommendation-early-ultrasound-pregnancy.
51. WHO, *WHO Study Group on Training in Diagnostic Ultrasound: Essentials, Principles, and Standards*, WHO, Editor. 1998, World Health Organization: Philadelphia, Pennsylvania.
52. Shah, S., et al., *Perceived barriers in the use of ultrasound in developing countries.* Crit Ultrasound J, 2015. **7**(1): p. 28.
53. LaGrone, L.N., et al., *A review of training opportunities for ultrasonography in low and middle income countries.* Trop Med Int Health, 2012. **17**(7): p. 808-19.
54. UN. *Goal 3: Ensure healthy lives and promote well-being for all at all ages*. Sustainable Development Goals 2015; Available from: https://www.un.org/sustainabledevelopment/health/.
55. WHO, *Sixty-Third World Health Assemby Report on Birth Defects*. 2010, World Health Organization: Geneva, Switzerland.

Confidential: For Review Only

Investigating the Use of Ultrasonography for the Antenatal Diagnosis of Structural Congenital Anomalies in Low- and Middle-Income Countries: A Systematic Review

Figure 1: Flowchart of the Screening Process

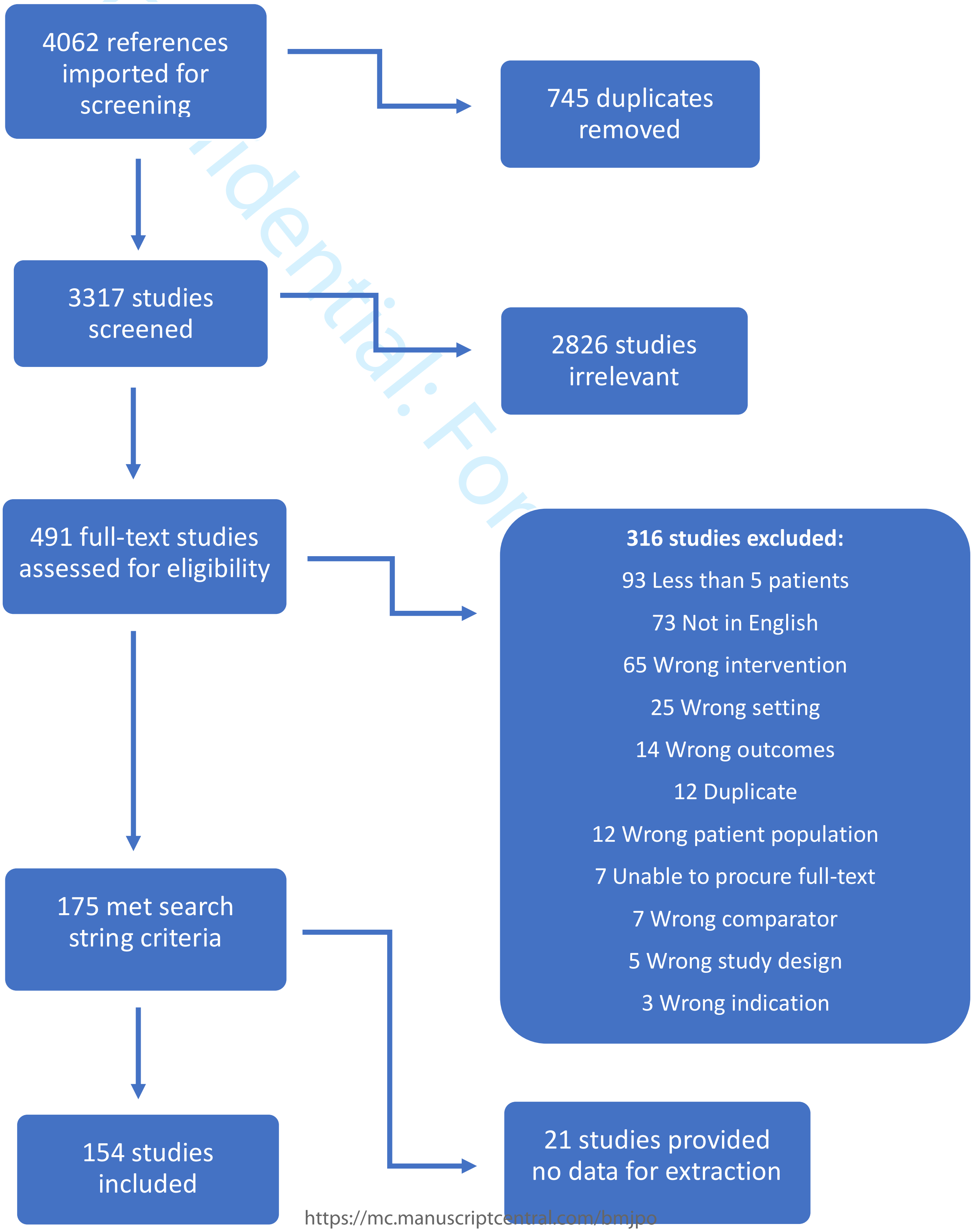

Investigating the Use of Ultrasonography for the Antenatal Diagnosis of Structural Congenital Anomalies in Low- and Middle-Income Countries: A Systematic Review

Figure 2: Regional Depiction of Articles included in the Systematic Review

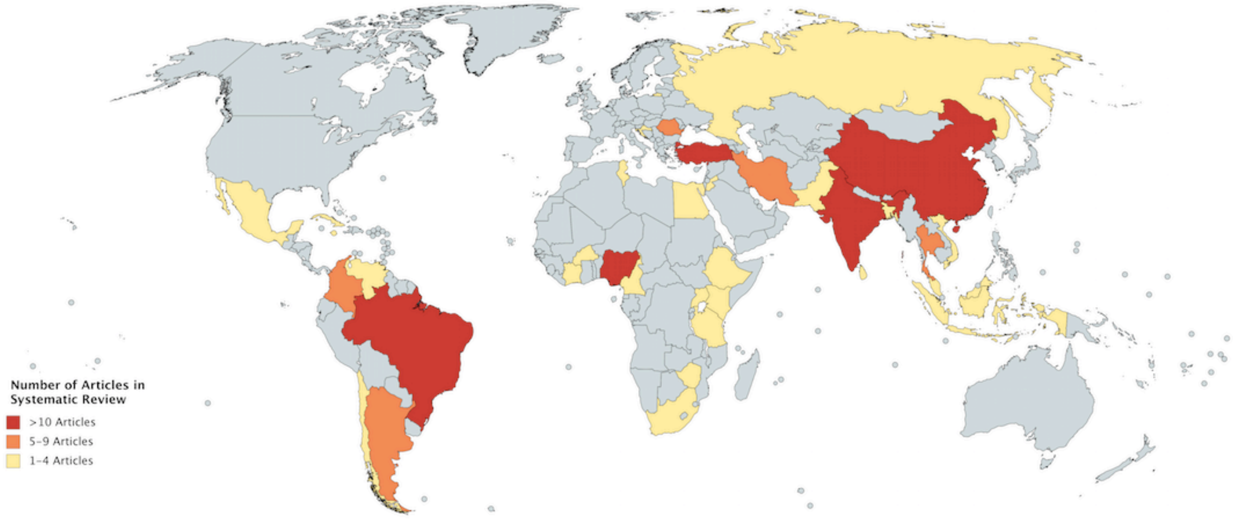

For Review Only

Supplementary File 1

Investigating the Use of Ultrasonography for the Antenatal Diagnosis of Structural Congenital Anomalies in Low- and Middle-Income Countries: A Systematic Review

PRISMA (Preferred Reporting Items for Systematic Reviews and Meta-Analyses) 2009 Checklist

Confidential: For Review Only

Section/topic	#	Checklist item	Section Heading
**TITLE**			
Title	1	Identify the report as a systematic review, meta-analysis, or both.	Title
**ABSTRACT**			
Structured summary	2	Provide a structured summary including, as applicable: background; objectives; data sources; study eligibility criteria, participants, and interventions; study appraisal and synthesis methods; results; limitations; conclusions and implications of key findings; systematic review registration number.	Abstract
**INTRODUCTION**			
Rationale	3	Describe the rationale for the review in the context of what is already known.	Introduction
Objectives	4	Provide an explicit statement of questions being addressed with reference to participants, interventions, comparisons, outcomes, and study design (PICOS).	Introduction
**METHODS**			
Protocol and registration	5	Indicate if a review protocol exists, if and where it can be accessed (e.g., Web address), and, if available, provide registration information including registration number.	Methodology
Eligibility criteria	6	Specify study characteristics (e.g., PICOS, length of follow-up) and report characteristics (e.g., years considered, language, publication status) used as criteria for eligibility, giving rationale.	Methodology
Information sources	7	Describe all information sources (e.g., databases with dates of coverage, contact with study authors to identify additional studies) in the search and date last searched.	Methodology
Search	8	Present full electronic search strategy for at least one database, including any limits used, such that it could be repeated.	Methodology
Study selection	9	State the process for selecting studies (i.e., screening, eligibility, included in systematic review, and, if applicable, included in the meta-analysis).	Methodology
Data collection process	10	Describe method of data extraction from reports (e.g., piloted forms, independently, in duplicate) and any processes for obtaining and confirming data from investigators.	Methodology
Data items	11	List and define all variables for which data were sought (e.g., PICOS, funding sources) and any assumptions and simplifications made.	Methodology

Risk of bias in individual studies	12	Describe methods used for assessing risk of bias of individual studies (including specification of whether this was done at the study or outcome level), and how this information is to be used in any data synthesis.	Methodology
Summary measures	13	State the principal summary measures (e.g., risk ratio, difference in means).	Methodology
Synthesis of results	14	Describe the methods of handling data and combining results of studies, if done, including measures of consistency (e.g., I^2) for each meta-analysis.	Methodology

From: Moher D, Liberati A, Tetzlaff J, Altman DG, The PRISMA Group (2009). Preferred Reporting Items for Systematic Reviews and Meta-Analyses: The PRISMA Statement. PLoS Med 6(6): e1000097. doi:10.1371/journal.pmed1000097

Confidential: For Review Only

Supplementary File 2

Investigating the Use of Ultrasonography for the Antenatal Diagnosis of Structural Congenital Anomalies in Low- and Middle-Income Countries: A Systematic Review

Database: **Ovid MEDLINE(R) Epub Ahead of Print, In-Process & Other Non-Indexed Citations, Ovid MEDLINE(R) Daily, Ovid MEDLINE and Versions(R)** <1946 to Present>

Search Strategy

No.	Search Terms	Results
1	exp Congenital Abnormalities/ or congenital anomal*.mp.	559922
2	exp Congenital Abnormalities/ or Congenital Abnormal*.mp.	555311
3	Congenital Malformation*.mp.	13826
4	Fetal Malformation*.mp.	1917
5	Birth Defect*.mp.	9122
6	Anencephaly.mp. or exp ANENCEPHALY/	3875
7	Conjoined Twins.mp. or exp Twins, Conjoined/	2022
8	exp Heart Defects, Congenital/ or Congenital Heart Defect*.mp.	142854
9	Anorectal Malformation*.mp.	1896
10	Anal Stenosis.mp.	389
11	Anal Atresia.mp. or exp Anus, Imperforate/	2815
12	Imperforate Anus.mp. or exp Anus, Imperforate/	2992
13	Biliary atresia.mp. or exp Biliary Atresia/	4482
14	exp Choledochal Cyst/ or Choledochal Cyst*.mp.	2825
15	exp Diaphragmatic Eventration/ or Diaphragmatic Eventration*.mp.	943
16	Esophageal Atresia.mp. or exp Esophageal Atresia/	4173
17	exp Tracheoesophageal Fistula/ or Tracheo-?esophageal fistula*.mp.	3676
18	Intestinal Atresia.mp. or exp Intestinal Atresia/	2301
19	Duodenal atresia.mp.	839
20	Colonic Atresia.mp.	152
21	Malrotation.mp.	2393
22	Apple peel syndrome.mp.	16
23	exp Hernias, Diaphragmatic, Congenital/ or congenital diaphragmatic hernia*.mp.	5530
24	Gastroschisis.mp. or exp GASTROSCHISIS/	2313
25	Abdominal Wall Defect*.mp.	2009
26	Exomphalos.mp. or exp Hernia, Umbilical/	3740
27	Omphalocele.mp. or exp Hernia, Umbilical/	4242
28	Congenital Limb Deformities.mp. or exp Limb Deformities, Congenital/	22483
29	exp Neural Tube Defects/ or Neural Tube Defect*.mp.	29051
30	exp Bronchogenic Cyst/ or Bronchogenic Cyst*.mp.	1810
31	exp Bronchopulmonary Sequestration/ or Bronchopulmonary sequestration*.mp.	2022

Confidential: For Review Only

32	exp "Cystic Adenomatoid Malformation of Lung, Congenital"/ or Congenital Cystic adenomatoid malformation of lung*.mp.	874
33	Renal Anomal*.mp.	1185
34	exp Urogenital Abnormalities/ or Genito-urinary Anomalies.mp.	57735
35	exp Maxillofacial Abnormalities/ or Maxillofacial Abnormalit*.mp.	28265
36	exp Mouth Abnormalities/ or Mouth Abnormalit*.mp.	24459
37	Umbilical Hernia.mp. or exp Hernia, Umbilical/	4200
38	Hirschsprung disease.mp. or exp Hirschsprung Disease/	4567
39	exp Megacolon/ or Ganglionic Megacolon.mp.	7053
40	Rectosigmoid aganglionosis.mp.	15
41	Colonic Aganglionosis.mp.	311
42	Intestinal Aganglionosis.mp.	190
43	Volvulus.mp. or exp Intestinal Volvulus/	8927
44	LMICs.mp.	2001
45	(low- and middle-income countr*).mp. [mp=title, abstract, original title, name of substance word, subject heading word, floating sub-heading word, keyword heading word, protocol supplementary concept word, rare disease supplementary concept word, unique identifier, synonyms]	10910
46	Low-resource setting*.mp.	2719
47	Underdeveloped Countries.mp.	817
48	low-income countries.mp.	4707
49	exp Developing Countries/ or Developing countr*.mp.	115302
50	middle-income countries.mp.	11136
51	limited resource setting*.mp.	253
52	Africa South of the Sahara.mp. or exp "Africa South of the Sahara"/	184102
53	Sub-Saharan Africa.mp. or exp "Africa South of the Sahara"/	190396
54	Less Resourced communities.mp.	1
55	Afghanistan.mp. or exp AFGHANISTAN/	6065
56	Albania.mp. or exp ALBANIA/	1182
57	Algeria.mp. or exp ALGERIA/	3621
58	American Samoa.mp.	354
59	Angola.mp. or exp ANGOLA/	1403
60	Argentina.mp. or exp ARGENTINA/	19610
61	Armenia.mp. or exp ARMENIA/	1676
62	Azerbaijan.mp. or exp AZERBAIJAN/	1765
63	Bangladesh.mp. or exp BANGLADESH/	12907
64	Belarus.mp. or exp "Republic of Belarus"/	2553
65	Belize.mp. or exp BELIZE/	868
66	Benin.mp. or exp BENIN/	3441
67	exp BHUTAN/ or Bhutan.mp.	628
68	Bolivia.mp. or exp BOLIVIA/	3594
69	(Bosnia and Herzegovina).mp. [mp=title, abstract, original title, name of substance word, subject heading word, floating sub-heading word, keyword heading word, protocol supplementary concept word, rare disease supplementary concept word, unique identifier, synonyms]	2477

Confidential: For Review Only

70	Botswana.mp. or exp BOTSWANA/	2247
71	Brazil.mp. or exp BRAZIL/	101919
72	Bulgaria.mp. or exp BULGARIA/	7554
73	Burkina Faso.mp. or exp Burkina Faso/	3904
74	Burundi.mp. or exp BURUNDI/	871
75	Cabo Verde.mp. or exp Cabo Verde/	211
76	Cambodia.mp. or exp CAMBODIA/	4102
77	Cameroon.mp. or exp CAMEROON/	6804
78	Central African Republic.mp. or exp Central African Republic/	1096
79	Chad.mp. or exp CHAD/	1144
80	China.mp. or exp CHINA/	205799
81	Colombia.mp. or exp COLOMBIA/	12533
82	Comoros.mp. or exp COMOROS/	427
83	Democratic Republic of the Congo.mp. or exp "Democratic Republic of the Congo"/	4506
84	exp "Democratic Republic of the Congo"/ or DRC.mp.	4827
85	Republic of the Congo.mp. or exp Congo/	6176
86	Costa Rica.mp. or exp Costa Rica/	5088
87	Cote d'Ivoire.mp. or exp Cote d'Ivoire/	3527
88	Ivory Coast.mp. or exp Cote d'Ivoire/	3432
89	Croatia.mp. or exp CROATIA/	8261
90	Cuba.mp. or exp CUBA/	6343
91	Djibouti.mp. or exp DJIBOUTI/	372
92	Dominica.mp. or exp DOMINICA/	430
93	Dominican Republic.mp. or exp Dominican Republic/	2128
94	Ecuador.mp. or exp ECUADOR/	4722
95	exp EGYPT/ or Egypt.mp.	18702
96	El Salvador.mp. or exp El Salvador/	1319
97	Equatorial Guinea.mp. or exp Equatorial Guinea/	419
98	Eritrea.mp. or exp ERITREA/	518
99	Ethiopia.mp. or exp ETHIOPIA/	13354
100	Fiji.mp. or exp FIJI/	1620
101	Gabon.mp. or exp GABON/	1925
102	exp GAMBIA/ or Gambia.mp.	2958
103	exp "GEORGIA (REPUBLIC)"/ or exp GEORGIA/ or Georgia.mp.	15842
104	Ghana.mp. or exp GHANA/	9432
105	Grenada.mp. or exp GRENADA/	308
106	Guatemala.mp. or exp GUATEMALA/	3907
107	Guinea.mp. or exp GUINEA-BISSAU/ or exp EQUATORIAL GUINEA/ or exp GUINEA/ or exp PAPUA NEW GUINEA/ or exp NEW GUINEA/	160074
108	Guyana.mp. or exp GUYANA/	1136
109	Haiti.mp. or exp HAITI/	3707
110	Honduras.mp. or exp HONDURAS/	1736
111	India.mp. or exp INDIA/	129215
112	Indonesia.mp. or exp INDONESIA/	13107

Confidential: For Review Only

113	exp Iran/ or Islamic Republic of Iran.mp.	21280
114	exp IRAQ/ or Iraq.mp.	9035
115	Jamaica.mp. or exp JAMAICA/	4427
116	Jordan.mp. or exp JORDAN/	6040
117	Kazakhstan.mp. or exp KAZAKHSTAN/	3153
118	Kenya.mp. or exp KENYA/	18798
119	Kiribati.mp. or exp Micronesia/	1934
120	Democratic People's Republic of Korea.mp. or exp "Democratic People's Republic of Korea"/	246
121	Kosovo.mp. or exp KOSOVO/	840
122	Kyrgyz Republic.mp. or exp Kyrgyzstan/	1225
123	exp Laos/ or Lao PDR.mp.	1876
124	Laos.mp. or exp LAOS/	2431
125	Lebanon.mp. or exp LEBANON/	5011
126	Lesotho.mp. or exp LESOTHO/	645
127	Liberia.mp. or exp LIBERIA/	1667
128	Libya.mp. or exp LIBYA/	1554
129	Macedonia Republic.mp. or exp "Macedonia (Republic)"/	475
130	Madagascar.mp. or exp MADAGASCAR/	4734
131	Malawi.mp. or exp MALAWI/	6317
132	Malaysia.mp. or exp MALAYSIA/	18390
133	Maldives.mp. or exp Indian Ocean Islands/	10595
134	Mali.mp. or exp MALI/	3511
135	Marshall Islands.mp. or exp Micronesia/	1959
136	Mauritania.mp. or exp MAURITANIA/	626
137	Mauritius.mp. or exp MAURITIUS/	953
138	exp MEXICO/ or Mexico.mp.	53282
139	Micronesia.mp. or exp MICRONESIA/	2243
140	Moldova.mp. or exp MOLDOVA/	920
141	Mongolia.mp. or exp MONGOLIA/	3809
142	Montenegro.mp. or exp MONTENEGRO/	749
143	Morocco.mp. or exp MOROCCO/	6855
144	Mozambique.mp. or exp MOZAMBIQUE/	3236
145	Myanmar.mp. or exp MYANMAR/	3198
146	Namibia.mp. or exp NAMIBIA	1502
147	Nauru.mp. or exp Micronesia/	1921
148	Nepal.mp. or exp NEPAL/	9337
149	Nicaragua.mp. or exp NICARAGUA/	1949
150	exp NIGER/ or Niger.mp.	12299
151	Nigeria.mp. or exp NIGERIA/	32627
152	Pakistan.mp. or exp PAKISTAN/	20153
153	exp PANAMA/ or Panama.mp.	4265
154	Papua New Guinea.mp. or exp Papua New Guinea/	5003
155	Paraguay.mp. or exp PARAGUAY/	1433
156	Peru.mp. or exp PERU/	11249

Confidential: For Review Only

157	Philippines.mp. or exp PHILIPPINES/	10698
158	Romania.mp. or exp ROMANIA/	11521
159	exp Russia/ or Russian Federation.mp.	51376
160	Rwanda.mp. or exp RWANDA/	2883
161	exp AMERICAN SAMOA/ or Samoa.mp. or exp SAMOA/ or exp "INDEPENDENT STATE OF SAMOA"/	1024
162	(Sao Tome and Principe).mp. [mp=title, abstract, original title, name of substance word, subject heading word, floating sub-heading word, keyword heading word, protocol supplementary concept word, rare disease supplementary concept word, unique identifier, synonyms]	135
163	Senegal.mp. or exp SENEGAL/	7067
164	Serbia.mp. or exp SERBIA/	4574
165	Sierra Leone.mp. or exp Sierra Leone/	2103
166	Solomon Islands.mp. or exp Melanesia/	6193
167	Somalia.mp. or exp SOMALIA/	1968
168	Somaliland.mp.	136
169	South Africa.mp. or exp South Africa/	47075
170	South Sudan.mp. or exp South Sudan/	397
171	Sri Lanka.mp. or exp Sri Lanka/	7342
172	Saint Lucia.mp. or exp Saint Lucia/	114
173	(Saint Vincent and the Grenadines).mp. [mp=title, abstract, original title, name of substance word, subject heading word, floating sub-heading word, keyword heading word, protocol supplementary concept word, rare disease supplementary concept word, unique identifier, synonyms]	57
174	exp SUDAN/ or Sudan.mp.	8429
175	Suriname.mp. or exp SURINAME/	1098
176	Swaziland.mp. or exp SWAZILAND/	818
177	Syrian Arab Republic.mp.	73
178	Syria.mp. or exp SYRIA/	2407
179	Tajikistan.mp. or exp TAJIKISTAN/	910
180	Tanzania.mp. or exp TANZANIA/	12921
181	Thailand.mp. or exp THAILAND/	32186
182	exp Timor-Leste/ or Timor-Leste.mp.	311
183	East Timor.mp.	187
184	Togo.mp. or exp TOGO/	1491
185	Tonga.mp. or exp TONGA/	463
186	Tunisia.mp. or exp TUNISIA/	9057
187	exp TURKEY/ or Turkey.mp.	46898
188	Turkmenistan.mp. or exp TURKMENISTAN/	717
189	Tuvalu.mp. or exp Micronesia/	1884
190	Uganda.mp. or exp UGANDA/	13956
191	Ukraine.mp. or exp UKRAINE/	16702
192	Uzbekistan.mp. or exp UZBEKISTAN/	2161
193	Vanuatu.mp. or exp VANUATU/	629
194	Venezuela.mp. or exp VENEZUELA/	6620

Confidential: For Review Only

195	exp VIETNAM/ or Vietnam.mp.	15919
196	(West Bank and Gaza).mp. [mp=title, abstract, original title, name of substance word, subject heading word, floating sub-heading word, keyword heading word, protocol supplementary concept word, rare disease supplementary concept word, unique identifier, synonyms]	194
197	Republic of Yemen.mp. or exp Yemen/	1264
198	Zambia.mp. or exp ZAMBIA/	5404
199	Zimbabwe.mp. or exp ZIMBABWE/	6879
200	Antenatal Diagnosis.mp.	2383
201	Prenatal Diagnosis.mp. or exp Prenatal Diagnosis/	74636
202	Antenatal Screening.mp.	1193
203	Prenatal Screening.mp.	2579
204	Antenatal Ultrasound.mp.	702
205	Prenatal Ultrasound.mp.	2203
206	Antenatal Ultrasonography.mp.	200
207	Prenatal Ultrasonography.mp. or exp Ultrasonography, Prenatal/	30427
208	1 or 2 or 3 or 4 or 5 or 6 or 7 or 8 or 9 or 10 or 11 or 12 or 13 or 14 or 15 or 16 or 17 or 18 or 19 or 20 or 21 or 22 or 23 or 24 or 25 or 26 or 27 or 28 or 29 or 30 or 31 or 32 or 33 or 34 or 35 or 36 or 37 or 38 or 39 or 40 or 41 or 42 or 43	607001
209	44 or 45 or 46 or 47 or 48 or 49 or 50 or 51 or 52 or 53 or 54 or 55 or 56 or 57 or 58 or 59 or 60 or 61 or 62 or 63 or 64 or 65 or 66 or 67 or 68 or 69 or 70 or 71 or 72 or 73 or 74 or 75 or 76 or 77 or 78 or 79 or 80 or 81 or 82 or 83 or 84 or 85 or 86 or 87 or 88 or 89 or 90 or 91 or 92 or 93 or 94 or 95 or 96 or 97 or 98 or 99 or 100 or 101 or 102 or 103 or 104 or 105 or 106 or 107 or 108 or 109 or 110 or 111 or 112 or 113 or 114 or 115 or 116 or 117 or 118 or 119 or 120 or 121 or 122 or 123 or 124 or 125 or 126 or 127 or 128 or 129 or 130 or 131 or 132 or 133 or 134 or 135 or 136 or 137 or 138 or 139 or 140 or 141 or 142 or 143 or 144 or 145 or 146 or 147 or 148 or 149 or 150 or 151 or 152 or 153 or 154 or 155 or 156 or 157 or 158 or 159 or 160 or 161 or 162 or 163 or 164 or 165 or 166 or 167 or 168 or 169 or 170 or 171 or 172 or 173 or 174 or 175 or 176 or 177 or 178 or 179 or 180 or 181 or 182 or 183 or 184 or 185 or 186 or 187 or 188 or 189 or 190 or 191 or 192 or 193 or 194 or 195 or 196 or 197 or 198 or 199 (1385344)	1385344
210	200 or 201 or 202 or 203 or 204 or 205 or 206 or 207	77525
211	208 and 209 and 210	896

Confidential: For Review Only

Supplementary File 3

Investigating the Use of Ultrasonography for the Antenatal Diagnosis of Structural Congenital Anomalies in Low- and Middle-Income Countries: A Systematic Review

Data Extraction Template

**Reference**	Reference (Full)
	Title
	Author(s)
	Publication Date
	Year of Publication
	Journal
**Place of Study**	Hospital
	City
	Country
	Continent
**Study Type**	Type of Study (RCT, Cohort, Case-Control, etc.)
**Publication Status**	Has Study Been Published?
**Study Population**	Number of Patients
	Patient Cohort (Institutional, Multi-Center, Regional, National, etc.)
	Patient Cohort (All Patients Presenting with a Condition vs. Those with an Antenatal Diagnosis)
	Gestational Age (Mean, Median)
	Type of Anomaly
**Diagnosis**	Type of Ultrasound (Abdominal vs. Transvaginal; 2D vs. 3D)
	Percent Receiving Antenatal Ultrasound
	Percent with Any Anomaly Detected
	Percent with Accurate Antenatal Diagnosis
	Incidence of Anomaly
	Training of Ultrasonographer
	Referral Rate for Further Antenatal Care
	Referral Rate to Tertiary Pediatric Surgery Center
	Percent Born in a Pediatric Surgery Center
**Outcomes**	Mortality Rate in Live Births
	Termination Rate
	Complications (Using Clavien-Dindo Classification)
	Additional Notes Regarding the Effect of Antenatal Diagnosis on Outcomes
	Other Benefits of Antenatal Diagnosis (i.e., Parental Counselling, Option for Termination, etc.)
	Comments Regarding Policy
	Additional Notes/Other Useful Information

Confidential: For Review Only

Supplementary File 4

Investigating the Use of Ultrasonography for the Antenatal Diagnosis of Structural Congenital Anomalies in Low- and Middle-Income Countries: A Systematic Review

References of the Articles Included in the Systematic Review

ABDUR-RAHMAN, L. O., ABDULRASHEED, N. A. & ADENIRAN, J. O. 2011. Challenges and outcomes of management of anterior abdominal wall defects in a Nigerian tertiary hospital. Afr J Paediatr Surg, 8, 159-63.

ACHARYA, P. M., J.; MALHOTRA, N.; SURESH, S.; KHURANA, A.; LULLA, C.; ACHARYA, H.; BORA, N. M.; BORA, R.; MALHOTRA, K. 2013. Screening for birth defects strategies for developing low resource countries. Donald School Journal of Ultrasound in Obstetrics and Gynecology, 7, 453-461.

ADELEYE, A. O. & JOEL-MEDEWASE, V. I. 2015. Awareness and uptake of measures for preventing CNS birth defects among mothers of affected children in a sub-Saharan African neurosurgeon's practice. Childs Nerv Syst, 31, 2311-7.

ADELEYE, A. O., DAIRO, M. D. & OLOWOOKERE, K. G. 2010. Central nervous system congenital malformations in a developing country: Issues and challenges against their prevention. Child's Nervous System, 26, 919-924.

AGARWAL, S., KADAGAD, P., PINTO, P. & BALIGA, S. 2013. Attitude of mothers of children with orofacial cleft towards antenatal diagnosis of non syndromic orofacial clefts in India. International Journal of Oral and Maxillofacial Surgery, 42 (10), 1193.

AKGUN, H., BASBUG, M., OZGUN, M. T., OZTURK, F. & OKTEN, T. 2014. Correlation between antenatal ultrasound and fetal autopsy findings on urinary system anomalies terminated in the second trimester. Prenat Diagn, 34, 285-90.

AKHTAR, M., QAISER, Q. A. & LATIF, A. 2016. Antenatal Diagnosis of Anencephaly to Determine the Prevalence and its Correlation with Folic Acid Deficiency by Using Peripheral Blood Picture in District Poonch, Azad Kashmir. Pakistan Journal of Medical and Health Sciences, 10, 628-630.

AKHTAR, N. & KARIM, S. 2012. Varieties of foetal congenital abnormality. Mymensingh Med J, 21, 662-7.

AKINMOLADUN, J. A., OGBOLE, G. I. & TA, O. O. 2018. Pattern and outcome of antenatally diagnosed major congenital anomalies at a Nigerian Tertiary Hospital. Nigerian Journal of Clinical Practice, 21, 560-565.

AKINMOLADUN, J. A., OGBOLE, G. I., LAWAL, T. A. & ADESINA, O. A. 2015. Routine antenatal ultrasound anomaly screening program in a Nigerian university hospital: Redefining obstetrics practice in a developing African country. Nigerian Medical Journal, 56, 263-7.

AL-MENDALAWI, M. D. 2014. Towards a national program to combat neural tube defects in Iraq. Congenit Anom (Kyoto), 54, 123.

AMADI, C. E. & EGHWRUDJAKPOR, P. O. 2013. The pattern of distribution of encephalocele in University of Port Harcourt Teaching Hospital--a three year experience. Niger J Med, 22, 19-23.

AQRABAWI, H. E. 2005. Incidence of neural tube defects among neonates at King Hussein Medical Centre, Jordan. Eastern Mediterranean Health Journal, 11, 819-23.

ARAWI, T. & NASSAR, A. 2011. Antenatally diagnosed foetal malformations and termination of pregnancy: the case of Lebanon. Developing World Bioethics, 11, 40-7.

ASLAN, H., YILDIRIM, G., ONGUT, C. & CEYLAN, Y. 2007. Termination of pregnancy for fetal anomaly. International Journal of Gynaecology & Obstetrics, 99, 221-4.
AYGUN, C., KURUCU, S., CAKMAK-CELIK, F., DAGCINAR, A., TANYERI, B. & KUCUKODUK, S. 2013. Experience of a tertiary care center on 100 newborns with neural tube defects. Turk J Pediatr, 55, 359-64.
BABU, R. S. & PASULA, S. 2013. Frequency of foetal anomalies in a tertiary care centre. J Clin Diagn Res, 7, 1276-9.
BANKOLE, O. B., ARIGBABU, S. O. & KANU, O. O. 2012. Spinal neural tube defects in Lagos University Teaching Hospital, Nigeria. Nigerian Quarterly Journal of Hospital Medicine, 22, 22-4.
BENUTE, G. R., NOMURA, R. M., LIAO, A. W., BRIZOT MDE, L., DE LUCIA, M. C. & ZUGAIB, M. 2012. Feelings of women regarding end-of-life decision making after ultrasound diagnosis of a lethal fetal malformation. Midwifery, 28, 472-5.
BHAT, Y. R., KUMAR, V. & RAO, A. 2008. Congenital diaphragmatic hernia in a developing country. Singapore Med J, 49, 715-8.
BRIZOT, M. L., LIAO, A. W., LOPES, L. M., OKUMURA, M., MARQUES, M. S., KREBS, V., SCHULTZ, R. & ZUGAIB, M. 2011. Conjoined twins pregnancies: experience with 36 cases from a single center. Prenat Diagn, 31, 1120-5.
CAMPANA, H., ERMINI, M., AIELLO, H. A., KRUPITZKI, H., CASTILLA, E. E. & LOPEZ-CAMELO, J. S. 2010. Antenatal sonographic detection of birth defects in 18 hospitals from South America. Journal of ultrasound in medicine : official journal of the American Institute of Ultrasound in Medicine, 29, 203-212.
CARVALHO, F. H., CORDEIRO, K. M., PEIXOTO, A. B., TONNI, G., MORON, A. F., FEITOSA, F. E., FEITOSA, H. N. & ARAUJO JUNIOR, E. 2016. Associated ultrasonographic findings in fetuses with microcephaly because of suspected Zika virus (ZIKV) infection during pregnancy. Prenat Diagn, 36, 882-7.
CARVALHO, M. H., BRIZOT, M. L., LOPES, L. M., CHIBA, C. H., MIYADAHIRA, S. & ZUGAIB, M. 2002. Detection of fetal structural abnormalities at the 11-14 week ultrasound scan. Prenat Diagn, 22, 1-4.
CASANUEVA, E., LISKER, R., CARNEVALE, A. & ALONSO, E. 1997. Attitudes of Mexican physicians toward induced abortion. International Journal of Gynaecology & Obstetrics, 56, 47-52.
CEYLANER, S., CEYLANER, G., GUNYELI, I., EKICI, E., TUG, M., TANER, D., EKERBICER, H., MOLLAMAHMUTOGLU, L. & DANISMAN, N. 2006. Postmortem evaluation of 220 antenatally diagnosed fetuses with neural tube defects: detection of associated anomalies in a Turkish population. Antenatal Diagnosis, 26, 147-53.
CHANOUFI, M. B., BEN TEMIME, R., MASMOUDI, A., OUNAISSA, K., JEBNOUN, S., ABID, W., NSIRI, R., CHELLI, H., KHROUF, N. & SIALA-GAIGI, S. 2004. Clinical and anatomic features of acardiac twins. Medical Principles & Practice, 13, 375-9.
CHOOPA, M. S. M., S. O.; VAN BILJON, G. 2017. Diagnosis, management and outcome of posterior urethral valves in children at a tertiary centre in South Africa. Pediatric Nephrology, 32.
CHUN, H., GANG, Y., XIAOCHUN, Z., JIN, T., BO, X., LIMIN, W. & CUIFEN, L. 2015. Diagnosis and management of intradiaphragmatic extralobar pulmonary sequestration: a report of 11 cases. J Pediatr Surg, 50, 1269-72.
CORREA, C. M., C.; PENA, R.; RINCON, L. C.; GRACIA, G.; ZARANTE, I. 2014. Congenital malformations of pediatric surgical interest: prevalence, risk factors, and antenatal diagnosis

Confidential: For Review Only

between 2005 and 2012 in the capital city of a developing country. Bogota, Colombia. Journal of Pediatric Surgery, 49, 1099-1103.

DANE, B., DANE, C., SIVRI, D., KIRAY, M., CETIN, A. & YAYLA, M. 2007. Ultrasound screening for fetal major abnormalities at 11-14 weeks. Acta Obstet Gynecol Scand, 86, 666-70.

DAYYABU, A. L. M., D. L.; AVIDIME, A. R.; YUSUF, M. 2016. A review of five cases of fetal congenital anomalies diagnosed during antenatal ultrasound: Management problems and limitations. Donald School Journal of Ultrasound in Obstetrics and Gynecology 10, 136-142.

DE PAUL DJIENTCHEU, V., NJAMNSHI, A. K., WONKAM, A., NJIKI, J., GUEMSE, M., MBU, R., OBAMA, M. T., TAKONGMO, S., KAGO, I., TETANYE, E. & TIETCHE, F. 2008. Management of neural tube defects in a Sub-Saharan African country: the situation in Yaounde, Cameroon. Journal of the Neurological Sciences, 275, 29-32.

DE ROVETTO, C. R., URCUQUI, L. A., VALENCIA, M., DE CASTANO, I. & MARTINEZ, A. M. 2010. Unilateral renal agenesis: Case review of ambulatory pediatric nephrology clinics in Cali. Colombia Medica, 41, 52-59.

DEKA, D., MALHOTRA, N., TAKKAR, D., MITTAL, S., KRIPLANI, A. & ROY, K. K. 1999. Antenatal diagnosis and assessment of fetal malformations by ultrasonography in India. Indian Journal of Pediatrics, 66, 737-49.

DENG, K., QIU, J., DAI, L., YI, L., DENG, C., MU, Y. & ZHU, J. 2014. Perinatal mortality in pregnancies with omphalocele: data from the Chinese national birth defects monitoring network, 1996-2006. BMC Pediatrics, 14, 160.

GAMMELTOFT, T. T., M. H.; NGUYEN, T. H.; NGUYEN, T. T. 2008. Late-term abortion for fetal anomaly: Vietnamese women's experiences. Reproductive Health Matters 16, 46-56.

GEERTS, L. T., BRAND, E. J. & THERON, G. B. 1996. Routine obstetric ultrasound examinations in South Africa: cost and effect on perinatal outcome--a prospective randomised controlled trial. Br J Obstet Gynaecol, 103, 501-7.

GERMANI, M., LIBERTO, D., ELMO, G., LOBOS, P. & RUIZ, E. 2011. Choledochal cyst in pediatric patients: a 10-year single institution experience. Acta Gastroenterol Latinoam, 41, 302-7.

GHAVAMI, M. & ABEDINZADEH, R. 2011. Prevalence of perinatal central nervous system anomalies in East azarbaijan-iran. Iran J Radiol, 8, 79-81.

GODBOLE, K., BHIDE, V., NERUNE, S., KULKARNI, A., MOGHE, M. & KANADE, A. 2014. Role of fetal autopsy as a complementary tool to antenatal ultrasound. Journal of Maternal-Fetal & Neonatal Medicine, 27, 1688-92.

GROISMAN, B., GILI, J., GIMENEZ, L., POLETTA, F., BIDONDO, M. P., BARBERO, P., LIASCOVICH, R. & LOPEZ-CAMELO, J. 2017. Geographic clusters of congenital anomalies in Argentina. Journal of Community Genetics, 8, 1-7.

GROISMAN, B., LIASCOVICH, R., BARBERO, P., ALBERG, C., MOORTHIE, S., NACUL, L. & SAGOO, G. S. 2013. The use of a Toolkit for health needs assessment on neural tube defects in Argentina. Journal of Community Genetics, 4, 77-86.

GROVER, S., GARG, B., SOOD, N. & ARORA, K. 2017. Lethal Congenital Malformations in Fetuses-Antenatal Ultrasound or Perinatal Autopsy. Fetal & Pediatric Pathology, 36, 220-231.

GUNENDI, T., ERGINEL, B., BASTU, E., KALELIOGLU, I., HAS, R., SOYSAL, F. G., KESKIN, E., CELIK, A. & SALMAN, T. 2017. Is there a determining factor that predicts mortality in patients with congenital diaphragmatic hernia? Kardiochir Torakochirurgia Pol, 14, 149-153.

GUPTA, S. G. & SINGH, H. 2001. ANTENATAL DIAGNOSIS OF NEURAL TUBE DEFECTS. Med J Armed Forces India, 57, 126-8.

Confidential: For Review Only

GUSEVA, O. 2015. Evaluation of antenatal diadnosis of congenital heart disease and perinatal outcomes in a regional study. Journal of Perinatal Medicine. Conference: 12th World Congress of Perinatal Medicine 43.

HO, J. J., THONG, M. K. & NURANI, N. K. 2006. Antenatal detection of birth defects in a Malaysian population: estimation of the influence of termination of pregnancy on birth prevalence in a developing country. Australian & New Zealand Journal of Obstetrics & Gynaecology, 46, 55-7.

HONG, L., WU, Y. M., YAN, Z. L., CHEN, S. & WANG, J. 2010. Modified silo technique-An easy and effective method to improve the survival rate of neonates with gastroschisis in Shanghai. European Journal of Obstetrics, Gynecology, & Reproductive Biology, 148, 31-4.

HOSTIUC, S., BUDA, O. & HOSTIUC, M. 2013. Late abortion. Attitudes amongst young physicians in Romania. Archives of Gynecology and Obstetrics, 288, 431-437.

HSIEH, Y. Y., CHANG, F. C., TSAI, H. D., HSU, T. Y. & YANG, T. C. 2000. Accuracy of sonography in predicting the outcome of fetal congenital diaphragmatic hernia. Zhonghua Yi Xue Za Zhi (Taipei), 63, 751-7.

IDOWU, O. E. & OLAWEHINMI, O. S. 2012. Surgical congenital central nervous system anomalies in a tropical teaching hospital. British Journal of Neurosurgery, 26, 726-9.

ILIESCU, D. T., S.; COMANESCU, A.; ANTSAKLIS, P.; COTARCEA, S.; NOVAC, L.; CERNEA, N.; ANTSAKLIS, A. 2013. Improved detection rate of structural abnormalities in the first trimester using an extended examination protocol. Ultrasound in Obstetrics & Gynecology, 42, 300-309.

JARURATANASIRIKUL, S. K.-A., O.; LIMPITIKUL, W.; DISSANEEVATE, P.; KHUNNARAKPONG, N.; SATTAPANYO, A. 2014. Prevalence of neural tube defect in southern Thailand: a population-based survey during 2009-2012. Childs Nervous System, 30, 1269-1275.

JIN, L., JIN, L., YU, J., XU, Y., LIU, H. & REN, A. 2017. Prevalence of Neural Tube Defects and the Impact of Antenatal Diagnosis in Three Districts of Beijing, China. Paediatric and Perinatal Epidemiology, 31, 293-300.

JOHNSON, N., BISHOP, K., TROTMAN, H. & REID, M. 2012. Congenital abnormalities at a tertiary center in Jamaica: an 18-month maternal-fetal medicine experience. J Matern Fetal Neonatal Med, 25, 687-91.

KASHYAP, N., PRADHAN, M., SINGH, N. & YADAV, S. 2015. Early Detection of Fetal Malformation, a Long Distance Yet to Cover! Present Status and Potential of First Trimester Ultrasonography in Detection of Fetal Congenital Malformation in a Developing Country: Experience at a Tertiary Care Centre in India. Journal of Pregnancy, 2015, 623059.

KAZMI, S. S., NEJAT, F., TAJIK, P. & ROOZBEH, H. 2006. The antenatal ultrasonographic detection of myelomeningocele in patients referred to Children's Hospital Medical Center: a cross sectional study. Reprod Health, 3, 6.

KITISOMPRAYOONKUL, N. & TONGSONG, T. 2001. Neural tube defects: a different pattern in northern Thai population. Journal of the Medical Association of Thailand, 84, 483-8.

KOKEN, G., GUNGOR, A. N., CELIK, F., BATMAZ, G., UNLU, S., ARIOZ, D. & YILMAZER, M. 2014. Is three-dimensional antenatal ultrasound necessary: pregnant women's point of view. Ginekol Pol, 85, 31-6.

KONG, L. J., FAN, L., LI, G. H. & ZHANG, W. Y. 2017. Prevalence of congenital malformations during pregnancy in China: Screening by ultrasound examination. Clinical and Experimental Obstetrics and Gynecology, 44, 772-776.

Confidential: For Review Only

KOS, M., HAFNER, T., FUNDUK-KURJAK, B., BOZEK, T. & KURJAK, A. 2002. Limb deformities and three-dimensional ultrasound. J Perinat Med, 30, 40-7.
KUMAR, B., AGRAWAL, L. D. & SHARMA, S. B. 2008. Congenital bronchopulmonary malformations: a single-center experience and a review of literature. Ann Thorac Med, 3, 135-9.
KUMAR, M., GUPTA, U., THAKUR, S., AGGRAWAL, S., MEENA, J., SHARMA, S. & TRIVEDI, S. S. 2012. Antenatal sonographic evaluation and postnatal outcome of renal anomalies. Indian J Hum Genet, 18, 75-82.
KUMAR, M., SHARMA, S., BHAGAT, M., GUPTA, U., ANAND, R., PURI, A. & SINGH, A. 2013. Postnatal outcome of congenital anomalies in low resource setting. Antenatal Diagnosis, 33, 983-989.
KUMAR, M., THAKUR, S., PURI, A., SHUKLA, S., SHARMA, S., PERUMAL, V., CHAWLA, R. & GUPTA, U. 2014. Fetal renal anomaly: factors that predict survival. Journal of pediatric urology, 10, 1001-7.
KURJAK, A. & KOS, M. 1998. Ultrasound screening for fetal anomalies in developing countries: wish or reality? Annals of the New York Academy of Sciences, 847, 233-7.
KURJAK, A., HAFNER, T., KOS, M., KUPESIC, S. & STANOJEVIC, M. 2000. Three-dimensional sonography in antenatal diagnosis: a luxury or a necessity? J Perinat Med, 28, 194-209.
KUTUK, M. S., ALTUN, O., TUTUS, S., DOGAN, M. E., OZGUN, M. T. & DUNDAR, M. 2017. Antenatal diagnosis of upper extremity malformations with ultrasonography: Diagnostic features and perinatal outcome. J Clin Ultrasound, 45, 267-276.
LI, Q., GUAN, B. & LI, D. 2008. Detection of fetal structural abnormalities by early pregnancy ultrasound in China. International Journal of Gynaecology & Obstetrics, 100, 277-8.
LI, Z., REN, A., ZHANG, L., YE, R., LI, S., ZHENG, J., HONG, S., WANG, T. & LI, Z. 2006. Extremely high prevalence of neural tube defects in a 4-county area in Shanxi Province, China. Birth Defects Research, 76, 237-40.
LIAO, Y. M., LI, S. L., LUO, G. Y., WEN, H. X., OUYANG, S. Y., CHEN, C. Y., YAO, Y., BI, J. R. & TIAN, X. X. 2016. Routine screening for fetal limb abnormalities in the first trimester. Antenatal Diagnosis, 36, 117-26.
LIMA, J. S., CAMARGOS, P. A., AGUIAR, R. A., CAMPOS, A. S. & AGUIAR, M. J. 2014. Pre and perinatal aspects of congenital cystic adenomatoid malformation of the lung. Journal of Maternal-Fetal & Neonatal Medicine, 27, 228-32.
LISKER, R., CARNEVALE, A. & VILLA, A. R. 2006. Acceptance of induced abortion amongst medical students and physicians in Mexico. Revista de Investigacion Clinica, 58, 305-12.
LIU, Q. G., SUN, J., XIAO, X. W. & SONG, G. R. 2016. Birth defects data from surveillance hospitals in Dalian city, China, 2006-2010. Journal of Maternal-Fetal & Neonatal Medicine, 29, 3615-21.
LIU, S., WU, Q. & CHEN, Z. 2017. Fetal anomalies detection in China by screening with ultrasound. Biomedical Research (India), 28, 4891-4896.
LU, Q. B., WANG, Z. P., GONG, R., SUN, X. H., GAO, L. J. & ZHAO, Z. T. 2011. Investigation of ultrasound screening efficiency for neural tube defects during pregnancy in rural areas of China. Public Health, 125, 639-644.
LUIZA, A., NORONHA DE GOIS, D., SANTOS, J. A., BRITO DE OLIVEIRA, R. L. & FERREIRA DA SILVA, L. C. 2013. A descriptive epidemiology study of oral cleft in sergipe, Brazil. Int Arch Otorhinolaryngol, 17, 390-4.
MIRLESSE, V. & VILLE, I. 2013. The uses of ultrasonography in relation to foetal malformations in Rio de Janeiro, Brazil. Social Science & Medicine, 87, 168-75.

Confidential: For Review Only

MIRSHEMIRANI, A., KHALEGHNEJAD, A., ROUZROKH, M., SADEGHI, A., MOHAJERZADEH, L. & SHARIFIAN, M. 2013. Posterior Urethral Valves; A single Center Experience. Iran J Pediatr, 23, 531-5.
MUNIM, S., MAHEEN, H., ZAINAB, G. & FATIMA, S. 2013. Fetal outcome of cases with diaphragmatic hernia. Journal of Maternal-Fetal & Neonatal Medicine, 26, 1439-42.
MUNJANJA, S. P., MASONA, D., GWAZE, I. & CHIPATO, T. 1987. Audit of ultrasound scanning: antenatal diagnosis of congenital abnormalities in Harare, Zimbabwe. East African Medical Journal, 64, 601-5.
NEOGI, S. B. 2010. Abortion for fetal abnormalities in India: need for critical review. Asia-Pacific Journal of Public Health, 22, 146-7.
NETO, C. N., DE SOUZA, A. S. R., DE MORAES FILHO, O. B. & NORONHA, A. M. B. 2009. Validation of ultrasound diagnosis of fetal anomalies at a reference center in Pernambuco. [Portuguese]. Revista da Associacao Medica Brasileira, 55, 541-546.
NOVAES, H. M. 2000. Social impacts of technological diffusion: antenatal diagnosis and induced abortion in Brazil. Social Science & Medicine, 50, 41-51.
OGNEAN, M. L., ZGARCEA, L. C., BALANESCU, L., BOANTA, O., DUMITRA, R. E., GROSU, F., BRATU, D. G., BOICEAN, A. G., COLDEA, L. & CHICEA, R. 2017. Esophageal atresia with distal fistula - unusual case series. Considerations related to epidemiological aspects, malformative associations, and antenatal diagnosis. Rom J Morphol Embryol, 58, 1069-1076.
OKAFOR, H. U., EKENZE, S. O. & UWAEZUOKE, S. N. 2013. Posterior urethral valves: Determinants of outcome in a developing country. Journal of Paediatrics and Child Health, 49, 115-119.
OLOYEDE, O. A. & OYEDELE, R. A. 2008. Women's attitude to antenatal screening services for congenital abnormalities in Nigeria. Journal of Obstetrics & Gynaecology, 28, 406-7.
ONKAR, D., ONKAR, P. & MITRA, K. 2014. Evaluation of Fetal Central Nervous System Anomalies by Ultrasound and Its Anatomical Co-relation. J Clin Diagn Res, 8, Ac05-7.
ONYAMBU, C. K. & THARAMBA, N. M. 2018. Screening for congenital fetal anomalies in low risk pregnancy: The Kenyatta National Hospital experience. BMC Pregnancy and Childbirth, 18 (1) (no pagination).
ORGUL, G., SOYER, T., YURDAKOK, M. & BEKSAC, M. S. 2018. Evaluation of pre- and postnatally diagnosed gastrointestinal tract obstructions. Journal of Maternal-Fetal & Neonatal Medicine, 1-6.
OSIFO, O. D., OVUENI, M. E. & EVBUOMWAN, I. 2011. Omphalocele management using goal-oriented classification in African centre with limited resources. Journal of Tropical Pediatrics, 57, 286-288.
OZTARHAN, K., GEDIKBASI, A., YILDIRIM, D., ARSLAN, O., ADAL, E., KAVUNCUOGLU, S., OZBEK, S. & CEYLAN, Y. 2010. Prevalence and distribution of congenital abnormalities in Turkey: differences between the antenatal and postnatal periods. Congenital Anomalies, 50, 221-5.
OZTEKIN, O., OZTEKIN, D., TINAR, S. & ADIBELLI, Z. 2009. Ultrasonographic diagnosis of fetal structural abnormalities in antenatal screening at 11-14 weeks. Diagn Interv Radiol, 15, 221-5.
PELIZZARI, E., VALDEZ, C. M., PICETTI JDOS, S., CUNHA, A. C., DIETRICH, C., FELL, P. R., TARGA, L. V., ZEN, P. R. & ROSA, R. F. 2015. Characteristics of fetuses evaluated due to suspected anencephaly: a population-based cohort study in southern Brazil. Sao Paulo Medical Journal = Revista Paulista de Medicina, 133, 101-8.

Confidential: For Review Only

PENCHASZADEH, V. B. 2002. Preventing congenital anomalies in developing countries. Community Genetics, 5, 61-69.
PEREIRA, A. M., MONTEIRO, D. L. M., WERNER, H., DALTRO, P., FAZECAS, T., GUEDES, B., TONNI, G., PEIXOTO, A. B. & ARAUJO JUNIOR, E. 2018. Zika virus and pregnancy in Brazil: What happened? J Turk Ger Gynecol Assoc, 19, 39-47.
PHADKE, S. R., AGARWAL, M. & AGGARWAL, S. 2011. Late termination of pregnancy for fetal abnormalities: The perspective of Indian lay persons and medical practitioners. Antenatal Diagnosis, 31, 1286-1291.
PITUKKIJRONNAKORN, S., CHITTACHAROEN, A., JETSAWANGSRI, T., PANBURANA, P., JAOVISIDHA, A., ROUNGSIPRAGARN, R., SAROPALA, N. & HERABUTYA, Y. 2009. The value of mid-trimester routine ultrasonographic screening in antenatal detection of congenital malformations. J Med Assoc Thai, 92, 748-53.
POSTOEV, V. A., GRJIBOVSKI, A. M., NIEBOER, E. & ODLAND, J. O. 2015. Changes in detection of birth defects and perinatal mortality after introduction of antenatal ultrasound screening in the Kola Peninsula (North-West Russia): combination of two birth registries. BMC Pregnancy & Childbirth, 15, 308.
QAISRANI, H. G., HUSSAIN, M. A., ALI, M., QAISRANI, G. H. & RIZVI, S. 2013. Role of 1st trimester ultrasonography in detecting fetal abnormalities versus 20 weeks scan in high risk women. Pakistan Journal of Medical and Health Sciences, 7, 1170-1173.
RAMAN, V. S., AGARWALA, S., BHATNAGAR, V., PANDA, S. S. & GUPTA, A. K. 2015. Congenital cystic lesions of the lungs: The perils of misdiagnosis - A single-center experience. Lung India, 32, 116-8.
RANJI, A. & DYKES, A. K. 2012. Ultrasound screening during pregnancy in Iran: womens' expectations, experiences and number of scans. Midwifery, 28, 24-9.
RASHID, S. Q. 2002. A study of fetal anomalies detected by ultrasound in Bangladesh and their relative frequencies. Journal of the Royal Society for the Promotion of Health, 122, 55-7.
RATTAN, K. N., SINGH, J. & DALAL, P. 2017. Clinical profile and short-term outcome of neonates with esophageal atresia and tracheoesophageal fistula at tertiary care center in a developing country: A 25-year experience. Journal of Clinical Neonatology, 6, 225-230.
RODRIGUEZ, L., SANCHEZ, R., HERNANDEZ, J., CARRILLO, L., OLIVA, J. & HEREDERO, L. 1997. Results of 12 years' combined maternal serum alpha-fetoprotein screening and ultrasound fetal monitoring for antenatal detection of fetal malformations in Havana City, Cuba. Antenatal Diagnosis, 17, 301-4.
ROSSELLI, P., NOSSA, S., HUERFANO, E., BETANCUR, G., GUZMAN, Y., CASTELLANOS, C. & MORCUENDE, J. 2015. Antenatal Ultrasound Diagnosis of Congenital Talipes Equinovarus in Bogota (Colombia) Between 2003 and 2012. Iowa Orthopaedic Journal, 35, 156-9.
ROUTHU, M., THAKKALLAPELLI, S., MOHAN, P. & AHMED, N. 2016. Role of Ultrasound in Body Stalk Anomaly and Amniotic Band Syndrome. Int J Reprod Med, 2016, 3974139.
RUANO, R., BUNDUKI, V., SILVA, M. M., YOSHIZAKI, C. T., TANURI, U., MACKSOUD, J. G. & ZUGAIB, M. 2006. Antenatal diagnosis and perinatal outcome of 38 cases with congenital diaphragmatic hernia: 8-year experience of a tertiary Brazilian center. Clinics (Sao Paulo), 61, 197-202.
SAHA, A., BATRA, P., CHATURVEDI, P., MEHERA, B. & TAYADE, A. 2009. Antenatal detection of renal malformations. Indian Pediatr, 46, 346-8.

Confidential: For Review Only

SAHIN, N. H. & GUNGOR, I. 2008. Congenital anomalies: parents' anxiety and women's concerns before antenatal testing and women's opinions towards the risk factors. Journal of Clinical Nursing, 17, 827-36.
SAHINOGLU, Z., ULUDOGAN, M., ARIK, H., AYDIN, A., KUCUKBAS, M., BILGIC, R. & TOKSOY, G. 2007. Antenatal ultrasonographical features of limb body wall complex: a review of etiopathogenesis and a new classification. Fetal Pediatr Pathol, 26, 135-51.
SALDARRIAGA, G. W., RUIZ MURCIA, F. A., FANDINO-LOSADA, A., CRUZ PEREA, M. E. & ISAZA DE LOURIDO, C. 2014. Evaluation of antenatal diagnosis of congenital anomalies diagnosable by antenatal ultrasound in patients in neonatal intensive care units of Cali, Colombia. Colombia Medica, 45, 32-38.
SAMADIRAD, B., KHAMNIAN, Z., HOSSEINI, M. B. & DASTGIRI, S. 2012. Congenital anomalies and termination of pregnancy in Iran. Journal of pregnancy, 2012, 574513.
SANGHVI, K. P., MERCHANT, R. H., GONDHALEKAR, A., LULLA, C. P., MEHTA, A. A. & MEHTA, K. P. 1998. Antenatal diagnosis of congenital renal malformations using ultrasound. J Trop Pediatr, 44, 235-40.
SANKAR, V. H. & PHADKE, S. R. 2006. Clinical utility of fetal autopsy and comparison with antenatal ultrasound findings. J Perinatol, 26, 224-9.
SANTOS, M. M., RUBAGUMYA, D. K., DOMINIC, I., BRIGHTON, A., COLOMBE, S., O'DONNELL, P., ZUBKOV, M. R. & HARTL, R. 2017. Infant hydrocephalus in sub-Saharan Africa: The reality on the Tanzanian side of the lake. Journal of Neurosurgery: Pediatrics, 20, 423-431.
SARIN, Y. K., SHARMA, A., SINHA, S. & DESHPANDE, V. P. 2012. Duodenal webs: an experience with 18 patients. J Neonatal Surg, 1, 20.
SAVRAN, B., ADIGUZEL, U., YUKSEL, K. B., SEVEN, A., FAZLI, O., KABIL KUCUR, S. & OZTURK GOZUKARA, I. 2016. The importance of antenatal diagnosis of congenital duodenal obstruction. Ir J Med Sci, 185, 695-8.
SEKABIRA, J. & HADLEY, G. P. 2009. Gastroschisis: a third world perspective. Pediatric Surgery International, 25, 327-9.
SENANAYAKE, H. M. & DE SILVA, M. V. 2001. Antenatal diagnosis of lethal congenital malformations in Sri Lanka. Ceylon Medical Journal, 46, 163.
SENANAYAKE, H., DE SILVA, D., PREMARATNE, S. & KULATUNGE, M. 2006. Psychological reactions and coping strategies of Sri Lankan women carrying fetuses with lethal congenital malformations. Ceylon Med J, 51, 14-7.
SHAHKAR, L. S., S.; BIGLARI, H. N.; POURBAKHTYARAN, E.; TABATABAI, S. A.; FAKHRAEE, S. H. 2016. An eleven-year review of congenital pulmonary lesions in a referral children's hospital in Tehran. Archives of Pediatric Infectious Diseases 4.
SHALABY, H. A., ELHADY, R. A., GAMAL, A. M. & BADRY, A. A. 2012. Antenatal diagnosis in low resource setting: is it acceptable? J Obstet Gynaecol India, 62, 515-9.
SHALABY, H., HEMIDA, R., NABIL, H. & IBRAHIM, M. 2016. Types and Outcome of Fetal Urinary Anomalies in Low Resource Setting Countries: A Retrospective Study. J Obstet Gynaecol India, 66, 316-20.
SHARADA, S., VIJAYAKUMAR, M., NAGESWARAN, P., EKAMBARAM, S. & UDANI, A. 2014. Multicystic dysplastic kidney: a retrospective study. Indian Pediatr, 51, 641-3.
SHI, C. R., CAI, W., JIN, H. M., CHEN, F., ZHOU, Y. & ZHOU, D. X. 2006. Surgical management to conjoined twins in Shanghai area. Pediatr Surg Int, 22, 791-5.

Confidential: For Review Only

SIDDESH, A., GUPTA, G., SHARAN, R., AGARWAL, M. & PHADKE, S. R. 2017. Spectrum of antenatally detected central nervous system malformations: Neural tube defects continue to be the leading foetal malformation. Indian Journal of Medical Research, 145, 471-478.
SINGH, J. K., KANOJIA, R. P. & NARASIMHAN, K. L. 2009. Multicystic dysplastic kidney in children--a need for conservative and long term approach. Indian Journal of Pediatrics, 76, 809-12.
SOLANKI, S., BHATNAGAR, V., GUPTA, A. K. & KUMAR, R. 2013. Crossed fused renal ectopia: Challenges in diagnosis and management. J Indian Assoc Pediatr Surg, 18, 7-10.
SOOD, M., AGARWAL, N., VERMA, S. & BHARGAVA, S. K. 1991. Neural tubal defects in an east Delhi hospital. Indian Journal of Pediatrics, 58, 363-5.
SORRI, G. & MESFIN, E. 2015. Patterns of Neural Tube Defects at Two Teaching Hospitals in Addis Ababa, Ethiopia a Three Years Retrospective Study. Ethiopian Medical Journal, 53, 119-26.
SRISUPUNDIT, K., TONGSONG, T., SIRICHOTIYAKUL, S. & CHANPRAPAPH, P. 2006. Fetal structural anomaly screening at 11-14 weeks of gestation at Maharaj Nakorn Chiang Mai Hospital. Journal of the Medical Association of Thailand, 89, 588-93.
TABEL, Y., HASKOLOGLU, Z. S., KARAKAS, H. M. & YAKINCI, C. 2010. Ultrasonographic screening of newborns for congenital anomalies of the kidney and the urinary tracts. Urol J, 7, 161-7.
TANNURI, A. C. A., SBRAGIA, L., TANNURI, U., SILVA, L. M., LEAL, A. J. G., SCHMIDT, A. F. S., OLIVEIRA-FILHO, A. G., BUSTORFF-SILVA, J. M., VICENTE, Y. A. M. V. A., TAZIMA, M. F. G. S., PILEGGI, F. O. & CAMPERONI, A. L. 2011. Evolution of critically ill patients with gastroschisis from three tertiary centers. Clinics, 66, 17-20.
TARCA, E. & APRODU, S. 2014. Past and present in omphalocele treatment in Romania. Chirurgia (Bucur), 109, 507-13.
TARCA, E. & APRODU, S. G. 2013. Gastroschisis treatment: What are the causes of high morbidity and mortality rates? Chirurgia (Romania), 108, 516-520.
TARCA, E., CIONGRADI, I. & APRODU, S. G. 2015. Birth Weight, Compromised Bowel and Sepsis are the Main Variables Significantly Influencing Outcome in Gastroschisis. Chirurgia (Bucuresti), 110, 151-6.
TASKAPILIOGLU, M. O., ISIK, S. & KORFALI, E. 2014. Outcome of Myelomeningocele: The importance of antenatal diagnosis. Neurosurgery Quarterly, 24, 214-217.
TITAPANT, V. C., P. 2015. Psychological effects of fetal diagnoses of non-lethal congenital anomalies on the experience of pregnant women during the remainder of their pregnancy. Journal of Obstetrics & Gynaecology Research, 41, 77-83.
TUDORACHE, S., CHIUTU, L. C., ILIESCU, D. G., GEORGESCU, R., STOICA, G. A., SIMIONESCU, C. E., GEORGESCU, E. F. & NEMES, R. N. 2014. Antenatal diagnosis and perinatal outcome in congenital diaphragmatic hernia. Single tertiary center report. Romanian journal of morphology and embryology = Revue roumaine de morphologie et embryologie, 55, 823-833.
ULKUMEN, B. A., PALA, H. G., BAYTUR, Y. & KOYUNCU, F. M. 2014. Retrospective analysis of fetal anterior abdominal wall defects. Saudi Medical Journal, 35, 1147-1149.
VELAZQUEZ-TORRES, B., GALLARDO-GAONA, J. M., ACEVEDO-GALLEGOS, S., RAMIREZ-CALVO, J. A., BENAVIDES-SERRALDE, J. A., CAMARGO-MARIN, L., CERVANTES-PARRA, L. E., MARTINEZ-JUAREZ, A. & GUZMAN-HUERTA, M. E. 2012. Major craniofacial defects: case series and antenatal diagnosis at the Instituto Nacional de Perinatologia, Mexico. Revista de Investigacion Clinica, 64, 420-8.

Confidential: For Review Only

VIJAYARAGHAVAN, S. B., PREMA, A. S. & SUGANYADEVI, P. 2011. Sonographic depiction of the fetal anus and its utility in the diagnosis of anorectal malformations. J Ultrasound Med, 30, 37-45.
VILELA, P. C., RAMOS DE AMORIM, M. M., FALBO, G. H. & SANTOS, L. C. 2001. Risk factors for adverse outcome of newborns with gastroschisis in a Brazilian hospital. J Pediatr Surg, 36, 559-64.
VILLE, I. & MIRLESSE, V. 2015. Antenatal diagnosis: From policy to practice. Two distinct ways of managing prognostic uncertainty and anticipating disability in Brazil and in France. Soc Sci Med, 141, 19-26.
WENG, R., HU, W., CAI, S., GUO, X. & LUO, Q. 2016. Antenatal diagnosis and prognosis assessment of congenital choledochal cyst in 21 cases. J Obstet Gynaecol, 36, 324-7.
WESONGA, A. S., FITZGERALD, T. N., KABUYE, R., KIRUNDA, S., LANGER, M., KAKEMBO, N., OZGEDIZ, D. & SEKABIRA, J. 2016. Gastroschisis in Uganda: Opportunities for improved survival. Journal of Pediatric Surgery, 51, 1772-1777.
WRIGHT, N. J., ZANI, A. & ADE-AJAYI, N. 2015. Epidemiology, management and outcome of gastroschisis in Sub-Saharan Africa: Results of an international survey. African journal of paediatric surgery : AJPS, 12, 1-6.
WYSZYNSKI, D. F., PERANDONES, C. & BENNUN, R. D. 2003. Attitudes toward antenatal diagnosis, termination of pregnancy, and reproduction by parents of children with nonsyndromic oral clefts in Argentina. Prenat Diagn, 23, 722-7.
XIE, H. N., LI, L. J., HE, H., SHI, H. J., PENG, R. & ZHU, Y. X. 2009. Antenatal surveillance of bronchopulmonary sequestration using 3-dimensional ultrasonography. J Ultrasound Med, 28, 989-94.
XU, H. X., ZHANG, Q. P., LU, M. D. & XIAO, X. T. 2002. Comparison of two-dimensional and three-dimensional sonography in evaluating fetal malformations. J Clin Ultrasound, 30, 515-25.
YONG, S. L. D., F.; HAMIZAH, I.; ROZIHAN, I.; MOKHTAR, A.; SUHAIZA, A. 2012. Maternal outcome of early versus late termination of pregnancy among pregnant mothers with antenatal diagnosis of lethal fetal anomalies: A retrospective review. BJOG: An International Journal of Obstetrics and Gynaecology, 1, 77.
ZHANG, H., TIAN, J., CHEN, Z., MA, X., YU, G., ZHANG, J., JIANG, G. & WANG, L. 2014. Retrospective study of antenatal diagnosed pulmonary sequestration. Pediatr Surg Int, 30, 47-53.

Confidential: For Review Only

Supplementary File 5

Investigating the Use of Ultrasonography for the Antenatal Diagnosis of Structural Congenital Anomalies in Low- and Middle-Income Countries: A Systematic Review

List of Countries Represented in Systematic Review Data Extraction

**Country**	**No. of Studies (%)**
India	27 (17.53%)
China	19 (12.34%)
Brazil	18 (11.69%)
Turkey	16 (10.39%)
Nigeria	11 (7.14%)
Romania	8 (5.20%)
Iran	6 (3.90%)
Argentina	5 (3.24%)
Colombia	5 (3.24%)
Thailand	4 (2.60%)
South Africa	3 (1.94%)
Mexico	3 (1.94%)
Pakistan	2 (1.30%)
Russia	2 (1.30%)
Kenya	2 (1.30%)
Malaysia	2 (1.30%)
Croatia	2 (1.30%)
Egypt	2 (1.30%)
Bangladesh	1 (0.65%)
Burkina Faso	1 (0.65%)
Cameroon	1 (0.65%)
Chile	1 (0.65%)
Cuba (USA)	1 (0.65%)
Ethiopia	1 (0.65%)
Indonesia	1 (0.65%)
Ivory Coast	1 (0.65%)
Jamaica	1 (0.65%)
Jordan	1 (0.65%)
Sri Lanka	1 (0.65%)
Tanzania	1 (0.65%)
Tunisia	1 (0.65%)
Uganda	1 (0.65%)
Venezuela	1 (0.65%)
Vietnam	1 (0.65%)
Zimbabwe	1 (0.65%)
**Total**	**154 (100%)**

Confidential: For Review Only

BMJ Paediatrics Open

Investigating the Use of Ultrasonography for the Antenatal Diagnosis of Structural Congenital Anomalies in Low- and Middle-Income Countries: A Systematic Review

Journal:	*BMJ Paediatrics Open*
Manuscript ID	bmjpo-2020-000684.R1
Article Type:	Original research
Date Submitted by the Author:	11-Jun-2020

Complete List of Authors:	Goley, Stephanie; King's College London, Department of Global Health & Social Medicine<br>Sakula-Barry, Sidonie; World Cancer Research Fund<br>Adofo-Ansong, Nana; Mafikeng Provincial Hospital<br>Isaaya Ntawunga, Laurence; University of Rwanda, Rwanda Spina Bifida and Hydrocephalus Relief<br>Tekyiwa Botchway, Maame; University of the Witwatersrand<br>Kelly, Ann; King's College London, Department of Global Health & Social Medicine<br>Wright, Naomi; King's College London, King's Centre for Global Health and Health Partnerships

Keywords:	Screening, Neonatology, Congenital Abnorm

SCHOLARONE™
Manuscripts

I, the Submitting Author has the right to grant and does grant on behalf of all authors of the Work (as defined in the below author licence), an exclusive licence and/or a non-exclusive licence for contributions from authors who are: i) UK Crown employees; ii) where BMJ has agreed a CC-BY licence shall apply, and/or iii) in accordance with the terms applicable for US Federal Government officers or employees acting as part of their official duties; on a worldwide, perpetual, irrevocable, royalty-free basis to BMJ Publishing Group Ltd ("BMJ") its licensees and where the relevant Journal is co-owned by BMJ to the co-owners of the Journal, to publish the Work in this journal and any other BMJ products and to exploit all rights, as set out in our licence.

The Submitting Author accepts and understands that any supply made under these terms is made by BMJ to the Submitting Author unless you are acting as an employee on behalf of your employer or a postgraduate student of an affiliated institution which is paying any applicable article publishing charge ("APC") for Open Access articles. Where the Submitting Author wishes to make the Work available on an Open Access basis (and intends to pay the relevant APC), the terms of reuse of such Open Access shall be governed by a Creative Commons licence – details of these licences and which Creative Commons licence will apply to this Work are set out in our licence referred to above.

Other than as permitted in any relevant BMJ Author's Self Archiving Policies, I confirm this Work has not been accepted for publication elsewhere, is not being considered for publication elsewhere and does not duplicate material already published. I confirm all authors consent to publication of this Work and authorise the granting of this licence.

Confidential: For Review Only

Investigating the Use of Ultrasonography for the Antenatal Diagnosis of Structural Congenital Anomalies in Low- and Middle-Income Countries: A Systematic Review

Stephanie Goley[1], Sidonie Sakula-Barry[2], Nana Adofo-Ansong[3], Laurence Isaaya Ntawunga[4], Maame Tekyiwa Botchway[5], Ann H. Kelly[1], Naomi Wright[6]

[1]Department of Global Health & Social Medicine, King's College London, London, UK
[2]World Cancer Research Fund UK, London, UK
[3]Mafikeng Provincial Hospital, Mafikeng, South Africa
[4]Rwanda Spina Bifida and Hydrocephalus Relief, University of Rwanda, Kigali, Rwanda
[5]University of Witwatersrand, Johannesburg, South Africa
[6]King's Centre for Global Health and Health Partnerships, School of Population Health and Environmental Sciences, King's College London, London, UK

Corresponding Author:

Stephanie Goley, BA MSc
Department of Global Health & Social Medicine, King's College London, London, UK
Correspondence to: Centre for Global Health and Health Partnerships, Suite 2.13 Weston Education Centre, Cutcombe Road, London, SE5 9RJ
Telephone: +1 (815) 830-5360

Authors' Email Addresses:

stephanie.goley@kcl.ac.uk
s.sakulabarry@gmail.com
adfnan001@myuct.ac.za
rsbhrelief@gmail.com
1596010@students.wits.ac.za
ann.kelly@kcl.ac.uk
naomiwright@doctors.org.uk

Confidential: For Review Only

What is known?

- Congenital anomalies are the fifth leading cause of death in children under 5-years of age globally.
- 97% of congenital anomaly deaths occur in LMICs, many of which may be preventable with antenatal diagnosis and planned surgical intervention following birth.
- Antenatal ultrasound examinations in HICs are commonplace and highly accurate, but accessibility and effectiveness are limited in LMICs.

What this study adds?

- Rates of antenatal ultrasound examination vary significantly in LMICs, ranging from 6.8% to 98.8%.
- There is significant variation in the accuracy of antenatal diagnosis in LMICs, with detection rates varying from 0% to 100% (mean of 28.0% in Africa).
- Available data suggests that the level of ultrasonographer training may affect the accuracy of diagnosis, but further research into this is required.

Confidential: For Review Only

Abstract

Background: Congenital anomalies are the 5th leading cause of under-5 mortality globally. The greatest burden is faced by those in low- and middle-income countries (LMICs), where over 95% of deaths occur. Many of these deaths may be preventable through antenatal diagnosis and early intervention. This systematic literature review investigates the use of antenatal ultrasound to diagnose congenital anomalies and improve the health outcomes of infants in LMICs.

Methods: A systematic literature review was conducted using three search strings: 1) structural congenital anomalies; 2) LMICs; and 3) antenatal diagnosis. The search was conducted on the following databases: MEDLINE, EMBASE, PubMed, and the Cochrane Library. Title, abstract and full-text screening was undertaken in duplicate by two reviewers independently. Consensus among the wider authorship was sought for discrepancies. The primary analysis focused on the availability and effectiveness of antenatal ultrasound for diagnosing structural congenital anomalies. Secondary outcomes included neonatal morbidity and mortality, termination rates, referral rates for further antenatal care and training level of the ultrasonographer. Relevant policy data was sought.

Results: The search produced 4,062 articles; 97 were included in the review. The proportion of women receiving an antenatal ultrasound examination was 13.0% in African studies, 90.6% in Asian studies and 98.2% in one Jamaican study (range 6.8% to 98.8%). Detection rates were: 28.0% Africa, 37.9% Asia, 42.6% Europe, 47.2% South America, 77.2% Jamaica (range 0 to 100%). The training level of the ultrasound provider may affect detection rates. Four articles compared morbidity and mortality outcomes, with inconclusive results. Significant variations in termination rates were found (0 to 98.3%). No articles addressed referral rates.

Conclusion: Antenatal detection of congenital anomalies remains highly variable across LMICs and is particularly low in sub-Saharan Africa. Further research is required to investigate the role of antenatal diagnosis for improving survival from congenital anomalies in LMICs.

PROSPERO Registration ID: CRD42019105620

Introduction

Congenital anomalies are one of the leading causes of neonatal morbidity and mortality globally. The greatest burden of disease is faced by those in low- and middle-income countries (LMICs), as 94% of congenital anomalies occur in these regions [1]. Congenital anomalies comprise 9% of the total global burden of surgical disease and account for 57.7 million disability-adjusted life years (DALYs) lost annually across the globe [2]. Recent estimates suggest that approximately 303,000 neonates die annually from congenital anomalies before reaching just four weeks of age [3]. However, many experts believe that this is an under-estimate, due to a lack of congenital anomaly registries and some neonates dying without a diagnosis or inclusion within current statistics.

The WHO defines congenital anomalies as either structural or functional abnormalities which occur during intrauterine development [3]. Structural anomalies are physical abnormalities that occur when the organs or skeletal structure are improperly formed. These can often be detected on ultrasound antenatally and are the focus of this review. Some common structural congenital anomalies include heart defects, cleft lip and palate, neural tube defects, limb deformities, and abdominal wall defects. Many structural anomalies require immediate surgical intervention at birth to avoid death or preventable disability. In such cases, antenatal diagnosis permits delivery at a center where the appropriate surgical care can be provided upon delivery. For example, gastroschisis where the intestines protrude through a hole in the abdominal wall at birth. In HICs, where the majority of cases are antenatally diagnosed, mortality is less than 5%, while in many LMICs, with limited antenatal diagnosis, the mortality rate can be as high as 100% [4-6].

The use of ultrasound technology in LMICs has significantly increased in recent years, as ultrasound machines have become more compact, transportable, and affordable [7]. Yet, a great number of congenital anomalies that can be detected antenatally via ultrasound go undiagnosed. Factors identified as barriers to effective antenatal ultrasound include limited training, equipment shortages, faulty ultrasound equipment, and lack of maintenance services [7]. In recent years, higher global priority has been given to neonatal health. Sustainable Development Goal (SDG) 3.2 aims to end all preventable under-5 deaths and reduce neonatal mortality in every country to 12 per 1,000 live births [8]. In 2010, the WHO released the 63rd World Health Assembly Report on Birth Defects, recommending 'prevention whenever possible, to implement screening programs and to provide care and ongoing support to children with birth defects and their families' [9].

To develop a better understanding of antenatal ultrasound provision in LMICs, this study aimed to systematically investigate the availability and effectiveness of antenatal ultrasound in the

Confidential: For Review Only

diagnosis of structural congenital anomalies in LMICs. It further aimed to evaluate the effects of antenatal ultrasound diagnosis on mortality and morbidity outcomes, termination rates and referral for further antenatal care and management planning. Additionally, it assessed the level of training of ultrasonographers undertaking antenatal scans and relevant antenatal ultrasound policies in LMICs. This information is vital to help clarify the existing disparities in antenatal ultrasound provision and the potential benefits for improved health outcomes.

Methodology

Preferred Reporting Items for Systematic review and Meta-Analysis (PRISMA) guidelines have been followed when conducting this systematic review (Table 1) [10, 11]. A protocol for this systematic review was published in *BMJ Paediatrics Open* [12].

Table 1: PRISMA (Preferred Reporting Items for Systematic Reviews and Meta-Analyses) 2009 Checklist [11]

Section/topic	#	Checklist item	Section Heading
**TITLE**			
Title	1	Identify the report as a systematic review, meta-analysis, or both.	Title
**ABSTRACT**			
Structured summary	2	Provide a structured summary including, as applicable: background; objectives; data sources; study eligibility criteria, participants, and interventions; study appraisal and synthesis methods; results; limitations; conclusions and implications of key findings; systematic review registration number.	Abstract
**INTRODUCTION**			
Rationale	3	Describe the rationale for the review in the context of what is already known.	Introduction
Objectives	4	Provide an explicit statement of questions being addressed with reference to participants, interventions, comparisons, outcomes, and study design (PICOS).	Introduction
**METHODS**			
Protocol and registration	5	Indicate if a review protocol exists, if and where it can be accessed (e.g., Web address), and, if available, provide registration information including registration number.	Methodology
Eligibility criteria	6	Specify study characteristics (e.g., PICOS, length of follow-up) and report characteristics (e.g., years considered, language, publication status) used as criteria for eligibility, giving rationale.	Methodology
Information sources	7	Describe all information sources (e.g., databases with dates of coverage, contact with study authors to identify additional studies) in the search and date last searched.	Methodology
Search	8	Present full electronic search strategy for at least one database, including any limits used, such that it could be repeated.	Methodology
Study selection	9	State the process for selecting studies (i.e., screening, eligibility, included in systematic review, and, if applicable, included in the	Methodology

Confidential: For Review Only

		meta-analysis).	
Data collection process	10	Describe method of data extraction from reports (e.g., piloted forms, independently, in duplicate) and any processes for obtaining and confirming data from investigators.	Methodology
Data items	11	List and define all variables for which data were sought (e.g., PICOS, funding sources) and any assumptions and simplifications made.	Methodology
Risk of bias in individual studies	12	Describe methods used for assessing risk of bias of individual studies (including specification of whether this was done at the study or outcome level), and how this information is to be used in any data synthesis.	Methodology
Summary measures	13	State the principal summary measures (e.g., risk ratio, difference in means).	Methodology
Synthesis of results	14	Describe the methods of handling data and combining results of studies, if done, including measures of consistency (e.g., I^2) for each meta-analysis.	Methodology

Search Strategy

A search was conducted using three search strings: 1) structural congenital anomalies, 2) LMICs, 3) antenatal diagnosis using ultrasound (Supplementary File 1). Using the Ovid program, an electronic database search was conducted on MEDLINE, EMBASE, PubMed, and the Cochrane Library. These searches were filtered to only include studies with human subjects. An example of the search in MEDLINE can be found in Supplementary File 2. Only fetuses with a structural congenital anomaly as listed in search string 1 were included. Only studies from LMICs were included; these were limited to the English language. Studies with less than five patients were excluded. A further search was conducted on the WHO website to identify relevant grey literature, particularly related to antenatal ultrasound policy. The following terms were searched in the WHO Reproductive Health Library: ultrasound, ultrasonography, congenital anomalies, congenital abnormalities, congenital anomaly, congenital abnormality, birth defect, antenatal detection, prenatal detection, antenatal diagnosis, and prenatal diagnosis. Following the search of each term, the results were expanded using a snowball strategy to optimize the inclusion of all relevant data.

Study Design

All forms of evidence-based research were included. This includes systematic reviews, meta-analyses, randomized controlled trials, descriptive observational studies, case-control studies, cohort studies, and case series.

Methodological Quality

Although the researchers intended to use the Cochrane Risk of Bias for Non-Randomized Studies of Interventions (ROBINS-I) and the revised tool to assess Risk of Bias in Randomized Trials (RoB) V.2.0. to evaluate methodological quality, the majority of studies included in this

Confidential: For Review Only

systematic review were not interventional studies. Overall, the data was heterogenous and descriptive in nature, which was not suitable for existing quality assessment tools.

Study Screening

References produced from the search results were added to EndNote X8 and duplicates were removed. The articles were then uploaded to Covidence and screened in duplicate. Articles that did not meet the study criteria were removed.

Data Extraction and Synthesis

Data extraction was undertaken by the principal investigator. The data extraction table can be found in Supplementary File 3. The primary analysis focused on the availability and effectiveness of antenatal ultrasound for structural congenital anomalies. Secondary outcomes included neonatal morbidity and mortality, termination rates, and referral rates for further antenatal care. The results are presented in tables and descriptive statistics (range and mean) have been calculated regionally.

Patient and Public Involvement

Given that this is a systematic literature review, there was no patient or public involvement for the collection of data and literature review. Public involvement will be important for prioritizing antenatal ultrasound on the political agenda and improving antenatal care programs. To disseminate the results of this study, international charities and organizations involving structural congenital anomalies will be approached to assist in circulation.

Results

Study Screening

The search produced 4,062 articles. Of these, 745 duplicates were removed. The remaining 3,317 articles underwent abstract and title review by two independent reviewers. Of the 3,317 articles screened, 2,826 were excluded. 491 articles were then reviewed by two independent reviewers in full-text. At this stage, 316 articles were excluded; 73 for non-English language (Supplementary File 4).

175 articles were found to meet all inclusion criteria listed in the search strings. Of these, 78 provided no data relevant to the study and thus were excluded. 97 studies were included in the data extraction phase (Figure 1). Although all LMICs as defined by the World Bank were included in the search, not all countries yielded results in the text screening. 138 LMICs were included in the literature search, however, only 29 countries (21%) had any data that met the inclusion criteria (Figure 2, Supplementary File 5).

It is also notable that the majority of included studies were conducted on an institutional level. Thus, while the data from these studies provides important information from the countries of this review, they are by no means a representative sample of an entire country or even an entire city.

Confidential: For Review Only

Each article also varied widely in the information it provided, ranging from antenatal detection rates to policy analysis. Given the heterogeneity of data extrapolated from these articles, it was not feasible to perform a meta-analysis.

Table 2: Percentage of Women Receiving Antenatal Ultrasound

Author(s)	Study Location	Study Type	Study Population	Number of Women in Study	Number of Women Who Received Antenatal Ultrasound (%)
**Africa**					
de Paul Djientcheu et al., 2008 [13]	Cameroon	Retrospective descriptive observational study	Institutional; patients with NTDs* admitted to neonatology unit	69	27 (39.1%)
Abdur-Rahman et al., 2011 [14]	Nigeria	Retrospective descriptive observational study	Institutional; patients with abdominal wall defects at a tertiary health center in the North-Central geopolitical zone of Nigeria	56	51 (91.1%)
Adeleye et al., 2010 [15]	Nigeria	Prospective cross-sectional study	Institutional; patients presenting with major CNS* anomalies at tertiary hospital	54	43 (79.6%)
Adeleye and Joel-Medewase, 2015 [16]	Nigeria	Retrospective cross-sectional survey	Institutional; patients with CNS defects at a neurosurgeon's practice	151	91 (60.3%)
Bankole et al., 2012 [17]	Nigeria	Prospective descriptive observational study	Institutional; patients presenting with CNS anomalies at tertiary hospital	108	54 (50%)
Idowu and Olawehinmi, 2012 [18]	Nigeria	Prospective descriptive observational study	Institutional; patients presenting with NTDs at tertiary hospital	94	91 (96.8%)
Okafor et al., 2013 [19]	Nigeria	Prospective cohort study	Institutional; patients with PUV* at tertiary hospital	31	22 (71%)
Sekabira and Hadley, 2009 [20]	South Africa	Retrospective descriptive observational study	Institutional; patients with gastroschisis at tertiary hospital	106	25 (23.6%)
Santos et al., 2017 [21]	Tanzania	Prospective descriptive observational study	Institutional; patients with hydrocephalus at tertiary medical facility	125	9 (6.8%)
Wesonga et al., 2016 [22]	Uganda	Prospective cohort study	Institutional; patients with gastroschisis at a tertiary hospital	41	10 (24.4%)
Munjanja et al., 1987 [23]	Zimbabwe	Prospective descriptive observational study	Institutional; all patients delivered at Greater Harare Obstetric Unit	36,514	4,429 (12.1%)
**Total**	**11 studies, 6 countries**	**4 retrospective, 7 prospective observational studies**	**11 institutional**	**37,349**	**4,852 (13.0%)** **Range: 6.8 - 96.8%**
**Asia**					
Lu et al., 2011 [24]	China	Retrospective cross-sectional study	National; fetuses with NTDs	424	419 (98.8%)
Bhat et al., 2008 [25]	India	Retrospective descriptive observational study	Institutional; patients admitted to NICU* with CDH*	16	11 (68.8%)
Raman et al., 2015 [26]	India	Retrospective descriptive observational study	Institutional; symptomatic patients with congenital cystic lung lesions at tertiary care center	40	6 (15%)
Saha et al., 2009 [27]	India	Retrospective descriptive observational study	Institutional; all deliveries at rural medical college	7,365	6,682 (90.7%)
Sood et al., 1991 [28]	India	Retrospective descriptive observational study	Institutional; patients with NTDs at tertiary hospital	65	44 (67.7%)
Kazmi et al., 2006 [29]	Iran	Prospective descriptive observational study	Institutional; patients referred to tertiary center for myelomeningocele evaluation and management	140	136 (97.1%)
Samadirad et al., 2012 [30]	Iran	Retrospective descriptive observational study	Regional; fetuses with congenital anomalies	639	557 (87.2%)
Ho et al., 2006 [31]	Malaysia	Retrospective cohort study	Regional; births in Kinta District (253 cases with congenital anomalies and 506 control cases)	759	705 (92.9%)
Kitisomprayoonkul and Tongsong, 2001 [32]	Thailand	Prospective descriptive observational study	Institutional; patients with NTDs at tertiary hospital	46	42 (91.3%)
**Total**	**9 studies, 5 countries**	**7 retrospective, 2 prospective observational studies**	**6 institutional, 2 regional, 1 national**	**9,494**	**8,602 (90.6%)** **Range: 15 – 98.8%**
**North America**					
Johnson et al., 2011 [33]	Jamaica	Retrospective observational review	Institutional; patients with congenital anomalies at tertiary hospital	55	54 (98.2%)
**Total**	**1 study, 1 country**	**1 retrospective observational study**	**1 institutional**	**55**	**54 (98.2%)** **Range: N/A**

* CDH: congenital diaphragmatic hernia, CNS: central nervous system, NICU: neonatal intensive care unit, NTD: neural tube defects, PUV: posterior urethral valves.

Confidential: For Review Only

Table 3: Effectiveness of Antenatal Ultrasound

Author(s)	Study Location	Study Type	Study Population	Number of Women in Study	Number of Women Who Received Antenatal Diagnosis (%)
**Africa**					
de Paul Djientcheu et al., 2008 [13]	Cameroon	Retrospective descriptive observational study	Institutional; patients with NTDs* admitted to neonatology unit	27	8 (29.6%)
Sorri and Mesfin, 2015 [34]	Ethiopia	Retrospective cross-sectional study	Multi-center; patients with NTDs at two tertiary hospitals	177	127 (71.8%)
Abdur-Rahman et al., 2011 [14]	Nigeria	Retrospective descriptive observational study	Institutional; patients with abdominal wall defects at a tertiary health center in the North-Central geopolitical zone of Nigeria	56	1 (1.8%)
Adeleye et al., 2010 [15]	Nigeria	Prospective cross-sectional study	Institutional; patients presenting with major CNS* anomalies at tertiary hospital	43	6 (14%)
Adeleye and Joel-Medwase, 2015 [16]	Nigeria	Retrospective cross-sectional survey	Institutional; patients presenting with CNS anomalies at a neurosurgeon's practice	146	26 (17.8%)
Akinmoladun et al., 2015 [35]	Nigeria	Prospective descriptive observational study	Institutional; patients attending clinic for ultrasound screening	16	15 (93.8%)
Amadi and Eghwrudjakpor, 2013 [36]	Nigeria	Retrospective descriptive observational study	Institutional; all patients with encephalocele at tertiary hospital	17	5 (29.4%)
Bankole et al., 2012 [17]	Nigeria	Prospective descriptive observational study	Institutional; patients presenting with CNS anomalies at tertiary hospital	108	0 (0%)
Idowu and Olawehinmi, 2012 [18]	Nigeria	Prospective descriptive observational study	Institutional; patients presenting with NTDs at tertiary hospital	91	23 (25.3%)
Okafor et al., 2013 [19]	Nigeria	Prospective cohort study	Institutional; patients with PUV* at tertiary hospital	31	2 (6.5%)
Choopa et al., 2017 [37]	South Africa	Retrospective descriptive observational study	Institutional; patients with PUV at pediatric nephrology unit	60	10 (16.7%)
Sekabira and Hadley, 2009 [20]	South Africa	Retrospective descriptive observational study	Institutional; patients with gastroschisis at tertiary hospital	106	13 (12.3%)
Chanoufi et al., 2004 [38]	Tunisia	Retrospective case series (6 cases)	Institutional; cases of acardiac twins at maternity center	6	1 (16.7%)
Wesonga et al., 2016 [22]	Uganda	Prospective cohort study	Institutional; patients with gastroschisis at a tertiary hospital	41	1 (2.4%)
Munjanja et al., 1987 [23]	Zimbabwe	Prospective descriptive observational study	Institutional; patients with congenital anomalies at obstetrical unit	91	46 (50.5%)
**Total**	**15 studies, 7 countries**	**8 retrospective, 7 prospective observational studies**	**14 institutional, 1 multi-center**	**1,016**	**284 (28.0%)** **Range: 0 – 93.8%**
**Asia**					
Deng et al., 2014 [39]	China	Retrospective cross-sectional study	National; patients with omphalocele as reported in Chinese national birth defects monitoring network 1996-2006	827	322 (38.9%)
Hong et al., 2010 [40]	China	Retrospective cohort study	Multi-center; patients with gastroschisis	17	3 (17.6%)
Liao et al., 2016 [41]	China	Retrospective descriptive observational study	Institutional; patients with limb abnormalities at maternity and child health hospital	36	28 (77.8%)
Liu et al., 2017 [42]	China	Retrospective cross-sectional study	Institutional; patients with congenital anomalies at a tertiary hospital	233	71 (30.5%)
Lu et al., 2011 [24]	China	Retrospective cross-sectional study	National; patients with NTDs	424	361 (85.1%)
Shi et al., 2006 [43]	China	Retrospective descriptive observational study	Institutional; cases of conjoined twins at tertiary hospital	7	4 (57.1%)
Weng et al., 2016 [44]	China	Retrospective descriptive observational study	Institutional; patients with congenital choledochal cyst at specialty women's hospital	21	19 (90.5%)
Bhat et al., 2008 [25]	India	Retrospective descriptive observational study	Institutional; patients admitted to NICU* with CDH*	16	4 (25%)
Kumar et al., 2008 [45]	India	Retrospective descriptive observational study	Institutional; symptomatic patients with congenital bronchopulmonary anomalies	25	2 (8%)

Raman et al., 2015 [26]	India	Retrospective descriptive observational study	Institutional; symptomatic patients with congenital cystic lung lesions at tertiary care center	40	3 (7.5%)
Rattan et al., 2017 [46]	India	Retrospective descriptive observational study	Institutional; patients operated on for esophageal atresia and tracheoesophageal fistula at a tertiary care center	693	63 (9.1%)
Sanghvi et al., 1998 [47]	India	Prospective descriptive observational study	Institutional; patients with renal anomalies at tertiary center	125	65 (52%)
Sarin et al., 2012 [48]	India	Retrospective case series (18 cases)	Institutional; patients with duodenal webs at tertiary hospital in India	18	2 (11.1%)
Sharada et al., 2014 [49]	India	Retrospective descriptive observational study	Institutional; patients diagnosed with unilateral multicystic dysplastic kidney at tertiary hospital	47	34 (72.3%)
Singh et al., 2009 [50]	India	Retrospective descriptive observational study	Institutional; patients with unilateral multicystic dysplastic kidney at tertiary center	22	12 (54.5%)
Solanki et al., 2013 [51]	India	Retrospective case series (6 cases)	Institutional; patients diagnosed with crossed fused renal ectopia at tertiary hospital	6	1 (16.7%)
Kazmi et al., 2006 [29]	Iran	Prospective descriptive observational study	Institutional; patients referred to tertiary center for myelomeningocele evaluation and management	136	33 (24.3%)
Mirshemirani et al., 2013 [52]	Iran	Retrospective descriptive observational study	Institutional; patients treated for PUV at a tertiary hospital	98	20 (20.4%)
Shahkar et al., 2016 [53]	Iran	Retrospective descriptive observational study	Institutional; patients with congenital pulmonary mass at a tertiary hospital	47	10 (21.3%)
Ho et al., 2006 [31]	Malaysia	Retrospective cohort study	Regional; births in Kinta District (253 cases with congenital anomalies and 506 control cases)	252	37 (14.7%)
Munim et al., 2013 [54]	Pakistan	Retrospective cohort study	Institutional; patients with diaphragmatic hernia at tertiary hospital	65	41 (63.1%)
Kitisomprayoonkul and Tongsong, 2001 [32]	Thailand	Prospective descriptive observational study	Institutional; patients with NTDs at tertiary hospital	42	42 (100%)
Pitukkijronnakorn et al., 2009 [55]	Thailand	Prospective cross-sectional study	Institutional; patients diagnosed with major congenital anomalies at tertiary hospital	316	144 (45.6%)
Srisupundit et al., 2006 [56]	Thailand	Prospective descriptive observational study	Institutional; patients undergoing antenatal ultrasound at a university teaching hospital in Chiang Mai	34	24 (70.6%)
**Total**	**24 studies, 6 countries**	**19 retrospective, 5 prospective observational studies**	**20 institutional, 1 multi-center, 1 regional, 2 national**	**3,547**	**1,345 (37.9%)**<br>**Range: 8 – 100%**
**Europe**					
Iliescu et al., 2013 [57]	Romania	Prospective descriptive observational study	Multi-center; patients at two institutions with major congenital anomalies	76	74 (97.4%)
Ognean et al., 2017 [58]	Romania	Retrospective case series (7 cases)	Institutional; patients with esophageal atresia at a tertiary center	7	0 (0%)
Tarca and Aprodu, 2014 [59]	Romania	Retrospective descriptive observational study	Institutional; patients with omphalocele at tertiary hospital	105	14 (13.3%)
Tarca and Aprodu, 2013 [60]	Romania	Retrospective descriptive observational study	Institutional; patients with gastroschisis at tertiary hospital	54	9 (16.7%)
Tarca et al., 2015 [61]	Romania	Retrospective descriptive observational study	Institutional; patients with gastroschisis at tertiary hospital	114	13 (11.4%)
Tudorache et al., 2014 [62]	Romania	Retrospective descriptive observational study	Institutional; patients with cases of left-sided CDH at tertiary hospital	21	11 (52.4%)
Postoev et al., 2015 [63]	Russia	Retrospective cross-sectional study	Regional; patients with congenital anomalies in the Kola Peninsula (data from two birth defect registries)	232	81 (34.9%)
Aygun et al., 2013 [64]	Turkey	Retrospective descriptive observational study	Institutional; patients with NTDs at tertiary hospital	100	72 (72%)
Dane et al., 2007 [65]	Turkey	Prospective descriptive observational study	Institutional; fetuses with incurable congenital anomalies and curable severe congenital anomalies at a training and research hospital	24	23 (95.8%)
Orgul et al., 2018 [66]	Turkey	Retrospective descriptive observational study	Institutional; patients with gastrointestinal tract malformations at a university children's hospital	56	34 (60.7%)

Confidential: For Review Only

Oztekin et al., 2009 [67]	Turkey	Prospective descriptive observational study	Institutional; patients with a major structural congenital anomaly at an obstetrics and gynecology teaching hospital	21	19 (90.5%)
Sahinoglu et al., 2007 [68]	Turkey	Retrospective case series (6 cases)	Institutional; patients with limb body wall complex at a women and children's research hospital	6	5 (83.3%)
Tabel et al., 2010 [69]	Turkey	Prospective descriptive observational study	Institutional; patients with kidney or urinary tract anomalies at a university hospital	76	32 (42.1%)
Taskapilioglu et al., 2014 [70]	Turkey	Retrospective descriptive observational study	Institutional; patients with open spina bifida at tertiary center	78	26 (33.3%)
**Total**	**14 studies, 3 countries**	**10 prospective, 4 retrospective observational studies**	**12 institutional, 1 multi-center, 1 regional**	**970**	**413 (42.6%)** **Range: 0 – 97.4%**
**North America**					
Johnson et al., 2012 [33]	Jamaica	Retrospective descriptive observational study	Institutional; patients with congenital anomalies at tertiary hospital	57	44 (77.2%)
**Total**	**1 study, 1 country**	**1 retrospective observational study**	**1 institutional**	**57**	**44 (77.2%)** **Range: N/A**
**South America**					
Campana et al., 2010 [71]	Argentina, Brazil, Chile, and Venezuela	Prospective descriptive observational study	Multi-country; patients with congenital anomalies in 18 Latin American hospitals	812	457 (56.3%)
Germani et al., 2011 [72]	Argentina	Retrospective descriptive observational study	Institutional; patients with choledochal cyst at a private hospital	12	4 (33.3%)
Wyszynski et al., 2003 [73]	Argentina	Survey	Institutional; patients with nonsyndromic oral cleft (collected from parents' survey data)	165	7 (4.2%)
Carvalho et al., 2002 [74]	Brazil	Prospective cohort study	Institutional; patients with major congenital anomalies at a tertiary hospital	130	93 (71.5%)
Luiza et al., 2013 [75]	Brazil	Retrospective cross-sectional study	Institutional; patients with orofacial cleft at a specialized society attending to cleft patients	165	7 (4.2%)
Tannuri et al., 2011 [76]	Brazil	Retrospective descriptive observational study	Multi-center; patients with gastroschisis at three tertiary centers	163	134 (82.2%)
Vilela et al., 2001 [77]	Brazil	Retrospective cross-sectional study	Institutional; patients with gastroschisis at a tertiary hospital	31	10 (32.3%)
Correa et al., 2014 [78]	Colombia	Retrospective case-control study	City-wide; data from Bogota Congenital Malformations Surveillance Program	167	82 (49.1%)
de Rovetto et al., 2010 [79]	Colombia	Retrospective descriptive observational study	City-wide; patients with congenital renal agenesis at centers in Cali, Colombia	38	8 (21.1%)
Rosselli et al., 2015 [80]	Colombia	Retrospective descriptive observational study	City-wide; patients with congenital talipes equinovarus in Bogota, Colombia	178	61 (34.3%)
Saldarriaga et al., 2014 [81]	Colombia	Retrospective cross-sectional study	City-wide; patients with congenital anomalies diagnosable by antenatal ultrasound in NICUs of Cali, Colombia	217	117 (53.9%)
**Total**	**11 studies, 5 countries**	**8 retrospective, 2 prospective observational studies, 1 survey**	**5 institutional, 1 multi-center, 4 city-wide, 1 multi-country**	**2,078**	**980 (47.2%)** **Range: 4.2 – 82.2%**

* CDH: congenital diaphragmatic hernia, CNS: central nervous system, NICU: neonatal intensive care unit, NTD: neural tube defects, PUV: posterior urethral valves.

Table 4: Training of Personnel Performing Ultrasound Examination

Author(s)	Study Location	Number of Anomalies Detected (%)	Information about Training of Personnel Performing Antenatal Ultrasound Examinations
**Africa**			
Adeleye et al., 2010 [15]	Nigeria	6/43 (14%)	Radiologists performed 5% of cases; medical doctors performed 11%; unknown training/status performed 84% of cases
Adeleye and Joel-Medewase, 2015 [16]	Nigeria	26/146 (17.8%)	22% of ultrasounds performed by a radiologist; sonographers in rest of the cases were personnel with unknown training; authors noted that prenatal diagnosis was significantly more likely in cases where sonographer was certified radiologist
Akinmoladun et al., 2015 [35]	Nigeria	15/16 (93.8%)	A consultant radiologist trained in fetal anomaly scanning performed all the scans (the authors note that this radiologist received extensive training at a renowned center in the UK)

Confidential: For Review Only

Idowu and Olawehinmi, 2012 [18]	Nigeria	23/91 (25.3%)	Authors noted that low diagnosis "may be due to the high prevalence of the test being done by non-specialist (untrained radiologist) in our environment"
Wesonga et al., 2016 [22]	Uganda	1/41 (2.4%)	Performed by ultrasound technicians holding a diploma; no further information about diploma
**Asia**			
Liao et al., 2016 [41]	China	28/36 (77.8%)	Ten certified physicians participated in the study protocol, each of whom has more than 5 years of experience in fetal sonography
Xie et al., 2009 [82]	China	Not specified	2 sonographers - 1 with 10 years of experience in obstetric sonography and the other with 22 years of experience
Sanghvi et al., 1998 [47]	India	65/125 (52%)	Performed by "experienced sonologists"
Ghavami and Abedinzadeh, 2011 [83]	Iran	Not specified	Performed by “two expert operators"
Pitukkijronnakorn et al., 2009 [55]	Thailand	144/316 (45.6%)	All scans were performed by an obstetrician who was trained as a level one ultrasonography; in cases of uncertain abnormal findings, the women were reviewed by a level two obstetrician with repeated scans
**Europe**			
Iliescu et al., 2013 [57]	Romania	74/76 (97.4%)	Scans performed by obstetricians specializing in prenatal diagnosis (including the anomaly scan and echocardiography) who had held accreditation for the 11 – 14-week assessment for at least 5 years prior to the start of the study period
Dane et al., 2007 [65]	Turkey	23/24 (95.8%)	2 operators with approximately 6 years & 2 years of experience in gestational ultrasound scanning
Kutuk et al., 2017 [84]	Turkey	Not specified	All ultrasound scans performed by “two experienced maternal-fetal specialists”
Oztekin et al., 2009 [67]	Turkey	19/21 (90.5%)	All scans performed by the same experienced radiologist
**North America**			
Johnson et al., 2011 [33]	Jamaica	44/57 (77.2%)	8 OB/GYN residents in training for at least 2 years

Table 5: Morbidity and Mortality Outcomes

Author(s)	Study Location	Patient Population	Mortality with Antenatal Diagnosis	Mortality without Antenatal Diagnosis	Morbidity with Antenatal Diagnosis	Morbidity without Antenatal Diagnosis
**Asia**						
Bhat et al., 2008 [25]	India	Institutional; patients with CDH*	4/4 (100%)	3/12 (25%)	N/A	4/9 (44.4%)
**Europe**						
Savran et al., 2015 [85]	Turkey	Institutional; patients with duodenal atresia	0/9 (0%)	0/6 (0%)	0/9 (0%)	1/6 (16.7%)
**North America**						
Johnson et al., 2011 [33]	Jamaica	Institutional; patients with congenital anomalies	19/44 (43.2%)	5/13 (38.5%)	11/29 (37.9%)	9/12 (75%)
**South America**						
Vilela et al., 2001 [77]	Brazil	Institutional; patients with gastroschisis	2/10 (20%)	14/21 (66.7%)	Not specified	Not specified

*CDH: congenital diaphragmatic hernia

Table 6: Termination Rates

Author(s)	Study Location	Study Type	Study Population	Number of Fetuses	Number of Fetuses Terminated (%)
**Africa**					
de Paul Djientcheu et al., 2008 [13]	Cameroon	Retrospective descriptive observational study	Institutional; patients with NTDs*	8	0 (0%)
Shalaby et al., 2016 [86]	Egypt	Retrospective cross-sectional study	Institutional; Patients with urinary anomalies	41	11 (26.8%)
Sorri and Mesfin, 2015 [34]	Ethiopia	Retrospective cross-sectional study	Multi-center; patients with NTDs at two tertiary hospitals	177	13 (7.3%)
Akinmoladun et al., 2015 [35]	Nigeria	Prospective descriptive observational study	Institutional; Patients with lethal congenital anomalies	5	4 (80%)

Confidential: For Review Only

**Total**	**4 studies, 4 countries**	**3 retrospective, 1 prospective observational studies**	**3 institutional, 1 multi-center**	**231**	**28 (12.1%)** **Range: 0 – 80%**
**Asia**					
Li et al., 2006 [87]	China	Retrospective descriptive observational survey	Regional; patients with NTDs	160	72 (45.0%)
Lu et al., 2011 [24]	China	Retrospective cross-sectional study	National (data from 20 counties); patients with NTDs	361	355 (98.3%)
Xie et al., 2009 [82]	China	Retrospective descriptive observational study	Institutional; patients with bronchopulmonary sequestration	22	8 (36.4%)
Zhang, 2014 [88]	China	Retrospective descriptive observational study	Institutional; patients with pulmonary sequestration	68	2 (2.9%)
Kashyap et al., 2015 [89]	India	Retrospective descriptive observational study	Institutional; patients with lethal congenital anomalies detected prior to 20 weeks of gestation	103	80 (77.7%)
Kumar et al., 2012 [90]	India	Prospective cohort study	Institutional; patients with severe renal anomalies	55	9 (16.4%)
Kumar et al., 2014 [91]	India	Prospective descriptive observational study	Institutional; patients with renal anomalies	136	12 (8.8%)
Sanghvi, et al., 1998 [47]	India	Prospective descriptive observational study	Institutional; patients with lethal renal anomalies	7	2 (28.6%)
Samadirad et al., 2012 [30]	Iran	Retrospective descriptive observational study	Regional; patients with congenital anomalies	603	201 (33.3%)
Munim et al., 2013 [54]	Pakistan	Retrospective cohort study	Institutional; patients with diaphragmatic hernia	41	6 (14.6%)
Hsieh et al., 2000 [92]	Taiwan	Retrospective descriptive observational study	Institutional; patients with CDH*	31	11 (35.5%)
Jaruratanasirikul et al., 2014 [93]	Thailand	Retrospective cross-sectional study	Regional; patients with NTDs	28	12 (42.9%)
Pitukkijronnakorn et al., 2009 [55]	Thailand	Retrospective cross-sectional study	Institutional; patients with congenital anomalies	316	87 (27.5%)
Gammeltoft et al., 2008 [94]	Vietnam	Ethnographic study	Institutional; patients with congenital anomalies	30	17 (56.7%)
**Total**	**14 studies, 7 countries**	**10 retrospective, 3 prospective observational studies; 1 ethnographic study**	**10 institutional, 3 regional, 1 national**	**1,961**	**874 (44.6%)** **Range: 2.9 – 98.3%**
**Europe**					
Tudorache et al., 2014 [62]	Romania	Retrospective descriptive observational study	Institutional; patients with severe CDH diagnosed in the second trimester of pregnancy	6	4 (66.7%)
Aygun et al., 2013 [64]	Turkey	Retrospective descriptive observational study	Institutional; patients with NTDs	72	0 (0%)
Oztarhan et al., 2010 [95]	Turkey	Retrospective cohort study	Institutional; patients with lethal congenital anomalies	1,906	640 (33.6%)
Sahinoglu et al., 2007 [68]	Turkey	Retrospective case series (6 cases)	Institutional; patients with body wall complex	6	4 (66.7%)
**Total**	**4 studies, 2 countries**	**4 retrospective observational studies**	**4 institutional**	**1,990**	**648 (32.6%)** **Range: 0 – 66.7%**
**North America**					
Johnson et al., 2012 [33]	Jamaica	Retrospective descriptive observational study	Institutional; patients with congenital anomalies	44	10 (22.7%)
**Total**	**1 study, 1 county**	**1 retrospective observational study**	**1 institutional**	**44**	**10 (22.7%)** **Range: N/A**
**South America**					
Brizot et al., [96]	Brazil	Retrospective descriptive observational study	Institutional; pairs of conjoined twins in which surgical separation was impossible and the condition lethal	36	30 (83.3%)
Pelizzari et al., 2015 [97]	Brazil	Retrospective cohort study	Institutional; patients with anencephaly	29	12 (41.4%)
**Total**	**2 studies, 1 country**	**2 retrospective observational studies**	**2 institutional**	**65**	**42 (64.6%)** **Range: 41.4 – 83.3%**

* CDH: congenital diaphragmatic hernia, NTD: neural tube defects.

Confidential: For Review Only

Referral Rates for Further Antenatal Care and Management Planning

No studies addressed this issue.

Table 7: Policy Data

Author(s)	Study Location	Policy Data about Antenatal Screening and/or Termination Legislation
**Africa**		
Oloyede and Oyedele, 2008 [98]	Nigeria	In Nigeria, the two existing pregnancy termination laws are restrictive in nature. However, termination is often done when a fetus is malformed on the grounds of preserving the mental health of the women.
**Asia**		
Acharya et al., 2013 [99]	India	India has no definite policy for the ultrasound screening for fetal abnormalities and antenatal diagnostic techniques. The law in India says that those who meet the criteria of the PCPNDT* Act can perform an ultrasound scan and they must be sufficiently trained & registered with the proper authority.
Neogi, 2010 [100]	India	Abortion was legalized in India in 1971 under the Medical Termination of Pregnancy Act. It permits abortion by 1 doctor before 12 weeks of gestation but if the duration of pregnancy is more than 12 weeks but less than 20 weeks, then the opinion of 2 medical practitioners is necessary to terminate the pregnancy.
Phadke et al., 2011 [101]	India	In India, the Medical Termination of Pregnancy Act of 1971 (The MTP Act, No. 34 of 1971) does not allow pregnancy termination on grounds of fetal abnormality after 20 weeks of gestation.
Ranji and Dykes, 2012 [102]	Iran	According to the regulations of the Iranian Ministry of Health, ultrasound examinations during pregnancy must be carried out by radiologists.
Arawi and Nassar, 2011 [103]	Lebanon	Lebanese law stipulates that pregnancy termination is forbidden except when the pregnancy endangers the health of the mother and only after consulting with two physicians.
Senanayake and de Silva, 2001 [104]	Sri Lanka	In Sri Lanka, it is illegal to terminate a pregnancy even in cases of early diagnosis (11-14 weeks of gestation).
**Europe**		
Hostiuc et al., 2013 [105]	Romania	According to Romanian law, abortion over 14 weeks is only allowed in cases of severe congenital defects and pregnancies that threaten the life of the mother.
Oztarhan et al., 2010 [95]	Turkey	Turkish law authorizes pregnancy termination voluntarily until 10 weeks in unwanted pregnancies and at any gestational age for medical indications that are considered potentially life-threatening to the mother or fetus. The legal process requires one obstetrician and one physician to agree that pregnancy termination is valid for a medical reason.
**North America**		
Lisker et al., 2006 [106]	Mexico	Pregnancy termination is illegal in most Mexican States, except in the case of rape or if the mother's life is at risk by the continuation of pregnancy. In Mexico City and 12 of the 31 states, the presence of a severe congenital anomalies has become a justification for the legal termination of pregnancy.
**South America**		
Groisman et al., 2013 [107]	Argentina	According to the Argentinian criminal code, termination of pregnancy is illegal unless the pregnancy is threat to woman's life or pregnancy is consequence of rape of a mentally retarded woman. In the city of Buenos Aires, it is legal to induce labor after 24 weeks of gestational age in case of anencephaly and other lethal conditions.
Benute et al., 2012 [108]	Brazil	Brazilian law does not include lethal fetal malformation as an indication for pregnancy termination, however, many couples ask a court for permission to terminate a pregnancy on the grounds that it is the option which creates less suffering.
Mirlesse and Ville, 2013 [109]	Brazil	Ultrasound is not explicitly recommended by Brazilian authorities. Brazilian legislation considers termination of pregnancy to be a crime (except in cases of rape or pregnancies which risk the mother's life). However, for lethal fetal malformations, it is possible to apply to the courts for an exceptional authorization to abort. These requests require a medical referral center to perform an ultrasound and prepare a very detailed file.

*PCPNDT: Pre-Conception and Pre-Natal Diagnostic Techniques Act

Policy Assessment

WHO guidelines recommend the need for one antenatal ultrasound scan prior to 24 weeks gestation [110]. Studies suggest that the ideal detection window for structural congenital anomalies is 19-21 weeks of gestation [111]. At this point, it is possible to detect most structural congenital anomalies and is within the legal termination timeframe for many countries. Of note, many of the congenital anomalies detected antenatally in this review were not diagnosed until after 24 weeks gestation. This may be explained by the timing of the first antenatal ultrasound and/or the level of ultrasonographer training. The WHO recommends that ultrasound trainees receive at least 3-6 months of training, culminating in 300-500 ultrasound examinations [112]. A recent study found that the majority of ultrasound providers in LMICs do not have the minimum training as set by the WHO [113]. Hence, many ultrasound practitioners in LMICs may not have the skills to accurately detect congenital anomalies.

Discussion

The findings of this study suggest that there is significant variation in the percentage of women receiving antenatal ultrasound scans, ranging from 6.8% in a Tanzanian study to 98.8% in a study from China. The data suggests a particularly low rate of women receiving antenatal ultrasound in Africa, with a mean of 13.0% compared to 90.6% in Asia and 98.2% in North America. However, even these statistics may be an overestimate considering that the majority of included studies were undertaken at tertiary facilities. To fully understand what percentage of women receive antenatal ultrasound, further studies must be conducted on a larger scale, regionally and nationally rather than single institutions. Research must also address the availability and accessibility of antenatal ultrasound and the barriers to receiving a scan.

The results further show that detection rates varied widely across studies, from 0% to 100%, with little correlation according to geographical region or type of anomaly. In Africa, the average detection rate was 28.0%, which is low compared to other LMICs, with 37.9% in Asia, 42.6% in Europe, 47.2% in South America, and 77.2% in North America. This may be because ultrasound providers did not specifically screen for congenital anomalies. Currently, many women in LMICs receive antenatal ultrasound examinations for the assessment of pregnancy progress, such as to determine the gestational age, the sex of the baby, and to hear the heartbeat, rather than to detect anomalies. This is in contrast to HICs where the majority of women receive an anomaly scan around 20 weeks gestation [111]. Another possible reason for the low detection may be the training level of the ultrasound provider. In the data presented, there appears to be a trend between higher levels of training and higher detection rates; this warrants further investigation.

Only four studies produced any data comparing the morbidity and mortality outcomes between neonates with an antenatal diagnosis versus neonates with a postnatal diagnosis. In the study that addressed gastroschisis, outcomes were more favorable for neonates who had received an antenatal diagnosis compared to those who had not (20% vs. 66.7% mortality). This was not the case for the study which addressed congenital diaphragmatic hernia (CDH), however this may reflect that more severe forms of anomalies are easier to detect antenatally. Termination rates were highly varied: 12.1% Africa, 22.7% North American, 32.6% Europe, 44.6% Asia and 64.6% South America (range 0% to 98.3%). Only one study from Africa evaluated termination rates for lethal anomalies and had just five participants. Thus, it is difficult to compare the termination rate of lethal anomalies with other regions, which contain such data. It must also be noted that termination of pregnancy remains a highly sensitive topic in many communities, which is reflected in the variation of policies across the globe.

The First Look Study is an important randomized controlled trial which assessed the use of antenatal ultrasound in the Democratic Republic of the Congo, Guatemala, Kenya, Pakistan, and Zambia [114]. Their findings showed that although 95% of women in the intervention group received antenatal ultrasound scans (compared to 43% in the control group) and detection rates improved, hospital delivery did not increase for complicated pregnancies and thus there was no resultant improvement in neonatal mortality. This suggests that there may have been an issue with the referral chains and/or access to such services in the countries where the study was conducted. In an additional survey from the First Look Study, barriers to referral attendance were

Confidential: For Review Only

cost, distance, and lack of transportation [115]. For women who did attend referral, barriers included not being connected to the correct provider and being told to return at a later time [115]. The authors of the First Look study conclude that without the improvement of subsequent care, antenatal ultrasound offers a limited impact [114]. Hence, to reduce neonatal morbidity and mortality, detection of an anomaly must be followed by referral for antenatal counselling and delivery at a tertiary center which can provide the necessary surgical care at birth where required. It is also necessary to offer termination for conditions which are incompatible with life, where culturally acceptable.

Thus, it is vital to further investigate barriers to accessing delivery in a pediatric surgery tertiary care center once a congenital anomaly has been diagnosed and ways to address these barriers. Future studies must also investigate the effects of both antenatal diagnosis and delivery in tertiary pediatric surgery center on morbidity and mortality outcomes in the LMIC setting. The recently completed Global PaedSurg study may provide further global data on the antenatal diagnosis of common gastrointestinal congenital anomalies and the effect on morbidity and mortality outcomes [116]. Finally, it is essential to further research the level of training acquired by antenatal ultrasound providers and the effect this has on detection rates in order to determine what level of training is necessary to provide an effective scan.

To address some of these issues, there is a need for global collaboration. This collaboration must include members of multi-disciplinary backgrounds, including policymakers, obstetricians, neonatologists, pediatric surgeons, midwives, and allied professionals. The Global Initiative for Children's Surgery (GICS) is a multidisciplinary collaborative whose aim is to improve health outcomes for children requiring surgery in LMICs [117]. This initiative connects the expertise of providers in LMICs and HICs and is committed to expanding the representation and leadership of stakeholders in LMICs. GICS has recently created a congenital anomalies working group, which is planning some of the following projects: 1) To produce guidelines on how to diagnose structural congenital anomalies via antenatal ultrasound; 2) To produce referral and management guidelines following an antenatal diagnosis; and 3) To produce information sheets that can be translated into various languages for parents that contain details about common congenital anomalies. Global collaboration must also extend to the level of the WHO and the Ministries of Health to ensure that recommendations are implemented into practice.

If these steps are taken, improvements in neonatal health outcomes may be realized, as seen in HICs. Early detection and immediate surgical intervention of congenital anomalies, such as gastroschisis, has been effective in significantly reducing neonatal mortality in HICs [5]. The mortality of gastroschisis has significantly improved in HICs over a period of 50 years, to less than 5% today [5]. This can be attributed to improvements in accurately diagnosing gastroschisis antenatally, monitoring the fetus for complications, and planning for delivery at a facility with pediatric surgeons available [118]. Similar trends have been seen for other congenital anomalies in HICs such as intestinal atresia, congenital diaphragmatic hernia, omphalocele, esophageal atresia and posterior urethral values. By understanding the current role of antenatal ultrasound in LMICs and the barriers to detection, referral, and management of structural congenital anomalies, appropriate interventions can be implemented to help improve outcomes.

Confidential: For Review Only

Although this systematic review provides useful data, it is also important to note a few of the limitations of the study. First, only articles in English were included in this systematic review, which may exclude other relevant studies. This study used four electronic databases for the search. The expansion of search databases to include African Journals Online, Sciello, and Regional WHO's African Index Medicus may have provided other studies from LMICs that were not indexed in the search engines used. It is vital to include these databases in future research focusing on LMICs. Furthermore, it is important to note that antenatal ultrasound has further diagnostic capabilities, such as detecting abnormal growth or improper placental position and this review only focused on the detection of structural congenital anomalies. Further studies could also include other uses of antenatal ultrasound for improving neonatal and indeed maternal health outcomes. Finally, the policy data in this study represents what was accurate when the studies were published. Some of the policy data may now be out-of-date.

Conclusion

The data from this review suggests that the percentage of women in LMICs who receive an antenatal ultrasound examination varies considerably and is particularly low in sub-Saharan African countries. Even when antenatal ultrasound scans are performed, accurate detection rates are often very low. The level of training (and the type of training) of the sonographer may be indicative of the accuracy of diagnosis. Only four studies delineated the morbidity and mortality outcomes amongst neonates with an antenatal diagnosis compared to postnatal diagnosis. Hence, although the benefits of antenatal ultrasound are widely documented in HICs, data is severely lacking in LMICs. It is clear that the use of antenatal ultrasound in LMICs is not maximized to its highest potential.

Acknowledgements

We would like to thank the library services at King's College London for help with the systematic review process.

Contributors

NW conceived the idea for this study. NW, AK, and SG devised the study design. SG, SSB, NAA, LIN, MTB, and NW performed the literature review. SG drafted the manuscript with significant contributions from NW and AK. All authors reviewed and approval the final submitted manuscript.

Funding

NW receives funding from the Wellcome Trust to undertake a Clinical PhD in Global Health at King's Centre for Global Health and Health Partnerships, King's College London (Funder Reference: 203905/Z/16/Z).

Competing Interests

Confidential: For Review Only

The authors have no competing interests to declare.

References

1. Sitkin, N.A., et al., *Congenital anomalies in low- and middle-income countries: the unborn child of global surgery.* World J Surg, 2015. **39**(1): p. 36-40.
2. Farmer, D., et al., *Surgical Interventions for Congenital Anomalies*, in *Essential Surgery: Disease Control Priorities, Third Edition (Volume 1)*, H.T. Debas, et al., Editors. 2015: Washington (DC).
3. WHO. *WHO Fact Sheets - Congenital Anomalies* 2015 May 12, 2018]; Available from: http://www.who.int/news-room/fact-sheets/detail/congenital-anomalies.
4. Badrinath, R., et al., *Outcomes and unmet need for neonatal surgery in a resource-limited environment: estimates of global health disparities from Kampala, Uganda.* J Pediatr Surg, 2014. **49**(12): p. 1825-30.
5. Bradnock, T.J., et al., *Gastroschisis: one year outcomes from national cohort study.* BMJ, 2011. **343**: p. d6749.
6. Wright, N.J., A. Zani, and N. Ade-Ajayi, *Epidemiology, management and outcome of gastroschisis in Sub-Saharan Africa: Results of an international survey.* Afr J Paediatr Surg, 2015. **12**(1): p. 1-6.
7. Shah, S., et al., *Perceived barriers in the use of ultrasound in developing countries.* Crit Ultrasound J, 2015. **7**(1): p. 28.
8. UN. *Goal 3: Ensure healthy lives and promote well-being for all at all ages*. Sustainable Development Goals 2015; Available from: https://www.un.org/sustainabledevelopment/health/.
9. WHO, *Sixty-Third World Health Assemby Report on Birth Defects*. 2010, World Health Organization: Geneva, Switzerland.
10. Liberati, A., et al., *The PRISMA statement for reporting systematic reviews and meta-analyses of studies that evaluate health care interventions: explanation and elaboration.* J Clin Epidemiol, 2009. **62**(10): p. e1-34.
11. Moher, D., et al., *Preferred reporting items for systematic reviews and meta-analyses: the PRISMA Statement.* Open Med, 2009. **3**(3): p. e123-30.
12. Goley, S., et al., *Investigating the use of ultrasonography for the antenatal diagnosis of structural congenital anomalies in low-income and middle-income countries: systematic review protocol* BMJ Paediatrics Open, 2019. **3**(1).
13. de Paul Djientcheu, V., et al., *Management of neural tube defects in a Sub-Saharan African country: the situation in Yaounde, Cameroon.* Journal of the Neurological Sciences, 2008. **275**(1-2): p. 29-32.
14. Abdur-Rahman, L.O., N.A. Abdulrasheed, and J.O. Adeniran, *Challenges and outcomes of management of anterior abdominal wall defects in a Nigerian tertiary hospital.* Afr J Paediatr Surg, 2011. **8**(2): p. 159-63.
15. Adeleye, A.O., M.D. Dairo, and K.G. Olowookere, *Central nervous system congenital malformations in a developing country: issues and challenges against their prevention.* Childs Nervous System, 2010. **26**(7): p. 919-24.
16. Adeleye, A.O. and V.I. Joel-Medewase, *Awareness and uptake of measures for preventing CNS birth defects among mothers of affected children in a sub-Saharan African neurosurgeon's practice.* Childs Nerv Syst, 2015. **31**(12): p. 2311-7.

Confidential: For Review Only

17. Bankole, O.B., S.O. Arigbabu, and O.O. Kanu, *Spinal neural tube defects in Lagos University Teaching Hospital, Nigeria.* Nigerian Quarterly Journal of Hospital Medicine, 2012. **22**(1): p. 22-4.
18. Idowu, O.E. and O.S. Olawehinmi, *Surgical congenital central nervous system anomalies in a tropical teaching hospital.* British Journal of Neurosurgery, 2012. **26**(5): p. 726-9.
19. Okafor, H.U., S.O. Ekenze, and S.N. Uwaezuoke, *Posterior urethral valves: determinants of outcome in a developing country.* J Paediatr Child Health, 2013. **49**(2): p. 115-9.
20. Sekabira, J. and G.P. Hadley, *Gastroschisis: a third world perspective.* Pediatric Surgery International, 2009. **25**(4): p. 327-9.
21. Santos, M.M., et al., *Infant hydrocephalus in sub-Saharan Africa: the reality on the Tanzanian side of the lake.* J Neurosurg Pediatr, 2017. **20**(5): p. 423-431.
22. Wesonga, A.S., et al., *Gastroschisis in Uganda: Opportunities for improved survival.* Journal of Pediatric Surgery, 2016. **51**(11): p. 1772-1777.
23. Munjanja, S.P., et al., *Audit of ultrasound scanning: antenatal diagnosis of congenital abnormalities in Harare, Zimbabwe.* East African Medical Journal, 1987. **64**(9): p. 601-5.
24. Lu, Q.B., et al., *Investigation of ultrasound screening efficiency for neural tube defects during pregnancy in rural areas of China.* Public Health, 2011. **125**(9): p. 639-44.
25. Bhat, Y.R., V. Kumar, and A. Rao, *Congenital diaphragmatic hernia in a developing country.* Singapore Med J, 2008. **49**(9): p. 715-8.
26. Raman, V.S., et al., *Congenital cystic lesions of the lungs: The perils of misdiagnosis - A single-center experience.* Lung India, 2015. **32**(2): p. 116-8.
27. Saha, A., et al., *Antenatal detection of renal malformations.* Indian Pediatrics, 2009. **46**(4): p. 346-8.
28. Sood, M., et al., *Neural tubal defects in an east Delhi hospital.* Indian Journal of Pediatrics, 1991. **58**(3): p. 363-5.
29. Kazmi, S.S., et al., *The prenatal ultrasonographic detection of myelomeningocele in patients referred to Children's Hospital Medical Center: a cross sectional study.* Reprod Health, 2006. **3**: p. 6.
30. Samadirad, B., et al., *Congenital anomalies and termination of pregnancy in Iran.* Journal of Pregnancy, 2012. **2012**: p. 574513.
31. Ho, J.J., M.K. Thong, and N.K. Nurani, *Prenatal detection of birth defects in a Malaysian population: estimation of the influence of termination of pregnancy on birth prevalence in a developing country.* Australian & New Zealand Journal of Obstetrics & Gynaecology, 2006. **46**(1): p. 55-7.
32. Kitisomprayoonkul, N. and T. Tongsong, *Neural tube defects: a different pattern in northern Thai population.* Journal of the Medical Association of Thailand, 2001. **84**(4): p. 483-8.
33. Johnson, N., et al., *Congenital abnormalities at a tertiary center in Jamaica: an 18-month maternal-fetal medicine experience.* Journal of Maternal-Fetal & Neonatal Medicine, 2012. **25**(6): p. 687-91.
34. Sorri, G. and E. Mesfin, *Patterns of Neural Tube Defects at Two Teaching Hospitals in Addis Ababa, Ethiopia a Three Years Retrospective Study.* Ethiopian Medical Journal, 2015. **53**(3): p. 119-26.

Confidential: For Review Only

35. Akinmoladun, J.A., et al., *Routine prenatal ultrasound anomaly screening program in a Nigerian university hospital: Redefining obstetrics practice in a developing African country.* Nigerian Medical Journal, 2015. **56**(4): p. 263-7.
36. Amadi, C.E. and P.O. Eghwrudjakpor, *The pattern of distribution of encephalocele in University of Port Harcourt Teaching Hospital--a three year experience.* Niger J Med, 2013. **22**(1): p. 19-23.
37. Choopa, M.S., S.O. Manda, and G. Van Biljon, *Diagnosis, management and outcome of posterior urethral valves in children at a tertiary centre in South Africa.* Pediatric Nephrology, 2017. **32 (9)**: p. 1706.
38. Chanoufi, M.B., et al., *Clinical and anatomic features of acardiac twins.* Medical Principles & Practice, 2004. **13**(6): p. 375-9.
39. Deng, K., et al., *Perinatal mortality in pregnancies with omphalocele: data from the Chinese national birth defects monitoring network, 1996-2006.* BMC Pediatrics, 2014. **14**: p. 160.
40. Hong, L., et al., *Modified silo technique-An easy and effective method to improve the survival rate of neonates with gastroschisis in Shanghai.* European Journal of Obstetrics, Gynecology, & Reproductive Biology, 2010. **148**(1): p. 31-4.
41. Liao, Y.M., et al., *Routine screening for fetal limb abnormalities in the first trimester.* Prenatal Diagnosis, 2016. **36**(2): p. 117-26.
42. Liu, S., Q. Wu, and Z. Chen, *Fetal anomalies detection in China by screening with ultrasound.* Biomedical Research (India), 2017. **28**(11): p. 4891-4896.
43. Shi, C.R., et al., *Surgical management to conjoined twins in Shanghai area.* Pediatr Surg Int, 2006. **22**(10): p. 791-5.
44. Weng, R., et al., *Prenatal diagnosis and prognosis assessment of congenital choledochal cyst in 21 cases.* J Obstet Gynaecol, 2016. **36**(3): p. 324-7.
45. Kumar, B., L.D. Agrawal, and S.B. Sharma, *Congenital bronchopulmonary malformations: A single-center experience and a review of literature.* Annals of Thoracic Medicine, 2008. **3**(4): p. 135-139.
46. Rattan, K.N., J. Singh, and P. Dalal, *Clinical profile and short-term outcome of neonates with esophageal atresia and tracheoesophageal fistula at tertiary care center in a developing country: A 25-year experience.* Journal of Clinical Neonatology, 2017. **6**(4): p. 225-230.
47. Sanghvi, K.P., et al., *Antenatal diagnosis of congenital renal malformations using ultrasound.* J Trop Pediatr, 1998. **44**(4): p. 235-40.
48. Sarin, Y.K., et al., *Duodenal webs: an experience with 18 patients.* J Neonatal Surg, 2012. **1**(2): p. 20.
49. Sharada, S., et al., *Multicystic dysplastic kidney: a retrospective study.* Indian Pediatrics, 2014. **51**(8): p. 641-3.
50. Singh, J.K., R.P. Kanojia, and K.L. Narasimhan, *Multicystic dysplastic kidney in children--a need for conservative and long term approach.* Indian Journal of Pediatrics, 2009. **76**(8): p. 809-12.
51. Solanki, S., et al., *Crossed fused renal ectopia: Challenges in diagnosis and management.* J Indian Assoc Pediatr Surg, 2013. **18**(1): p. 7-10.
52. Mirshemirani, A., et al., *Posterior Urethral Valves; A single Center Experience.* Iran J Pediatr, 2013. **23**(5): p. 531-5.

Confidential: For Review Only

53. Shahkar, L., et al., *An eleven-year review of congenital pulmonary lesions in a referral children's hospital in Tehran.* Archives of Pediatric Infectious Diseases, 2016. **4 (4) (no pagination)**(e32792).
54. Munim, S., et al., *Fetal outcome of cases with diaphragmatic hernia.* Journal of Maternal-Fetal & Neonatal Medicine, 2013. **26**(14): p. 1439-42.
55. Pitukkijronnakorn, S., et al., *The value of mid-trimester routine ultrasonographic screening in antenatal detection of congenital malformations.* Journal of the Medical Association of Thailand, 2009. **92**(6): p. 748-53.
56. Srisupundit, K., et al., *Fetal structural anomaly screening at 11-14 weeks of gestation at Maharaj Nakorn Chiang Mai Hospital.* Journal of the Medical Association of Thailand, 2006. **89**(5): p. 588-93.
57. Iliescu, D., et al., *Improved detection rate of structural abnormalities in the first trimester using an extended examination protocol.* Ultrasound Obstet Gynecol, 2013. **42**(3): p. 300-9.
58. Ognean, M.L., et al., *Esophageal atresia with distal fistula - unusual case series. Considerations related to epidemiological aspects, malformative associations, and prenatal diagnosis.* Rom J Morphol Embryol, 2017. **58**(3): p. 1069-1076.
59. Tarca, E. and S. Aprodu, *Past and present in omphalocele treatment in Romania.* Chirurgia (Bucuresti), 2014. **109**(4): p. 507-13.
60. Tarca, E. and S.G. Aprodu, *Gastroschisis treatment: what are the causes of high morbidity and mortality rates?* Chirurgia (Bucuresti), 2013. **108**(4): p. 516-20.
61. Tarca, E., I. Ciongradi, and S.G. Aprodu, *Birth Weight, Compromised Bowel and Sepsis are the Main Variables Significantly Influencing Outcome in Gastroschisis.* Chirurgia (Bucuresti), 2015. **110**(2): p. 151-6.
62. Tudorache, S., et al., *Prenatal diagnosis and perinatal outcome in congenital diaphragmatic hernia. Single tertiary center report.* Romanian Journal of Morphology & Embryology, 2014. **55**(3): p. 823-33.
63. Postoev, V.A., et al., *Changes in detection of birth defects and perinatal mortality after introduction of prenatal ultrasound screening in the Kola Peninsula (North-West Russia): combination of two birth registries.* BMC Pregnancy & Childbirth, 2015. **15**: p. 308.
64. Aygun, C., et al., *Experience of a tertiary care center on 100 newborns with neural tube defects.* Turk J Pediatr, 2013. **55**(4): p. 359-64.
65. Dane, B., et al., *Ultrasound screening for fetal major abnormalities at 11-14 weeks.* Acta Obstet Gynecol Scand, 2007. **86**(6): p. 666-70.
66. Orgul, G., et al., *Evaluation of pre- and postnatally diagnosed gastrointestinal tract obstructions.* Journal of Maternal-Fetal & Neonatal Medicine, 2018: p. 1-6.
67. Oztekin, O., et al., *Ultrasonographic diagnosis of fetal structural abnormalities in prenatal screening at 11-14 weeks.* Diagn Interv Radiol, 2009. **15**(3): p. 221-5.
68. Sahinoglu, Z., et al., *The prenatal diagnosis of cloacal dysgenesis sequence in six cases: can the termination of pregnancy always be the first choice?* Prenatal Diagnosis, 2004. **24**(1): p. 10-6.
69. Tabel, Y., et al., *Ultrasonographic screening of newborns for congenital anomalies of the kidney and the urinary tracts.* Urol J, 2010. **7**(3): p. 161-7.
70. Taskapilioglu, M.O., S. Isik, and E. Korfali, *Outcome of Myelomeningocele: The importance of prenatal diagnosis.* Neurosurgery Quarterly, 2014. **24**(3): p. 214-217.

Confidential: For Review Only

71. Campana, H., et al., *Prenatal sonographic detection of birth defects in 18 hospitals from South America.* Journal of Ultrasound in Medicine, 2010. **29**(2): p. 203-12.
72. Germani, M., et al., *Choledochal cyst in pediatric patients: a 10-year single institution experience.* Acta Gastroenterol Latinoam, 2011. **41**(4): p. 302-7.
73. Wyszynski, D.F., C. Perandones, and R.D. Bennun, *Attitudes toward prenatal diagnosis, termination of pregnancy, and reproduction by parents of children with nonsyndromic oral clefts in Argentina.* Prenatal Diagnosis, 2003. **23**(9): p. 722-7.
74. Carvalho, M.H., et al., *Detection of fetal structural abnormalities at the 11-14 week ultrasound scan.* Prenat Diagn, 2002. **22**(1): p. 1-4.
75. Luiza, A., et al., *A descriptive epidemiology study of oral cleft in sergipe, Brazil.* Int Arch Otorhinolaryngol, 2013. **17**(4): p. 390-4.
76. Tannuri, A.C., et al., *Evolution of critically ill patients with gastroschisis from three tertiary centers.* Clinics (Sao Paulo, Brazil), 2011. **66**(1): p. 17-20.
77. Vilela, P.C., et al., *Risk factors for adverse outcome of newborns with gastroschisis in a Brazilian hospital.* Journal of Pediatric Surgery, 2001. **36**(4): p. 559-64.
78. Correa, C., et al., *Congenital malformations of pediatric surgical interest: prevalence, risk factors, and prenatal diagnosis between 2005 and 2012 in the capital city of a developing country. Bogota, Colombia.* Journal of Pediatric Surgery, 2014. **49**(7): p. 1099-103.
79. De Rovetto, C.R., et al., *Unilateral renal agenesis: Case review of ambulatory pediatric nephrology clinics in Cali.* Colombia Medica, 2010. **41**(1): p. 52-59.
80. Rosselli, P., et al., *Prenatal Ultrasound Diagnosis of Congenital Talipes Equinovarus in Bogota (Colombia) Between 2003 and 2012.* Iowa Orthopaedic Journal, 2015. **35**: p. 156-9.
81. Saldarriaga-Gil, W., et al., *Evaluation of prenatal diagnosis of congenital anomalies diagnosable by prenatal ultrasound in patients in neonatal intensive care units of Cali, Colombia.* Colombia Medica, 2014. **45**(1): p. 32-8.
82. Xie, H.N., et al., *Prenatal surveillance of bronchopulmonary sequestration using 3-dimensional ultrasonography.* J Ultrasound Med, 2009. **28**(8): p. 989-94.
83. Ghavami, M. and R. Abedinzadeh, *Prevalence of perinatal central nervous system anomalies in East azarbaijan-iran.* Iran J Radiol, 2011. **8**(2): p. 79-81.
84. Kutuk, M.S., et al., *Prenatal diagnosis of upper extremity malformations with ultrasonography: Diagnostic features and perinatal outcome.* J Clin Ultrasound, 2017. **45**(5): p. 267-276.
85. Savran, B., et al., *The importance of antenatal diagnosis of congenital duodenal obstruction.* Ir J Med Sci, 2016. **185**(3): p. 695-8.
86. Shalaby, H., et al., *Types and Outcome of Fetal Urinary Anomalies in Low Resource Setting Countries: A Retrospective Study.* J Obstet Gynaecol India, 2016. **66**(5): p. 316-20.
87. Li, Z., et al., *Extremely high prevalence of neural tube defects in a 4-county area in Shanxi Province, China.* Birth Defects Research, 2006. **76**(4): p. 237-40.
88. Zhang, H., et al., *Retrospective study of prenatal diagnosed pulmonary sequestration.* Pediatric Surgery International, 2014. **30**(1): p. 47-53.
89. Kashyap, N., et al., *Early Detection of Fetal Malformation, a Long Distance Yet to Cover! Present Status and Potential of First Trimester Ultrasonography in Detection of*

Fetal Congenital Malformation in a Developing Country: Experience at a Tertiary Care Centre in India. Journal of Pregnancy, 2015. **2015**: p. 623059.
90. Kumar, M., et al., *Prenatal sonographic evaluation and postnatal outcome of renal anomalies.* Indian J Hum Genet, 2012. **18**(1): p. 75-82.
91. Kumar, M., et al., *Fetal renal anomaly: factors that predict survival.* Journal of pediatric urology, 2014. **10**(6): p. 1001-7.
92. Hsieh, Y.Y., et al., *Accuracy of sonography in predicting the outcome of fetal congenital diaphragmatic hernia.* Zhonghua Yi Xue Za Zhi (Taipei), 2000. **63**(10): p. 751-7.
93. Jaruratanasirikul, S., et al., *Prevalence of neural tube defect in southern Thailand: a population-based survey during 2009-2012.* Childs Nervous System, 2014. **30**(7): p. 1269-75.
94. Gammeltoft, T., et al., *Late-term abortion for fetal anomaly: Vietnamese women's experiences.* Reproductive Health Matters, 2008. **16**(31 Suppl): p. 46-56.
95. Oztarhan, K., et al., *Prevalence and distribution of congenital abnormalities in Turkey: differences between the prenatal and postnatal periods.* Congenital Anomalies, 2010. **50**(4): p. 221-5.
96. Brizot, M.L., et al., *Conjoined twins pregnancies: experience with 36 cases from a single center.* Prenat Diagn, 2011. **31**(12): p. 1120-5.
97. Pelizzari, E., et al., *Characteristics of fetuses evaluated due to suspected anencephaly: a population-based cohort study in southern Brazil.* Sao Paulo Medical Journal = Revista Paulista de Medicina, 2015. **133**(2): p. 101-8.
98. Oloyede, O.A. and R.A. Oyedele, *Women's attitude to prenatal screening services for congenital abnormalities in Nigeria.* Journal of Obstetrics & Gynaecology, 2008. **28**(4): p. 406-7.
99. Acharya, P., et al., *Screening for birth defects strategies for developing low resource countries.* Donald School Journal of Ultrasound in Obstetrics and Gynecology, 2013. **7**(4): p. 453-461.
100. Neogi, S.B., *Abortion for fetal abnormalities in India: need for critical review.* Asia-Pacific Journal of Public Health, 2010. **22**(1): p. 146-7.
101. Phadke, S.R., M. Agarwal, and S. Aggarwal, *Late termination of pregnancy for fetal abnormalities: The perspective of Indian lay persons and medical practitioners.* Prenatal Diagnosis, 2011. **31**(13): p. 1286-1291.
102. Ranji, A. and A.K. Dykes, *Ultrasound screening during pregnancy in Iran: womens' expectations, experiences and number of scans.* Midwifery, 2012. **28**(1): p. 24-9.
103. Arawi, T. and A. Nassar, *Prenatally diagnosed foetal malformations and termination of pregnancy: the case of Lebanon.* Developing World Bioethics, 2011. **11**(1): p. 40-7.
104. Senanayake, H.M. and M.V. de Silva, *Prenatal diagnosis of lethal congenital malformations in Sri Lanka.* Ceylon Medical Journal, 2001. **46**(4): p. 163.
105. Hostiuc, S., O. Buda, and M. Hostiuc, *Late abortion. Attitudes amongst young physicians in Romania.* Archives of Gynecology & Obstetrics, 2013. **288**(2): p. 431-7.
106. Lisker, R., A. Carnevale, and A.R. Villa, *Acceptance of induced abortion amongst medical students and physicians in Mexico.* Revista de Investigacion Clinica, 2006. **58**(4): p. 305-12.
107. Groisman, B., et al., *The use of a Toolkit for health needs assessment on neural tube defects in Argentina.* Journal of Community Genetics, 2013. **4**(1): p. 77-86.

Confidential: For Review Only

108. Benute, G.R., et al., *Feelings of women regarding end-of-life decision making after ultrasound diagnosis of a lethal fetal malformation.* Midwifery, 2012. **28**(4): p. 472-5.
109. Mirlesse, V. and I. Ville, *The uses of ultrasonography in relation to foetal malformations in Rio de Janeiro, Brazil.* Social Science & Medicine, 2013. **87**: p. 168-75.
110. WHO. *WHO recommendation on early ultrasound in pregnancy*. The WHO Reproductive Health Library 2016; Available from: https://extranet.who.int/rhl/topics/preconception-pregnancy-childbirth-and-postpartum-care/antenatal-care/who-recommendation-early-ultrasound-pregnancy.
111. Todros, T., E. Capuzzo, and P. Gaglioti, *Prenatal diagnosis of congenital anomalies.* Images Paediatr Cardiol, 2001. **3**(2): p. 3-18.
112. WHO, *WHO Study Group on Training in Diagnostic Ultrasound: Essentials, Principles, and Standards*, WHO, Editor. 1998, World Health Organization: Philadelphia, Pennsylvania.
113. LaGrone, L.N., et al., *A review of training opportunities for ultrasonography in low and middle income countries.* Trop Med Int Health, 2012. **17**(7): p. 808-19.
114. Goldenberg, R.L., et al., *Routine antenatal ultrasound in low- and middle-income countries: first look - a cluster randomised trial.* BJOG, 2018. **125**(12): p. 1591-1599.
115. Franklin, H.L., et al., *Factors influencing referrals for ultrasound-diagnosed complications during prenatal care in five low and middle income countries.* Reprod Health, 2018. **15**(1): p. 204.
116. Wright, N.J. and C. Global PaedSurg Research, *Management and outcomes of gastrointestinal congenital anomalies in low, middle and high income countries: protocol for a multicentre, international, prospective cohort study.* BMJ Open, 2019. **9**(8): p. e030452.
117. Global Initiative for Children's Surgery, *Global Initiative for Children's Surgery: A Model of Global Collaboration to Advance the Surgical Care of Children.* World J Surg, 2019. **43**(6): p. 1416-1425.
118. Anyanwu, L.C., N. Ade-Ajayi, and U. Rolle, *Major abdominal wall defects in the low- and middle-income setting: current status and priorities.* Pediatr Surg Int, 2020. **36**(5): p. 579-590.

Confidential: For Review Only

Investigating the Use of Ultrasonography for the Antenatal Diagnosis of Structural Congenital Anomalies in Low- and Middle-Income Countries: A Systematic Review

Figure 1: Flowchart of the Screening Process

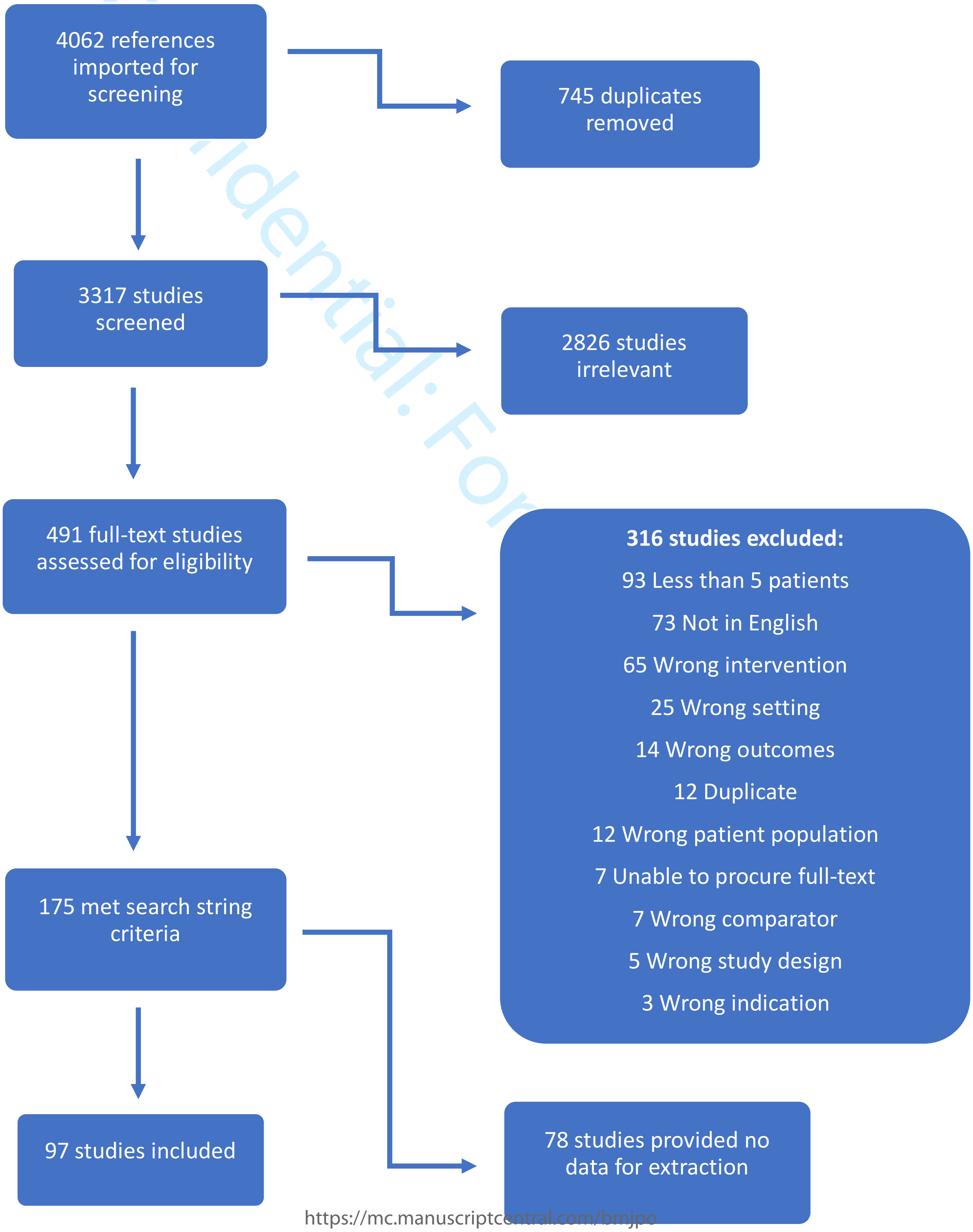

Confidential: For

Investigating the Use of Ultrasonography for the Antenatal Diagnosis of Structural Congenital Anomalies in Low- and Middle-Income Countries: A Systematic Review

Figure 2: Regional Depiction of Articles included in the Systematic Review

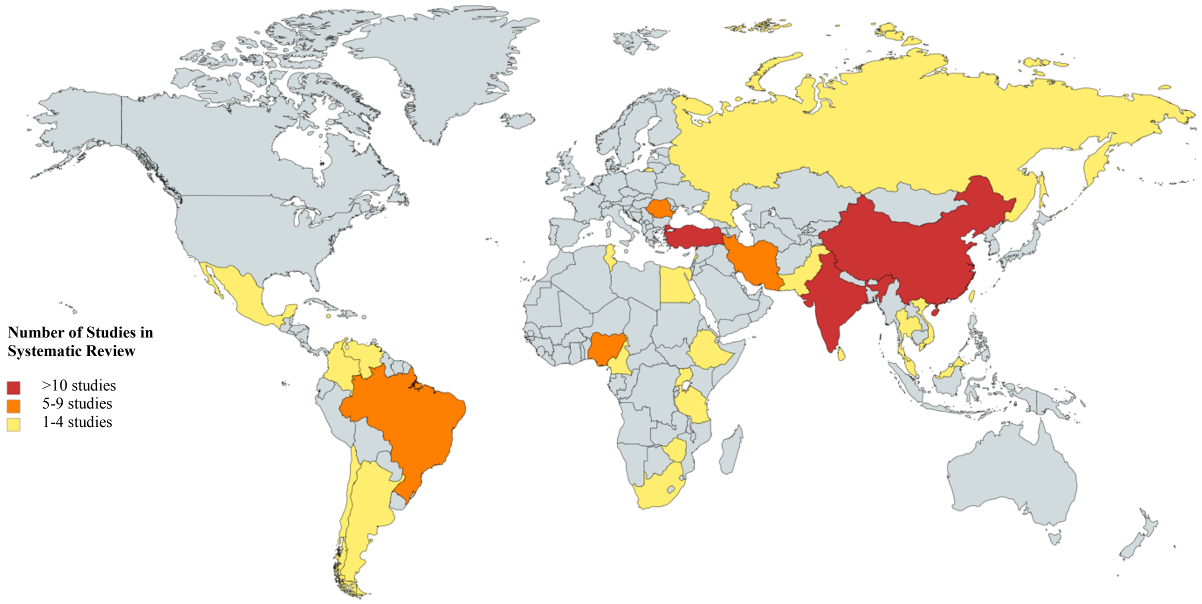

or Review Only

Supplementary File 1

Investigating the Use of Ultrasonography for the Antenatal Diagnosis of Structural Congenital Anomalies in Low- and Middle-Income Countries: A Systematic Review

Search Strings

Search String 1	Search String 2	Search String 3
Congenital Anomalies, Congenital Abnormalities, Congenital Malformation, Fetal Malformation, Birth Defects, Anencephaly, Conjoined Twins, Congenital Heart Defects, Anorectal Malformations, Anal Stenosis, Anal Atresia, Imperforate Anus, Biliary Atresia, Choledochal Cyst, Diaphragmatic Eventration, Esophageal Atresia, Tracheoesophageal Fistula, Intestinal Atresia, Duodenal Obstruction, Duodenal Atresia, Colonic Atresia, Malrotation, Apple Peel Syndrome, Congenital Diaphragmatic Hernias, Gastroschisis, Abdominal Wall Defects, Exomphalos, Omphalocele, Congenital Limb Deformities, Neural Tube Defects, Bronchogenic Cyst, Bronchopulmonary Sequestration, Congenital Cystic Adenomatoid Malformation of Lung, Renal Anomalies, Genito-urinary Anomalies, Maxillofacial Abnormalities, Mouth Abnormalities, Umbilical Hernia, Hirschsprung Disease, Ganglionic Megacolon, Rectosigmoid Aganglionosis, Colonic Aganglionosis, Intestinal Aganglionosis, Volvulus, Intestinal Volvulus	LMICs, Low- and Middle-Income Countries, Developing Countries, Low-Resource Settings, Underdeveloped Countries, Low-Income Countries, Middle-Income Countries, Limited Resource Settings, Africa South of the Sahara, Sub-Saharan Africa, Less Resourced Communities, Afghanistan, Albania, Algeria, American Samoa, Angola, Argentina, Armenia, Azerbaijan, Bangladesh, Belarus, Belize, Benin, Bhutan, Bolivia, Bosnia and Herzegovina, Botswana, Brazil, Bulgaria, Burkina Faso, Burundi, Cabo Verde, Cambodia, Cameroon, Central African Republic, Chad, China, Colombia, Comoros, Democratic Republic of the Congo, DRC, Republic of the Congo, Costa Rica, Cote d'Ivoire, Ivory Coast, Croatia, Cuba, Djibouti, Dominica, Dominican Republic, Ecuador, Egypt, El Salvador, Equatorial Guinea, Eritrea, Ethiopia, Fiji, Gabon, Gambia, Georgia, Ghana, Grenada, Guatemala, Guinea, Guinea-Bissau, Guyana, Haiti, Honduras, India, Indonesia, Islamic Republic of Iran, Iraq, Jamaica, Jordan, Kazakhstan, Kenya, Kiribati, Democratic People's Republic of Korea, Kosovo, Kyrgyz Republic, Lao PDR, Laos, Lebanon, Lesotho, Liberia, Libya, Macedonia Republic, Madagascar, Malawi, Malaysia, Maldives, Mali, Marshall Islands, Mauritania, Mauritius, Mexico, Micronesia, Moldova, Mongolia, Montenegro, Morocco, Mozambique, Myanmar, Namibia, Nauru, Nepal, Nicaragua, Niger, Nigeria, Pakistan, Panama, Papua New Guinea, Paraguay, Peru, Philippines, Romania, Russian Federation, Rwanda, Samoa, Sao Tome and Principe, Senegal, Serbia, Sierra Leone, Solomon Islands, Somalia, Somaliland, South Africa, South Sudan, Sri Lanka, Saint Lucia, Saint Vincent and the Grenadines, Sudan, Suriname, Swaziland, Syrian Arab Republic, Syria, Tajikistan, Tanzania, Thailand, Timor-Leste, East Timor, Togo, Tonga, Tunisia, Turkey, Turkmenistan, Tuvalu, Uganda, Ukraine, Uzbekistan, Vanuatu, Venezuela, Vietnam, West Bank and Gaza, Republic of Yemen, Zambia, Zimbabwe	Antenatal Diagnosis, Prenatal Diagnosis, Antenatal Screening, Prenatal Screening, Antenatal Ultrasound, Prenatal Ultrasound, Antenatal Ultrasonography, Prenatal Ultrasonography

Confidential: For Review Only

Supplementary File 2

Investigating the Use of Ultrasonography for the Antenatal Diagnosis of Structural Congenital Anomalies in Low- and Middle-Income Countries: A Systematic Review

Search Strategy as Run on Ovid MEDLINE(R) Epub Ahead of Print, In-Process & Other Non-Indexed Citations, Ovid MEDLINE(R) Daily, Ovid MEDLINE and Versions(R) <1946 to June 27, 2018>

Confidential: For Review Only

No.	Search Terms	Results
1	exp Congenital Abnormalities/ or congenital anomal*.mp.	559922
2	exp Congenital Abnormalities/ or Congenital Abnormal*.mp.	555311
3	Congenital Malformation*.mp.	13826
4	Fetal Malformation*.mp.	1917
5	Birth Defect*.mp.	9122
6	Anencephaly.mp. or exp ANENCEPHALY/	3875
7	Conjoined Twins.mp. or exp Twins, Conjoined/	2022
8	exp Heart Defects, Congenital/ or Congenital Heart Defect*.mp.	142854
9	Anorectal Malformation*.mp.	1896
10	Anal Stenosis.mp.	389
11	Anal Atresia.mp. or exp Anus, Imperforate/	2815
12	Imperforate Anus.mp. or exp Anus, Imperforate/	2992
13	Biliary atresia.mp. or exp Biliary Atresia/	4482
14	exp Choledochal Cyst/ or Choledochal Cyst*.mp.	2825
15	exp Diaphragmatic Eventration/ or Diaphragmatic Eventration*.mp.	943
16	Esophageal Atresia.mp. or exp Esophageal Atresia/	4173
17	exp Tracheoesophageal Fistula/ or Tracheo-?esophageal fistula*.mp.	3676
18	Intestinal Atresia.mp. or exp Intestinal Atresia/	2301
19	Duodenal atresia.mp.	839
20	Colonic Atresia.mp.	152
21	Malrotation.mp.	2393
22	Apple peel syndrome.mp.	16
23	exp Hernias, Diaphragmatic, Congenital/ or congenital diaphragmatic hernia*.mp.	5530
24	Gastroschisis.mp. or exp GASTROSCHISIS/	2313
25	Abdominal Wall Defect*.mp.	2009
26	Exomphalos.mp. or exp Hernia, Umbilical/	3740
27	Omphalocele.mp. or exp Hernia, Umbilical/	4242
28	Congenital Limb Deformities.mp. or exp Limb Deformities, Congenital/	22483
29	exp Neural Tube Defects/ or Neural Tube Defect*.mp.	29051
30	exp Bronchogenic Cyst/ or Bronchogenic Cyst*.mp.	1810
31	exp Bronchopulmonary Sequestration/ or Bronchopulmonary sequestration*.mp.	2022
32	exp "Cystic Adenomatoid Malformation of Lung, Congenital"/ or Congenital Cystic adenomatoid malformation of lung*.mp.	874
33	Renal Anomal*.mp.	1185
34	exp Urogenital Abnormalities/ or Genito-urinary Anomalies.mp.	57735
35	exp Maxillofacial Abnormalities/ or Maxillofacial Abnormalit*.mp.	28265
36	exp Mouth Abnormalities/ or Mouth Abnormalit*.mp.	24459
37	Umbilical Hernia.mp. or exp Hernia, Umbilical/	4200
38	Hirschsprung disease.mp. or exp Hirschsprung Disease/	4567
39	exp Megacolon/ or Ganglionic Megacolon.mp.	7053
40	Rectosigmoid aganglionosis.mp.	15

41	Colonic Aganglionosis.mp.	311
42	Intestinal Aganglionosis.mp.	190
43	Volvulus.mp. or exp Intestinal Volvulus/	8927
44	LMICs.mp.	2001
45	(low- and middle-income countr*).mp. [mp=title, abstract, original title, name of substance word, subject heading word, floating sub-heading word, keyword heading word, protocol supplementary concept word, rare disease supplementary concept word, unique identifier, synonyms]	10910
46	Low-resource setting*.mp.	2719
47	Underdeveloped Countries.mp.	817
48	low-income countries.mp.	4707
49	exp Developing Countries/ or Developing countr*.mp.	115302
50	middle-income countries.mp.	11136
51	limited resource setting*.mp.	253
52	Africa South of the Sahara.mp. or exp "Africa South of the Sahara"/	184102
53	Sub-Saharan Africa.mp. or exp "Africa South of the Sahara"/	190396
54	Less Resourced communities.mp.	1
55	Afghanistan.mp. or exp AFGHANISTAN/	6065
56	Albania.mp. or exp ALBANIA/	1182
57	Algeria.mp. or exp ALGERIA/	3621
58	American Samoa.mp.	354
59	Angola.mp. or exp ANGOLA/	1403
60	Argentina.mp. or exp ARGENTINA/	19610
61	Armenia.mp. or exp ARMENIA/	1676
62	Azerbaijan.mp. or exp AZERBAIJAN/	1765
63	Bangladesh.mp. or exp BANGLADESH/	12907
64	Belarus.mp. or exp "Republic of Belarus"/	2553
65	Belize.mp. or exp BELIZE/	868
66	Benin.mp. or exp BENIN/	3441
67	exp BHUTAN/ or Bhutan.mp.	628
68	Bolivia.mp. or exp BOLIVIA/	3594
69	(Bosnia and Herzegovina).mp. [mp=title, abstract, original title, name of substance word, subject heading word, floating sub-heading word, keyword heading word, protocol supplementary concept word, rare disease supplementary concept word, unique identifier, synonyms]	2477
70	Botswana.mp. or exp BOTSWANA/	2247
71	Brazil.mp. or exp BRAZIL/	101919
72	Bulgaria.mp. or exp BULGARIA/	7554
73	Burkina Faso.mp. or exp Burkina Faso/	3904
74	Burundi.mp. or exp BURUNDI/	871
75	Cabo Verde.mp. or exp Cabo Verde/	211
76	Cambodia.mp. or exp CAMBODIA/	4102
77	Cameroon.mp. or exp CAMEROON/	6804
78	Central African Republic.mp. or exp Central African Republic/	1096
79	Chad.mp. or exp CHAD/	1144
80	China.mp. or exp CHINA/	205799
81	Colombia.mp. or exp COLOMBIA/	12533
82	Comoros.mp. or exp COMOROS/	427
83	Democratic Republic of the Congo.mp. or exp "Democratic Republic of the Congo"/	4506
84	exp "Democratic Republic of the Congo"/ or DRC.mp.	4827
85	Republic of the Congo.mp. or exp Congo/	6176
86	Costa Rica.mp. or exp Costa Rica/	5088
87	Cote d'Ivoire.mp. or exp Cote d'Ivoire/	3527
88	Ivory Coast.mp. or exp Cote d'Ivoire/	3432

Confidential: For Review Only

89	Croatia.mp. or exp CROATIA/	8261
90	Cuba.mp. or exp CUBA/	6343
91	Djibouti.mp. or exp DJIBOUTI/	372
92	Dominica.mp. or exp DOMINICA/	430
93	Dominican Republic.mp. or exp Dominican Republic/	2128
94	Ecuador.mp. or exp ECUADOR/	4722
95	exp EGYPT/ or Egypt.mp.	18702
96	El Salvador.mp. or exp El Salvador/	1319
97	Equatorial Guinea.mp. or exp Equatorial Guinea/	419
98	Eritrea.mp. or exp ERITREA/	518
99	Ethiopia.mp. or exp ETHIOPIA/	13354
100	Fiji.mp. or exp FIJI/	1620
101	Gabon.mp. or exp GABON/	1925
102	exp GAMBIA/ or Gambia.mp.	2958
103	exp "GEORGIA (REPUBLIC)"/ or exp GEORGIA/ or Georgia.mp.	15842
104	Ghana.mp. or exp GHANA/	9432
105	Grenada.mp. or exp GRENADA/	308
106	Guatemala.mp. or exp GUATEMALA/	3907
107	Guinea.mp. or exp GUINEA-BISSAU/ or exp EQUATORIAL GUINEA/ or exp GUINEA/ or exp PAPUA NEW GUINEA/ or exp NEW GUINEA/	160074
108	Guyana.mp. or exp GUYANA/	1136
109	Haiti.mp. or exp HAITI/	3707
110	Honduras.mp. or exp HONDURAS/	1736
111	India.mp. or exp INDIA/	129215
112	Indonesia.mp. or exp INDONESIA/	13107
113	exp Iran/ or Islamic Republic of Iran.mp.	21280
114	exp IRAQ/ or Iraq.mp.	9035
115	Jamaica.mp. or exp JAMAICA/	4427
116	Jordan.mp. or exp JORDAN/	6040
117	Kazakhstan.mp. or exp KAZAKHSTAN/	3153
118	Kenya.mp. or exp KENYA/	18798
119	Kiribati.mp. or exp Micronesia/	1934
120	Democratic People's Republic of Korea.mp. or exp "Democratic People's Republic of Korea"/	246
121	Kosovo.mp. or exp KOSOVO/	840
122	Kyrgyz Republic.mp. or exp Kyrgyzstan/	1225
123	exp Laos/ or Lao PDR.mp.	1876
124	Laos.mp. or exp LAOS/	2431
125	Lebanon.mp. or exp LEBANON/	5011
126	Lesotho.mp. or exp LESOTHO/	645
127	Liberia.mp. or exp LIBERIA/	1667
128	Libya.mp. or exp LIBYA/	1554
129	Macedonia Republic.mp. or exp "Macedonia (Republic)"/	475
130	Madagascar.mp. or exp MADAGASCAR/	4734
131	Malawi.mp. or exp MALAWI/	6317
132	Malaysia.mp. or exp MALAYSIA/	18390
133	Maldives.mp. or exp Indian Ocean Islands/	10595
134	Mali.mp. or exp MALI/	3511
135	Marshall Islands.mp. or exp Micronesia/	1959
136	Mauritania.mp. or exp MAURITANIA/	626
137	Mauritius.mp. or exp MAURITIUS/	953
138	exp MEXICO/ or Mexico.mp.	53282
139	Micronesia.mp. or exp MICRONESIA/	2243
140	Moldova.mp. or exp MOLDOVA/	920

Confidential: For Review Only

141	Mongolia.mp. or exp MONGOLIA/	3809
142	Montenegro.mp. or exp MONTENEGRO/	749
143	Morocco.mp. or exp MOROCCO/	6855
144	Mozambique.mp. or exp MOZAMBIQUE/	3236
145	Myanmar.mp. or exp MYANMAR/	3198
146	Namibia.mp. or exp NAMIBIA	1502
147	Nauru.mp. or exp Micronesia/	1921
148	Nepal.mp. or exp NEPAL/	9337
149	Nicaragua.mp. or exp NICARAGUA/	1949
150	exp NIGER/ or Niger.mp.	12299
151	Nigeria.mp. or exp NIGERIA/	32627
152	Pakistan.mp. or exp PAKISTAN/	20153
153	exp PANAMA/ or Panama.mp.	4265
154	Papua New Guinea.mp. or exp Papua New Guinea/	5003
155	Paraguay.mp. or exp PARAGUAY/	1433
156	Peru.mp. or exp PERU/	11249
157	Philippines.mp. or exp PHILIPPINES/	10698
158	Romania.mp. or exp ROMANIA/	11521
159	exp Russia/ or Russian Federation.mp.	51376
160	Rwanda.mp. or exp RWANDA/	2883
161	exp AMERICAN SAMOA/ or Samoa.mp. or exp SAMOA/ or exp "INDEPENDENT STATE OF SAMOA"/	1024
162	(Sao Tome and Principe).mp. [mp=title, abstract, original title, name of substance word, subject heading word, floating sub-heading word, keyword heading word, protocol supplementary concept word, rare disease supplementary concept word, unique identifier, synonyms]	135
163	Senegal.mp. or exp SENEGAL/	7067
164	Serbia.mp. or exp SERBIA/	4574
165	Sierra Leone.mp. or exp Sierra Leone/	2103
166	Solomon Islands.mp. or exp Melanesia/	6193
167	Somalia.mp. or exp SOMALIA/	1968
168	Somaliland.mp.	136
169	South Africa.mp. or exp South Africa/	47075
170	South Sudan.mp. or exp South Sudan/	397
171	Sri Lanka.mp. or exp Sri Lanka/	7342
172	Saint Lucia.mp. or exp Saint Lucia/	114
173	(Saint Vincent and the Grenadines).mp. [mp=title, abstract, original title, name of substance word, subject heading word, floating sub-heading word, keyword heading word, protocol supplementary concept word, rare disease supplementary concept word, unique identifier, synonyms]	57
174	exp SUDAN/ or Sudan.mp.	8429
175	Suriname.mp. or exp SURINAME/	1098
176	Swaziland.mp. or exp SWAZILAND/	818
177	Syrian Arab Republic.mp.	73
178	Syria.mp. or exp SYRIA/	2407
179	Tajikistan.mp. or exp TAJIKISTAN/	910
180	Tanzania.mp. or exp TANZANIA/	12921
181	Thailand.mp. or exp THAILAND/	32186
182	exp Timor-Leste/ or Timor-Leste.mp.	311
183	East Timor.mp.	187
184	Togo.mp. or exp TOGO/	1491
185	Tonga.mp. or exp TONGA/	463
186	Tunisia.mp. or exp TUNISIA/	9057
187	exp TURKEY/ or Turkey.mp.	46898

Confidential: For Review Only

188	Turkmenistan.mp. or exp TURKMENISTAN/	717
189	Tuvalu.mp. or exp Micronesia/	1884
190	Uganda.mp. or exp UGANDA/	13956
191	Ukraine.mp. or exp UKRAINE/	16702
192	Uzbekistan.mp. or exp UZBEKISTAN/	2161
193	Vanuatu.mp. or exp VANUATU/	629
194	Venezuela.mp. or exp VENEZUELA/	6620
195	exp VIETNAM/ or Vietnam.mp.	15919
196	(West Bank and Gaza).mp. [mp=title, abstract, original title, name of substance word, subject heading word, floating sub-heading word, keyword heading word, protocol supplementary concept word, rare disease supplementary concept word, unique identifier, synonyms]	194
197	Republic of Yemen.mp. or exp Yemen/	1264
198	Zambia.mp. or exp ZAMBIA/	5404
199	Zimbabwe.mp. or exp ZIMBABWE/	6879
200	Antenatal Diagnosis.mp.	2383
201	Prenatal Diagnosis.mp. or exp Prenatal Diagnosis/	74636
202	Antenatal Screening.mp.	1193
203	Prenatal Screening.mp.	2579
204	Antenatal Ultrasound.mp.	702
205	Prenatal Ultrasound.mp.	2203
206	Antenatal Ultrasonography.mp.	200
207	Prenatal Ultrasonography.mp. or exp Ultrasonography, Prenatal/	30427
208	1 or 2 or 3 or 4 or 5 or 6 or 7 or 8 or 9 or 10 or 11 or 12 or 13 or 14 or 15 or 16 or 17 or 18 or 19 or 20 or 21 or 22 or 23 or 24 or 25 or 26 or 27 or 28 or 29 or 30 or 31 or 32 or 33 or 34 or 35 or 36 or 37 or 38 or 39 or 40 or 41 or 42 or 43	607001
209	44 or 45 or 46 or 47 or 48 or 49 or 50 or 51 or 52 or 53 or 54 or 55 or 56 or 57 or 58 or 59 or 60 or 61 or 62 or 63 or 64 or 65 or 66 or 67 or 68 or 69 or 70 or 71 or 72 or 73 or 74 or 75 or 76 or 77 or 78 or 79 or 80 or 81 or 82 or 83 or 84 or 85 or 86 or 87 or 88 or 89 or 90 or 91 or 92 or 93 or 94 or 95 or 96 or 97 or 98 or 99 or 100 or 101 or 102 or 103 or 104 or 105 or 106 or 107 or 108 or 109 or 110 or 111 or 112 or 113 or 114 or 115 or 116 or 117 or 118 or 119 or 120 or 121 or 122 or 123 or 124 or 125 or 126 or 127 or 128 or 129 or 130 or 131 or 132 or 133 or 134 or 135 or 136 or 137 or 138 or 139 or 140 or 141 or 142 or 143 or 144 or 145 or 146 or 147 or 148 or 149 or 150 or 151 or 152 or 153 or 154 or 155 or 156 or 157 or 158 or 159 or 160 or 161 or 162 or 163 or 164 or 165 or 166 or 167 or 168 or 169 or 170 or 171 or 172 or 173 or 174 or 175 or 176 or 177 or 178 or 179 or 180 or 181 or 182 or 183 or 184 or 185 or 186 or 187 or 188 or 189 or 190 or 191 or 192 or 193 or 194 or 195 or 196 or 197 or 198 or 199 (1385344)	1385344
210	200 or 201 or 202 or 203 or 204 or 205 or 206 or 207	77525
211	208 and 209 and 210	896

Confidential: For Review Only

Supplementary File 3

Investigating the Use of Ultrasonography for the Antenatal Diagnosis of Structural Congenital Anomalies in Low- and Middle-Income Countries: A Systematic Review

Data Extraction Template

**Reference**	Reference (Full)
	Title
	Author(s)
	Publication Date
	Year of Publication
	Journal
**Place of Study**	Hospital
	City
	Country
	Continent
**Study Type**	Type of Study (RCT, Cohort, Case-Control, etc.)
**Publication Status**	Has Study Been Published?
**Study Population**	Number of Patients
	Patient Cohort (Institutional, Multi-Center, Regional, National, etc.)
	Patient Cohort (All Patients Presenting with a Condition vs. Those with an Antenatal Diagnosis)
	Gestational Age (Mean, Median)
	Type of Anomaly

Confidential: For Review Only

**Diagnosis**	Type of Ultrasound (Abdominal vs. Transvaginal; 2D vs. 3D)
	Percent Receiving Antenatal Ultrasound
	Percent with Any Anomaly Detected
	Percent with Accurate Antenatal Diagnosis
	Incidence of Anomaly
	Training of Ultrasonographer
	Referral Rate for Further Antenatal Care
	Referral Rate to Tertiary Clinic
	Percentage Born in a Pediatric Surgery Center
**Outcomes**	Mortality Rate in Live Births
	Termination Rate
	Complications (Using Clavien-Dindo Classification)
	Additional Notes Regarding the Effect of Antenatal Diagnosis on Outcomes
	Other Benefits of Antenatal Diagnosis (i.e., Parental Counselling, Option for Termination, etc.)
	Comments Regarding Policy
	Additional Notes/Other Useful Information

Confidential: For Review Only

Supplementary File 4

Investigating the Use of Ultrasonography for the Antenatal Diagnosis of Structural Congenital Anomalies in Low- and Middle-Income Countries: A Systematic Review

Table of Studies Excluded By Language

**Language**	**Number of Studies**
Chinese	23
Russian	16
Spanish	15
French	12
Portuguese	6
Romanian	1
**Total**	**73**

Confidential: For Review Only

Supplementary File 5

Investigating the Use of Ultrasonography for the Antenatal Diagnosis of Structural Congenital Anomalies in Low- and Middle-Income Countries: A Systematic Review

List of Countries Represented in Systematic Review Data Extraction

Country	No. of Studies (%)
India	17 (17.0%)
China	10 (10.0%)
Turkey	10 (10.0%)
Brazil	9 (9.0%)
Nigeria	9 (9.0%)
Romania	7 (7.0%)
Iran	6 (6.0%)
Argentina	4 (4.0%)
Colombia	4 (4.0%)
Thailand	4 (4.0%)
South Africa	2 (2.0%)
Cameroon	1 (1.0%)
Chile	1 (1.0%)
Egypt	1 (1.0%)
Ethiopia	1 (1.0%)
Jamaica	1 (1.0%)
Lebanon	1 (1.0%)
Malaysia	1 (1.0%)
Mexico	1 (1.0%)
Pakistan	1 (1.0%)
Russia	1 (1.0%)
Sri Lanka	1 (1.0%)
Taiwan	1 (1.0%)
Tanzania	1 (1.0%)
Tunisia	1 (1.0%)
Uganda	1 (1.0%)
Venezuela	1 (1.0%)
Vietnam	1 (1.0%)
Zimbabwe	1 (1.0%)
**Total**	**100**

This table represents all of the countries from the 97 included studies. 96 studies were conducted in a single country and 1 study was conducted across four countries.

Confidential: For Review Only

BMJ Paediatrics Open

Investigating the Use of Ultrasonography for the Antenatal Diagnosis of Structural Congenital Anomalies in Low- and Middle-Income Countries: A Systematic Review

Journal:	*BMJ Paediatrics Open*
Manuscript ID	bmjpo-2020-000684.R2
Article Type:	Original research
Date Submitted by the Author:	17-Jun-2020
Complete List of Authors:	Goley, Stephanie; King's College London, Department of Global Health & Social Medicine<br>Sakula-Barry, Sidonie; World Cancer Research Fund<br>Adofo-Ansong, Nana; Mafikeng Provincial Hospital<br>Isaaya Ntawunga, Laurence; University of Rwanda, Rwanda Spina Bifida and Hydrocephalus Relief<br>Tekyiwa Botchway, Maame; University of the Witwatersrand<br>Kelly, Ann; King's College London, Department of Global Health & Social Medicine<br>Wright, Naomi; King's College London, King's Centre for Global Health and Health Partnerships
Keywords:	Screening, Neonatology, Congenital Abnorm

SCHOLARONE™
Manuscripts

I, the Submitting Author has the right to grant and does grant on behalf of all authors of the Work (as defined in the below author licence), an exclusive licence and/or a non-exclusive licence for contributions from authors who are: i) UK Crown employees; ii) where BMJ has agreed a CC-BY licence shall apply, and/or iii) in accordance with the terms applicable for US Federal Government officers or employees acting as part of their official duties; on a worldwide, perpetual, irrevocable, royalty-free basis to BMJ Publishing Group Ltd ("BMJ") its licensees and where the relevant Journal is co-owned by BMJ to the co-owners of the Journal, to publish the Work in this journal and any other BMJ products and to exploit all rights, as set out in our licence.

The Submitting Author accepts and understands that any supply made under these terms is made by BMJ to the Submitting Author unless you are acting as an employee on behalf of your employer or a postgraduate student of an affiliated institution which is paying any applicable article publishing charge ("APC") for Open Access articles. Where the Submitting Author wishes to make the Work available on an Open Access basis (and intends to pay the relevant APC), the terms of reuse of such Open Access shall be governed by a Creative Commons licence – details of these licences and which Creative Commons licence will apply to this Work are set out in our licence referred to above.

Other than as permitted in any relevant BMJ Author's Self Archiving Policies, I confirm this Work has not been accepted for publication elsewhere, is not being considered for publication elsewhere and does not duplicate material already published. I confirm all authors consent to publication of this Work and authorise the granting of this licence.

Confidential: For Review Only

Investigating the Use of Ultrasonography for the Antenatal Diagnosis of Structural Congenital Anomalies in Low- and Middle-Income Countries: A Systematic Review

Stephanie Goley[1], Sidonie Sakula-Barry[2], Nana Adofo-Ansong[3], Laurence Isaaya Ntawunga[4], Maame Tekyiwa Botchway[5], Ann H. Kelly[1], Naomi Wright[6]

[1]Department of Global Health & Social Medicine, King's College London, London, UK
[2]World Cancer Research Fund UK, London, UK
[3]Mafikeng Provincial Hospital, Mafikeng, South Africa
[4]Rwanda Spina Bifida and Hydrocephalus Relief, University of Rwanda, Kigali, Rwanda
[5]University of Witwatersrand, Johannesburg, South Africa
[6]King's Centre for Global Health and Health Partnerships, School of Population Health and Environmental Sciences, King's College London, London, UK

Corresponding Author:

Stephanie Goley, BA MSc
Department of Global Health & Social Medicine, King's College London, London, UK
Correspondence to: Centre for Global Health and Health Partnerships, Suite 2.13 Weston Education Centre, Cutcombe Road, London, SE5 9RJ
Telephone: +1 (815) 830-5360

Authors' Email Addresses:

stephanie.goley@kcl.ac.uk
s.sakulabarry@gmail.com
adfnan001@myuct.ac.za
rsbhrelief@gmail.com
1596010@students.wits.ac.za
ann.kelly@kcl.ac.uk
naomiwright@doctors.org.uk

Confidential: For Review Only

What is known?

- Congenital anomalies are the fifth leading cause of death in children under 5-years of age globally.
- 97% of congenital anomaly deaths occur in LMICs, many of which may be preventable with antenatal diagnosis and planned surgical intervention following birth.
- Antenatal ultrasound examinations in HICs are commonplace and highly accurate, but accessibility and effectiveness are limited in LMICs.

What this study adds?

- Rates of antenatal ultrasound examination vary significantly in LMICs, ranging from 6.8% to 98.8%.
- There is significant variation in the accuracy of antenatal diagnosis in LMICs, with detection rates varying from 0% to 100% (median of 16.7% in Africa).
- Available data suggests that the level of ultrasonographer training may affect the accuracy of diagnosis, but further research into this is required.

Confidential: For Review Only

Abstract

Background: Congenital anomalies are the 5th leading cause of under-5 mortality globally. The greatest burden is faced by those in low- and middle-income countries (LMICs), where over 95% of deaths occur. Many of these deaths may be preventable through antenatal diagnosis and early intervention. This systematic literature review investigates the use of antenatal ultrasound to diagnose congenital anomalies and improve the health outcomes of infants in LMICs.

Methods: A systematic literature review was conducted using three search strings: 1) structural congenital anomalies; 2) LMICs; and 3) antenatal diagnosis. The search was conducted on the following databases: MEDLINE, EMBASE, PubMed, and the Cochrane Library. Title, abstract and full-text screening was undertaken in duplicate by two reviewers independently. Consensus among the wider authorship was sought for discrepancies. The primary analysis focused on the availability and effectiveness of antenatal ultrasound for diagnosing structural congenital anomalies. Secondary outcomes included neonatal morbidity and mortality, termination rates, referral rates for further antenatal care and training level of the ultrasonographer. Relevant policy data was sought.

Results: The search produced 4,062 articles; 97 were included in the review. The median percentage of women receiving an antenatal ultrasound examination was 50.0% in African studies and 90.7% in Asian studies (range 6.8% to 98.8%). Median detection rates were: 16.7% Africa, 34.3% South America, 34.7% Asia, and 47.3% Europe, (range 0 to 100%). The training level of the ultrasound provider may affect detection rates. Four articles compared morbidity and mortality outcomes, with inconclusive results. Significant variations in termination rates were found (0 to 98.3%). No articles addressed referral rates.

Conclusion: Antenatal detection of congenital anomalies remains highly variable across LMICs and is particularly low in sub-Saharan Africa. Further research is required to investigate the role of antenatal diagnosis for improving survival from congenital anomalies in LMICs.

PROSPERO Registration ID: CRD42019105620

Confidential: For Review Only

Introduction

Congenital anomalies are one of the leading causes of neonatal morbidity and mortality globally. The greatest burden of disease is faced by those in low- and middle-income countries (LMICs), as 94% of congenital anomalies occur in these regions [1]. Congenital anomalies comprise 9% of the total global burden of surgical disease and account for 57.7 million disability-adjusted life years (DALYs) lost annually across the globe [2]. Recent estimates suggest that approximately 303,000 neonates die annually from congenital anomalies before reaching just four weeks of age [3]. However, many experts believe that this is an under-estimate, due to a lack of congenital anomaly registries and some neonates dying without a diagnosis or inclusion within current statistics.

The WHO defines congenital anomalies as either structural or functional abnormalities which occur during intrauterine development [3]. Structural anomalies are physical abnormalities that occur when the organs or skeletal structure are improperly formed. These can often be detected on ultrasound antenatally and are the focus of this review. Some common structural congenital anomalies include heart defects, cleft lip and palate, neural tube defects, limb deformities, and abdominal wall defects. Many structural anomalies require immediate surgical intervention at birth to avoid death or preventable disability. In such cases, antenatal diagnosis permits delivery at a center where the appropriate surgical care can be provided upon delivery. For example, gastroschisis where the intestines protrude through a hole in the abdominal wall at birth. In HICs, where the majority of cases are antenatally diagnosed, mortality is less than 5%, while in many LMICs, with limited antenatal diagnosis, the mortality rate can be as high as 100% [4-6].

The use of ultrasound technology in LMICs has significantly increased in recent years, as ultrasound machines have become more compact, transportable, and affordable [7]. Yet, a great number of congenital anomalies that can be detected antenatally via ultrasound go undiagnosed. Factors identified as barriers to effective antenatal ultrasound include limited training, equipment shortages, faulty ultrasound equipment, and lack of maintenance services [7]. In recent years, higher global priority has been given to neonatal health. Sustainable Development Goal (SDG) 3.2 aims to end all preventable under-5 deaths and reduce neonatal mortality in every country to 12 per 1,000 live births [8]. In 2010, the WHO released the 63rd World Health Assembly Report on Birth Defects, recommending ‘prevention whenever possible, to implement screening programs and to provide care and ongoing support to children with birth defects and their families’ [9].

To develop a better understanding of antenatal ultrasound provision in LMICs, this study aimed to systematically investigate the availability and effectiveness of antenatal ultrasound in the

diagnosis of structural congenital anomalies in LMICs. It further aimed to evaluate the effects of antenatal ultrasound diagnosis on mortality and morbidity outcomes, termination rates and referral for further antenatal care and management planning. Additionally, it assessed the level of training of ultrasonographers undertaking antenatal scans and relevant antenatal ultrasound policies in LMICs. This information is vital to help clarify the existing disparities in antenatal ultrasound provision and the potential benefits for improved health outcomes.

Methodology

Preferred Reporting Items for Systematic review and Meta-Analysis (PRISMA) guidelines have been followed when conducting this systematic review (Supplementary File 1) [10, 11]. A protocol for this systematic review was published in *BMJ Paediatrics Open* [12].

Search Strategy

A search was conducted using three search strings: 1) structural congenital anomalies, 2) LMICs, 3) antenatal diagnosis using ultrasound (Supplementary File 2). Using the Ovid program, an electronic database search was conducted on MEDLINE, EMBASE, PubMed, and the Cochrane Library. These searches were filtered to only include studies with human subjects. An example of the search in MEDLINE can be found in Supplementary File 3. Only fetuses with a structural congenital anomaly as listed in search string 1 were included. Only studies from LMICs were included; these were limited to the English language. Studies with less than five patients were excluded. A further search was conducted on the WHO website to identify relevant grey literature, particularly related to antenatal ultrasound policy. The following terms were searched in the WHO Reproductive Health Library: ultrasound, ultrasonography, congenital anomalies, congenital abnormalities, congenital anomaly, congenital abnormality, birth defect, antenatal detection, prenatal detection, antenatal diagnosis, and prenatal diagnosis. Following the search of each term, the results were expanded using a snowball strategy to optimize the inclusion of all relevant data.

Study Design

All forms of evidence-based research were included. This includes systematic reviews, meta-analyses, randomized controlled trials, descriptive observational studies, case-control studies, cohort studies, and case series.

Methodological Quality

Although the researchers intended to use the Cochrane Risk of Bias for Non-Randomized Studies of Interventions (ROBINS-I) and the revised tool to assess Risk of Bias in Randomized Trials (RoB) V.2.0. to evaluate methodological quality, the majority of studies included in this systematic review were not interventional studies. Overall, the data was heterogenous and descriptive in nature, which was not suitable for existing quality assessment tools.

Study Screening

Confidential: For Review Only

References produced from the search results were added to EndNote X8 and duplicates were removed. The articles were then uploaded to Covidence and screened in duplicate. Articles that did not meet the study criteria were removed.

Data Extraction and Synthesis

Data extraction was undertaken by the principal investigator. The data extraction table can be found in Supplementary File 4. The primary analysis focused on the availability and effectiveness of antenatal ultrasound for structural congenital anomalies. Secondary outcomes included neonatal morbidity and mortality, termination rates, and referral rates for further antenatal care. The results are presented in tables and descriptive statistics (range and mean) have been calculated regionally.

Patient and Public Involvement

Given that this is a systematic literature review, there was no patient or public involvement for the collection of data and literature review. Public involvement will be important for prioritizing antenatal ultrasound on the political agenda and improving antenatal care programs. To disseminate the results of this study, international charities and organizations involving structural congenital anomalies will be approached to assist in circulation.

Results

Study Screening

The search produced 4,062 articles. Of these, 745 duplicates were removed. The remaining 3,317 articles underwent abstract and title review by two independent reviewers. Of the 3,317 articles screened, 2,826 were excluded. 491 articles were then reviewed by two independent reviewers in full-text. At this stage, 316 articles were excluded; 73 for non-English language (Supplementary File 5).

175 articles were found to meet all inclusion criteria listed in the search strings. Of these, 78 provided no data relevant to the study and thus were excluded. 97 studies were included in the data extraction phase (Figure 1). Although all LMICs as defined by the World Bank were included in the search, not all countries yielded results in the text screening. 138 LMICs were included in the literature search, however, only 29 countries (21%) had any data that met the inclusion criteria (Figure 2, Supplementary File 6).

It is also notable that the majority of included studies were conducted on an institutional level. Thus, while the data from these studies provides important information from the countries of this review, they are by no means a representative sample of an entire country or even an entire city. Each article also varied widely in the information it provided, ranging from antenatal detection rates to policy analysis. Given the heterogeneity of data extrapolated from these articles, it was not feasible to perform a meta-analysis.

Percentage of Women Receiving Antenatal Ultrasound

Confidential: For Review Only

Twenty-one studies (12 retrospective and 9 prospective observational studies) in 12 countries provided data on this (Table 1). There was significant variation in the percentage of women receiving antenatal ultrasound scans, ranging from 6.8% in a Tanzanian study to 98.8% in a study from China. The data suggests a particularly low rate of women receiving antenatal ultrasound in Africa, with a median of 50.0% compared to 90.7% in Asia. No studies were conducted in Europe or South America, and only one study was conducted in North America (Jamaica, 98.2%).

Table 1: Percentage of Women Receiving Antenatal Ultrasound

**Author(s)**	**Study Location**	**Study Type**	**Study Population**	**# of Women in Study**	**# of Women Who Received Antenatal Ultrasound (%)**
**Africa**					
de Paul Djientcheu et al., 2008 [13]	Cameroon	Retrospective descriptive observational study	Institutional; patients with NTDs* admitted to neonatology unit	69	27 (39.1%)
Abdur-Rahman et al., 2011 [14]	Nigeria	Retrospective descriptive observational study	Institutional; patients with abdominal wall defects at a tertiary health center in the North-Central geopolitical zone of Nigeria	56	51 (91.1%)
Adeleye et al., 2010 [15]	Nigeria	Prospective cross-sectional study	Institutional; patients presenting with major CNS* anomalies at tertiary hospital	54	43 (79.6%)
Adeleye and Joel-Medewase, 2015 [16]	Nigeria	Retrospective cross-sectional survey	Institutional; patients with CNS defects at a neurosurgeon's practice	151	91 (60.3%)
Bankole et al., 2012 [17]	Nigeria	Prospective descriptive observational study	Institutional; patients presenting with CNS anomalies at tertiary hospital	108	54 (50%)
Idowu and Olawehinmi, 2012 [18]	Nigeria	Prospective descriptive observational study	Institutional; patients presenting with NTDs at tertiary hospital	94	91 (96.8%)
Okafor et al., 2013 [19]	Nigeria	Prospective cohort study	Institutional; patients with PUV* at tertiary hospital	31	22 (71%)
Sekabira and Hadley, 2009 [20]	South Africa	Retrospective descriptive observational study	Institutional; patients with gastroschisis at tertiary hospital	106	25 (23.6%)
Santos et al., 2017 [21]	Tanzania	Prospective descriptive observational study	Institutional; patients with hydrocephalus at tertiary medical facility	125	9 (6.8%)
Wesonga et al., 2016 [22]	Uganda	Prospective cohort study	Institutional; patients with gastroschisis at a tertiary hospital	41	10 (24.4%)
Munjanja et al., 1987 [23]	Zimbabwe	Prospective descriptive observational study	Institutional; all patients delivered at Greater Harare Obstetric Unit	36,514	4,429 (12.1%)
**Total**	**11 studies, 6 countries**	**4 retrospective, 7 prospective observational studies**	**11 institutional**	**37,349**	**4,852 Median: 50% Range: 6.8 - 96.8%**
**Asia**					
Lu et al., 2011 [24]	China	Retrospective cross-sectional study	National; fetuses with NTDs	424	419 (98.8%)
Bhat et al., 2008 [25]	India	Retrospective descriptive observational study	Institutional; patients admitted to NICU* with CDH*	16	11 (68.8%)
Raman et al., 2015 [26]	India	Retrospective descriptive observational study	Institutional; symptomatic patients with congenital cystic lung lesions at tertiary care center	40	6 (15%)
Saha et al., 2009 [27]	India	Retrospective descriptive observational study	Institutional; all deliveries at rural medical college	7,365	6,682 (90.7%)
Sood et al., 1991 [28]	India	Retrospective descriptive observational study	Institutional; patients with NTDs at tertiary hospital	65	44 (67.7%)
Kazmi et al., 2006 [29]	Iran	Prospective descriptive observational study	Institutional; patients referred to tertiary center for myelomeningocele evaluation and management	140	136 (97.1%)
Samadirad et al., 2012 [30]	Iran	Retrospective descriptive observational study	Regional; fetuses with congenital anomalies	639	557 (87.2%)
Ho et al., 2006 [31]	Malaysia	Retrospective cohort study	Regional; births in Kinta District (253 cases with congenital anomalies and 506 control cases)	759	705 (92.9%)
Kitisomprayoonkul and Tongsong, 2001 [32]	Thailand	Prospective descriptive observational study	Institutional; patients with NTDs at tertiary hospital	46	42 (91.3%)

Confidential: For Review Only

**Total**	**9 studies, 5 countries**	**7 retrospective, 2 prospective observational studies**	**6 institutional, 2 regional, 1 national**	**9,494**	**8,602 Median: 90.7% Range: 15 – 98.8%**
**North America**					
Johnson et al., 2011 [33]	Jamaica	Retrospective observational review	Institutional; patients with congenital anomalies at tertiary hospital	55	54 (98.2%)
**Total**	**1 study, 1 country**	**1 retrospective observational study**	**1 institutional**	**55**	**54 Median: N/A Range: N/A**

* CDH: congenital diaphragmatic hernia, CNS: central nervous system, NICU: neonatal intensive care unit, NTD: neural tube defects, PUV: posterior urethral valves.

Effectiveness of Antenatal Ultrasound

Sixty-five studies (46 retrospective and 18 prospective observational studies and a parent survey) in 22 countries provided data on detection rates (Table 2). Detection rates varied widely across studies, from 0% to 100%, with little correlation according to geographical region or type of anomaly. In Africa, the median detection rate was 16.7%, which is low compared to other LMICs, with 34.3% in South America, 34.7% in Asia, and 47.3% in Europe. There was only one study from North America (Jamaica, 77.2%). Of the studies conducted from Africa, 8 of the 15 were in Nigeria and hence may not be representative of the whole region.

Table 2: Effectiveness of Antenatal Ultrasound

Author(s)	Study Location	Study Type	Study Population	# of Women in Study	# of Women Who Received Antenatal Diagnosis (%)
**Africa**					
de Paul Djientcheu et al., 2008 [13]	Cameroon	Retrospective descriptive observational study	Institutional; patients with NTDs* admitted to neonatology unit	27	8 (29.6%)
Sorri and Mesfin, 2015 [34]	Ethiopia	Retrospective cross-sectional study	Multi-center; patients with NTDs at two tertiary hospitals	177	127 (71.8%)
Abdur-Rahman et al., 2011 [14]	Nigeria	Retrospective descriptive observational study	Institutional; patients with abdominal wall defects at a tertiary health center in the North-Central geopolitical zone of Nigeria	56	1 (1.8%)
Adeleye et al., 2010 [15]	Nigeria	Prospective cross-sectional study	Institutional; patients presenting with major CNS* anomalies at tertiary hospital	43	6 (14%)
Adeleye and Joel-Medwase, 2015 [16]	Nigeria	Retrospective cross-sectional survey	Institutional; patients presenting with CNS anomalies at a neurosurgeon's practice	146	26 (17.8%)
Akinmoladun et al., 2015 [35]	Nigeria	Prospective descriptive observational study	Institutional; patients attending clinic for ultrasound screening	16	15 (93.8%)
Amadi and Eghwrudjakpor, 2013 [36]	Nigeria	Retrospective descriptive observational study	Institutional; all patients with encephalocele at tertiary hospital	17	5 (29.4%)
Bankole et al., 2012 [17]	Nigeria	Prospective descriptive observational study	Institutional; patients presenting with CNS anomalies at tertiary hospital	108	0 (0%)
Idowu and Olawehinmi, 2012 [18]	Nigeria	Prospective descriptive observational study	Institutional; patients presenting with NTDs at tertiary hospital	91	23 (25.3%)
Okafor et al., 2013 [19]	Nigeria	Prospective cohort study	Institutional; patients with PUV* at tertiary hospital	31	2 (6.5%)
Choopa et al., 2017 [37]	South Africa	Retrospective descriptive observational study	Institutional; patients with PUV at pediatric nephrology unit	60	10 (16.7%)
Sekabira and Hadley, 2009 [20]	South Africa	Retrospective descriptive observational study	Institutional; patients with gastroschisis at tertiary hospital	106	13 (12.3%)
Chanoufi et al., 2004 [38]	Tunisia	Retrospective case series (6 cases)	Institutional; cases of acardiac twins at maternity center	6	1 (16.7%)
Wesonga et al., 2016 [22]	Uganda	Prospective cohort study	Institutional; patients with gastroschisis at a tertiary hospital	41	1 (2.4%)
Munjanja et al., 1987 [23]	Zimbabwe	Prospective descriptive observational study	Institutional; patients with congenital anomalies at obstetrical unit	91	46 (50.5%)
**Total**	**15 studies, 7 countries**	**8 retrospective, 7 prospective observational studies**	**14 institutional, 1 multi-center**	**1,016**	**284 Median: 16.7% Range: 0 – 93.8%**
**Asia**					
Deng et al., 2014 [39]	China	Retrospective cross-sectional study	National; patients with omphalocele as reported in Chinese national birth defects monitoring network 1996-2006	827	322 (38.9%)
Hong et al., 2010 [40]	China	Retrospective cohort study	Multi-center; patients with gastroschisis	17	3 (17.6%)
Liao et al., 2016 [41]	China	Retrospective descriptive observational study	Institutional; patients with limb abnormalities at maternity and child health hospital	36	28 (77.8%)
Liu et al., 2017 [42]	China	Retrospective cross-sectional study	Institutional; patients with congenital anomalies at a tertiary hospital	233	71 (30.5%)

Confidential: For Review Only

Lu et al., 2011 [24]	China	Retrospective cross-sectional study	National; patients with NTDs	424	361 (85.1%)
Shi et al., 2006 [43]	China	Retrospective descriptive observational study	Institutional; cases of conjoined twins at tertiary hospital	7	4 (57.1%)
Weng et al., 2016 [44]	China	Retrospective descriptive observational study	Institutional; patients with congenital choledochal cyst at specialty women's hospital	21	19 (90.5%)
Bhat et al., 2008 [25]	India	Retrospective descriptive observational study	Institutional; patients admitted to NICU* with CDH*	16	4 (25%)
Kumar et al., 2008 [45]	India	Retrospective descriptive observational study	Institutional; symptomatic patients with congenital bronchopulmonary anomalies	25	2 (8%)
Raman et al., 2015 [26]	India	Retrospective descriptive observational study	Institutional; symptomatic patients with congenital cystic lung lesions at tertiary care center	40	3 (7.5%)
Rattan et al., 2017 [46]	India	Retrospective descriptive observational study	Institutional; patients operated on for esophageal atresia and tracheoesophageal fistula at a tertiary care center	693	63 (9.1%)
Sanghvi et al., 1998 [47]	India	Prospective descriptive observational study	Institutional; patients with renal anomalies at tertiary center	125	65 (52%)
Sarin et al., 2012 [48]	India	Retrospective case series (18 cases)	Institutional; patients with duodenal webs at tertiary hospital in India	18	2 (11.1%)
Sharada et al., 2014 [49]	India	Retrospective descriptive observational study	Institutional; patients diagnosed with unilateral multicystic dysplastic kidney at tertiary hospital	47	34 (72.3%)
Singh et al., 2009 [50]	India	Retrospective descriptive observational study	Institutional; patients with unilateral multicystic dysplastic kidney at tertiary center	22	12 (54.5%)
Solanki et al., 2013 [51]	India	Retrospective case series (6 cases)	Institutional; patients diagnosed with crossed fused renal ectopia at tertiary hospital	6	1 (16.7%)
Kazmi et al., 2006 [29]	Iran	Prospective descriptive observational study	Institutional; patients referred to tertiary center for myelomeningocele evaluation and management	136	33 (24.3%)
Mirshemirani et al., 2013 [52]	Iran	Retrospective descriptive observational study	Institutional; patients treated for PUV at a tertiary hospital	98	20 (20.4%)
Shahkar et al., 2016 [53]	Iran	Retrospective descriptive observational study	Institutional; patients with congenital pulmonary mass at a tertiary hospital	47	10 (21.3%)
Ho et al., 2006 [31]	Malaysia	Retrospective cohort study	Regional; births in Kinta District (253 cases with congenital anomalies and 506 control cases)	252	37 (14.7%)
Munim et al., 2013 [54]	Pakistan	Retrospective cohort study	Institutional; patients with diaphragmatic hernia at tertiary hospital	65	41 (63.1%)
Kitisomprayoonkul and Tongsong, 2001 [32]	Thailand	Prospective descriptive observational study	Institutional; patients with NTDs at tertiary hospital	42	42 (100%)
Pitukkijronnakorn et al., 2009 [55]	Thailand	Prospective cross-sectional study	Institutional; patients diagnosed with major congenital anomalies at tertiary hospital	316	144 (45.6%)
Srisupundit et al., 2006 [56]	Thailand	Prospective descriptive observational study	Institutional; patients undergoing antenatal ultrasound at a university teaching hospital in Chiang Mai	34	24 (70.6%)
**Total**	**24 studies, 6 countries**	**19 retrospective, 5 prospective observational studies**	**20 institutional, 1 multi-center, 1 regional, 2 national**	**3,547**	**1,345 Median: 34.7% Range: 7.5 – 100%**
**Europe**					
Iliescu et al., 2013 [57]	Romania	Prospective descriptive observational study	Multi-center; patients at two institutions with major congenital anomalies	76	74 (97.4%)
Ognean et al., 2017 [58]	Romania	Retrospective case series (7 cases)	Institutional; patients with esophageal atresia at a tertiary center	7	0 (0%)
Tarca and Aprodu, 2014 [59]	Romania	Retrospective descriptive observational study	Institutional; patients with omphalocele at tertiary hospital	105	14 (13.3%)
Tarca and Aprodu, 2013 [60]	Romania	Retrospective descriptive observational study	Institutional; patients with gastroschisis at tertiary hospital	54	9 (16.7%)
Tarca et al., 2015 [61]	Romania	Retrospective descriptive observational study	Institutional; patients with gastroschisis at tertiary hospital	114	13 (11.4%)
Tudorache et al., 2014 [62]	Romania	Retrospective descriptive observational study	Institutional; patients with cases of left-sided CDH at tertiary hospital	21	11 (52.4%)
Postoev et al., 2015 [63]	Russia	Retrospective cross-sectional study	Regional; patients with congenital anomalies in the Kola Peninsula (data from two birth defect registries)	232	81 (34.9%)
Aygun et al., 2013 [64]	Turkey	Retrospective descriptive observational study	Institutional; patients with NTDs at tertiary hospital	100	72 (72%)
Dane et al., 2007 [65]	Turkey	Prospective descriptive observational study	Institutional; fetuses with incurable congenital anomalies and curable severe congenital anomalies at a training and research hospital	24	23 (95.8%)
Orgul et al., 2018 [66]	Turkey	Retrospective descriptive observational study	Institutional; patients with gastrointestinal tract malformations at a university children's hospital	56	34 (60.7%)
Oztekin et al., 2009 [67]	Turkey	Prospective descriptive observational study	Institutional; patients with a major structural congenital anomaly at an obstetrics and gynecology teaching hospital	21	19 (90.5%)
Sahinoglu et al., 2007 [68]	Turkey	Retrospective case series (6 cases)	Institutional; patients with limb body wall complex at a women and children's research hospital	6	5 (83.3%)
Tabel et al., 2010 [69]	Turkey	Prospective descriptive observational study	Institutional; patients with kidney or urinary tract anomalies at a university hospital	76	32 (42.1%)
Taskapilioglu et al., 2014 [70]	Turkey	Retrospective descriptive observational study	Institutional; patients with open spina bifida at tertiary center	78	26 (33.3%)
**Total**	**14 studies, 3 countries**	**10 retrospective, 4 prospective observational studies**	**12 institutional, 1 multi-center, 1 regional**	**970**	**413 Median: 47.3% Range: 0-97.4%**
**North America**					

Confidential: For Review Only

Johnson et al., 2012 [33]	Jamaica	Retrospective descriptive observational study	Institutional; patients with congenital anomalies at tertiary hospital	57	44 (77.2%)
**Total**	**1 study, 1 country**	**1 retrospective observational study**	**1 institutional**	**57**	**44 Median: N/A Range: N/A**
**South America**					
Campana et al., 2010 [71]	Argentina, Brazil, Chile, and Venezuela	Prospective descriptive observational study	Multi-country; patients with congenital anomalies in 18 Latin American hospitals	812	457 (56.3%)
Germani et al., 2011 [72]	Argentina	Retrospective descriptive observational study	Institutional; patients with choledochal cyst at a private hospital	12	4 (33.3%)
Wyszynski et al., 2003 [73]	Argentina	Survey	Institutional; patients with nonsyndromic oral cleft (collected from parents' survey data)	165	7 (4.2%)
Carvalho et al., 2002 [74]	Brazil	Prospective cohort study	Institutional; patients with major congenital anomalies at a tertiary hospital	130	93 (71.5%)
Luiza et al., 2013 [75]	Brazil	Retrospective cross-sectional study	Institutional; patients with orofacial cleft at a specialized society attending to cleft patients	168	7 (4.2%)
Tannuri et al., 2011 [76]	Brazil	Retrospective descriptive observational study	Multi-center; patients with gastroschisis at three tertiary centers	163	134 (82.2%)
Vilela et al., 2001 [77]	Brazil	Retrospective cross-sectional study	Institutional; patients with gastroschisis at a tertiary hospital	31	10 (32.3%)
Correa et al., 2014 [78]	Colombia	Retrospective case-control study	City-wide; data from Bogota Congenital Malformations Surveillance Program	167	82 (49.1%)
de Rovetto et al., 2010 [79]	Colombia	Retrospective descriptive observational study	City-wide; patients with congenital renal agenesis at centers in Cali, Colombia	38	8 (21.1%)
Rosselli et al., 2015 [80]	Colombia	Retrospective descriptive observational study	City-wide; patients with congenital talipes equinovarus in Bogota, Colombia	178	61 (34.3%)
Saldarriaga et al., 2014 [81]	Colombia	Retrospective cross-sectional study	City-wide; patients with congenital anomalies diagnosable by antenatal ultrasound in NICUs of Cali, Colombia	217	117 (53.9%)
**Total**	**11 studies, 5 countries**	**8 retrospective, 2 prospective observational studies, 1 survey**	**5 institutional, 1 multi-center, 4 city-wide, 1 multi-country**	**2,078**	**980 Median: 34.3% Range: 4.2-82.2%**

* CDH: congenital diaphragmatic hernia, CNS: central nervous system, NICU: neonatal intensive care unit, NTD: neural tube defects, PUV: posterior urethral valves.

Training of Personnel Performing Ultrasound Examination

Fifteen of the studies detailed the training of the personnel providing the ultrasound scans (Table 3). Several of the included studies mentioned that the scans were performed by 'experienced sonographers,' but provided little detail as to the actual level of training of these providers. This makes it difficult to accurately assess the role that training may have in the detection of structural congenital anomalies.

Table 3: Training of Personnel Performing Ultrasound Examination

Author(s)	Study Location	# of Anomalies Detected (%)	Information about Training of Personnel Performing Antenatal Ultrasound Examinations
**Africa**			
Adeleye et al., 2010 [15]	Nigeria	6/43 (14%)	Radiologists performed 5% of cases; medical doctors performed 11%; unknown training/status performed 84% of cases
Adeleye and Joel-Medewase, 2015 [16]	Nigeria	26/146 (17.8%)	22% of ultrasounds performed by a radiologist; sonographers in rest of the cases were personnel with unknown training; authors noted that prenatal diagnosis was significantly more likely in cases where sonographer was certified radiologist
Akinmoladun et al., 2015 [35]	Nigeria	15/16 (93.8%)	A consultant radiologist trained in fetal anomaly scanning performed all the scans (the authors note that this radiologist received extensive training at a renowned center in the UK)
Idowu and Olawehinmi, 2012 [18]	Nigeria	23/91 (25.3%)	Authors noted that low diagnosis "may be due to the high prevalence of the test being done by non-specialist (untrained radiologist) in our environment"
Wesonga et al., 2016 [22]	Uganda	1/41 (2.4%)	Performed by ultrasound technicians holding a diploma; no further information about diploma
**Asia**			
Liao et al., 2016 [41]	China	28/36 (77.8%)	Ten certified physicians participated in the study protocol, each of whom has more than 5 years of experience in fetal sonography
Xie et al., 2009 [82]	China	Not specified	2 sonographers – 1 with 10 years of experience in obstetric sonography and the other with 22 years of experience
Sanghvi et al., 1998 [47]	India	65/125 (52%)	Performed by "experienced sonologists"

Confidential: For Review Only

Ghavami and Abedinzadeh, 2011 [83]	Iran	Not specified	Performed by “two expert operators”
Pitukkijronnakorn et al., 2009 [55]	Thailand	144/316 (45.6%)	All scans were performed by an obstetrician who was trained as a level one ultrasonography; in cases of uncertain abnormal findings, the women were reviewed by a level two obstetrician with repeated scans
**Europe**			
Iliescu et al., 2013 [57]	Romania	74/76 (97.4%)	Scans performed by obstetricians specializing in prenatal diagnosis (including the anomaly scan and echocardiography) who had held accreditation for the 11 – 14-week assessment for at least 5 years prior to the start of the study period
Dane et al., 2007 [65]	Turkey	23/24 (95.8%)	2 operators with approximately 6 years & 2 years of experience in gestational ultrasound scanning
Kutuk et al., 2017 [84]	Turkey	Not specified	All ultrasound scans performed by “two experienced maternal-fetal specialists”
Oztekin et al., 2009 [67]	Turkey	19/21 (90.5%)	All scans performed by the same experienced radiologist
**North America**			
Johnson et al., 2011 [33]	Jamaica	44/57 (77.2%)	8 OB/GYN residents in training for at least 2 years

Morbidity and Mortality Outcomes

Only four studies produced any data comparing the morbidity and mortality outcomes between neonates with an antenatal diagnosis versus neonates with a postnatal diagnosis (Table 4). In the study that addressed gastroschisis, outcomes were more favorable for neonates who had received an antenatal diagnosis compared to those who had not (20% vs. 66.7% mortality). This was not the case for the study which addressed congenital diaphragmatic hernia (CDH), however this may reflect that more severe forms of anomalies are easier to detect antenatally.

Table 4: Morbidity and Mortality Outcomes

Author(s)	Study Location	Patient Population	Mortality with Antenatal Diagnosis	Mortality without Antenatal Diagnosis	Morbidity with Antenatal Diagnosis	Morbidity without Antenatal Diagnosis
**Asia**						
Bhat et al., 2008 [25]	India	Institutional; patients with CDH*	4/4 (100%)	3/12 (25%)	N/A	4/9 (44.4%)
**Europe**						
Savran et al., 2015 [85]	Turkey	Institutional; patients with duodenal atresia	0/9 (0%)	0/6 (0%)	0/9 (0%)	1/6 (16.7%)
**North America**						
Johnson et al., 2011 [33]	Jamaica	Institutional; patients with congenital anomalies	19/44 (43.2%)	5/13 (38.5%)	11/29 (37.9%)	9/12 (75%)
**South America**						
Vilela et al., 2001 [77]	Brazil	Institutional; patients with gastroschisis	2/10 (20%)	14/21 (66.7%)	Not specified	Not specified

*CDH: congenital diaphragmatic hernia

Termination Rates

Twenty-five studies (20 retrospective and 4 prospective observational studies and an ethnographic study) in 15 countries provided data on termination rates (Table 5). Termination rates were highly varied, with a median of 17.1% in Africa, 34.4% in Asia, 50.2% in Europe, and 62.4% in South America (range 0% to 98.3%). Only one study from Africa evaluated termination rates for lethal anomalies and had just five participants. Thus, it is difficult to compare the termination rate of lethal anomalies with other regions, which contain such data.

Confidential: For Review Only

Termination rates can also be affected by the type of anomaly, the severity, the gestational age at diagnosis, the national termination policies and the cultural appropriateness of termination. Hence, while these termination rates offer valuable insight, it is necessary to also consider the underlying determinants that have impacted termination decisions.

Table 5: Termination Rates

Author(s)	Study Location	Study Type	Study Population	# of Fetuses	# of Fetuses Terminated (%)
**Africa**					
de Paul Djientcheu et al., 2008 [13]	Cameroon	Retrospective descriptive observational study	Institutional; patients with NTDs*	8	0 (0%)
Shalaby et al., 2016 [86]	Egypt	Retrospective cross-sectional study	Institutional; Patients with urinary anomalies	41	11 (26.8%)
Sorri and Mesfin, 2015 [34]	Ethiopia	Retrospective cross-sectional study	Multi-center; patients with NTDs at two tertiary hospitals	177	13 (7.3%)
Akinmoladun et al., 2015 [35]	Nigeria	Prospective descriptive observational study	Institutional; Patients with lethal congenital anomalies	5	4 (80%)
**Total**	**4 studies, 4 countries**	**3 retrospective, 1 prospective observational studies**	**3 institutional, 1 multi-center**	**231**	**28 Median: 17.1% Range: 0 – 80%**
**Asia**					
Li et al., 2006 [87]	China	Retrospective descriptive observational survey	Regional; patients with NTDs	160	72 (45%)
Lu et al., 2011 [24]	China	Retrospective cross-sectional study	National (data from 20 counties); patients with NTDs	361	355 (98.3%)
Xie et al., 2009 [82]	China	Retrospective descriptive observational study	Institutional; patients with bronchopulmonary sequestration	22	8 (36.4%)
Zhang, 2014 [88]	China	Retrospective descriptive observational study	Institutional; patients with pulmonary sequestration	68	2 (2.9%)
Kashyap et al., 2015 [89]	India	Retrospective descriptive observational study	Institutional; patients with lethal congenital anomalies detected prior to 20 weeks of gestation	103	80 (77.7%)
Kumar et al., 2012 [90]	India	Prospective cohort study	Institutional; patients with severe renal anomalies	55	9 (16.4%)
Kumar et al., 2014 [91]	India	Prospective descriptive observational study	Institutional; patients with renal anomalies	136	12 (8.8%)
Sanghvi, et al., 1998 [47]	India	Prospective descriptive observational study	Institutional; patients with lethal renal anomalies	7	2 (28.6%)
Samadirad et al., 2012 [30]	Iran	Retrospective descriptive observational study	Regional; patients with congenital anomalies	603	201 (33.3%)
Munim et al., 2013 [54]	Pakistan	Retrospective cohort study	Institutional; patients with diaphragmatic hernia	41	6 (14.6%)
Hsieh et al., 2000 [92]	Taiwan	Retrospective descriptive observational study	Institutional; patients with CDH*	31	11 (35.5%)
Jaruratanasirikul et al., 2014 [93]	Thailand	Retrospective cross-sectional study	Regional; patients with NTDs	28	12 (42.9%)
Pitukkijronnakorn et al., 2009 [55]	Thailand	Retrospective cross-sectional study	Institutional; patients with congenital anomalies	316	87 (27.5%)
Gammeltoft et al., 2008 [94]	Vietnam	Ethnographic study	Institutional; patients with congenital anomalies	30	17 (56.7%)
**Total**	**14 studies, 7 countries**	**10 retrospective, 3 prospective observational studies; 1 ethnographic study**	**10 institutional, 3 regional, 1 national**	**1,961**	**874 Median: 34.4% Range: 2.9 – 98.3%**
**Europe**					
Tudorache et al., 2014 [62]	Romania	Retrospective descriptive observational study	Institutional; patients with severe CDH diagnosed in the second trimester of pregnancy	6	4 (66.7%)
Aygun et al., 2013 [64]	Turkey	Retrospective descriptive observational study	Institutional; patients with NTDs	72	0 (0%)
Oztarhan et al., 2010 [95]	Turkey	Retrospective cohort study	Institutional; patients with lethal congenital anomalies	1,906	640 (33.6%)

Confidential: For Review Only

Sahinoglu et al., 2007 [68]	Turkey	Retrospective case series (6 cases)	Institutional; patients with body wall complex	6	4 (66.7%)
**Total**	**4 studies, 2 countries**	**4 retrospective observational studies**	**4 institutional**	**1,990**	**648 Median: 50.2% Range: 0 – 66.7%**
**North America**					
Johnson et al., 2012 [33]	Jamaica	Retrospective descriptive observational study	Institutional; patients with congenital anomalies	44	10 (22.7%)
**Total**	**1 study, 1 country**	**1 retrospective observational study**	**1 institutional**	**44**	**10 Median: N/A Range: N/A**
**South America**					
Brizot et al., [96]	Brazil	Retrospective descriptive observational study	Institutional; pairs of conjoined twins in which surgical separation was impossible and the condition lethal	36	30 (83.3%)
Pelizzari et al., 2015 [97]	Brazil	Retrospective cohort study	Institutional; patients with anencephaly	29	12 (41.4%)
**Total**	**2 studies, 1 country**	**2 retrospective observational studies**	**2 institutional**	**65**	**42 (64.6%) Median: 62.4% Range: 41.4 – 83.3%**

* CDH: congenital diaphragmatic hernia, NTD: neural tube defects.

Referral Rates for Further Antenatal Care and Management Planning

No studies addressed this issue.

Policy Data

Thirteen articles provided policy data from 10 countries (Table 6). Only two studies, in India and Brazil, mentioned national policies for antenatal ultrasound simply stating that they didn't exist. Termination of pregnancy remains a highly sensitive topic in many communities, which is reflected in the variation of policies across the globe.

Table 6: Policy Data

Author(s)	Study Location	Policy Data about Antenatal Screening and/or Termination Legislation
**Africa**		
Oloyede and Oyedele, 2008 [98]	Nigeria	In Nigeria, the two existing pregnancy termination laws are restrictive in nature. However, termination is often done when a fetus is malformed on the grounds of preserving the mental health of the women.
**Asia**		
Acharya et al., 2013 [99]	India	India has no definite policy for the ultrasound screening for fetal abnormalities and antenatal diagnostic techniques. The law in India says that those who meet the criteria of the PCPNDT* Act can perform an ultrasound scan and they must be sufficiently trained & registered with the proper authority.
Neogi, 2010 [100]	India	Abortion was legalized in India in 1971 under the Medical Termination of Pregnancy Act. It permits abortion by 1 doctor before 12 weeks of gestation but if the duration of pregnancy is more than 12 weeks but less than 20 weeks, then the opinion of 2 medical practitioners is necessary to terminate the pregnancy.
Phadke et al., 2011 [101]	India	In India, the Medical Termination of Pregnancy Act of 1971 (The MTP Act, No. 34 of 1971) does not allow pregnancy termination on grounds of fetal abnormality after 20 weeks of gestation.
Ranji and Dykes, 2012 [102]	Iran	According to the regulations of the Iranian Ministry of Health, ultrasound examinations during pregnancy must be carried out by radiologists.
Arawi and Nassar, 2011 [103]	Lebanon	Lebanese law stipulates that pregnancy termination is forbidden except when the pregnancy endangers the health of the mother and only after consulting with two physicians.
Senanayake and de Silva, 2001 [104]	Sri Lanka	In Sri Lanka, it is illegal to terminate a pregnancy even in cases of early diagnosis (11-14 weeks of gestation).
**Europe**		
Hostiuc et al., 2013 [105]	Romania	According to Romanian law, abortion over 14 weeks is only allowed in cases of severe congenital defects and pregnancies that threaten the life of the mother.
Oztarhan et al., 2010 [95]	Turkey	Turkish law authorizes pregnancy termination voluntarily until 10 weeks in unwanted pregnancies and at any gestational age for medical indications that are considered potentially life-threatening to the mother or fetus. The legal process requires one obstetrician and one physician to agree that pregnancy termination is valid for a medical reason.
**North America**		

Confidential: For Review Only

Lisker et al., 2006 [106]	Mexico	Pregnancy termination is illegal in most Mexican States, except in the case of rape or if the mother's life is at risk by the continuation of pregnancy. In Mexico City and 12 of the 31 states, the presence of a severe congenital anomalies has become a justification for the legal termination of pregnancy.
**South America**		
Groisman et al., 2013 [107]	Argentina	According to the Argentinian criminal code, termination of pregnancy is illegal unless the pregnancy is threat to woman's life or pregnancy is consequence of rape of a mentally retarded woman. In the city of Buenos Aires, it is legal to induce labor after 24 weeks of gestational age in case of anencephaly and other lethal conditions.
Benute et al., 2012 [108]	Brazil	Brazilian law does not include lethal fetal malformation as an indication for pregnancy termination, however, many couples ask a court for permission to terminate a pregnancy on the grounds that it is the option which creates less suffering.
Mirlesse and Ville, 2013 [109]	Brazil	Ultrasound is not explicitly recommended by Brazilian authorities. Brazilian legislation considers termination of pregnancy to be a crime (except in cases of rape or pregnancies which risk the mother's life). However, for lethal fetal malformations, it is possible to apply to the courts for an exceptional authorization to abort. These requests require a medical referral center to perform an ultrasound and prepare a very detailed file.

*PCPNDT: Pre-Conception and Pre-Natal Diagnostic Techniques Act

Policy Assessment

WHO guidelines recommend the need for one antenatal ultrasound scan prior to 24 weeks gestation [110]. Studies suggest that the ideal detection window for structural congenital anomalies is 19-21 weeks of gestation [111]. At this point, it is possible to detect most structural congenital anomalies and is within the legal termination timeframe for many countries. Of note, many of the congenital anomalies detected antenatally in this review were not diagnosed until after 24 weeks gestation. This may be explained by the timing of the first antenatal ultrasound and/or the level of ultrasonographer training. The WHO recommends that ultrasound trainees receive at least 3-6 months of training, culminating in 300-500 ultrasound examinations [112]. A recent study found that the majority of ultrasound providers in LMICs do not have the minimum training as set by the WHO [113]. Hence, many ultrasound practitioners in LMICs may not have the skills to accurately detect congenital anomalies.

Discussion

The median proportion of women receiving an antenatal ultrasound varied from 50.0% in Africa to 90.7% in Asia. It is likely these are an overestimate of the true population rates considering that the majority of studies were undertaken at tertiary facilities. To fully understand what percentage of women receive antenatal ultrasound, further studies must be conducted at a population level, regionally and nationally, rather than at an institutional level. Research must also address the availability and accessibility of antenatal ultrasound and the barriers to receiving a scan.

Detection rates varied widely, from 0% to 100%, with the lowest reported rates in Africa (16.7%). Low detection rates may be because ultrasound providers did not specifically screen for congenital anomalies. Currently, many women in LMICs receive antenatal ultrasound examinations for the assessment of pregnancy progress, such as to determine the gestational age, sex of the baby, and to hear the heartbeat, rather than to detect anomalies. This is in contrast to HICs where the majority of women receive an anomaly scan around 20 weeks gestation [111]. Another possible reason for low detection may be the training level of the ultrasound provider; there appears to be a trend between higher levels of training and higher detection rates. This warrants further investigation to determine minimum training requirements and associated policy and monitoring.

Confidential: For Review Only

The First Look Study is an important randomized controlled trial which assessed the use of antenatal ultrasound in the Democratic Republic of the Congo, Guatemala, Kenya, Pakistan, and Zambia [114]. Although 95% of women in their intervention group received antenatal ultrasound scans (compared to 43% in the control group) and detection rates improved, hospital delivery did not increase for complicated pregnancies and thus there was no resultant improvement in neonatal mortality. In an additional survey by the same group, barriers to referral attendance included cost, distance, and lack of transportation [115]. For women who did attend referral, barriers included not being connected to the correct provider and being told to return at a later time [115]. The authors conclude that without improvement of subsequent care, antenatal ultrasound offered limited impact [114]. Hence, to reduce neonatal morbidity and mortality, detection of an anomaly must be followed by referral for antenatal counselling and delivery at a tertiary center which can provide the necessary surgical care at birth where required. It is also necessary to offer termination for conditions which are incompatible with life, where culturally acceptable.

Hence, it is vital to further investigate barriers to accessing delivery at a pediatric surgery center once a congenital anomaly has been diagnosed and ways to address these barriers. Future studies must also investigate the effects of both antenatal diagnosis and delivery at a tertiary pediatric surgery center on morbidity and mortality outcomes in the LMIC setting; this systematic review highlighted a severe lack of such vital data. The recently completed Global PaedSurg study may provide such data for a selection of common gastrointestinal congenital anomalies globally, which collectively have a particularly high mortality in the LMIC setting [116]. As anomaly screening rates increase in LMICs, it will be also be important to monitor termination rates along with reasons for termination, to ensure the benefits of antenatal diagnosis are optimized both clinically and ethically.

To address some of these issues, there is a need for global collaboration. This collaboration must include members from multi-disciplinary backgrounds, including policymakers, obstetricians, neonatologists, pediatric surgeons, midwives, and allied professionals. The Global Initiative for Children's Surgery (GICS) is a multidisciplinary collaborative whose aim is to improve health outcomes for children requiring surgery in LMICs [117]. This initiative connects the expertise of providers in LMICs and HICs and is committed to expanding the representation and leadership of stakeholders in LMICs. GICS has recently created a congenital anomalies working group, which is planning some of the following projects: 1) To produce guidelines on how to diagnose structural congenital anomalies via antenatal ultrasound; 2) To produce referral and management guidelines following an antenatal diagnosis; and 3) To produce information sheets that can be translated into various languages for parents that contain details about common congenital anomalies. Global collaboration must also extend to the level of the WHO and the Ministries of Health to ensure that recommendations are detailed in policy and implemented into practice.

If these steps are taken, improvements in neonatal health outcomes may be realized, as seen in HICs. Early detection and immediate surgical intervention of congenital anomalies, such as gastroschisis, has been effective in significantly reducing neonatal mortality in HICs [5]. The mortality of gastroschisis has significantly improved in HICs over a period of 50 years, to less than 5% today [5]. This can be attributed to improvements in accurately diagnosing gastroschisis antenatally, monitoring the fetus for complications, and planning for delivery at a facility with

Confidential: For Review Only

pediatric surgeons available [118]. Similar trends have been seen for other congenital anomalies in HICs such as intestinal atresia, congenital diaphragmatic hernia, omphalocele, esophageal atresia and posterior urethral values. By understanding the current role of antenatal ultrasound in LMICs and the barriers to detection, referral, and management of structural congenital anomalies, appropriate interventions can be implemented to help improve outcomes.

Although this systematic review provides useful data, it is also important to note a few of the limitations of the study. First, only articles in English were included in this systematic review, which may exclude other relevant studies. This study used four electronic databases for the search. The expansion of search databases to include African Journals Online, Scielo, and Regional WHO's African Index Medicus may have provided other studies from LMICs that were not indexed in the search engines used. It is vital to include these databases in future research focusing on LMICs. Furthermore, it is important to note that antenatal ultrasound has further diagnostic capabilities, such as detecting abnormal growth or improper placental position and this review only focused on the detection of structural congenital anomalies. Further studies could also include other uses of antenatal ultrasound for improving neonatal and indeed maternal health outcomes. Finally, the policy data in this study represents what was accurate when the studies were published. Some of the policy data may now be out-of-date.

Conclusion

The data from this review suggests that the percentage of women in LMICs who receive an antenatal ultrasound examination varies considerably and is particularly low in sub-Saharan African countries. Even when antenatal ultrasound scans are performed, accurate detection rates are often very low. The level of training (and the type of training) of the sonographer may be indicative of the accuracy of diagnosis. Only four studies delineated the morbidity and mortality outcomes amongst neonates with an antenatal diagnosis compared to postnatal diagnosis. Hence, although the benefits of antenatal ultrasound are widely documented in HICs, data is severely lacking in LMICs. It is clear that the use of antenatal ultrasound in LMICs is not maximized to its highest potential.

Acknowledgements

We would like to thank the library services at King's College London for help with the systematic review process.

Contributors

NW conceived the idea for this study. NW, AK, and SG devised the study design. SG, SSB, NAA, LIN, MTB, and NW performed the literature review. SG drafted the manuscript with significant contributions from NW and AK. All authors reviewed and approval the final submitted manuscript.

Funding

Confidential: For Review Only

NW receives funding from the Wellcome Trust to undertake a Clinical PhD in Global Health at King's Centre for Global Health and Health Partnerships, King's College London (Funder Reference: 203905/Z/16/Z).

Competing Interests

The authors have no competing interests to declare.

References

1. Sitkin, N.A., et al., *Congenital anomalies in low- and middle-income countries: the unborn child of global surgery.* World J Surg, 2015. **39**(1): p. 36-40.
2. Farmer, D., et al., *Surgical Interventions for Congenital Anomalies*, in *Essential Surgery: Disease Control Priorities, Third Edition (Volume 1)*, H.T. Debas, et al., Editors. 2015: Washington (DC).
3. WHO. *WHO Fact Sheets - Congenital Anomalies* 2015 May 12, 2018]; Available from: http://www.who.int/news-room/fact-sheets/detail/congenital-anomalies.
4. Badrinath, R., et al., *Outcomes and unmet need for neonatal surgery in a resource-limited environment: estimates of global health disparities from Kampala, Uganda.* J Pediatr Surg, 2014. **49**(12): p. 1825-30.
5. Bradnock, T.J., et al., *Gastroschisis: one year outcomes from national cohort study.* BMJ, 2011. **343**: p. d6749.
6. Wright, N.J., A. Zani, and N. Ade-Ajayi, *Epidemiology, management and outcome of gastroschisis in Sub-Saharan Africa: Results of an international survey.* Afr J Paediatr Surg, 2015. **12**(1): p. 1-6.
7. Shah, S., et al., *Perceived barriers in the use of ultrasound in developing countries.* Crit Ultrasound J, 2015. **7**(1): p. 28.
8. UN. *Goal 3: Ensure healthy lives and promote well-being for all at all ages*. Sustainable Development Goals 2015; Available from: https://www.un.org/sustainabledevelopment/health/.
9. WHO, *Sixty-Third World Health Assemby Report on Birth Defects*. 2010, World Health Organization: Geneva, Switzerland.
10. Liberati, A., et al., *The PRISMA statement for reporting systematic reviews and meta-analyses of studies that evaluate health care interventions: explanation and elaboration.* J Clin Epidemiol, 2009. **62**(10): p. e1-34.
11. Moher, D., et al., *Preferred reporting items for systematic reviews and meta-analyses: the PRISMA Statement.* Open Med, 2009. **3**(3): p. e123-30.
12. Goley, S., et al., *Investigating the use of ultrasonography for the antenatal diagnosis of structural congenital anomalies in low-income and middle-income countries: systematic review protocol* BMJ Paediatrics Open, 2019. **3**(1).
13. de Paul Djientcheu, V., et al., *Management of neural tube defects in a Sub-Saharan African country: the situation in Yaounde, Cameroon.* Journal of the Neurological Sciences, 2008. **275**(1-2): p. 29-32.
14. Abdur-Rahman, L.O., N.A. Abdulrasheed, and J.O. Adeniran, *Challenges and outcomes of management of anterior abdominal wall defects in a Nigerian tertiary hospital.* Afr J Paediatr Surg, 2011. **8**(2): p. 159-63.

Confidential: For Review Only

15. Adeleye, A.O., M.D. Dairo, and K.G. Olowookere, *Central nervous system congenital malformations in a developing country: issues and challenges against their prevention.* Childs Nervous System, 2010. **26**(7): p. 919-24.
16. Adeleye, A.O. and V.I. Joel-Medewase, *Awareness and uptake of measures for preventing CNS birth defects among mothers of affected children in a sub-Saharan African neurosurgeon's practice.* Childs Nerv Syst, 2015. **31**(12): p. 2311-7.
17. Bankole, O.B., S.O. Arigbabu, and O.O. Kanu, *Spinal neural tube defects in Lagos University Teaching Hospital, Nigeria.* Nigerian Quarterly Journal of Hospital Medicine, 2012. **22**(1): p. 22-4.
18. Idowu, O.E. and O.S. Olawehinmi, *Surgical congenital central nervous system anomalies in a tropical teaching hospital.* British Journal of Neurosurgery, 2012. **26**(5): p. 726-9.
19. Okafor, H.U., S.O. Ekenze, and S.N. Uwaezuoke, *Posterior urethral valves: determinants of outcome in a developing country.* J Paediatr Child Health, 2013. **49**(2): p. 115-9.
20. Sekabira, J. and G.P. Hadley, *Gastroschisis: a third world perspective.* Pediatric Surgery International, 2009. **25**(4): p. 327-9.
21. Santos, M.M., et al., *Infant hydrocephalus in sub-Saharan Africa: the reality on the Tanzanian side of the lake.* J Neurosurg Pediatr, 2017. **20**(5): p. 423-431.
22. Wesonga, A.S., et al., *Gastroschisis in Uganda: Opportunities for improved survival.* Journal of Pediatric Surgery, 2016. **51**(11): p. 1772-1777.
23. Munjanja, S.P., et al., *Audit of ultrasound scanning: antenatal diagnosis of congenital abnormalities in Harare, Zimbabwe.* East African Medical Journal, 1987. **64**(9): p. 601-5.
24. Lu, Q.B., et al., *Investigation of ultrasound screening efficiency for neural tube defects during pregnancy in rural areas of China.* Public Health, 2011. **125**(9): p. 639-44.
25. Bhat, Y.R., V. Kumar, and A. Rao, *Congenital diaphragmatic hernia in a developing country.* Singapore Med J, 2008. **49**(9): p. 715-8.
26. Raman, V.S., et al., *Congenital cystic lesions of the lungs: The perils of misdiagnosis - A single-center experience.* Lung India, 2015. **32**(2): p. 116-8.
27. Saha, A., et al., *Antenatal detection of renal malformations.* Indian Pediatrics, 2009. **46**(4): p. 346-8.
28. Sood, M., et al., *Neural tubal defects in an east Delhi hospital.* Indian Journal of Pediatrics, 1991. **58**(3): p. 363-5.
29. Kazmi, S.S., et al., *The prenatal ultrasonographic detection of myelomeningocele in patients referred to Children's Hospital Medical Center: a cross sectional study.* Reprod Health, 2006. **3**: p. 6.
30. Samadirad, B., et al., *Congenital anomalies and termination of pregnancy in Iran.* Journal of Pregnancy, 2012. **2012**: p. 574513.
31. Ho, J.J., M.K. Thong, and N.K. Nurani, *Prenatal detection of birth defects in a Malaysian population: estimation of the influence of termination of pregnancy on birth prevalence in a developing country.* Australian & New Zealand Journal of Obstetrics & Gynaecology, 2006. **46**(1): p. 55-7.
32. Kitisomprayoonkul, N. and T. Tongsong, *Neural tube defects: a different pattern in northern Thai population.* Journal of the Medical Association of Thailand, 2001. **84**(4): p. 483-8.

Confidential: For Review Only

33. Johnson, N., et al., *Congenital abnormalities at a tertiary center in Jamaica: an 18-month maternal-fetal medicine experience.* Journal of Maternal-Fetal & Neonatal Medicine, 2012. **25**(6): p. 687-91.
34. Sorri, G. and E. Mesfin, *Patterns of Neural Tube Defects at Two Teaching Hospitals in Addis Ababa, Ethiopia a Three Years Retrospective Study.* Ethiopian Medical Journal, 2015. **53**(3): p. 119-26.
35. Akinmoladun, J.A., et al., *Routine prenatal ultrasound anomaly screening program in a Nigerian university hospital: Redefining obstetrics practice in a developing African country.* Nigerian Medical Journal, 2015. **56**(4): p. 263-7.
36. Amadi, C.E. and P.O. Eghwrudjakpor, *The pattern of distribution of encephalocele in University of Port Harcourt Teaching Hospital--a three year experience.* Niger J Med, 2013. **22**(1): p. 19-23.
37. Choopa, M.S., S.O. Manda, and G. Van Biljon, *Diagnosis, management and outcome of posterior urethral valves in children at a tertiary centre in South Africa.* Pediatric Nephrology, 2017. **32 (9)**: p. 1706.
38. Chanoufi, M.B., et al., *Clinical and anatomic features of acardiac twins.* Medical Principles & Practice, 2004. **13**(6): p. 375-9.
39. Deng, K., et al., *Perinatal mortality in pregnancies with omphalocele: data from the Chinese national birth defects monitoring network, 1996-2006.* BMC Pediatrics, 2014. **14**: p. 160.
40. Hong, L., et al., *Modified silo technique-An easy and effective method to improve the survival rate of neonates with gastroschisis in Shanghai.* European Journal of Obstetrics, Gynecology, & Reproductive Biology, 2010. **148**(1): p. 31-4.
41. Liao, Y.M., et al., *Routine screening for fetal limb abnormalities in the first trimester.* Prenatal Diagnosis, 2016. **36**(2): p. 117-26.
42. Liu, S., Q. Wu, and Z. Chen, *Fetal anomalies detection in China by screening with ultrasound.* Biomedical Research (India), 2017. **28**(11): p. 4891-4896.
43. Shi, C.R., et al., *Surgical management to conjoined twins in Shanghai area.* Pediatr Surg Int, 2006. **22**(10): p. 791-5.
44. Weng, R., et al., *Prenatal diagnosis and prognosis assessment of congenital choledochal cyst in 21 cases.* J Obstet Gynaecol, 2016. **36**(3): p. 324-7.
45. Kumar, B., L.D. Agrawal, and S.B. Sharma, *Congenital bronchopulmonary malformations: A single-center experience and a review of literature.* Annals of Thoracic Medicine, 2008. **3**(4): p. 135-139.
46. Rattan, K.N., J. Singh, and P. Dalal, *Clinical profile and short-term outcome of neonates with esophageal atresia and tracheoesophageal fistula at tertiary care center in a developing country: A 25-year experience.* Journal of Clinical Neonatology, 2017. **6**(4): p. 225-230.
47. Sanghvi, K.P., et al., *Antenatal diagnosis of congenital renal malformations using ultrasound.* J Trop Pediatr, 1998. **44**(4): p. 235-40.
48. Sarin, Y.K., et al., *Duodenal webs: an experience with 18 patients.* J Neonatal Surg, 2012. **1**(2): p. 20.
49. Sharada, S., et al., *Multicystic dysplastic kidney: a retrospective study.* Indian Pediatrics, 2014. **51**(8): p. 641-3.

Confidential: For Review Only

50. Singh, J.K., R.P. Kanojia, and K.L. Narasimhan, *Multicystic dysplastic kidney in children--a need for conservative and long term approach.* Indian Journal of Pediatrics, 2009. **76**(8): p. 809-12.
51. Solanki, S., et al., *Crossed fused renal ectopia: Challenges in diagnosis and management.* J Indian Assoc Pediatr Surg, 2013. **18**(1): p. 7-10.
52. Mirshemirani, A., et al., *Posterior Urethral Valves; A single Center Experience.* Iran J Pediatr, 2013. **23**(5): p. 531-5.
53. Shahkar, L., et al., *An eleven-year review of congenital pulmonary lesions in a referral children's hospital in Tehran.* Archives of Pediatric Infectious Diseases, 2016. **4 (4) (no pagination)**(e32792).
54. Munim, S., et al., *Fetal outcome of cases with diaphragmatic hernia.* Journal of Maternal-Fetal & Neonatal Medicine, 2013. **26**(14): p. 1439-42.
55. Pitukkijronnakorn, S., et al., *The value of mid-trimester routine ultrasonographic screening in antenatal detection of congenital malformations.* Journal of the Medical Association of Thailand, 2009. **92**(6): p. 748-53.
56. Srisupundit, K., et al., *Fetal structural anomaly screening at 11-14 weeks of gestation at Maharaj Nakorn Chiang Mai Hospital.* Journal of the Medical Association of Thailand, 2006. **89**(5): p. 588-93.
57. Iliescu, D., et al., *Improved detection rate of structural abnormalities in the first trimester using an extended examination protocol.* Ultrasound Obstet Gynecol, 2013. **42**(3): p. 300-9.
58. Ognean, M.L., et al., *Esophageal atresia with distal fistula - unusual case series. Considerations related to epidemiological aspects, malformative associations, and prenatal diagnosis.* Rom J Morphol Embryol, 2017. **58**(3): p. 1069-1076.
59. Tarca, E. and S. Aprodu, *Past and present in omphalocele treatment in Romania.* Chirurgia (Bucuresti), 2014. **109**(4): p. 507-13.
60. Tarca, E. and S.G. Aprodu, *Gastroschisis treatment: what are the causes of high morbidity and mortality rates?* Chirurgia (Bucuresti), 2013. **108**(4): p. 516-20.
61. Tarca, E., I. Ciongradi, and S.G. Aprodu, *Birth Weight, Compromised Bowel and Sepsis are the Main Variables Significantly Influencing Outcome in Gastroschisis.* Chirurgia (Bucuresti), 2015. **110**(2): p. 151-6.
62. Tudorache, S., et al., *Prenatal diagnosis and perinatal outcome in congenital diaphragmatic hernia. Single tertiary center report.* Romanian Journal of Morphology & Embryology, 2014. **55**(3): p. 823-33.
63. Postoev, V.A., et al., *Changes in detection of birth defects and perinatal mortality after introduction of prenatal ultrasound screening in the Kola Peninsula (North-West Russia): combination of two birth registries.* BMC Pregnancy & Childbirth, 2015. **15**: p. 308.
64. Aygun, C., et al., *Experience of a tertiary care center on 100 newborns with neural tube defects.* Turk J Pediatr, 2013. **55**(4): p. 359-64.
65. Dane, B., et al., *Ultrasound screening for fetal major abnormalities at 11-14 weeks.* Acta Obstet Gynecol Scand, 2007. **86**(6): p. 666-70.
66. Orgul, G., et al., *Evaluation of pre- and postnatally diagnosed gastrointestinal tract obstructions.* Journal of Maternal-Fetal & Neonatal Medicine, 2018: p. 1-6.
67. Oztekin, O., et al., *Ultrasonographic diagnosis of fetal structural abnormalities in prenatal screening at 11-14 weeks.* Diagn Interv Radiol, 2009. **15**(3): p. 221-5.

Confidential: For Review Only

68. Sahinoglu, Z., et al., *The prenatal diagnosis of cloacal dysgenesis sequence in six cases: can the termination of pregnancy always be the first choice?* Prenatal Diagnosis, 2004. **24**(1): p. 10-6.
69. Tabel, Y., et al., *Ultrasonographic screening of newborns for congenital anomalies of the kidney and the urinary tracts.* Urol J, 2010. **7**(3): p. 161-7.
70. Taskapilioglu, M.O., S. Isik, and E. Korfali, *Outcome of Myelomeningocele: The importance of prenatal diagnosis.* Neurosurgery Quarterly, 2014. **24**(3): p. 214-217.
71. Campana, H., et al., *Prenatal sonographic detection of birth defects in 18 hospitals from South America.* Journal of Ultrasound in Medicine, 2010. **29**(2): p. 203-12.
72. Germani, M., et al., *Choledochal cyst in pediatric patients: a 10-year single institution experience.* Acta Gastroenterol Latinoam, 2011. **41**(4): p. 302-7.
73. Wyszynski, D.F., C. Perandones, and R.D. Bennun, *Attitudes toward prenatal diagnosis, termination of pregnancy, and reproduction by parents of children with nonsyndromic oral clefts in Argentina.* Prenatal Diagnosis, 2003. **23**(9): p. 722-7.
74. Carvalho, M.H., et al., *Detection of fetal structural abnormalities at the 11-14 week ultrasound scan.* Prenat Diagn, 2002. **22**(1): p. 1-4.
75. Luiza, A., et al., *A descriptive epidemiology study of oral cleft in sergipe, Brazil.* Int Arch Otorhinolaryngol, 2013. **17**(4): p. 390-4.
76. Tannuri, A.C., et al., *Evolution of critically ill patients with gastroschisis from three tertiary centers.* Clinics (Sao Paulo, Brazil), 2011. **66**(1): p. 17-20.
77. Vilela, P.C., et al., *Risk factors for adverse outcome of newborns with gastroschisis in a Brazilian hospital.* Journal of Pediatric Surgery, 2001. **36**(4): p. 559-64.
78. Correa, C., et al., *Congenital malformations of pediatric surgical interest: prevalence, risk factors, and prenatal diagnosis between 2005 and 2012 in the capital city of a developing country. Bogota, Colombia.* Journal of Pediatric Surgery, 2014. **49**(7): p. 1099-103.
79. De Rovetto, C.R., et al., *Unilateral renal agenesis: Case review of ambulatory pediatric nephrology clinics in Cali.* Colombia Medica, 2010. **41**(1): p. 52-59.
80. Rosselli, P., et al., *Prenatal Ultrasound Diagnosis of Congenital Talipes Equinovarus in Bogota (Colombia) Between 2003 and 2012.* Iowa Orthopaedic Journal, 2015. **35**: p. 156-9.
81. Saldarriaga-Gil, W., et al., *Evaluation of prenatal diagnosis of congenital anomalies diagnosable by prenatal ultrasound in patients in neonatal intensive care units of Cali, Colombia.* Colombia Medica, 2014. **45**(1): p. 32-8.
82. Xie, H.N., et al., *Prenatal surveillance of bronchopulmonary sequestration using 3-dimensional ultrasonography.* J Ultrasound Med, 2009. **28**(8): p. 989-94.
83. Ghavami, M. and R. Abedinzadeh, *Prevalence of perinatal central nervous system anomalies in East azarbaijan-iran.* Iran J Radiol, 2011. **8**(2): p. 79-81.
84. Kutuk, M.S., et al., *Prenatal diagnosis of upper extremity malformations with ultrasonography: Diagnostic features and perinatal outcome.* J Clin Ultrasound, 2017. **45**(5): p. 267-276.
85. Savran, B., et al., *The importance of antenatal diagnosis of congenital duodenal obstruction.* Ir J Med Sci, 2016. **185**(3): p. 695-8.
86. Shalaby, H., et al., *Types and Outcome of Fetal Urinary Anomalies in Low Resource Setting Countries: A Retrospective Study.* J Obstet Gynaecol India, 2016. **66**(5): p. 316-20.

Confidential: For Review Only

87. Li, Z., et al., *Extremely high prevalence of neural tube defects in a 4-county area in Shanxi Province, China.* Birth Defects Research, 2006. **76**(4): p. 237-40.
88. Zhang, H., et al., *Retrospective study of prenatal diagnosed pulmonary sequestration.* Pediatric Surgery International, 2014. **30**(1): p. 47-53.
89. Kashyap, N., et al., *Early Detection of Fetal Malformation, a Long Distance Yet to Cover! Present Status and Potential of First Trimester Ultrasonography in Detection of Fetal Congenital Malformation in a Developing Country: Experience at a Tertiary Care Centre in India.* Journal of Pregnancy, 2015. **2015**: p. 623059.
90. Kumar, M., et al., *Prenatal sonographic evaluation and postnatal outcome of renal anomalies.* Indian J Hum Genet, 2012. **18**(1): p. 75-82.
91. Kumar, M., et al., *Fetal renal anomaly: factors that predict survival.* Journal of pediatric urology, 2014. **10**(6): p. 1001-7.
92. Hsieh, Y.Y., et al., *Accuracy of sonography in predicting the outcome of fetal congenital diaphragmatic hernia.* Zhonghua Yi Xue Za Zhi (Taipei), 2000. **63**(10): p. 751-7.
93. Jaruratanasirikul, S., et al., *Prevalence of neural tube defect in southern Thailand: a population-based survey during 2009-2012.* Childs Nervous System, 2014. **30**(7): p. 1269-75.
94. Gammeltoft, T., et al., *Late-term abortion for fetal anomaly: Vietnamese women's experiences.* Reproductive Health Matters, 2008. **16**(31 Suppl): p. 46-56.
95. Oztarhan, K., et al., *Prevalence and distribution of congenital abnormalities in Turkey: differences between the prenatal and postnatal periods.* Congenital Anomalies, 2010. **50**(4): p. 221-5.
96. Brizot, M.L., et al., *Conjoined twins pregnancies: experience with 36 cases from a single center.* Prenat Diagn, 2011. **31**(12): p. 1120-5.
97. Pelizzari, E., et al., *Characteristics of fetuses evaluated due to suspected anencephaly: a population-based cohort study in southern Brazil.* Sao Paulo Medical Journal = Revista Paulista de Medicina, 2015. **133**(2): p. 101-8.
98. Oloyede, O.A. and R.A. Oyedele, *Women's attitude to prenatal screening services for congenital abnormalities in Nigeria.* Journal of Obstetrics & Gynaecology, 2008. **28**(4): p. 406-7.
99. Acharya, P., et al., *Screening for birth defects strategies for developing low resource countries.* Donald School Journal of Ultrasound in Obstetrics and Gynecology, 2013. **7**(4): p. 453-461.
100. Neogi, S.B., *Abortion for fetal abnormalities in India: need for critical review.* Asia-Pacific Journal of Public Health, 2010. **22**(1): p. 146-7.
101. Phadke, S.R., M. Agarwal, and S. Aggarwal, *Late termination of pregnancy for fetal abnormalities: The perspective of Indian lay persons and medical practitioners.* Prenatal Diagnosis, 2011. **31**(13): p. 1286-1291.
102. Ranji, A. and A.K. Dykes, *Ultrasound screening during pregnancy in Iran: womens' expectations, experiences and number of scans.* Midwifery, 2012. **28**(1): p. 24-9.
103. Arawi, T. and A. Nassar, *Prenatally diagnosed foetal malformations and termination of pregnancy: the case of Lebanon.* Developing World Bioethics, 2011. **11**(1): p. 40-7.
104. Senanayake, H.M. and M.V. de Silva, *Prenatal diagnosis of lethal congenital malformations in Sri Lanka.* Ceylon Medical Journal, 2001. **46**(4): p. 163.
105. Hostiuc, S., O. Buda, and M. Hostiuc, *Late abortion. Attitudes amongst young physicians in Romania.* Archives of Gynecology & Obstetrics, 2013. **288**(2): p. 431-7.

Confidential: For Review Only

106. Lisker, R., A. Carnevale, and A.R. Villa, *Acceptance of induced abortion amongst medical students and physicians in Mexico.* Revista de Investigacion Clinica, 2006. **58**(4): p. 305-12.
107. Groisman, B., et al., *The use of a Toolkit for health needs assessment on neural tube defects in Argentina.* Journal of Community Genetics, 2013. **4**(1): p. 77-86.
108. Benute, G.R., et al., *Feelings of women regarding end-of-life decision making after ultrasound diagnosis of a lethal fetal malformation.* Midwifery, 2012. **28**(4): p. 472-5.
109. Mirlesse, V. and I. Ville, *The uses of ultrasonography in relation to foetal malformations in Rio de Janeiro, Brazil.* Social Science & Medicine, 2013. **87**: p. 168-75.
110. WHO. *WHO recommendation on early ultrasound in pregnancy*. The WHO Reproductive Health Library 2016; Available from: https://extranet.who.int/rhl/topics/preconception-pregnancy-childbirth-and-postpartum-care/antenatal-care/who-recommendation-early-ultrasound-pregnancy.
111. Todros, T., E. Capuzzo, and P. Gaglioti, *Prenatal diagnosis of congenital anomalies.* Images Paediatr Cardiol, 2001. **3**(2): p. 3-18.
112. WHO, *WHO Study Group on Training in Diagnostic Ultrasound: Essentials, Principles, and Standards*, WHO, Editor. 1998, World Health Organization: Philadelphia, Pennsylvania.
113. LaGrone, L.N., et al., *A review of training opportunities for ultrasonography in low and middle income countries.* Trop Med Int Health, 2012. **17**(7): p. 808-19.
114. Goldenberg, R.L., et al., *Routine antenatal ultrasound in low- and middle-income countries: first look - a cluster randomised trial.* BJOG, 2018. **125**(12): p. 1591-1599.
115. Franklin, H.L., et al., *Factors influencing referrals for ultrasound-diagnosed complications during prenatal care in five low and middle income countries.* Reprod Health, 2018. **15**(1): p. 204.
116. Wright, N.J. and C. Global PaedSurg Research, *Management and outcomes of gastrointestinal congenital anomalies in low, middle and high income countries: protocol for a multicentre, international, prospective cohort study.* BMJ Open, 2019. **9**(8): p. e030452.
117. Global Initiative for Children's Surgery, *Global Initiative for Children's Surgery: A Model of Global Collaboration to Advance the Surgical Care of Children.* World J Surg, 2019. **43**(6): p. 1416-1425.
118. Anyanwu, L.C., N. Ade-Ajayi, and U. Rolle, *Major abdominal wall defects in the low- and middle-income setting: current status and priorities.* Pediatr Surg Int, 2020. **36**(5): p. 579-590.

Confidential: For Review Only

Investigating the Use of Ultrasonography for the Antenatal Diagnosis of Structural Congenital Anomalies in Low- and Middle-Income Countries: A Systematic Review

Figure 1: Flowchart of the Screening Process

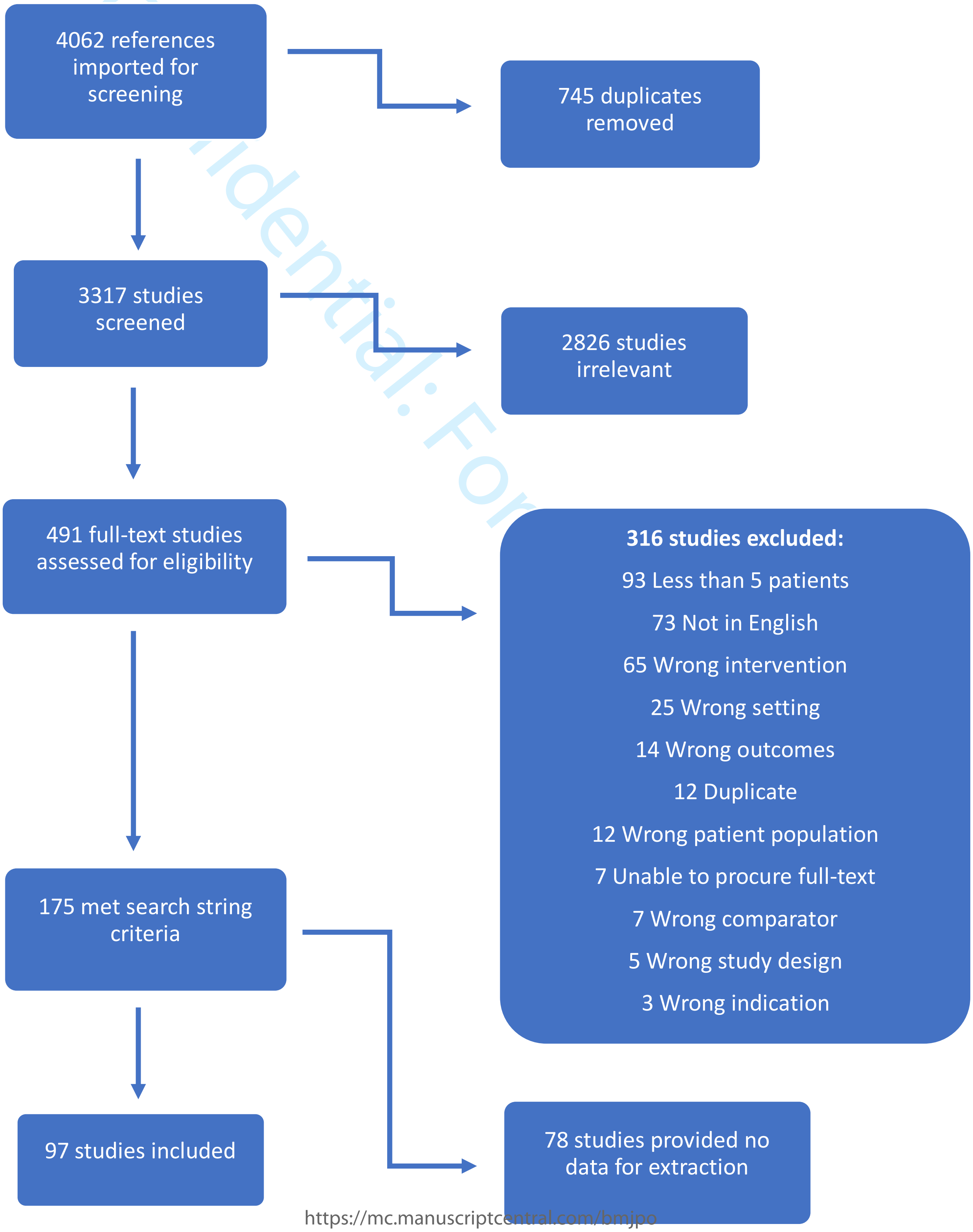

Investigating the Use of Ultrasonography for the Antenatal Diagnosis of Structural Congenital Anomalies in Low- and Middle-Income Countries: A Systematic Review

Figure 2: Regional Depiction of Articles included in the Systematic Review

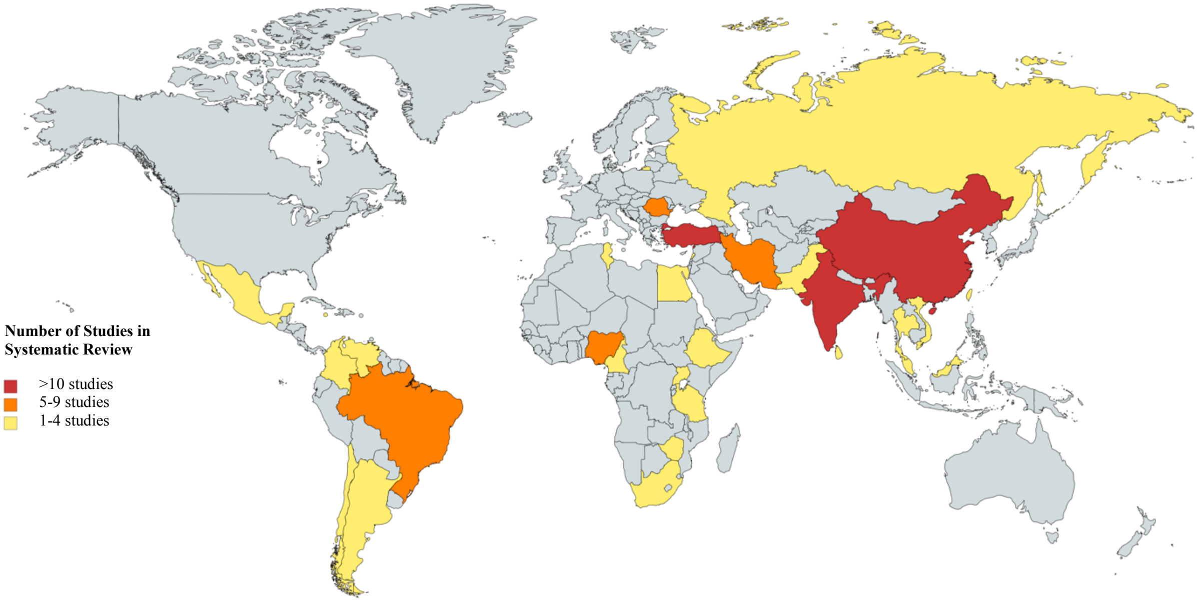

For Review Only

Supplementary File 1

Investigating the Use of Ultrasonography for the Antenatal Diagnosis of Structural Congenital Anomalies in Low- and Middle-Income Countries: A Systematic Review

PRISMA (Preferred Reporting Items for Systematic Reviews and Meta-Analyses) 2009 Checklist

Confidential: For Review Only

Section/topic	#	Checklist item	Section Heading
**TITLE**			
Title	1	Identify the report as a systematic review, meta-analysis, or both.	Title
**ABSTRACT**			
Structured summary	2	Provide a structured summary including, as applicable: background; objectives; data sources; study eligibility criteria, participants, and interventions; study appraisal and synthesis methods; results; limitations; conclusions and implications of key findings; systematic review registration number.	Abstract
**INTRODUCTION**			
Rationale	3	Describe the rationale for the review in the context of what is already known.	Introduction
Objectives	4	Provide an explicit statement of questions being addressed with reference to participants, interventions, comparisons, outcomes, and study design (PICOS).	Introduction
**METHODS**			
Protocol and registration	5	Indicate if a review protocol exists, if and where it can be accessed (e.g., Web address), and, if available, provide registration information including registration number.	Methodology
Eligibility criteria	6	Specify study characteristics (e.g., PICOS, length of follow-up) and report characteristics (e.g., years considered, language, publication status) used as criteria for eligibility, giving rationale.	Methodology
Information sources	7	Describe all information sources (e.g., databases with dates of coverage, contact with study authors to identify additional studies) in the search and date last searched.	Methodology
Search	8	Present full electronic search strategy for at least one database, including any limits used, such that it could be repeated.	Methodology
Study selection	9	State the process for selecting studies (i.e., screening, eligibility, included in systematic review, and, if applicable, included in the meta-analysis).	Methodology
Data collection process	10	Describe method of data extraction from reports (e.g., piloted forms, independently, in duplicate) and any processes for obtaining and confirming data from investigators.	Methodology
Data items	11	List and define all variables for which data were sought (e.g., PICOS, funding sources) and any assumptions and simplifications made.	Methodology

Risk of bias in individual studies	12	Describe methods used for assessing risk of bias of individual studies (including specification of whether this was done at the study or outcome level), and how this information is to be used in any data synthesis.	Methodology
Summary measures	13	State the principal summary measures (e.g., risk ratio, difference in means).	Methodology
Synthesis of results	14	Describe the methods of handling data and combining results of studies, if done, including measures of consistency (e.g., I^2) for each meta-analysis.	Methodology

From: Moher D, Liberati A, Tetzlaff J, Altman DG, The PRISMA Group (2009). Preferred Reporting Items for Systematic Reviews and Meta-Analyses: The PRISMA Statement. PLoS Med 6(6): e1000097. doi:10.1371/journal.pmed1000097

Confidential: For Review Only

Supplementary File 2

Investigating the Use of Ultrasonography for the Antenatal Diagnosis of Structural Congenital Anomalies in Low- and Middle-Income Countries: A Systematic Review

Search Strings

Confidential: For Review Only

Search String 1	Search String 2	Search String 3
Congenital Anomalies, Congenital Abnormalities, Congenital Malformation, Fetal Malformation, Birth Defects, Anencephaly, Conjoined Twins, Congenital Heart Defects, Anorectal Malformations, Anal Stenosis, Anal Atresia, Imperforate Anus, Biliary Atresia, Choledochal Cyst, Diaphragmatic Eventration, Esophageal Atresia, Tracheoesophageal Fistula, Intestinal Atresia, Duodenal Obstruction, Duodenal Atresia, Colonic Atresia, Malrotation, Apple Peel Syndrome, Congenital Diaphragmatic Hernias, Gastroschisis, Abdominal Wall Defects, Exomphalos, Omphalocele, Congenital Limb Deformities, Neural Tube Defects, Bronchogenic Cyst, Bronchopulmonary Sequestration, Congenital Cystic Adenomatoid Malformation of Lung, Renal Anomalies, Genito-urinary Anomalies, Maxillofacial Abnormalities, Mouth Abnormalities, Umbilical Hernia, Hirschsprung Disease, Ganglionic Megacolon, Rectosigmoid Aganglionosis, Colonic Aganglionosis, Intestinal Aganglionosis, Volvulus, Intestinal Volvulus	LMICs, Low- and Middle-Income Countries, Developing Countries, Low-Resource Settings, Underdeveloped Countries, Low-Income Countries, Middle-Income Countries, Limited Resource Settings, Africa South of the Sahara, Sub-Saharan Africa, Less Resourced Communities, Afghanistan, Albania, Algeria, American Samoa, Angola, Argentina, Armenia, Azerbaijan, Bangladesh, Belarus, Belize, Benin, Bhutan, Bolivia, Bosnia and Herzegovina, Botswana, Brazil, Bulgaria, Burkina Faso, Burundi, Cabo Verde, Cambodia, Cameroon, Central African Republic, Chad, China, Colombia, Comoros, Democratic Republic of the Congo, DRC, Republic of the Congo, Costa Rica, Cote d'Ivoire, Ivory Coast, Croatia, Cuba, Djibouti, Dominica, Dominican Republic, Ecuador, Egypt, El Salvador, Equatorial Guinea, Eritrea, Ethiopia, Fiji, Gabon, Gambia, Georgia, Ghana, Grenada, Guatemala, Guinea, Guinea-Bissau, Guyana, Haiti, Honduras, India, Indonesia, Islamic Republic of Iran, Iraq, Jamaica, Jordan, Kazakhstan, Kenya, Kiribati, Democratic People's Republic of Korea, Kosovo, Kyrgyz Republic, Lao PDR, Laos, Lebanon, Lesotho, Liberia, Libya, Macedonia Republic, Madagascar, Malawi, Malaysia, Maldives, Mali, Marshall Islands, Mauritania, Mauritius, Mexico, Micronesia, Moldova, Mongolia, Montenegro, Morocco, Mozambique, Myanmar, Namibia, Nauru, Nepal, Nicaragua, Niger, Nigeria, Pakistan, Panama, Papua New Guinea, Paraguay, Peru, Philippines, Romania, Russian Federation, Rwanda, Samoa, Sao Tome and Principe, Senegal, Serbia, Sierra Leone, Solomon Islands, Somalia, Somaliland, South Africa, South Sudan, Sri Lanka, Saint Lucia, Saint Vincent and the Grenadines, Sudan, Suriname, Swaziland, Syrian Arab Republic, Syria, Tajikistan, Tanzania, Thailand, Timor-Leste, East Timor, Togo, Tonga, Tunisia, Turkey, Turkmenistan, Tuvalu, Uganda, Ukraine, Uzbekistan, Vanuatu, Venezuela, Vietnam, West Bank and Gaza, Republic of Yemen, Zambia, Zimbabwe	Antenatal Diagnosis, Prenatal Diagnosis, Antenatal Screening, Prenatal Screening, Antenatal Ultrasound, Prenatal Ultrasound, Antenatal Ultrasonography, Prenatal Ultrasonography

Supplementary File 3

Investigating the Use of Ultrasonography for the Antenatal Diagnosis of Structural Congenital Anomalies in Low- and Middle-Income Countries: A Systematic Review

Search Strategy as Run on Ovid MEDLINE(R) Epub Ahead of Print, In-Process & Other Non-Indexed Citations, Ovid MEDLINE(R) Daily, Ovid MEDLINE and Versions(R) <1946 to June 27, 2018>

Confidential: For Review Only

No.	Search Terms	Results
1	exp Congenital Abnormalities/ or congenital anomal*.mp.	559922
2	exp Congenital Abnormalities/ or Congenital Abnormal*.mp.	555311
3	Congenital Malformation*.mp.	13826
4	Fetal Malformation*.mp.	1917
5	Birth Defect*.mp.	9122
6	Anencephaly.mp. or exp ANENCEPHALY/	3875
7	Conjoined Twins.mp. or exp Twins, Conjoined/	2022
8	exp Heart Defects, Congenital/ or Congenital Heart Defect*.mp.	142854
9	Anorectal Malformation*.mp.	1896
10	Anal Stenosis.mp.	389
11	Anal Atresia.mp. or exp Anus, Imperforate/	2815
12	Imperforate Anus.mp. or exp Anus, Imperforate/	2992
13	Biliary atresia.mp. or exp Biliary Atresia/	4482
14	exp Choledochal Cyst/ or Choledochal Cyst*.mp.	2825
15	exp Diaphragmatic Eventration/ or Diaphragmatic Eventration*.mp.	943
16	Esophageal Atresia.mp. or exp Esophageal Atresia/	4173
17	exp Tracheoesophageal Fistula/ or Tracheo-?esophageal fistula*.mp.	3676
18	Intestinal Atresia.mp. or exp Intestinal Atresia/	2301
19	Duodenal atresia.mp.	839
20	Colonic Atresia.mp.	152
21	Malrotation.mp.	2393
22	Apple peel syndrome.mp.	16
23	exp Hernias, Diaphragmatic, Congenital/ or congenital diaphragmatic hernia*.mp.	5530
24	Gastroschisis.mp. or exp GASTROSCHISIS/	2313
25	Abdominal Wall Defect*.mp.	2009
26	Exomphalos.mp. or exp Hernia, Umbilical/	3740
27	Omphalocele.mp. or exp Hernia, Umbilical/	4242
28	Congenital Limb Deformities.mp. or exp Limb Deformities, Congenital/	22483
29	exp Neural Tube Defects/ or Neural Tube Defect*.mp.	29051
30	exp Bronchogenic Cyst/ or Bronchogenic Cyst*.mp.	1810
31	exp Bronchopulmonary Sequestration/ or Bronchopulmonary sequestration*.mp.	2022
32	exp "Cystic Adenomatoid Malformation of Lung, Congenital"/ or Congenital Cystic adenomatoid malformation of lung*.mp.	874
33	Renal Anomal*.mp.	1185
34	exp Urogenital Abnormalities/ or Genito-urinary Anomalies.mp.	57735
35	exp Maxillofacial Abnormalities/ or Maxillofacial Abnormalit*.mp.	28265
36	exp Mouth Abnormalities/ or Mouth Abnormalit*.mp.	24459
37	Umbilical Hernia.mp. or exp Hernia, Umbilical/	4200
38	Hirschsprung disease.mp. or exp Hirschsprung Disease/	4567
39	exp Megacolon/ or Ganglionic Megacolon.mp.	7053
40	Rectosigmoid aganglionosis.mp.	15

41	Colonic Aganglionosis.mp.	311
42	Intestinal Aganglionosis.mp.	190
43	Volvulus.mp. or exp Intestinal Volvulus/	8927
44	LMICs.mp.	2001
45	(low- and middle-income countr*).mp. [mp=title, abstract, original title, name of substance word, subject heading word, floating sub-heading word, keyword heading word, protocol supplementary concept word, rare disease supplementary concept word, unique identifier, synonyms]	10910
46	Low-resource setting*.mp.	2719
47	Underdeveloped Countries.mp.	817
48	low-income countries.mp.	4707
49	exp Developing Countries/ or Developing countr*.mp.	115302
50	middle-income countries.mp.	11136
51	limited resource setting*.mp.	253
52	Africa South of the Sahara.mp. or exp "Africa South of the Sahara"/	184102
53	Sub-Saharan Africa.mp. or exp "Africa South of the Sahara"/	190396
54	Less Resourced communities.mp.	1
55	Afghanistan.mp. or exp AFGHANISTAN/	6065
56	Albania.mp. or exp ALBANIA/	1182
57	Algeria.mp. or exp ALGERIA/	3621
58	American Samoa.mp.	354
59	Angola.mp. or exp ANGOLA/	1403
60	Argentina.mp. or exp ARGENTINA/	19610
61	Armenia.mp. or exp ARMENIA/	1676
62	Azerbaijan.mp. or exp AZERBAIJAN/	1765
63	Bangladesh.mp. or exp BANGLADESH/	12907
64	Belarus.mp. or exp "Republic of Belarus"/	2553
65	Belize.mp. or exp BELIZE/	868
66	Benin.mp. or exp BENIN/	3441
67	exp BHUTAN/ or Bhutan.mp.	628
68	Bolivia.mp. or exp BOLIVIA/	3594
69	(Bosnia and Herzegovina).mp. [mp=title, abstract, original title, name of substance word, subject heading word, floating sub-heading word, keyword heading word, protocol supplementary concept word, rare disease supplementary concept word, unique identifier, synonyms]	2477
70	Botswana.mp. or exp BOTSWANA/	2247
71	Brazil.mp. or exp BRAZIL/	101919
72	Bulgaria.mp. or exp BULGARIA/	7554
73	Burkina Faso.mp. or exp Burkina Faso/	3904
74	Burundi.mp. or exp BURUNDI/	871
75	Cabo Verde.mp. or exp Cabo Verde/	211
76	Cambodia.mp. or exp CAMBODIA/	4102
77	Cameroon.mp. or exp CAMEROON/	6804
78	Central African Republic.mp. or exp Central African Republic/	1096
79	Chad.mp. or exp CHAD/	1144
80	China.mp. or exp CHINA/	205799
81	Colombia.mp. or exp COLOMBIA/	12533
82	Comoros.mp. or exp COMOROS/	427
83	Democratic Republic of the Congo.mp. or exp "Democratic Republic of the Congo"/	4506
84	exp "Democratic Republic of the Congo"/ or DRC.mp.	4827
85	Republic of the Congo.mp. or exp Congo/	6176
86	Costa Rica.mp. or exp Costa Rica/	5088
87	Cote d'Ivoire.mp. or exp Cote d'Ivoire/	3527
88	Ivory Coast.mp. or exp Cote d'Ivoire/	3432

Confidential: For Review Only

89	Croatia.mp. or exp CROATIA/	8261
90	Cuba.mp. or exp CUBA/	6343
91	Djibouti.mp. or exp DJIBOUTI/	372
92	Dominica.mp. or exp DOMINICA/	430
93	Dominican Republic.mp. or exp Dominican Republic/	2128
94	Ecuador.mp. or exp ECUADOR/	4722
95	exp EGYPT/ or Egypt.mp.	18702
96	El Salvador.mp. or exp El Salvador/	1319
97	Equatorial Guinea.mp. or exp Equatorial Guinea/	419
98	Eritrea.mp. or exp ERITREA/	518
99	Ethiopia.mp. or exp ETHIOPIA/	13354
100	Fiji.mp. or exp FIJI/	1620
101	Gabon.mp. or exp GABON/	1925
102	exp GAMBIA/ or Gambia.mp.	2958
103	exp "GEORGIA (REPUBLIC)"/ or exp GEORGIA/ or Georgia.mp.	15842
104	Ghana.mp. or exp GHANA/	9432
105	Grenada.mp. or exp GRENADA/	308
106	Guatemala.mp. or exp GUATEMALA/	3907
107	Guinea.mp. or exp GUINEA-BISSAU/ or exp EQUATORIAL GUINEA/ or exp GUINEA/ or exp PAPUA NEW GUINEA/ or exp NEW GUINEA/	160074
108	Guyana.mp. or exp GUYANA/	1136
109	Haiti.mp. or exp HAITI/	3707
110	Honduras.mp. or exp HONDURAS/	1736
111	India.mp. or exp INDIA/	129215
112	Indonesia.mp. or exp INDONESIA/	13107
113	exp Iran/ or Islamic Republic of Iran.mp.	21280
114	exp IRAQ/ or Iraq.mp.	9035
115	Jamaica.mp. or exp JAMAICA/	4427
116	Jordan.mp. or exp JORDAN/	6040
117	Kazakhstan.mp. or exp KAZAKHSTAN/	3153
118	Kenya.mp. or exp KENYA/	18798
119	Kiribati.mp. or exp Micronesia/	1934
120	Democratic People's Republic of Korea.mp. or exp "Democratic People's Republic of Korea"/	246
121	Kosovo.mp. or exp KOSOVO/	840
122	Kyrgyz Republic.mp. or exp Kyrgyzstan/	1225
123	exp Laos/ or Lao PDR.mp.	1876
124	Laos.mp. or exp LAOS/	2431
125	Lebanon.mp. or exp LEBANON/	5011
126	Lesotho.mp. or exp LESOTHO/	645
127	Liberia.mp. or exp LIBERIA/	1667
128	Libya.mp. or exp LIBYA/	1554
129	Macedonia Republic.mp. or exp "Macedonia (Republic)"/	475
130	Madagascar.mp. or exp MADAGASCAR/	4734
131	Malawi.mp. or exp MALAWI/	6317
132	Malaysia.mp. or exp MALAYSIA/	18390
133	Maldives.mp. or exp Indian Ocean Islands/	10595
134	Mali.mp. or exp MALI/	3511
135	Marshall Islands.mp. or exp Micronesia/	1959
136	Mauritania.mp. or exp MAURITANIA/	626
137	Mauritius.mp. or exp MAURITIUS/	953
138	exp MEXICO/ or Mexico.mp.	53282
139	Micronesia.mp. or exp MICRONESIA/	2243
140	Moldova.mp. or exp MOLDOVA/	920

Confidential: For Review Only

141	Mongolia.mp. or exp MONGOLIA/	3809
142	Montenegro.mp. or exp MONTENEGRO/	749
143	Morocco.mp. or exp MOROCCO/	6855
144	Mozambique.mp. or exp MOZAMBIQUE/	3236
145	Myanmar.mp. or exp MYANMAR/	3198
146	Namibia.mp. or exp NAMIBIA	1502
147	Nauru.mp. or exp Micronesia/	1921
148	Nepal.mp. or exp NEPAL/	9337
149	Nicaragua.mp. or exp NICARAGUA/	1949
150	exp NIGER/ or Niger.mp.	12299
151	Nigeria.mp. or exp NIGERIA/	32627
152	Pakistan.mp. or exp PAKISTAN/	20153
153	exp PANAMA/ or Panama.mp.	4265
154	Papua New Guinea.mp. or exp Papua New Guinea/	5003
155	Paraguay.mp. or exp PARAGUAY/	1433
156	Peru.mp. or exp PERU/	11249
157	Philippines.mp. or exp PHILIPPINES/	10698
158	Romania.mp. or exp ROMANIA/	11521
159	exp Russia/ or Russian Federation.mp.	51376
160	Rwanda.mp. or exp RWANDA/	2883
161	exp AMERICAN SAMOA/ or Samoa.mp. or exp SAMOA/ or exp "INDEPENDENT STATE OF SAMOA"/	1024
162	(Sao Tome and Principe).mp. [mp=title, abstract, original title, name of substance word, subject heading word, floating sub-heading word, keyword heading word, protocol supplementary concept word, rare disease supplementary concept word, unique identifier, synonyms]	135
163	Senegal.mp. or exp SENEGAL/	7067
164	Serbia.mp. or exp SERBIA/	4574
165	Sierra Leone.mp. or exp Sierra Leone/	2103
166	Solomon Islands.mp. or exp Melanesia/	6193
167	Somalia.mp. or exp SOMALIA/	1968
168	Somaliland.mp.	136
169	South Africa.mp. or exp South Africa/	47075
170	South Sudan.mp. or exp South Sudan/	397
171	Sri Lanka.mp. or exp Sri Lanka/	7342
172	Saint Lucia.mp. or exp Saint Lucia/	114
173	(Saint Vincent and the Grenadines).mp. [mp=title, abstract, original title, name of substance word, subject heading word, floating sub-heading word, keyword heading word, protocol supplementary concept word, rare disease supplementary concept word, unique identifier, synonyms]	57
174	exp SUDAN/ or Sudan.mp.	8429
175	Suriname.mp. or exp SURINAME/	1098
176	Swaziland.mp. or exp SWAZILAND/	818
177	Syrian Arab Republic.mp.	73
178	Syria.mp. or exp SYRIA/	2407
179	Tajikistan.mp. or exp TAJIKISTAN/	910
180	Tanzania.mp. or exp TANZANIA/	12921
181	Thailand.mp. or exp THAILAND/	32186
182	exp Timor-Leste/ or Timor-Leste.mp.	311
183	East Timor.mp.	187
184	Togo.mp. or exp TOGO/	1491
185	Tonga.mp. or exp TONGA/	463
186	Tunisia.mp. or exp TUNISIA/	9057
187	exp TURKEY/ or Turkey.mp.	46898

Confidential: For Review Only

188	Turkmenistan.mp. or exp TURKMENISTAN/	717
189	Tuvalu.mp. or exp Micronesia/	1884
190	Uganda.mp. or exp UGANDA/	13956
191	Ukraine.mp. or exp UKRAINE/	16702
192	Uzbekistan.mp. or exp UZBEKISTAN/	2161
193	Vanuatu.mp. or exp VANUATU/	629
194	Venezuela.mp. or exp VENEZUELA/	6620
195	exp VIETNAM/ or Vietnam.mp.	15919
196	(West Bank and Gaza).mp. [mp=title, abstract, original title, name of substance word, subject heading word, floating sub-heading word, keyword heading word, protocol supplementary concept word, rare disease supplementary concept word, unique identifier, synonyms]	194
197	Republic of Yemen.mp. or exp Yemen/	1264
198	Zambia.mp. or exp ZAMBIA/	5404
199	Zimbabwe.mp. or exp ZIMBABWE/	6879
200	Antenatal Diagnosis.mp.	2383
201	Prenatal Diagnosis.mp. or exp Prenatal Diagnosis/	74636
202	Antenatal Screening.mp.	1193
203	Prenatal Screening.mp.	2579
204	Antenatal Ultrasound.mp.	702
205	Prenatal Ultrasound.mp.	2203
206	Antenatal Ultrasonography.mp.	200
207	Prenatal Ultrasonography.mp. or exp Ultrasonography, Prenatal/	30427
208	1 or 2 or 3 or 4 or 5 or 6 or 7 or 8 or 9 or 10 or 11 or 12 or 13 or 14 or 15 or 16 or 17 or 18 or 19 or 20 or 21 or 22 or 23 or 24 or 25 or 26 or 27 or 28 or 29 or 30 or 31 or 32 or 33 or 34 or 35 or 36 or 37 or 38 or 39 or 40 or 41 or 42 or 43	607001
209	44 or 45 or 46 or 47 or 48 or 49 or 50 or 51 or 52 or 53 or 54 or 55 or 56 or 57 or 58 or 59 or 60 or 61 or 62 or 63 or 64 or 65 or 66 or 67 or 68 or 69 or 70 or 71 or 72 or 73 or 74 or 75 or 76 or 77 or 78 or 79 or 80 or 81 or 82 or 83 or 84 or 85 or 86 or 87 or 88 or 89 or 90 or 91 or 92 or 93 or 94 or 95 or 96 or 97 or 98 or 99 or 100 or 101 or 102 or 103 or 104 or 105 or 106 or 107 or 108 or 109 or 110 or 111 or 112 or 113 or 114 or 115 or 116 or 117 or 118 or 119 or 120 or 121 or 122 or 123 or 124 or 125 or 126 or 127 or 128 or 129 or 130 or 131 or 132 or 133 or 134 or 135 or 136 or 137 or 138 or 139 or 140 or 141 or 142 or 143 or 144 or 145 or 146 or 147 or 148 or 149 or 150 or 151 or 152 or 153 or 154 or 155 or 156 or 157 or 158 or 159 or 160 or 161 or 162 or 163 or 164 or 165 or 166 or 167 or 168 or 169 or 170 or 171 or 172 or 173 or 174 or 175 or 176 or 177 or 178 or 179 or 180 or 181 or 182 or 183 or 184 or 185 or 186 or 187 or 188 or 189 or 190 or 191 or 192 or 193 or 194 or 195 or 196 or 197 or 198 or 199 (1385344)	1385344
210	200 or 201 or 202 or 203 or 204 or 205 or 206 or 207	77525
211	208 and 209 and 210	896

Confidential: For Review Only

Supplementary File 4

Investigating the Use of Ultrasonography for the Antenatal Diagnosis of Structural Congenital Anomalies in Low- and Middle-Income Countries: A Systematic Review

Data Extraction Template

**Reference**	Reference (Full)
	Title
	Author(s)
	Publication Date
	Year of Publication
	Journal
**Place of Study**	Hospital
	City
	Country
	Continent
**Study Type**	Type of Study (RCT, Cohort, Case-Control, etc.)
**Publication Status**	Has Study Been Published?
**Study Population**	Number of Patients
	Patient Cohort (Institutional, Multi-Center, Regional, National, etc.)
	Patient Cohort (All Patients Presenting with a Condition vs. Those with an Antenatal Diagnosis)
	Gestational Age (Mean, Median)
	Type of Anomaly

Confidential: For Review Only

**Diagnosis**	Type of Ultrasound (Abdominal vs. Transvaginal; 2D vs. 3D)
	Percent Receiving Antenatal Ultrasound
	Percent with Any Anomaly Detected
	Percent with Accurate Antenatal Diagnosis
	Incidence of Anomaly
	Training of Ultrasonographer
	Referral Rate for Further Antenatal Care
	Referral Rate to Tertiary Clinic
	Percentage Born in a Pediatric Surgery Center
**Outcomes**	Mortality Rate in Live Births
	Termination Rate
	Complications (Using Clavien-Dindo Classification)
	Additional Notes Regarding the Effect of Antenatal Diagnosis on Outcomes
	Other Benefits of Antenatal Diagnosis (i.e., Parental Counselling, Option for Termination, etc.)
	Comments Regarding Policy
	Additional Notes/Other Useful Information

Confidential: For Review Only

Supplementary File 5

Investigating the Use of Ultrasonography for the Antenatal Diagnosis of Structural Congenital Anomalies in Low- and Middle-Income Countries: A Systematic Review

Table of Studies Excluded By Language

**Language**	**Number of Studies**
Chinese	23
Russian	16
Spanish	15
French	12
Portuguese	6
Romanian	1
**Total**	**73**

Confidential: For Review Only

Supplementary File 6

Investigating the Use of Ultrasonography for the Antenatal Diagnosis of Structural Congenital Anomalies in Low- and Middle-Income Countries: A Systematic Review

List of Countries Represented in Systematic Review Data Extraction

Country	No. of Studies (%)
India	17 (17.0%)
China	10 (10.0%)
Turkey	10 (10.0%)
Brazil	9 (9.0%)
Nigeria	9 (9.0%)
Romania	7 (7.0%)
Iran	6 (6.0%)
Argentina	4 (4.0%)
Colombia	4 (4.0%)
Thailand	4 (4.0%)
South Africa	2 (2.0%)
Cameroon	1 (1.0%)
Chile	1 (1.0%)
Egypt	1 (1.0%)
Ethiopia	1 (1.0%)
Jamaica	1 (1.0%)
Lebanon	1 (1.0%)
Malaysia	1 (1.0%)
Mexico	1 (1.0%)
Pakistan	1 (1.0%)
Russia	1 (1.0%)
Sri Lanka	1 (1.0%)
Taiwan	1 (1.0%)
Tanzania	1 (1.0%)
Tunisia	1 (1.0%)
Uganda	1 (1.0%)
Venezuela	1 (1.0%)
Vietnam	1 (1.0%)
Zimbabwe	1 (1.0%)
**Total**	**100**

This table represents all of the countries from the 97 included studies. 96 studies were conducted in a single country and 1 study was conducted across four countries.

Confidential: For Review Only
